# Comparative Efficacy of all Available Pharmaceutical Therapies for Moderate to Severe Crohn’s Disease: A Systematic Review and Network Meta-Analysis

**DOI:** 10.1016/j.gastha.2025.100764

**Published:** 2025-08-12

**Authors:** Matthijs Versteegh, Demy L. Idema, Simone Huygens, Kevin Jenniskens, Marieke Pierik, Tessa Römkens, Fiona van Schaik, Peter Wahab, Linde F. Huis in’t Veld, Mike Kusters, Kim van der Braak, Lotty Hooft, Johanna A.A. Damen

**Affiliations:** 1 Department of Research, Development & Medicines, National Health Care Institute, Diemen, The Netherlands; 2 Cochrane Netherlands, Julius Center for Health Sciences and Primary Care, University Medical Center Utrecht, Utrecht University, Utrecht, The Netherlands; 3 Department of Gastroenterology-Hepatology, Medical University Medical Centre, Maastricht, The Netherlands; 4 Department of Gastroenterology, Jeroen Bosch Hospital, 's-Hertogenbosch, The Netherlands; 5 Department of Gastroenterology and Hepatology, University Medical Centre Utrecht, Utrecht, The Netherlands; 6 Department of Gastroenterology, Rijnstate Hospital, Arnhem, The Netherlands

**Keywords:** Crohn’s disease, immunomodulators, network meta-analysis, biologic naive, biologic exposed

## Abstract

**Background and Aims:**

The therapeutic landscape for Crohn's disease (CD) has expanded, offering increased treatment possibilities, but with limited comparative evidence.

This study compares efficacy and discontinuation rates of all pharmaceutical therapies in moderate-to-severe CD.

**Methods:**

We conducted a systematic review (until October 2023) and network meta-analyses (NMAs) of phase-III randomized controlled trials for induction and maintenance of clinical remission and drug discontinuation rates. Frequentist NMA results and surface under the cumulative ranking (SUCRA) rankings were reported for immunomodulator (IM)-naive, biologic-naive and -exposed patients. Confidence in results was evaluated using Confidence in Network Meta-Analysis.

**Results:**

The search resulted in 3017 references, of which 77 randomized controlled trials from 1990 and later were included in the NMA. Networks were sparse and therapies had overlapping confidence intervals. The smaller IM-naive network and the larger biologic-naive network produced highly comparable relative risks. In biologic-naive patients, adalimumab (high induction regimen) had the highest SUCRA ranking for induction of clinical remission, and infliximab/azathioprine combination therapy had the highest SUCRA ranking for maintenance of remission. Among IMs, methotrexate had the highest ranking for induction and azathioprine for maintenance of remission. In biologic-exposed patients, upadacitinib had the highest SUCRA ranking for induction and maintenance of clinical remission, although for maintenance this finding may be biased due to the trial designs. Adverse event related discontinuation was numerically highest for methotrexate, azathioprine and upadacitinib. Confidence rating was moderate, low, or very low for most comparisons.

**Conclusion:**

This NMA including IMs, biologics and small molecules, suggests that anti–tumor necrosis factor (combination) therapy is most efficacious in biologic-naive patients and upadacitinib in biologic-exposed patients. Differences in relative risks were small and confidence intervals overlapping. Findings show that conventional therapies remain important in the treatment algorithm for CD patients.

## Introduction

Therapeutic options for the management of Crohn’s disease (CD) have expanded over the past decades. Since the approval of vedolizumab in 2014, several biologics and small molecules, together so called ‘advanced therapies’, have been added to the therapeutic armamentarium of patients with CD, including ustekinumab, risankizumab, and upadacitinib. Indeed, in the beginning of this century health outcomes were achieved with steroids, immunomodulators (IMs) and anti–tumor necrosis factor (TNF) therapy and surgery as rescue therapy if medical therapy failed. Nowadays, inflammatory bowel disease (IBD) specialists face the challenge of positioning drugs in a treatment pathway, simultaneously considering efficacy, disease phenotypes (eg, fistulizing disease), comorbidities and extra-intestinal manifestations. Shared-decision making has become of great importance, since adherence might be compromised if patient preferences are not considered. While the expanding therapeutic options are beneficial to patients, the societal impact of increasing costs of newly developed drugs creates urgency for IBD specialists to balance the potential benefits of these drugs against the less costly conventional therapies such as thiopurines and methotrexate.^1^ This requires insight into the relative efficacy of treatment options.

The efficacy of current available medical therapies for CD has been investigated in randomized controlled trials (RCTs), mostly compared to placebo rather than head-to-head to active comparators. As such, the comparative benefit relative to each other of all available therapies in CD is unknown, hampering clinical decision-making. Network meta-analyses (NMAs) can be used to address this evidence gap. NMAs estimate relative effects by identifying shared comparators of trials (such as placebo arms) creating a network of trials from which direct and indirect evidence can be derived. In CD, NMAs have generally focused on the comparative efficacy of advanced therapies including anti-TNF, anti-interleukin, anti-integrin agents, and Janus Kinase inhibitors,^2^, ^3^, ^4^ but there is paucity of data on how these therapies compare with conventional immunosuppressants such as thiopurines and methotrexate, particularly in moderate-to-severe CD. This comparison is increasingly important, as sustainability and cost control play a crucial role in the current health-care landscape, and advanced therapies have been identified as main cost drivers.^1^ We aimed to overcome the limitations of previous NMAs by explicitly comparing the full spectrum of available therapies for patients with moderate-to-severe CD.

## Materials and Methods

We registered our review with the international prospective register of systematic reviews (PROSPERO), (CRD42023468124) and adhered to the Preferred Reporting Items for Systematic reviews and Meta-Analyses guideline extension for network meta-analyses (NMAs).^5^

### Search Strategy and Selection of Studies

The Cochrane CENTRAL register was searched on October 18, 2023, using a combination of Medical Subject Headings terms and keywords for CD, pharmacological interventions and RCTs (Appendix 1). In addition, reference lists from relevant systematic reviews were reviewed to identify additional eligible studies. Reviewers (KvdB, DI, LHV, and MK) independently screened references in duplicate, first title and abstract and subsequently on full text. Disagreements were resolved by a third reviewer (KJ or JAAD) during title and abstract screening and through discussion or consulting a third reviewer during full text screening.

Articles were eligible for inclusion if they described parallel group RCTs on pharmacological treatments for adults (aged 18 years or older) with CD with or without previous treatment with biologics (Appendix 2). Relevant comparators were another eligible pharmacological intervention, placebo or a different dose of the same intervention. Studies including compounds that did not receive European Medicines Agency (EMA) approval for treatment of CD were also included. A priori exclusion was not considered appropriate as these studies may inform event rates in the comparator arm of the trial (often placebo or a registered treatment) and thus strengthen the network, regardless of the registration status of the included drugs. To be eligible, RCTs had to report at least one of the following outcomes: clinical remission, steroid-free remission, discontinuations, serious infections, malignancies or major cardiovascular events. As endoscopic response/remission was often not available as an end point in older trials, these outcomes were not extracted.

Induction studies were eligible for inclusion if they had a minimum treatment duration of 2 weeks, while maintenance studies were eligible for inclusion if there was a minimum treatment duration of 22 weeks. Phase III RCTs were included, as for these types there is the highest certainty that its design was sufficiently powered to establish both efficacy and adverse events, but randomized phase II RCTs were allowed if phase III was unavailable for a specific intervention that is used (off-label) in clinical practice. For maintenance studies, both treat-through and responder rerandomization studies were included.

### Data Extraction and Quality Assessment

Data extraction and risk of bias (RoB) assessment was performed by one out of five reviewers and verified by a second reviewer (KvdB, DI, LHV, MK, KJ, or JAAD). Disagreements were resolved through discussion between the two reviewers, or by group discussion. RoB was assessed using the Cochrane RoB 2 tool.^6^ Data were extracted on study design, study dates, eligibility criteria (including details on previous treatments), population characteristics (eg, age, sex, disease location, Crohn’s disease activity index (CDAI) score, previous surgery), details on interventions and comparators (eg, dosage, frequency, duration of treatment), sample size, outcome definitions, funding, and conflicts of interest. For all outcomes we extracted the number of patients with that outcome and the total number of patients analyzed for that outcome, separately for each study arm. For induction and maintenance treatment, outcomes measured at week 12 and week 52 of follow-up respectively, were extracted. If these were unavailable, the outcome with the nearest follow-up duration was taken.

### Transitivity Assumption and Subgroup Definitions

For trials to be combined in an NMA, the transitivity assumption must be met. This means that the distribution of effect modifiers must be equally distributed across studies. In CD, study characteristics have been shown to cause considerable heterogeneity in placebo remission rates.^7^ Notable design elements that influence these rates are study duration, number of study visits, and CDAI score at baseline, with study duration the most important. In multivariate analysis, Jairath et al.^8^ identified the number of study centers, drug class and concomitant IM use to be significant predictors of placebo response. For maintenance, another design element that may affect results is differential carryover effects from the induction treatment to the maintenance phase when comparing responder rerandomization and treat-through studies. For drugs whose efficacy is maintained after crossing over to placebo, this will result in a lower relative effect than for drugs whose efficacy is not maintained.

To account for these design elements, we require CDAI at inclusion to reflect moderate-to-severe CD, and selected subgroups of trials with the most similar follow-up. We also developed separate networks for IM naive, biologic-naive, and biologic-exposed. For the biologic-exposed subgroup, subgroup analyses of biologic-exposed patients were preferred, but for the minority of studies where these subgroups were not reported, studies were assigned to the exposed subgroup of the NMA when more than 60% of the trial population was exposed to biologics. When the percentage of biologics exposure was missing, studies before 1999 were all categorized as having biologic-naive populations and full text screening was used to allocate studies after 1999 to one of the defined subgroups. As the Harvey-Bradshaw Index (HBI) and CDAI are highly correlated and HBI can be transformed to CDAI,^9^ we allow for HBI to be an end point in the assessment of clinical remission. It should be noted that factors that explain heterogeneity in placebo response, do not necessarily also impact the relative risk (RR) between drugs when the risk factor is distributed equally across treatment arms. Therefore, we also estimate a full biologic-naive network not accounting for status of previous IM use and compare its results to the IM-naive network.

### Exclusions From Network Meta-analysis

Studies were excluded from the NMA if they focused solely on postoperative populations or if they had zero outcome events (as a continuity correction on this data would bias results and the zero-events occurred in subgroups with 10 or fewer patients^10^). No distinction was made between azathioprine and 6-mercaptopurine in the analyses of induction and maintenance of clinical remission, but these were kept separate for the analyses of discontinuation due to adverse events.

### End Points

Clinical remission was defined as CDAI<150. If both steroid-free and nonsteroid-free outcomes were available, steroid-free outcomes were preferred. Loss of remission during maintenance treatment was defined as CDAI>150. If CDAI was not available, the HBI and the cutoff points of the original studies for remission were used. Adverse events were analyzed as adverse event-related discontinuations in maintenance trials. In addition, specific adverse events of interest were serious infections, malignancies and major cardiovascular events during maintenance treatment.

### Analyses

Scenario analyses of the NMA were performed excluding trials with a high RoB or trials and using only studies with steroid-free end points to test robustness of findings.

The NMA was conducted using frequentist methods and random-effects models, presenting RRs. Inconsistency of direct and indirect evidence was assessed through the I-squared statistic, net-heat plot inspection and the Q-statistic for individual studies. The Cochrane handbook was followed for reporting inconsistency with the I-square statistic ordered in four groups: limited (0%–40%), moderate (30%–60%), substantial (50%–90%) and considerable heterogeneity (75%–100%).^11^ Rankings were based on surface under the cumulative ranking (SUCRA) curves.^12^ The SUCRA ranking represents the proportion of treatments worse than treatment for which the value is reported. League tables were created to allow inspection of all head-to-head comparisons.

Confidence in results was evaluated with the Confidence in Network Meta-Analysis (CINeMA) framework,^13^ specifically developed for NMA’s. Indirectness was scored on the percentage of biologics, follow-up duration and the use of composite end points. We used an RR of 0.8 and 1.25 as minimally important difference.

Analyses were conducted in R (version 4.1.3) with RStudio (2023.06.1 + 524) using the ‘netmeta’ (version 2.1–0) library.^14^

## Results

**Figure 1:**
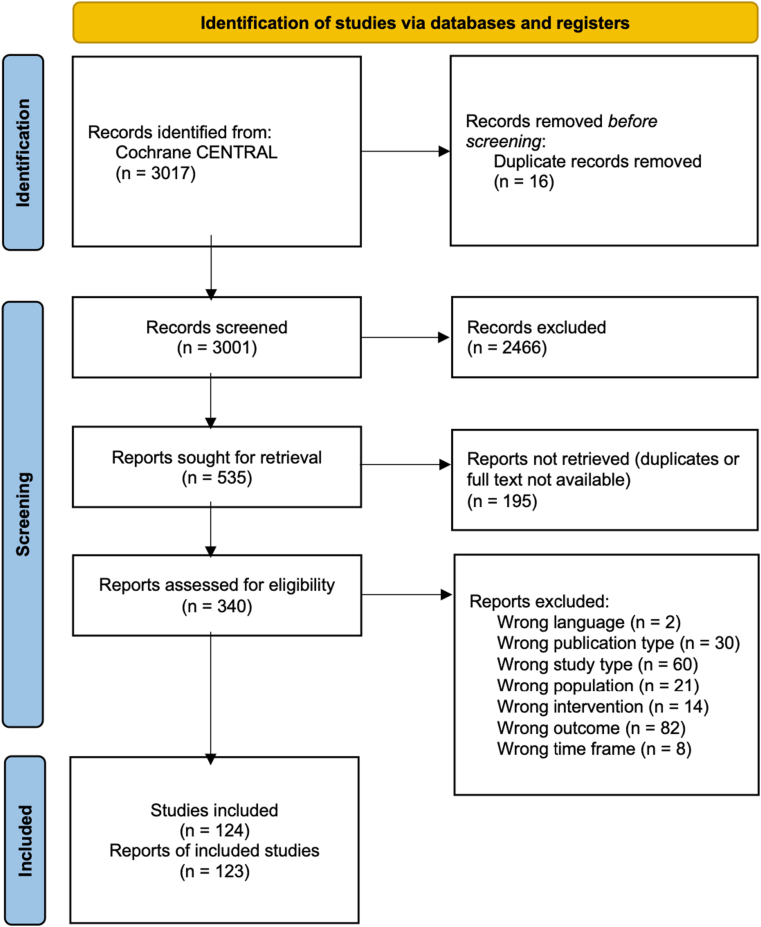
Prisma flow-chart. PRISMA, Preferred Reporting Items for Systematic reviews and Meta-Analyses.

The search strategy identified 3001 unique records of which 340 records were assessed on full text. After full text screening, 123 reports on 124 RCTs were included in the systematic literature review (Figure 1 and Appendix 3). The most frequent reasons for excluding articles on full text screening were discrepancies in outcome (n = 82), study type (n = 60) and publication type (n = 30) (Appendix 4). Appendix 5 describes the characteristics of included studies.

### Risk of Bias

Twenty studies (16%) were assessed to have a high overall RoB. Sixteen studies (13%) were judged to be at overall low RoB (Appendix 6). Most important reasons for scoring a high RoB were missing outcome data, selection of the reported result, and deviations from intended interventions. Eighty-six studies (55%) received industry sponsoring.

### Included Studies

For the NMA, a further 8 studies were excluded that did not report efficacy results as well as 6 phase II studies. Thirteen further studies were excluded as they focused solely on postoperative populations. Three studies^15^, ^16^, ^17^ were excluded as they included patients with mild CD rather than moderate to severe disease activity. One study with zero outcome events was excluded,^18^ and one study was excluded because it could not inform efficacy in the maintenance phase (the inclusion criterion was being stable on azathioprine for 4 years and hence represented a select subgroup).^19^

The remaining 92 trials were allocated to two categories: induction of clinical remission, and maintenance of clinical remission. Each category was stratified to the IM-naive network, biologic-naive and -exposed patients, yielding 6 subgroups. Fifteen studies did not report outcomes for solely biologic-naive or exposed subgroups. Thirteen studies were allocated to a subgroup based on the cutoff point of 60% exposure to biologics. For the maintenance biologic-naive subgroup, azathioprine dosages between 2 and 2.5 mg were pooled to link as many studies to the network as possible. The comparator in Mantzaris et al. (2009),^20^ budesonide 6–9 mg, was included as 6 mg to link to the network.

Of the 92 included studies, 13 were not connected to a network for any of the three outcomes in the naive or exposed subgroups. For the induction outcome in naive patients, both Targan-1997^21^ and Campieri-1997^22^ were a major source of inconsistency when inspecting direct versus indirect evidence, resulting in their exclusion from the main analyses. Therefore, the NMA was informed by 77 unique trials. Appendix 7 includes details of included studies.

### Results of the Network Meta-analysis

The main results of the NMA are described below. The appendices include network plots (Appendix 8), league tables (Appendix 9), SUCRA rankings (Appendix 10), direct versus indirect evidence plots (Appendix 11), scenario analyses (Appendix 12), and the analysis of adverse events (Appendices 13–15), and full CINeMA results (Appendix 16).

### Induction of Clinical Remission in Immunomodulator and Biologic-naive Patients

**Figure 2:**
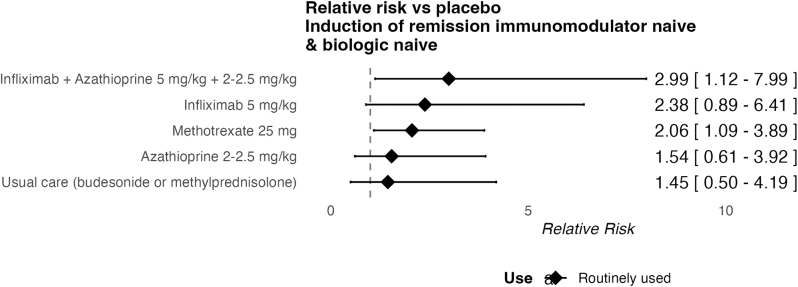
Forest plot of induction treatments in immunomodulator-naïve and biologic-naïve patients.

In total 9 out of 33 induction of clinical remission studies reported the proportion of previous IM use. Four studies included fully IM-naive patients and one study included less than <15% previous (see Appendix 7). Matsumoto et al.^23^ was not linked to the network. Therefore, 4 studies were included in the network with 6 therapies and 832 patients (Figure 2). Median follow-up was 13 weeks (interquartile range (IQR): 10-14). The network displayed no inconsistency. All studies reported steroid-free remission.

Infliximab + azathioprine and methotrexate significantly improved induction of remission compared to placebo with confidence intervals excluding 1. The comparison of infliximab monotherapy vs placebo was indirect, widening confidence intervals. The highest SUCRA ranking was for infliximab + azathioprine (0.95), followed by infliximab mono (0.74), methotrexate 25 mg (0.6), and azathioprine (0.3).

### Induction of Clinical Remission in Biologic-naive Patients

**Figure 3:**
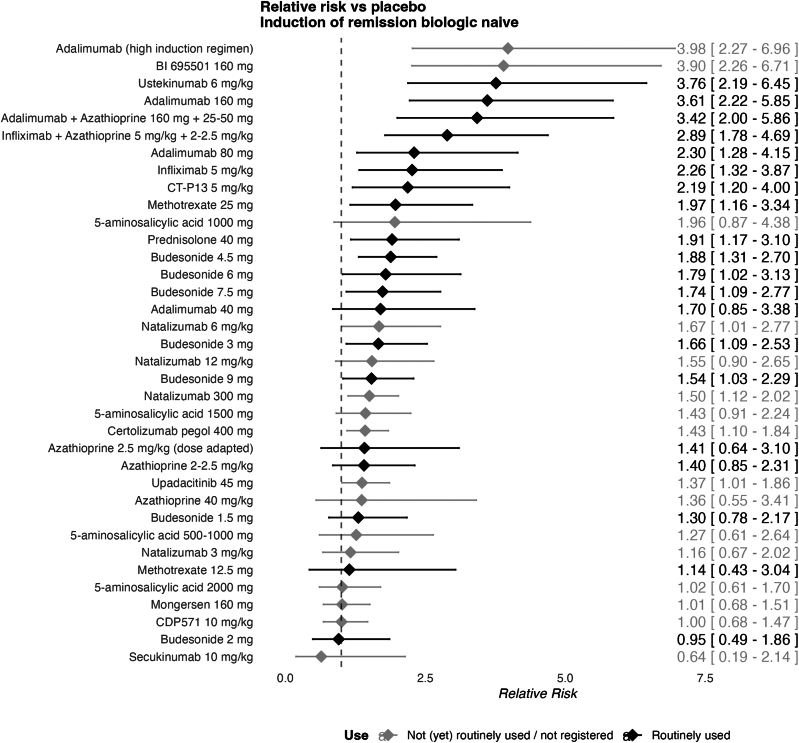
Forest plot of induction treatments in biologic-naive patients.

For the biologic-naive subgroup, 33 studies were included in the network, with 37 different therapies and 7504 patients, suggesting a sparse network (a limited number of studies for each treatment evaluated) (Figure 3). Median follow-up was 10 weeks (IQR: 8-12). The network displayed limited inconsistency (I^2^: 9.9%).

The following therapies significantly improved induction of remission compared to placebo in biologic-naive patients (RR > 1, with confidence intervals (CIs) excluding 1): adalimumab, BI695501 (adalimumab biosimilar), ustekinumab, adalimumab + azathioprine, infliximab + azathioprine, infliximab, CT-P13 (infliximab biosimilar), methotrexate, prednisolone, budesonide, natalizumab, certolizumab pegol, and upadacitinib. Adalimumab in a high induction regime (160 mg at weeks 0, 1, 2, and 3 followed by 40 mg from week 4 onwards) had the highest SUCRA score (0.94). The highest-ranking IM was methotrexate 25 mg with a SUCRA score of 0.64. The highest-ranking biologic other than anti-TNF was ustekinumab 6 mg/kg (0.93) (Appendix 10). The results show very limited difference with the IM naive network, granting credibility to the transitivity assumption for the full biologic-naive NMA. When compared against anti-TNF combination therapy, the CINeMA derived confidence rating was ‘moderate’ vs azathioprine 2–2.5mg, 5-ASA 1500mg, budesonide 1.5mg and 3mg, CDP571 10mg/kg certolizumab pegol 400mg, mongersen 160mg, natalizumab 3mg/kg, placebo, and upadacitinib 45mg. Other comparisons had ‘low’ to ‘very low’ confidence.

### Sensitivity Analyses

Excluding trials with a high RoB did not substantially influence the rank order of the RRs of therapies whose trials were not excluded.

Conducting the analysis in the subgroup of trials with only steroid-free end points decreased the certainty of superiority of treatments versus placebo, increased the ranking of budesonide, but did not affect the overall ordering of RRs (Appendix 12).

### Induction of Clinical Remission in Biologic-exposed Patients

**Figure 4:**
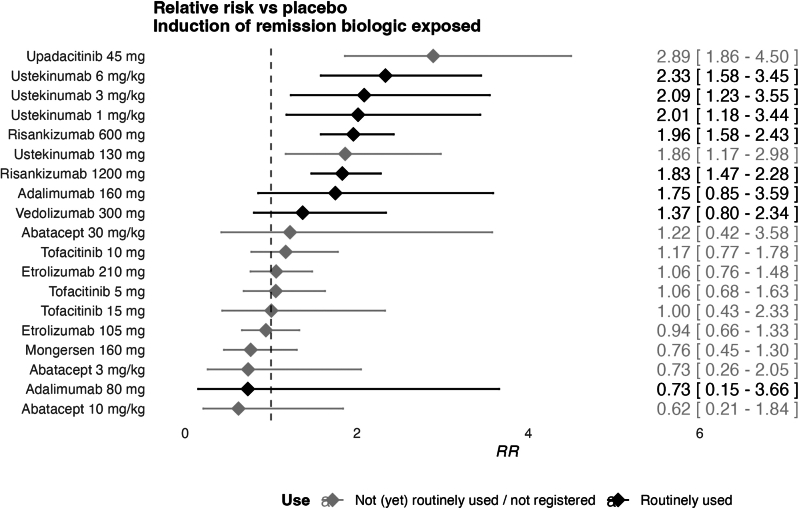
Forest plot of induction treatments in biologic-exposed patients.

For the biologic-exposed subgroup, 14 studies were included in the network, with 20 therapies and 5070 patients, suggesting a sparse network (Figure 4). Median follow-up was 10 weeks (IQR: 8-12). The network displayed no heterogeneity as it consisted mainly of direct evidence.

The following therapies significantly improved induction of remission compared to placebo in biologic-exposed patients (RR > 1 with confidence intervals excluding 1): upadacitinib, ustekinumab and risankizumab. Upadacitinib 45 mg had the highest SUCRA score (0.95). The highest-ranking anti-TNF was adalimumab 160/80/40 mg with a SUCRA score of 0.66. The highest-ranking biologic other than anti-TNF was ustekinumab 6 mg/kg (0.86) (Appendix 10). When compared against ustekinumab, the CINeMA derived confidence rating was ‘high’ vs placebo, ‘moderate’ vs mongersen 160mg and tofacitinib 5mg. Other comparisons had ‘low’ to ‘very low’ confidence.

### Sensitivity Analysis

No sensitivity analysis was performed for induction of clinical remission in the biologic-exposed subgroup because there were no trials at a high overall RoB. The ranking of RRs and the significant difference versus placebo of three trials with steroid-free outcomes was similar to the main analysis, but with a less pronounced numerical difference between adalimumab and vedolizumab (Appendix 12).

### Maintenance of Clinical Remission in Immunomodulator and Biologic-naive Patients

In total 5 out of 29 maintenance of clinical remission studies reported the proportion of previous IM use. In 2 studies, no or a small proportion (<3%) of patients was previously treated with IM.^24^^,^^25^ Direct evidence of the RR for losing clinical remission in these studies was: 0.57 [95% CI = 0.35–0.04] for methotrexate 15mg vs placebo and 0.67 [95% CI = 0.46–0.97] for infliximab vs usual care (70% IM use at week 52). It was not possible to form a network of the two studies with IM and biologic naive patients, therefore no NMA was performed.

### Maintenance of Clinical Remission in Biologic-naive Subgroup

**Figure 5:**
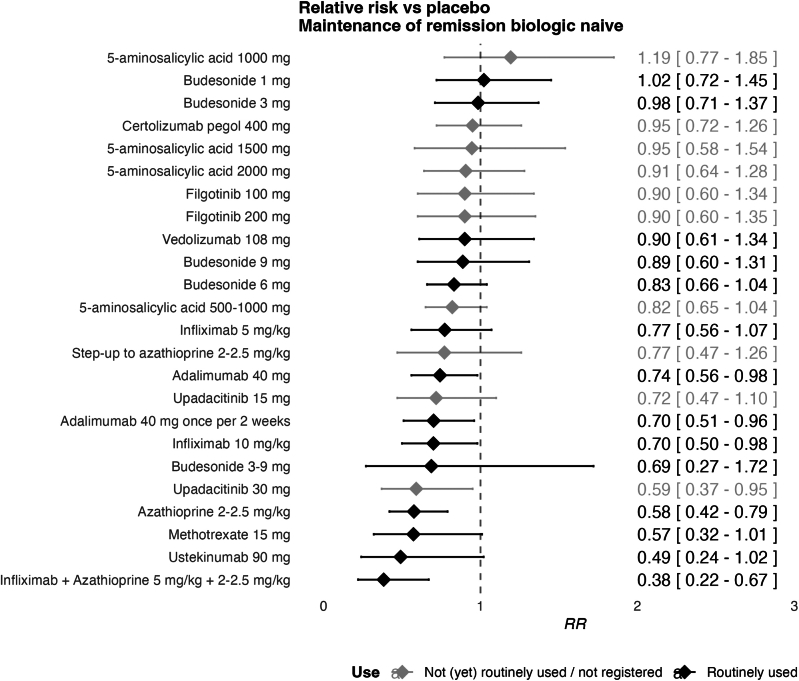
Forest plot of maintenance treatments in biologic-naive patients. Results represent ability to maintain remission relative to placebo, for those who achieved remission on the treatment in an induction setting.

For the biologic-naive subgroup 29 studies were included in the network, with 25 different therapies and 4630 patients, suggesting a sparse network (Figure 5). Median follow-up was 52 weeks (IQR: 47.5-52). The network displayed limited inconsistency (I^2^: 27.5%).

The following therapies significantly improved maintenance of remission compared to placebo in biologic-naive patients (RRs < 1 with confidence intervals excluding 1): infliximab with azathioprine, azathioprine, upadacitinib, adalimumab and infliximab. Infliximab 5 mg/kg combined with azathioprine 2–2.5 mg/kg had the highest SUCRA score (0.96). The highest-ranking IM was azathioprine 2–2.5 mg/kg monotherapy with a SUCRA score of 0.83. The highest-ranking biologic that was not anti-TNF was ustekinumab 90 mg (0.84) (Appendix 10). When compared against anti-TNF combination therapy, the CINeMA derived confidence rating was ‘moderate’ vs budesonide 1mg and 9mg and vs vedolizmab 108mg. Other comparisons had ‘low’ or ‘very low’ confidence.

### Sensitivity Analysis

For maintenance of clinical remission in the biologic-naive subgroup, excluding trials with a high overall RoB increased the ranking adalimumab 40 mg but with greater imprecision (RR 0.34 [95% CI 0.11-1.04]).

### Maintenance of Clinical Remission in Biologic-exposed Subgroup

**Figure 6:**
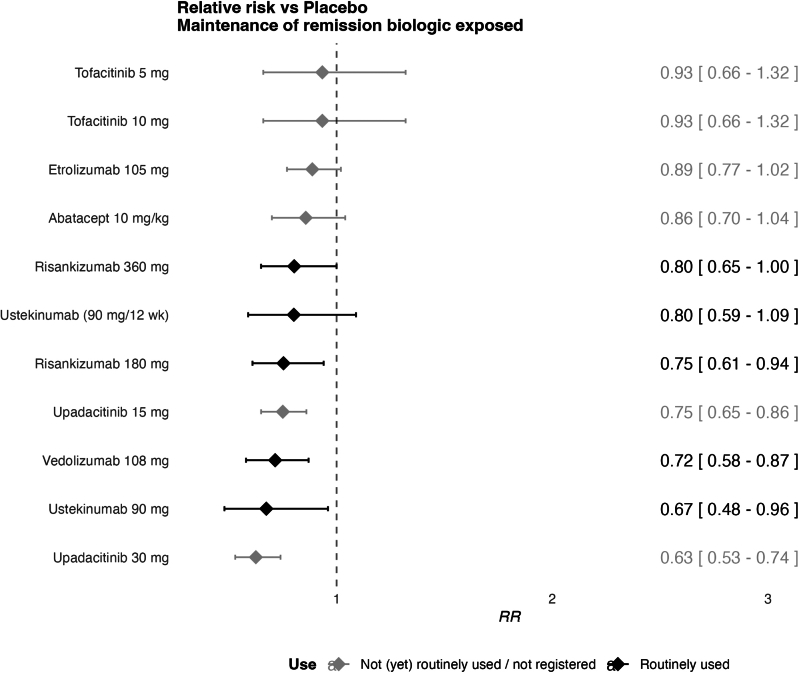
Forest plot of maintenance treatments in biologic-exposed patients. Results represent ability to maintain remission relative to placebo, for those who achieved remission on the treatment in an induction setting.

For the biologic-exposed subgroup seven studies were included in the network, with 12 treatments and 1909 patients, suggesting a sparse network (Figure 6). Median follow-up was 46 weeks (IQR: 41-52). The network displayed no heterogeneity.

The following therapies significantly improved maintenance of remission compared to placebo in biologic-exposed patients (RR < 1 with confidence intervals excluding 1): upadacitinib, ustekinumab, vedolizumab, and risankizumab. Upadacitinib 30mg had the highest SUCRA score (0.93). The highest-ranking biologic that was not anti-TNF was ustekinumab 90 mg with a SUCRA score of 0.76 (Appendix 10). When compared against ustekinumab 90mg, the CINeMA derived confidence rating was downgraded to ‘very low’ for all comparisons due to indirectness, imprecision and heterogeneity.

### Sensitivity Analysis

No sensitivity analysis was performed for maintenance of clinical remission in biologic-exposed subgroup because there were no trials with a high overall RoB.

### Responder-Rerandomized vs Treat Through Maintenance Studies

In the biologic-naive network, 7 studies were specified as responder rerandomization studies, of which only one included a biologic (ie adalimumab).^26^ Given that the efficacy of adalimumab in responders may carry over to those rerandomized to the placebo arm, it could be that the NMA underestimates the efficacy of adalimumab. In the biologic-exposed network, all included studies were responder rerandomization trials. The lowest placebo remission rate of 0.11 was observed for the only nonbiologic trial included in the NMA (ie upadactinib^27^). A meta-analysis of placebo arms of other studies, without the placebo arm of the upadacitinib trial, yielded a remission rate of 0.31. To account for this imbalance, a scenario analysis in which the event-rate was assumed equal to that of the meta-analysis for all placebo arms reduced the efficacy of upadacitinib vs placebo to a RR of 0.8 (95% CI: 0.65-0.97].

## Discussion

This study reports on the efficacy of pharmacological treatments for moderate to severe CD based on a NMA with evidence from 77 RCTs since 1990. We show that when IM-naive trials are pooled in a network with trials of which the previous IM-exposure status is unknown or known to be exposed but to an uncertain degree, there is a negligible decline in the RR when compared to the RRs of the IM-naive network. Thus, pooling studies in a large ‘biologic-naive’ network is feasible and does not demonstrate violations of the transitivity assumption. We demonstrate that all therapies that are in use for CD have overlapping confidence intervals, suggesting that no claims to superiority can be made based on current data synthesis. Nevertheless, we show that anti-TNF (combination) therapy has higher SUCRA rankings in biologic-naive patients than recently approved therapies, suggesting it is more likely to improve outcomes, but differences are small and uncertain. Methotrexate is the highest-ranking IM (10^th^) in the induction of clinical remission and azathioprine (3^rd^) in maintenance of clinical remission in biologic-naive patients, and its SUCRA score is consistent between the IM-naive and biologic-naive network. Upadacitinib is ranking highest in biologic-exposed patients for both induction and maintenance of clinical remission.

This study is the first NMA to report on the comparative efficacy of IMs relative to advanced therapies in moderate to severe CD. Findings show that thiopurines and methotrexate are effective in achieving and maintaining (steroid-free) clinical remission. Long-term efficacy of IMs is confirmed by observational studies.^28^^,^^29^ Tolerability and long-term safety are known issues with IMs causing discontinuation of methotrexate in the induction phase and a moderately increased risk of nonmelanoma skin cancer,^30^ and a moderately increased risk of cancer overall.^31^ Therapeutic drug monitoring could aid optimal use of this drug class. While there are also known long-term safety risk for sustained anti-TNF therapy, which moderately increases risk of melanoma in IBD patients,^32^ these need to be balanced against the unknown long-term risk of newer drug classes. Ongoing prospective cohort studies will shed more light on the risk-benefit of all currently available therapeutic options for CD.^33^ It is worth noting that the difference in costs of recently approved therapies compared to IMs or anti-TNF biosimilars is proportionally larger than the difference in clinical efficacy.

In accordance with previous studies, we also identify infliximab + azathioprine as the best performing compound. We found a smaller (but more certain) effect estimate for infliximab versus placebo, compared to that reported in Singh et al.,^3^ or infliximab versus azathioprine, compared to that reported in Hazelwood et al.^34^). The difference with the estimate of infliximab versus placebo from Singh et al. may be explained by the exclusion of Targan-1997 in our NMA. Targan-1997 is a relatively small but important study (n = 27, 28, 28, 24 in the four arms of the trial) comparing infliximab to placebo.^21^ We excluded Targan-1997 because it increased inconsistency from an I^2^ of 9.9%–19.3%. This was due to a higher efficacy of infliximab versus placebo in Targan-1997 than expected from indirect evidence in our NMA. This may be explained by the placebo event rates reported in Targan-1997: 17% for response and 4% for remission, considerably lower than those identified in a systematic review of placebo event rates (response 28% [95% CI: 24%–32%] and remission 18% [95% CI: 16%–21%]).^8^ In contrast to our NMA, Singh et al. included Targan-1997, which increased their efficacy estimate for infliximab. This was demonstrated by the large difference between the effect estimate for infliximab from Targan-1997 (OR = 18.4 CI 2.16-156.58) and another RCT evaluating infliximab^16^ (OR = 5.03 (CI 2.25–11.22).^2^ These findings suggest that while Targan-1997 may have good internal validity, the transitivity assumption of no relevant differences between studies besides the intervention may not hold.

A difference with the NMA of Hazelwood et al. is that their study reports no overlapping confidence intervals for the RRs of infliximab versus azathioprine for the induction of clinical remission. This may be caused by several differences: Hazelwood et al. included Targan-1997, studies that reported laboratory end points other than CDAI reduction (2 studies), studies that were not in moderate to severe Crohn patients (3 studies), and studies that did not differentiate between remission and response (1 study).

Following the cutoff date for the search, other evidence has emerged that could have impact on the estimates of this NMA: SEQUENCE, VIVID-1, and GALAXI-2 and 3. The SEQUENCE trial was a noninferiority study comparing ustekinumab and risankizumab standard dosing in biologic-exposed patients, demonstrating noninferiority, but numerically stronger results for risankizumab.^35^ Including this comparison would increase the RR of induction of clinical remission (maintenance of remission) in exposed patients to 2.21 [95% CI 1.76-2.78] (0.67 [95% CI 0.39-1.14 for maintenance) risankizumab vs 1.71 [95% CI 1.29; 2.25] (0.84 [95% CI 0.48-1.47] for maintenance) for ustekinumab, thus, suggesting improved relative efficacy of risankizumab but with overlapping confidence intervals. VIVID-1 demonstrated superiority of mirikizumab (EMA approval end 2024) over placebo.^36^ Ustekinumab was included in VIVID-1, but results were sparsely reported. The GALAXI trials compare guselkumab (EMA approval may 2025) with ustekinumab and placebo in patients who had inadequate response or tolerance of IMs or biologic therapies.^37^ The trial demonstrated superiority of guselkumab on several end points, but not clinical remission, the end point utilized in this NMA. For the GALAXI trial only conference abstracts are available hindering uptake in this NMA.

### Strengths and Limitations

This systematic literature review with NMA provides the most recent update of all available phase III RCT evidence on treatment for CD. Unique to our study was the comparison of relative efficacy between all treatments available for moderate-to-severe CD, including older IMs. The inclusion criteria were set to include all relevant RCTs irrespective of Food and Drug Administration/EMA approval of the compound. This allowed us to draw insights from many more studies which strengthened the network and consequently the estimates of our NMA. In this study, optimal transitivity was sought through adopting strict inclusion criteria, using similar end point definitions and study follow-up time horizons, as well as by creating different subgroups for IM and biologic exposure status. We repeated analyses for different RoB outcomes and steroid use with similar results as the base case analysis. Our results suggest that previous exposure to IMs does not affect the transitivity assumption: the results of the IM naive network are similar to the results of the biologic-naive network (including both IM naive and exposed patient groups). Furthermore, the Study of Biologic and Immunomodulater Naive Patients in Crohn's Disease (SONIC) trial (which included patients naive to IMs and biologics) week 10 remission outcomes are highly similar to the induction of clinical remission results from our NMA, even though the NMA includes a majority of evidence from studies in which included patients had prior exposure to IMs.

Our study also has several limitations. First, we reported estimates of clinical remission as measured by CDAI and HBI that have demonstrated a poor correlation with mucosal inflammation.^38^ However, it was not possible to report on endoscopic remission or other biomarkers because these outcomes are generally not available from trials in the prebiologic era.

Second, as newer therapies are most often compared against placebo in RCTs, in this NMA the majority of comparisons against an older active comparator are based on indirect evidence in sparse networks where the number of treatments was often higher or similar to the number of studies. The variance of indirect evidence is the sum of the variance of the direct evidence and hence is likely to produce wider confidence intervals. The sparsity of the networks and the wide confidence intervals suggest that any result must be interpreted with caution, as holds true for all NMA’s in CD.

Third, not all trials report biologic-naive or biologic-exposed subgroups. We defined a cutoff point to define a mixed-population as being ‘exposed’ or ‘naive’. While the authors agreed that a cutoff point of more than half the sample (60%) was appropriate, it remains an arbitrary selection. However, this only affected 13 of the included studies. In the IM-naive network, none of the trials was affected by this allocation. In the biologic-naive subgroup for induction of remission, 7 trials were allocated based on the cutoff and had a mean exposure rate of 21.4%. For biologic-exposed induction two trials were allocated and had a mean exposure rate of 68.2%. In the biologic-naive maintenance subgroup four trials were allocated and had a mean exposure rate of 48% (these trials compared filgotinib, certolizumab, and adalimumab with placebo), and one study in the biologic-exposed maintenance subgroup with an exposure rate of 60%.

Fourth, we excluded studies without events in one of the arms. While common for frequentist NMAs, such an exclusion is not required for Bayesian approaches to NMAs.

Fifth, the NMA for the end point discontinuation due to adverse events yielded wide confidence intervals due to a low frequency of events and hence was not as informative as we had hoped for the analysis of adverse events. Post marketing surveillance studies reveal rare safety events years after registration of new drugs.^39^ If, as demonstrated in this NMA, there are no large differences in efficacy, the limited information on safety may cause clinicians to favor prescription of drugs for which extensive real world safety data is available.

A last limitation is related to the responder-rerandomization trial design for maintenance studies. Notably in the biologic-exposed subgroup, all but one of the included therapies is biologics that are expected to maintain efficacy after withdrawal, except for upadacitinib. Upadacitinib is a Janus Kinase 1 inhibitor with a half-life of 9–14 hours^40^ that may not have sustained efficacy after withdrawal. Hence, the upadacitinib trial is the only trial for which the responder-rerandomization does not impact placebo rates, giving it a favorable result relative to the comparators in that network.

## Conclusion

Our study demonstrates that recently approved therapies do not rank highest for the induction or maintenance of clinical remission, except in biologic-exposed patients, and even then, numerical differences are small and confidence intervals overlap. The relatively high efficacy and low costs, of IMs and anti-TNF (combination) regimens suggests that recently approved therapies best serve the population who have loss of response or adverse events on at least one biologic. The highly uncertain discontinuation due to adverse rates was numerically unfavorable for methotrexate, azathioprine and upadacitinib, which requires vigilance. Experience of health care professionals with a drug class and patient preferences should be considered when selecting a treatment for an individual patient. Health economic analyses ought to be conducted to investigate the balance between costs and effects of different treatment sequences in CD.

## Supplementary data

Appendix

Extended PDF
