# Supplementary material for: Comparative Efficacy of all Available Pharmaceutical Therapies for Moderate to Severe Crohn’s Disease: A Systematic Review and Network Meta-Analysis
Source: Gastro Hep Adv. 2025 Aug 12;5(1):100764. doi: 10.1016/j.gastha.2025.100764 (PMC12546778; doi:10.1016/j.gastha.2025.100764)
Supplement: Extended PDF [file mmc2.pdf]

Gastro Hep Advances 2026;5:100764

SYSTEMATIC REVIEWS AND META-ANALYSIS

Comparative Efficacy of all Available Pharmaceutical Therapies for Moderate to Severe Crohn's Disease: A Systematic Review and Network Meta-Analysis

Matthijs Versteegh,[1] Demy L. Idema,[2] Simone Huygens,[1] Kevin Jenniskens,[2] Marieke Pierik,[3] Tessa Römkens,[4] Fiona van Schaik,[5] Peter Wahab,[6] Linde F. Huis in't Veld,[2] Mike Kusters,[2] Kim van der Braak,[2] Lotty Hooft,[2] and Johanna A. A. Damen[2]

[1]*Department of Research, Development & Medicines, National Health Care Institute, Diemen, The Netherlands;* [2]*Cochrane Netherlands, Julius Center for Health Sciences and Primary Care, University Medical Center Utrecht, Utrecht University, Utrecht, the Netherlands;* [3]*Department of Gastroenterology-Hepatology, Medical University Medical Centre, Maastricht, The Netherlands;* [4]*Department of Gastroenterology, Jeroen Bosch Hospital, 's-Hertogenbosch, The Netherlands;* [5]*Department of Gastroenterology and Hepatology, University Medical Centre Utrecht, Utrecht, The Netherlands; and* [6]*Department of Gastroenterology, Rijnstate Hospital, Arnhem, The Netherlands*

BACKGROUND AND AIMS: The therapeutic landscape for Crohn's disease (CD) has expanded, offering increased treatment possibilities, but with limited comparative evidence. This study compares efficacy and discontinuation rates of all pharmaceutical therapies in moderate-to-severe CD. **METHODS:** We conducted a systematic review (until October 2023) and network meta-analyses (NMAs) of phase-III randomized controlled trials for induction and maintenance of clinical remission and drug discontinuation rates. Frequentist NMA results and surface under the cumulative ranking (SUCRA) rankings were reported for immunomodulator (IM)-naive, biologic-naive and -exposed patients. Confidence in results was evaluated using Confidence in Network Meta-Analysis. **RESULTS:** The search resulted in 3017 references, of which 77 randomized controlled trials from 1990 and later were included in the NMA. Networks were sparse and therapies had overlapping confidence intervals. The smaller IM-naive network and the larger biologic-naive network produced highly comparable relative risks. In biologic-naive patients, adalimumab (high induction regimen) had the highest SUCRA ranking for induction of clinical remission, and infliximab/azathioprine combination therapy had the highest SUCRA ranking for maintenance of remission. Among IMs, methotrexate had the highest ranking for induction and azathioprine for maintenance of remission. In biologic-exposed patients, upadacitinib had the highest SUCRA ranking for induction and maintenance of clinical remission, although for maintenance this finding may be biased due to the trial designs. Adverse event related discontinuation was numerically highest for methotrexate, azathioprine and upadacitinib. Confidence rating was moderate, low, or very low for most comparisons. **CONCLUSION:** This NMA including IMs, biologics and small molecules, suggests that anti–tumor necrosis factor (combination) therapy is most efficacious in biologic-naive patients and upadacitinib in biologic-exposed patients. Differences in relative risks were small and confidence intervals overlapping. Findings show that conventional therapies remain important in the treatment algorithm for CD patients.

Keywords: Crohn's disease; immunomodulators; network meta-analysis; biologic naive; biologic exposed

Introduction

Therapeutic options for the management of Crohn's disease (CD) have expanded over the past decades. Since the approval of vedolizumab in 2014, several biologics and small molecules, together so called 'advanced therapies', have been added to the therapeutic armamentarium of patients with CD, including ustekinumab, risankizumab, and upadacitinib. Indeed, in the beginning of this century health outcomes were achieved with steroids, immunomodulators (IMs) and anti–tumor necrosis factor (TNF) therapy and surgery as rescue therapy if medical therapy failed. Nowadays, inflammatory bowel disease (IBD) specialists face the challenge of positioning drugs in a treatment pathway, simultaneously considering efficacy, disease phenotypes (eg, fistulizing disease), comorbidities and extra-intestinal manifestations. Shared-decision making has become of great importance, since adherence might be compromised if patient preferences are not considered.

Abbreviations used in this paper: **CD, Crohn's disease; CDAI, Crohn's Disease Activity Index; CINEMA, Confidence in Network Meta-Analysis; EMA, European Medicines Agency; HBI, Harvey-Bradshaw Index; IBD, Inflammatory bowel disease; IM, Immunomodulator; IQR, Interquartile Range; NMA, Network meta-analysis; RCT, Randomized controlled trial; RoB, Risk of Bias; RR, Relative risk; SUCRA, Surface under the cumulative ranking; TNF, Tumor necrosis factor.**

Most current article

Copyright © 2025 The Authors. Published by Elsevier Inc. on behalf of the AGA Institute. This is an open access article under the CC BY license (http://creativecommons.org/licenses/by/4.0/).
2772-5723
https://doi.org/10.1016/j.gastha.2025.100764

While the expanding therapeutic options are beneficial to patients, the societal impact of increasing costs of newly developed drugs creates urgency for IBD specialists to balance the potential benefits of these drugs against the less costly conventional therapies such as thiopurines and methotrexate.[1] This requires insight into the relative efficacy of treatment options.

The efficacy of current available medical therapies for CD has been investigated in randomized controlled trials (RCTs), mostly compared to placebo rather than head-to-head to active comparators. As such, the comparative benefit relative to each other of all available therapies in CD is unknown, hampering clinical decision-making. Network meta-analyses (NMAs) can be used to address this evidence gap. NMAs estimate relative effects by identifying shared comparators of trials (such as placebo arms) creating a network of trials from which direct and indirect evidence can be derived. In CD, NMAs have generally focused on the comparative efficacy of advanced therapies including anti-TNF, anti-interleukin, anti-integrin agents, and Janus Kinase inhibitors,[2–4] but there is paucity of data on how these therapies compare with conventional immunosuppressants such as thiopurines and methotrexate, particularly in moderate-to-severe CD. This comparison is increasingly important, as sustainability and cost control play a crucial role in the current health-care landscape, and advanced therapies have been identified as main cost drivers.[1] We aimed to overcome the limitations of previous NMAs by explicitly comparing the full spectrum of available therapies for patients with moderate-to-severe CD.

Materials and Methods

We registered our review with the international prospective register of systematic reviews (PROSPERO), (CRD42023468124) and adhered to the Preferred Reporting Items for Systematic reviews and Meta-Analyses guideline extension for network meta-analyses (NMAs).[5]

Search Strategy and Selection of Studies

The Cochrane CENTRAL register was searched on October 18, 2023, using a combination of Medical Subject Headings terms and keywords for CD, pharmacological interventions and RCTs (Appendix 1). In addition, reference lists from relevant systematic reviews were reviewed to identify additional eligible studies. Reviewers (KvdB, DI, LHV, and MK) independently screened references in duplicate, first title and abstract and subsequently on full text. Disagreements were resolved by a third reviewer (KJ or JAAD) during title and abstract screening and through discussion or consulting a third reviewer during full text screening.

Articles were eligible for inclusion if they described parallel group RCTs on pharmacological treatments for adults (aged 18 years or older) with CD with or without previous treatment with biologics (Appendix 2). Relevant comparators were another eligible pharmacological intervention, placebo or a different dose of the same intervention. Studies including compounds that did not receive European Medicines Agency (EMA) approval for treatment of CD were also included. A priori exclusion was not considered appropriate as these studies may inform event rates in the comparator arm of the trial (often placebo or a registered treatment) and thus strengthen the network, regardless of the registration status of the included drugs. To be eligible, RCTs had to report at least one of the following outcomes: clinical remission, steroid-free remission, discontinuations, serious infections, malignancies or major cardiovascular events. As endoscopic response/remission was often not available as an end point in older trials, these outcomes were not extracted.

Induction studies were eligible for inclusion if they had a minimum treatment duration of 2 weeks, while maintenance studies were eligible for inclusion if there was a minimum treatment duration of 22 weeks. Phase III RCTs were included, as for these types there is the highest certainty that its design was sufficiently powered to establish both efficacy and adverse events, but randomized phase II RCTs were allowed if phase III was unavailable for a specific intervention that is used (off-label) in clinical practice. For maintenance studies, both treat-through and responder rerandomization studies were included.

Data Extraction and Quality Assessment

Data extraction and risk of bias (RoB) assessment was performed by one out of five reviewers and verified by a second reviewer (KvdB, DI, LHV, MK, KJ, or JAAD). Disagreements were resolved through discussion between the two reviewers, or by group discussion. RoB was assessed using the Cochrane RoB 2 tool.[6] Data were extracted on study design, study dates, eligibility criteria (including details on previous treatments), population characteristics (eg, age, sex, disease location, Crohn's disease activity index (CDAI) score, previous surgery), details on interventions and comparators (eg, dosage, frequency, duration of treatment), sample size, outcome definitions, funding, and conflicts of interest. For all outcomes we extracted the number of patients with that outcome and the total number of patients analyzed for that outcome, separately for each study arm. For induction and maintenance treatment, outcomes measured at week 12 and week 52 of follow-up respectively, were extracted. If these were unavailable, the outcome with the nearest follow-up duration was taken.

Transitivity Assumption and Subgroup Definitions

For trials to be combined in an NMA, the transitivity assumption must be met. This means that the distribution of effect modifiers must be equally distributed across studies. In CD, study characteristics have been shown to cause considerable heterogeneity in placebo remission rates.[7] Notable design elements that influence these rates are study duration, number of study visits, and CDAI score at baseline, with study duration the most important. In multivariate analysis, Jairath et al.[8] identified the number of study centers, drug class and concomitant IM use to be significant predictors of placebo response. For maintenance, another design element that may affect results is differential carryover effects from the induction treatment to the maintenance phase when comparing responder rerandomization and treat-through studies. For drugs whose efficacy is maintained after crossing over to placebo, this will result in a lower relative effect than for drugs whose efficacy is not maintained.

To account for these design elements, we require CDAI at inclusion to reflect moderate-to-severe CD, and selected subgroups of trials with the most similar follow-up. We also developed separate networks for IM naive, biologic-naive, and biologic-exposed. For the biologic-exposed subgroup, subgroup analyses of biologic-exposed patients were preferred, but for the minority of studies where these subgroups were not reported, studies were assigned to the exposed subgroup of the NMA when more than 60% of the trial population was exposed to biologics. When the percentage of biologics exposure was missing, studies before 1999 were all categorized as having biologic-naive populations and full text screening was used to allocate studies after 1999 to one of the defined subgroups. As the Harvey-Bradshaw Index (HBI) and CDAI are highly correlated and HBI can be transformed to CDAI,[9] we allow for HBI to be an end point in the assessment of clinical remission. It should be noted that factors that explain heterogeneity in placebo response, do not necessarily also impact the relative risk (RR) between drugs when the risk factor is distributed equally across treatment arms. Therefore, we also estimate a full biologic-naive network not accounting for status of previous IM use and compare its results to the IM-naive network.

Exclusions From Network Meta-analysis

Studies were excluded from the NMA if they focused solely on postoperative populations or if they had zero outcome events (as a continuity correction on this data would bias results and the zero-events occurred in subgroups with 10 or fewer patients[10]). No distinction was made between azathioprine and 6-mercaptopurine in the analyses of induction and maintenance of clinical remission, but these were kept separate for the analyses of discontinuation due to adverse events.

End Points

Clinical remission was defined as CDAI<150. If both steroid-free and nonsteroid-free outcomes were available, steroid-free outcomes were preferred. Loss of remission during maintenance treatment was defined as CDAI>150. If CDAI was not available, the HBI and the cutoff points of the original studies for remission were used. Adverse events were analyzed as adverse event-related discontinuations in maintenance trials. In addition, specific adverse events of interest were serious infections, malignancies and major cardiovascular events during maintenance treatment.

Analyses

Scenario analyses of the NMA were performed excluding trials with a high RoB or trials and using only studies with steroid-free end points to test robustness of findings.

The NMA was conducted using frequentist methods and random-effects models, presenting RRs. Inconsistency of direct and indirect evidence was assessed through the I-squared statistic, net-heat plot inspection and the Q-statistic for individual studies. The Cochrane handbook was followed for reporting inconsistency with the I-square statistic ordered in four groups: limited (0%–40%), moderate (30%–60%), substantial (50%–90%) and considerable heterogeneity (75%–100%).[11] Rankings were based on surface under the cumulative ranking (SUCRA) curves.[12] The SUCRA ranking represents the proportion of treatments worse than treatment for which the value is reported. League tables were created to allow inspection of all head-to-head comparisons.

Confidence in results was evaluated with the Confidence in Network Meta-Analysis (CINeMA) framework,[13] specifically developed for NMA's. Indirectness was scored on the percentage of biologics, follow-up duration and the use of composite end points. We used an RR of 0.8 and 1.25 as minimally important difference.

Analyses were conducted in R (version 4.1.3) with RStudio (2023.06.1 + 524) using the 'netmeta' (version 2.1–0) library.[14]

Results

The search strategy identified 3001 unique records of which 340 records were assessed on full text. After full text screening, 123 reports on 124 RCTs were included in the systematic literature review (Figure 1 and Appendix 3). The most frequent reasons for excluding articles on full text screening were discrepancies in outcome (n = 82), study type (n = 60) and publication type (n = 30) (Appendix 4). Appendix 5 describes the characteristics of included studies.

Risk of Bias

Twenty studies (16%) were assessed to have a high overall RoB. Sixteen studies (13%) were judged to be at overall low RoB (Appendix 6). Most important reasons for scoring a high RoB were missing outcome data, selection of the reported result, and deviations from intended interventions. Eighty-six studies (55%) received industry sponsoring.

Included Studies

For the NMA, a further 8 studies were excluded that did not report efficacy results as well as 6 phase II studies. Thirteen further studies were excluded as they focused solely on postoperative populations. Three studies[15–17] were excluded as they included patients with mild CD rather than moderate to severe disease activity. One study with zero outcome events was excluded,[18] and one study was excluded because it could not inform efficacy in the maintenance phase (the inclusion criterion was being stable on azathioprine for 4 years and hence represented a select subgroup).[19]

The remaining 92 trials were allocated to two categories: induction of clinical remission, and maintenance of clinical remission. Each category was stratified to the IM-naive network, biologic-naive and -exposed patients, yielding 6 subgroups. Fifteen studies did not report outcomes for solely biologic-naive or exposed subgroups. Thirteen studies were allocated to a subgroup based on the cutoff point of 60% exposure to biologics. For the maintenance biologic-naive subgroup, azathioprine dosages between 2 and 2.5 mg were pooled to link as many studies to the network as possible. The comparator in Mantzaris et al.

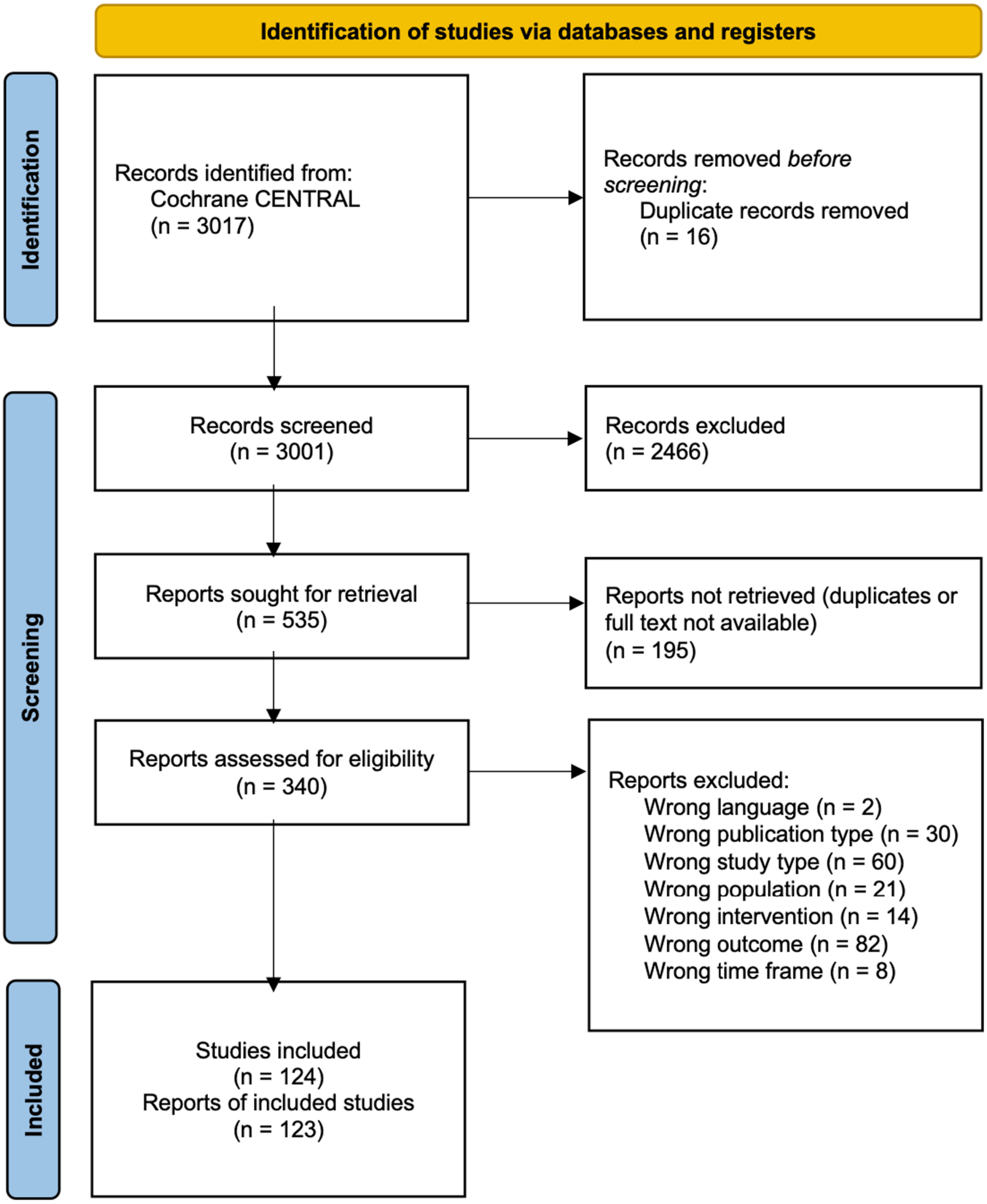

Figure 1. Prisma flow-chart. PRISMA, Preferred Reporting Items for Systematic reviews and Meta-Analyses.

(2009),[20] budesonide 6–9 mg, was included as 6 mg to link to the network.

Of the 92 included studies, 13 were not connected to a network for any of the three outcomes in the naive or exposed subgroups. For the induction outcome in naive patients, both Targan-1997[21] and Campieri-1997[22] were a major source of inconsistency when inspecting direct versus indirect evidence, resulting in their exclusion from the main analyses. Therefore, the NMA was informed by 77 unique trials. Appendix 7 includes details of included studies.

Results of the Network Meta-analysis

The main results of the NMA are described below. The appendices include network plots (Appendix 8), league tables (Appendix 9), SUCRA rankings (Appendix 10), direct versus indirect evidence plots (Appendix 11), scenario analyses (Appendix 12), and the analysis of adverse events (Appendices 13–15), and full CINeMA results (Appendix 16).

Induction of Clinical Remission in Immunomodulator and Biologic-naive Patients

In total 9 out of 33 induction of clinical remission studies reported the proportion of previous IM use. Four studies included fully IM-naive patients and one study included less than <15% previous (see Appendix 7). Matsumoto et al.[23] was not linked to the network. Therefore, 4 studies were included in the network with 6 therapies and 832 patients (Figure 2). Median follow-up was 13 weeks (interquartile

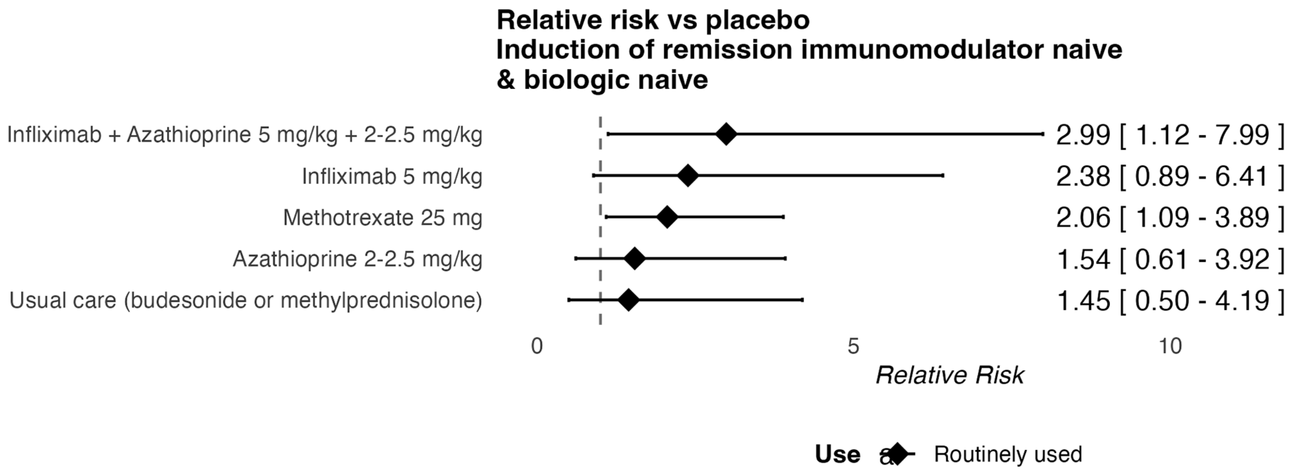

Figure 2. Forest plot of induction treatments in immunomodulator-naïve and biologic-naïve patients.

range (IQR): 10-14). The network displayed no inconsistency. All studies reported steroid-free remission.

Infliximab + azathioprine and methotrexate significantly improved induction of remission compared to placebo with confidence intervals excluding 1. The comparison of infliximab monotherapy vs placebo was indirect, widening confidence intervals. The highest SUCRA ranking was for infliximab + azathioprine (0.95), followed by infliximab mono (0.74), methotrexate 25 mg (0.6), and azathioprine (0.3).

Induction of Clinical Remission in Biologic-naive Patients

For the biologic-naive subgroup, 33 studies were included in the network, with 37 different therapies and 7504 patients, suggesting a sparse network (a limited number of studies for each treatment evaluated) (Figure 3). Median follow-up was 10 weeks (IQR: 8-12). The network displayed limited inconsistency (I^2: 9.9%).

The following therapies significantly improved induction of remission compared to placebo in biologic-naive patients (RR > 1, with confidence intervals (CIs) excluding 1): adalimumab, BI695501 (adalimumab biosimilar), ustekinumab, adalimumab + azathioprine, infliximab + azathioprine, infliximab, CT-P13 (infliximab biosimilar), methotrexate, prednisolone, budesonide, natalizumab, certolizumab pegol, and upadacitinib. Adalimumab in a high induction regime (160 mg at weeks 0, 1, 2, and 3 followed by 40 mg from week 4 onwards) had the highest SUCRA score (0.94). The highest-ranking IM was methotrexate 25 mg with a SUCRA score of 0.64. The highest-ranking biologic other than anti-TNF was ustekinumab 6 mg/kg (0.93) (Appendix 10). The results show very limited difference with the IM naive network, granting credibility to the transitivity assumption for the full biologic-naive NMA. When compared against anti-TNF combination therapy, the CINeMA derived confidence rating was 'moderate' vs azathioprine 2–2.5mg, 5-ASA 1500mg, budesonide 1.5mg and 3mg, CDP571 10mg/kg certolizumab pegol 400mg, mongersen 160mg, natalizumab 3mg/kg, placebo, and upadacitinib 45mg. Other comparisons had 'low' to 'very low' confidence.

Sensitivity Analyses

Excluding trials with a high RoB did not substantially influence the rank order of the RRs of therapies whose trials were not excluded.

Conducting the analysis in the subgroup of trials with only steroid-free end points decreased the certainty of superiority of treatments versus placebo, increased the ranking of budesonide, but did not affect the overall ordering of RRs (Appendix 12).

Induction of Clinical Remission in Biologic-exposed Patients

For the biologic-exposed subgroup, 14 studies were included in the network, with 20 therapies and 5070 patients, suggesting a sparse network (Figure 4). Median follow-up was 10 weeks (IQR: 8-12). The network displayed no heterogeneity as it consisted mainly of direct evidence.

The following therapies significantly improved induction of remission compared to placebo in biologic-exposed patients (RR > 1 with confidence intervals excluding 1): upadacitinib, ustekinumab and risankizumab. Upadacitinib 45 mg had the highest SUCRA score (0.95). The highest-ranking anti-TNF was adalimumab 160/80/40 mg with a SUCRA score of 0.66. The highest-ranking biologic other than anti-TNF was ustekinumab 6 mg/kg (0.86) (Appendix 10). When compared against ustekinumab, the CINeMA derived confidence rating was 'high' vs placebo, 'moderate' vs mongersen 160mg and tofacitinib 5mg. Other comparisons had 'low' to 'very low' confidence.

Sensitivity Analysis

No sensitivity analysis was performed for induction of clinical remission in the biologic-exposed subgroup because

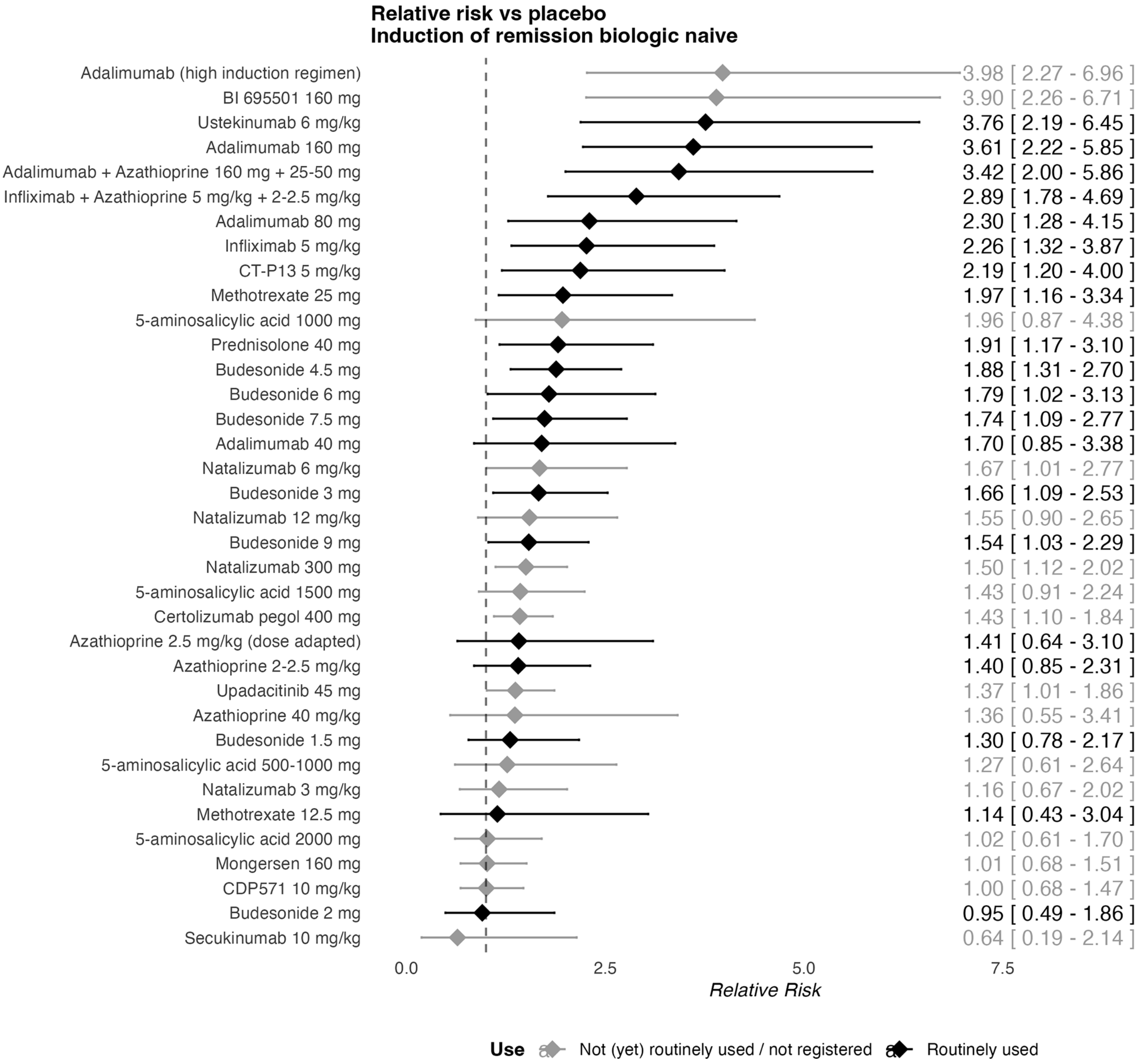

Figure 3. Forest plot of induction treatments in biologic-naive patients.

there were no trials at a high overall RoB. The ranking of RRs and the significant difference versus placebo of three trials with steroid-free outcomes was similar to the main analysis, but with a less pronounced numerical difference between adalimumab and vedolizumab (Appendix 12).

Maintenance of Clinical Remission in Immunomodulator and Biologic-naive Patients

In total 5 out of 29 maintenance of clinical remission studies reported the proportion of previous IM use. In 2 studies, no or a small proportion (<3%) of patients was previously treated with IM.[24,25] Direct evidence of the RR for losing clinical remission in these studies was: 0.57 [95% CI = 0.35–0.04] for methotrexate 15mg vs placebo and 0.67 [95% CI = 0.46–0.97] for infliximab vs usual care (70% IM use at week 52). It was not possible to form a network of the two studies with IM and biologic naive patients, therefore no NMA was performed.

Maintenance of Clinical Remission in Biologic-naive Subgroup

For the biologic-naive subgroup 29 studies were included in the network, with 25 different therapies and 4630 patients, suggesting a sparse network (Figure 5). Median follow-up was 52 weeks (IQR: 47.5-52). The network displayed limited inconsistency (I^2: 27.5%).

The following therapies significantly improved maintenance of remission compared to placebo in biologic-naive patients (RRs < 1 with confidence intervals excluding 1): infliximab with azathioprine, azathioprine, upadacitinib,

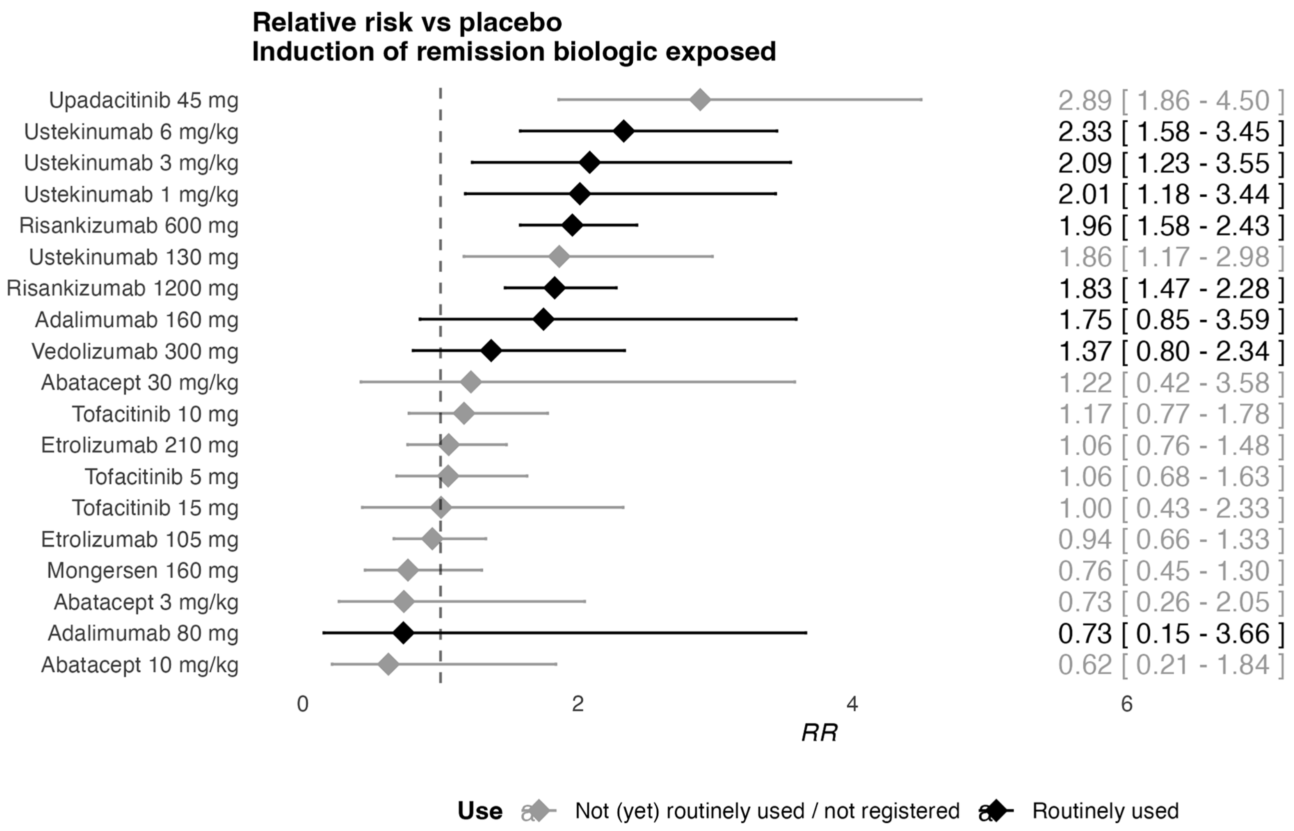

Figure 4. Forest plot of induction treatments in biologic-exposed patients.

adalimumab and infliximab. Infliximab 5 mg/kg combined with azathioprine 2–2.5 mg/kg had the highest SUCRA score (0.96). The highest-ranking IM was azathioprine 2–2.5 mg/kg monotherapy with a SUCRA score of 0.83. The highest-ranking biologic that was not anti-TNF was ustekinumab 90 mg (0.84) (Appendix 10). When compared against anti-TNF combination therapy, the CINeMA derived confidence rating was 'moderate' vs budesonide 1mg and 9mg and vs vedolizmab 108mg. Other comparisons had 'low' or 'very low' confidence.

Sensitivity Analysis

For maintenance of clinical remission in the biologic-naive subgroup, excluding trials with a high overall RoB increased the ranking adalimumab 40 mg but with greater imprecision (RR 0.34 [95% CI 0.11-1.04]).

Maintenance of Clinical Remission in Biologic-exposed Subgroup

For the biologic-exposed subgroup seven studies were included in the network, with 12 treatments and 1909 patients, suggesting a sparse network (Figure 6). Median follow-up was 46 weeks (IQR: 41-52). The network displayed no heterogeneity.

The following therapies significantly improved maintenance of remission compared to placebo in biologic-exposed patients (RR $<$ 1 with confidence intervals excluding 1): upadacitinib, ustekinumab, vedolizumab, and risankizumab. Upadacitinib 30mg had the highest SUCRA score (0.93). The highest-ranking biologic that was not anti-TNF was ustekinumab 90 mg with a SUCRA score of 0.76 (Appendix 10). When compared against ustekinumab 90mg, the CINeMA derived confidence rating was downgraded to 'very low' for all comparisons due to indirectness, imprecision and heterogeneity.

Sensitivity Analysis

No sensitivity analysis was performed for maintenance of clinical remission in biologic-exposed subgroup because there were no trials with a high overall RoB.

Responder-Rerandomized vs Treat Through Maintenance Studies

In the biologic-naive network, 7 studies were specified as responder rerandomization studies, of which only one included a biologic (ie adalimumab).[26] Given that the efficacy of adalimumab in responders may carry over to those rerandomized to the placebo arm, it could be that the NMA underestimates the efficacy of adalimumab. In the biologic-exposed network, all included studies were responder rerandomization trials. The lowest placebo remission rate of 0.11 was observed for the only nonbiologic trial included

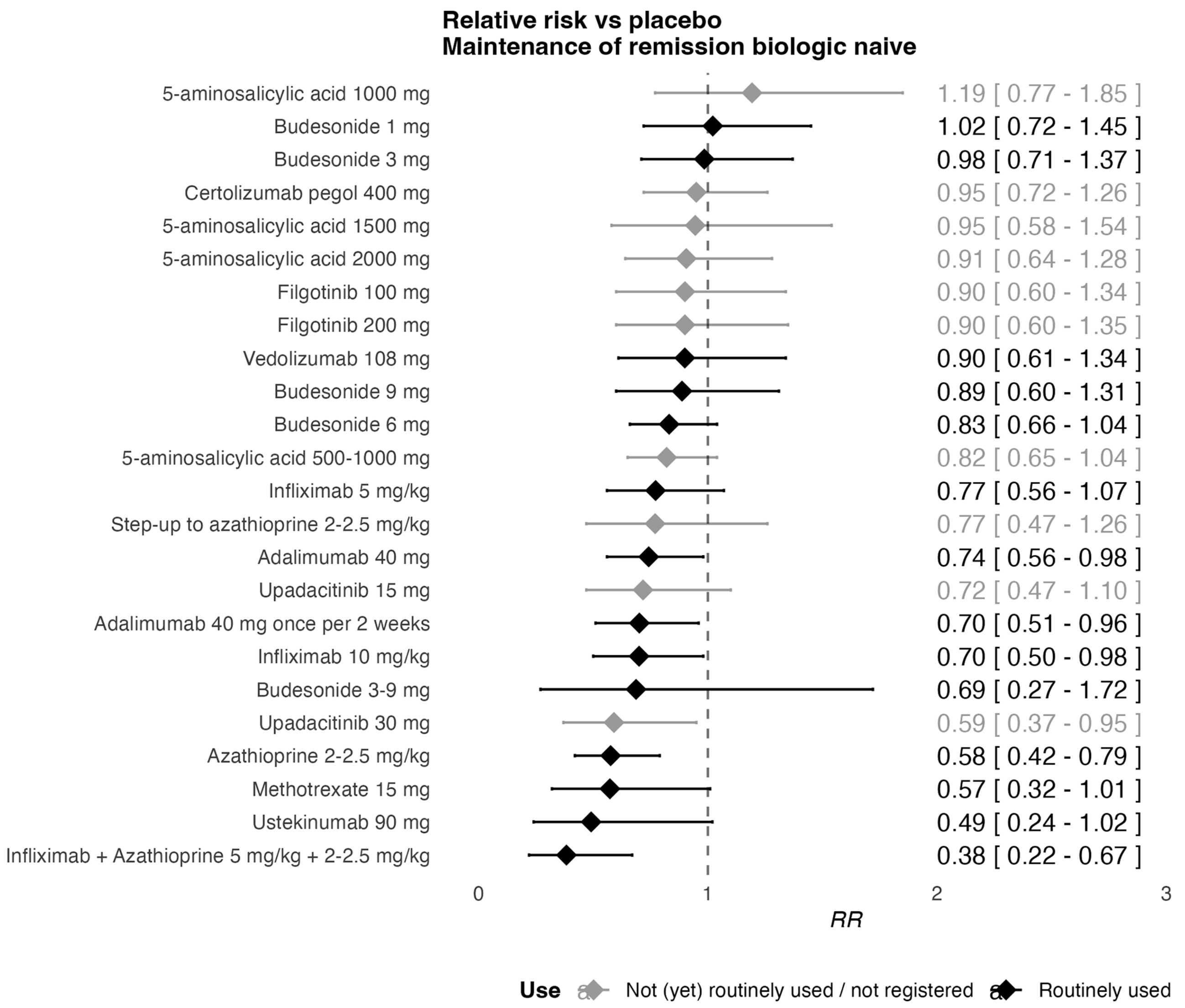

Figure 5. Forest plot of maintenance treatments in biologic-naive patients. Results represent ability to maintain remission relative to placebo, for those who achieved remission on the treatment in an induction setting.

in the NMA (ie upadactinib[27]). A meta-analysis of placebo arms of other studies, without the placebo arm of the upadacitinib trial, yielded a remission rate of 0.31. To account for this imbalance, a scenario analysis in which the event-rate was assumed equal to that of the meta-analysis for all placebo arms reduced the efficacy of upadacitinib vs placebo to a RR of 0.8 (95% CI: 0.65-0.97].

Discussion

This study reports on the efficacy of pharmacological treatments for moderate to severe CD based on a NMA with evidence from 77 RCTs since 1990. We show that when IM-naive trials are pooled in a network with trials of which the previous IM-exposure status is unknown or known to be exposed but to an uncertain degree, there is a negligible decline in the RR when compared to the RRs of the IM-naive network. Thus, pooling studies in a large 'biologic-naive' network is feasible and does not demonstrate violations of the transitivity assumption. We demonstrate that all therapies that are in use for CD have overlapping confidence intervals, suggesting that no claims to superiority can be made based on current data synthesis. Nevertheless, we show that anti-TNF (combination) therapy has higher SUCRA rankings in biologic-naive patients than recently approved therapies, suggesting it is more likely to improve outcomes, but differences are small and uncertain. Methotrexate is the highest-ranking IM (10th) in the induction of clinical remission and azathioprine (3rd) in maintenance of clinical remission in biologic-naive patients, and its SUCRA score is consistent between the IM-naive and biologic-naive network. Upadacitinib is ranking highest in biologic-exposed patients for both induction and maintenance of clinical remission.

This study is the first NMA to report on the comparative efficacy of IMs relative to advanced therapies in moderate to

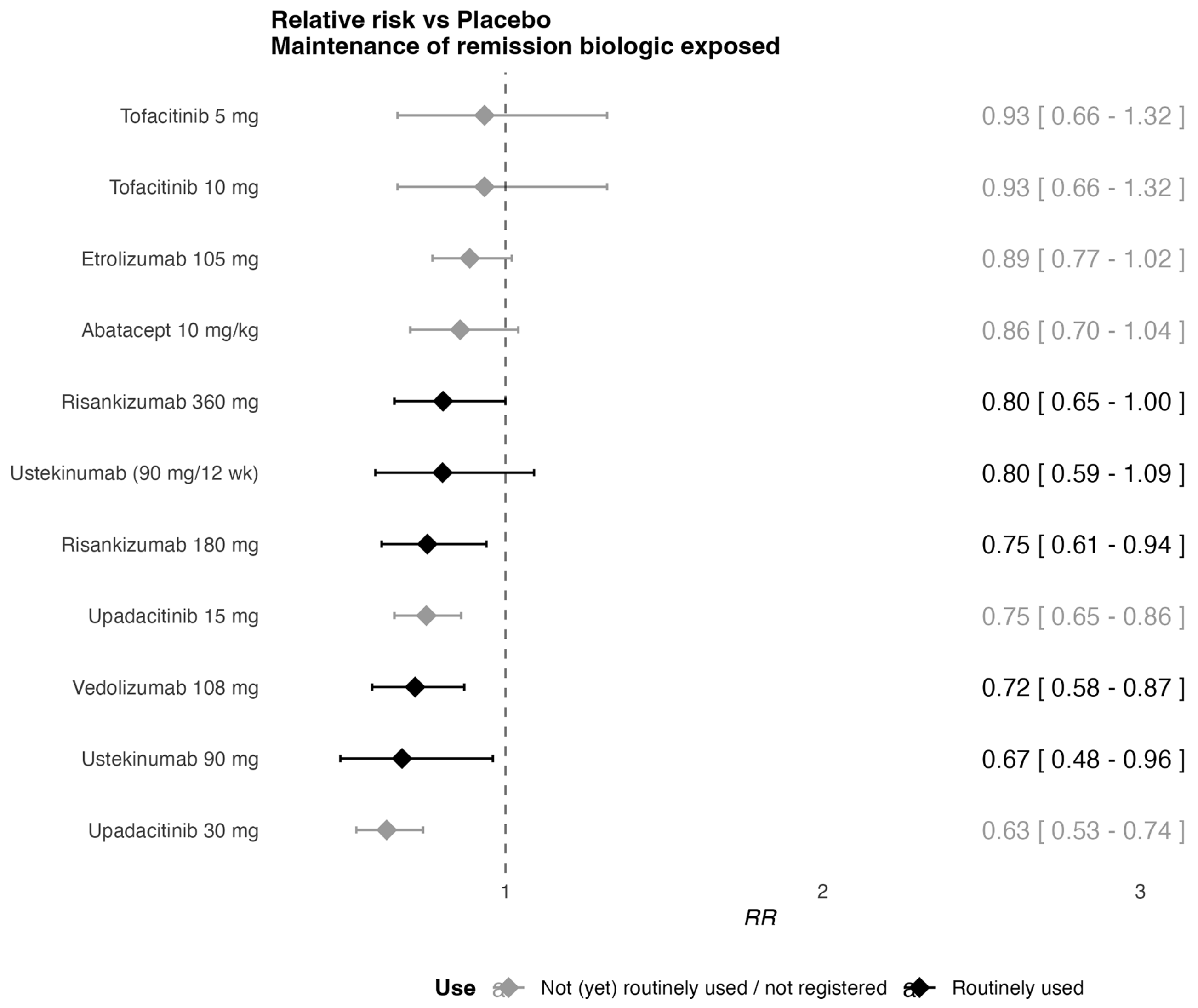

Figure 6. Forest plot of maintenance treatments in biologic-exposed patients. Results represent ability to maintain remission relative to placebo, for those who achieved remission on the treatment in an induction setting.

severe CD. Findings show that thiopurines and methotrexate are effective in achieving and maintaining (steroid-free) clinical remission. Long-term efficacy of IMs is confirmed by observational studies.[28,29] Tolerability and long-term safety are known issues with IMs causing discontinuation of methotrexate in the induction phase and a moderately increased risk of nonmelanoma skin cancer,[30] and a moderately increased risk of cancer overall.[31] Therapeutic drug monitoring could aid optimal use of this drug class. While there are also known long-term safety risk for sustained anti-TNF therapy, which moderately increases risk of melanoma in IBD patients,[32] these need to be balanced against the unknown long-term risk of newer drug classes. Ongoing prospective cohort studies will shed more light on the risk-benefit of all currently available therapeutic options for CD.[33] It is worth noting that the difference in costs of recently approved therapies compared to IMs or anti-TNF biosimilars is proportionally larger than the difference in clinical efficacy.

In accordance with previous studies, we also identify infliximab + azathioprine as the best performing compound. We found a smaller (but more certain) effect estimate for infliximab versus placebo, compared to that reported in Singh et al.,[3] or infliximab versus azathioprine, compared to that reported in Hazelwood et al.[34]). The difference with the estimate of infliximab versus placebo from Singh et al. may be explained by the exclusion of Targan-1997 in our NMA. Targan-1997 is a relatively small but important study (n = 27, 28, 28, 24 in the four arms of the trial) comparing infliximab to placebo.[21] We excluded Targan-1997 because it increased inconsistency from an I^2 of 9.9%–19.3%. This was due to a higher efficacy of infliximab versus placebo in Targan-1997 than expected from indirect evidence in our NMA. This may be explained by the placebo event rates reported in Targan-1997: 17% for response and 4% for remission, considerably lower than those identified in a systematic review of placebo

event rates (response 28% [95% CI: 24%–32%] and remission 18% [95% CI: 16%–21%]).[8] In contrast to our NMA, Singh et al. included Targan-1997, which increased their efficacy estimate for infliximab. This was demonstrated by the large difference between the effect estimate for infliximab from Targan-1997 (OR = 18.4 CI 2.16-156.58) and another RCT evaluating infliximab[16] (OR = 5.03 (CI 2.25–11.22).[2] These findings suggest that while Targan-1997 may have good internal validity, the transitivity assumption of no relevant differences between studies besides the intervention may not hold.

A difference with the NMA of Hazelwood et al. is that their study reports no overlapping confidence intervals for the RRs of infliximab versus azathioprine for the induction of clinical remission. This may be caused by several differences: Hazelwood et al. included Targan-1997, studies that reported laboratory end points other than CDAI reduction (2 studies), studies that were not in moderate to severe Crohn patients (3 studies), and studies that did not differentiate between remission and response (1 study).

Following the cutoff date for the search, other evidence has emerged that could have impact on the estimates of this NMA: SEQUENCE, VIVID-1, and GALAXI-2 and 3. The SEQUENCE trial was a noninferiority study comparing ustekinumab and risankizumab standard dosing in biologic-exposed patients, demonstrating noninferiority, but numerically stronger results for risankizumab.[35] Including this comparison would increase the RR of induction of clinical remission (maintenance of remission) in exposed patients to 2.21 [95% CI 1.76-2.78] (0.67 [95% CI 0.39-1.14 for maintenance) risankizumab vs 1.71 [95% CI 1.29; 2.25] (0.84 [95% CI 0.48-1.47] for maintenance) for ustekinumab, thus, suggesting improved relative efficacy of risankizumab but with overlapping confidence intervals. VIVID-1 demonstrated superiority of mirikizumab (EMA approval end 2024) over placebo.[36] Ustekinumab was included in VIVID-1, but results were sparsely reported. The GALAXI trials compare guselkumab (EMA approval may 2025) with ustekinumab and placebo in patients who had inadequate response or tolerance of IMs or biologic therapies.[37] The trial demonstrated superiority of guselkumab on several end points, but not clinical remission, the end point utilized in this NMA. For the GALAXI trial only conference abstracts are available hindering uptake in this NMA.

Strengths and Limitations

This systematic literature review with NMA provides the most recent update of all available phase III RCT evidence on treatment for CD. Unique to our study was the comparison of relative efficacy between all treatments available for moderate-to-severe CD, including older IMs. The inclusion criteria were set to include all relevant RCTs irrespective of Food and Drug Administration/EMA approval of the compound. This allowed us to draw insights from many more studies which strengthened the network and consequently the estimates of our NMA. In this study, optimal transitivity was sought through adopting strict inclusion criteria, using similar end point definitions and study follow-up time horizons, as well as by creating different subgroups for IM and biologic exposure status. We repeated analyses for different RoB outcomes and steroid use with similar results as the base case analysis. Our results suggest that previous exposure to IMs does not affect the transitivity assumption: the results of the IM naive network are similar to the results of the biologic-naive network (including both IM naive and exposed patient groups). Furthermore, the Study of Biologic and Immunomodulater Naive Patients in Crohn's Disease (SONIC) trial (which included patients naive to IMs and biologics) week 10 remission outcomes are highly similar to the induction of clinical remission results from our NMA, even though the NMA includes a majority of evidence from studies in which included patients had prior exposure to IMs.

Our study also has several limitations. First, we reported estimates of clinical remission as measured by CDAI and HBI that have demonstrated a poor correlation with mucosal inflammation.[38] However, it was not possible to report on endoscopic remission or other biomarkers because these outcomes are generally not available from trials in the prebiologic era.

Second, as newer therapies are most often compared against placebo in RCTs, in this NMA the majority of comparisons against an older active comparator are based on indirect evidence in sparse networks where the number of treatments was often higher or similar to the number of studies. The variance of indirect evidence is the sum of the variance of the direct evidence and hence is likely to produce wider confidence intervals. The sparsity of the networks and the wide confidence intervals suggest that any result must be interpreted with caution, as holds true for all NMA's in CD.

Third, not all trials report biologic-naive or biologic-exposed subgroups. We defined a cutoff point to define a mixed-population as being 'exposed' or 'naive'. While the authors agreed that a cutoff point of more than half the sample (60%) was appropriate, it remains an arbitrary selection. However, this only affected 13 of the included studies. In the IM-naive network, none of the trials was affected by this allocation. In the biologic-naive subgroup for induction of remission, 7 trials were allocated based on the cutoff and had a mean exposure rate of 21.4%. For biologic-exposed induction two trials were allocated and had a mean exposure rate of 68.2%. In the biologic-naive maintenance subgroup four trials were allocated and had a mean exposure rate of 48% (these trials compared filgotinib, certolizumab, and adalimumab with placebo), and one study in the biologic-exposed maintenance subgroup with an exposure rate of 60%.

Fourth, we excluded studies without events in one of the arms. While common for frequentist NMAs, such an exclusion is not required for Bayesian approaches to NMAs.

Fifth, the NMA for the end point discontinuation due to adverse events yielded wide confidence intervals due to a low

frequency of events and hence was not as informative as we had hoped for the analysis of adverse events. Post marketing surveillance studies reveal rare safety events years after registration of new drugs.[39] If, as demonstrated in this NMA, there are no large differences in efficacy, the limited information on safety may cause clinicians to favor prescription of drugs for which extensive real world safety data is available.

A last limitation is related to the responder-rerandomization trial design for maintenance studies. Notably in the biologic-exposed subgroup, all but one of the included therapies is biologics that are expected to maintain efficacy after withdrawal, except for upadacitinib. Upadacitinib is a Janus Kinase 1 inhibitor with a half-life of 9–14 hours[40] that may not have sustained efficacy after withdrawal. Hence, the upadacitinib trial is the only trial for which the responder-rerandomization does not impact placebo rates, giving it a favorable result relative to the comparators in that network.

Conclusion

Our study demonstrates that recently approved therapies do not rank highest for the induction or maintenance of clinical remission, except in biologic-exposed patients, and even then, numerical differences are small and confidence intervals overlap. The relatively high efficacy and low costs, of IMs and anti-TNF (combination) regimens suggests that recently approved therapies best serve the population who have loss of response or adverse events on at least one biologic. The highly uncertain discontinuation due to adverse rates was numerically unfavorable for methotrexate, azathioprine and upadacitinib, which requires vigilance. Experience of health care professionals with a drug class and patient preferences should be considered when selecting a treatment for an individual patient. Health economic analyses ought to be conducted to investigate the balance between costs and effects of different treatment sequences in CD.

Supplementary Materials

Supplementary data associated with this article can be found, in the online version, at https://doi.org/10.1016/j.gastha.2025.100764.

References

1. Burisch J, Kaplan GG, Duricova D, et al. The cost of inflammatory bowel disease in high-income settings: a lancet gastroenterology & hepatology commission. Lancet Gastroenterol Hepatol 2023;8(5):458–492. http://doi.org/10.1016/S2468-1253(23)00003-1.
2. Singh S, Murad MH, Fumery M, et al. Comparative efficacy and safety of biologic therapies for moderate-to-severe Crohn's disease: a systematic review and network meta-analysis. Lancet Gastroenterol Hepatol 2021;6(12):1002–1014. http://doi.org/10.1016/S2468-1253(21)00312-5.
3. Barberio B, Gracie DJ, Black CJ, et al. Efficacy of biological therapies and small molecules in induction and maintenance of remission in luminal Crohn's disease: systematic review and network meta-analysis. Gut 2023;72(2):264–274. http://doi.org/10.1136/gutjnl-2022-328052.
4. Shehab M, Alrashed F, Heron V, et al. Comparative efficacy of biologic therapies for inducing response and remission in fistulizing Crohn's disease: systematic review and network meta-analysis of randomized controlled trials. Inflamm Bowel Dis 2023;29(3):367–375. http://doi.org/10.1093/ibd/izac103.
5. Hutton B, Salanti G, Caldwell DM, et al. The PRISMA extension statement for reporting of systematic reviews incorporating network meta-analyses of health care interventions: checklist and explanations. Ann Intern Med 2015;162(11):777–784. http://doi.org/10.7326/M14-2385.
6. Sterne JAC, Savović J, Page MJ, et al. RoB 2: a revised tool for assessing risk of bias in randomised trials. BMJ 2019;366:l4898. http://doi.org/10.1136/bmj.l4898.
7. Su C, Lichtenstein GR, Krok K, et al. A meta-analysis of the placebo rates of remission and response in clinical trials of active crohn's disease. Gastroenterology 2004;126(5):1257–1269. http://doi.org/10.1053/j.gastro.2004.01.024.
8. Jairath V, Zou G, Parker CE, et al. Systematic review with meta-analysis: placebo rates in induction and maintenance trials of Crohn's disease. Aliment Pharmacol Ther 2017;45(8):1021–1042. http://doi.org/10.1111/apt.13973.
9. Vermeire S, Schreiber S, Sandborn WJ, et al. Correlation between the Crohn's disease activity and harvey–bradshaw indices in assessing Crohn's disease severity. Clin Gastroenterol Hepatol 2010;8(4):357–363. http://doi.org/10.1016/j.cgh.2010.01.001.
10. Dias S, Ades AE, Welton NJ, et al. Network meta-analysis for decision-making. John Wiley & Sons, 2018.
11. Deeks JJ, Higgins JP, Altman DG, and on behalf of the C. S. M. Group. Analysing data and undertaking meta-analyses. In: Cochrane handbook for systematic reviews of interventions. John Wiley & Sons, Ltd, 2019:241–284. http://doi.org/10.1002/9781119536604.ch10.
12. Rücker G, Schwarzer G. Ranking treatments in frequentist network meta-analysis works without resampling methods. BMC Med Res Methodol 2015;15(1):58. http://doi.org/10.1186/s12874-015-0060-8.
13. Nikolakopoulou A, Higgins JPT, Papakonstantinou T, et al. CINeMA: an approach for assessing confidence in the results of a network meta-analysis. PLoS Med 2020;17(4):e1003082. http://doi.org/10.1371/journal.pmed.1003082.
14. Balduzzi S, Rücker G, Nikolakopoulou A, et al. Netmeta: an R package for network meta-analysis using frequentist methods. J Stat Softw 2023;106:1–40. http://doi.org/10.18637/jss.v106.i02.
15. Maté-Jiménez J, Hermida C, Cantero-Perona J, et al. 6-mercaptopurine or methotrexate added to prednisone induces and maintains remission in steroid-dependent inflammatory bowel disease. Eur J Gastroenterol Hepatol 2000;12(11):1227–1233. http://doi.org/10.1097/00042737-200012110-00010.
16. LÉmann M, Mary JY, Duclos B, et al. Infliximab plus azathioprine for steroid-dependent crohn's disease

patients: a randomized placebo-controlled trial. Gastroenterology 2006;130(4):1054–1061. http://doi.org/10.1053/j.gastro.2006.02.014.

17. Prantera C, Pallone F, Brunetti G, et al. Oral 5-aminosalicylic acid (Asacol) in the maintenance treatment of Crohn's disease. Gastroenterology 1992;103(2):363–368. http://doi.org/10.1016/0016-5085(92)90822-G.
18. Stack WA, Mann SD, Roy AJ, et al. Randomised controlled trial of CDP571 antibody to tumour necrosis factor-α in Crohn's disease. The Lancet 1997;349(9051):521–524. http://doi.org/10.1016/S0140-6736(97)80083-9.
19. Wenzl HH, Primas C, Novacek G, et al. Withdrawal of long-term maintenance treatment with azathioprine tends to increase relapse risk in patients with Crohn's disease. Dig Dis Sci 2015;60(5):1414–1423. http://doi.org/10.1007/s10620-014-3419-5.
20. Mantzaris GJ, Christidou A, Sfakianakis M, et al. Azathioprine is superior to budesonide in achieving and maintaining mucosal healing and histologic remission in steroid-dependent crohn's disease. Inflamm Bowel Dis 2009;15(3):375–382. http://doi.org/10.1002/ibd.20777.
21. Targan SR, Hanauer SB, van Deventer SJ, et al. A short-term study of chimeric monoclonal antibody cA2 to tumor necrosis factor α for Crohn's disease. New Engl J Med 1997;337(15):1029–1036. http://doi.org/10.1056/NEJM199710093371502.
22. Campieri M, Ferguson A, Doe W, et al. Oral budesonide is as effective as oral prednisolone in active Crohn's disease. Gut 1997;41(2):209–214. http://doi.org/10.1136/gut.41.2.209.
23. Matsumoto T, Motoya S, Watanabe K, et al. Adalimumab monotherapy and a combination with azathioprine for Crohn's disease: a prospective, randomized trial. J Crohn's Colitis 2016;10(11):1259–1266. http://doi.org/10.1093/ecco-jcc/jjw152.
24. D'Haens G, Baert F, van Assche G, et al. Early combined immunosuppression or conventional management in patients with newly diagnosed Crohn's disease: an open randomised trial. The Lancet 2008;371(9613):660–667. http://doi.org/10.1016/S0140-6736(08)60304-9.
25. Feagan BG, Rochon J, Fedorak RN, et al. Methotrexate for the treatment of Crohn's disease. New Engl J Med 1995;332(5):292–297. http://doi.org/10.1056/NEJM199502023320503.
26. Sandborn WJ, Hanauer SB, Rutgeerts P, et al. Adalimumab for maintenance treatment of Crohn's disease: results of the CLASSIC II trial. Gut 2007;56(9):1232–1239. http://doi.org/10.1136/gut.2006.106781.
27. Loftus EV, Panés J, Lacerda AP, et al. Upadacitinib induction and maintenance therapy for Crohn's disease. New Engl J Med 2023;388(21):1966–1980. http://doi.org/10.1056/NEJMoa2212728.
28. Rezazadeh Ardabili A, Jeuring S, Mujagic Z, et al. Classic drugs in the time of new drugs: real-world, long-term outcomes of thiopurine monotherapy in 1016 patients with inflammatory bowel disease. Aliment Pharmacol Ther 2022;56(6):1030–1043. http://doi.org/10.1111/apt.17128.
29. Mesonero F, Castro-Poceiro J, Benítez JM, et al. Effectiveness and safety of methotrexate monotherapy in patients with Crohn's disease refractory to anti-TNF-α: results from the ENEIDA registry. Aliment Pharmacol Ther 2021;53(9):1021–1029. http://doi.org/10.1111/apt.16315.
30. Peyrin–Biroulet L, Khosrotehrani K, Carrat F, et al. Increased risk for nonmelanoma skin cancers in patients who receive thiopurines for inflammatory bowel disease. Gastroenterology 2011;141(5):1621–1628.e5. http://doi.org/10.1053/j.gastro.2011.06.050.
31. van den Heuvel TRA, Wintjens DSJ, Jeuring SFG, et al. Inflammatory bowel disease, cancer and medication: cancer risk in the Dutch population-based IBDSL cohort. Int J Cancer 2016;139(6):1270–1280. http://doi.org/10.1002/ijc.30183.
32. Long MD, Martin CF, Pipkin CA, et al. Risk of melanoma and nonmelanoma skin cancer among patients with inflammatory bowel disease. Gastroenterology 2012;143(2):390–399.e1. http://doi.org/10.1053/j.gastro.2012.05.004.
33. Peyrin-Biroulet L, Rahier JF, Kirchgesner J, et al. I-CARE, a European prospective cohort study assessing safety and effectiveness of biologics in inflammatory bowel disease. Clin Gastroenterol Hepatol 2023;21(3):771–788.e10. http://doi.org/10.1016/j.cgh.2022.09.018.
34. Hazlewood GS, Rezaie A, Borman M, et al. Comparative effectiveness of immunosuppressants and biologics for inducing and maintaining remission in crohn's disease: a network meta-analysis. Gastroenterology 2015;148(2):344–354.e5. http://doi.org/10.1053/j.gastro.2014.10.011.
35. Peyrin-Biroulet L, Chapman JC, Colombel JF, et al. Risankizumab versus ustekinumab for moderate-to-severe crohn's disease. New Engl J Med 2024;391(3):213–223. http://doi.org/10.1056/NEJMoa2314585.
36. Ferrante M, D'Haens G, Jairath V, et al. Efficacy and safety of mirikizumab in patients with moderately-to-severely active Crohn's disease: a phase 3, multicentre, randomised, double-blind, placebo-controlled and active-controlled, treat-through study. The Lancet 2024;404(10470):2423–2436. http://doi.org/10.1016/S0140-6736(24)01762-8.
37. Janssen research & development, LLC, "A Phase 2/3, Randomized, Double-blind, Placebo- and Active-controlled, Parallel-group, Multicenter Protocol to Evaluate the Efficacy and Safety of Guselkumab in Participants With Moderately to Severely Active Crohn's Disease," clinicaltrials.gov, clinical trial registration NCT03466411. [Online]. Available: https://clinicaltrials.gov/study/NCT03466411. Accessed June 2, 2025.
38. Ricanek P, Brackmann S, Perminow G, et al. Evaluation of disease activity in IBD at the time of diagnosis by the use of clinical, biochemical, and fecal markers. Scand J Gastroenterol 2011;46(9):1081–1091. http://doi.org/10.3109/00365521.2011.584897.
39. Downing NS, Shah ND, Aminawung JA, et al. Post-market safety events among novel therapeutics approved by the US food and drug administration between 2001 and 2010. JAMA 2017;317(18):1854–1863. http://doi.org/10.1001/jama.2017.5150.
40. European Medicines Agency. Rinvoq: EPAR - product information. [Online]. Available: https://www.ema.europa.eu/en/documents/product-information/rinvoq-epar-product-information_en.pdf. Accessed March 4, 2025.

Received March 11, 2025. Accepted August 6, 2025.

Correspondence:
Address correspondence to: Matthijs Versteegh, PhD, National Health Care Institute, Willem Dudokhof 1, Diemen 1112 ZA, The Netherlands. e-mail: matthijs@huygensandversteegh.com or mversteegh@zinl.nl.

Authors' Contributions:
Matthijs Versteegh: conceptualization, methodology, formal analysis, software, writing – Original Draft (lead), supervision. Demy Idema: conceptualization, investigation, writing – Original Draft. Simone Huygens: conceptualization, methodology, formal analysis, software, writing – Original Draft. Kevin Jenniskens: conceptualization, methodology, writing – Original Draft. Marieke Pierik: Investigation, Writing – Review & Editing, Tessa Römkens, Investigation, Writing – Review & Editing, Fiona van Schaik, Investigation, Writing – Review & Editing, Peter Wahab, Investigation, Writing – Review & Editing, Linde Huis in 't Veld, Investigation, Writing – Review & Editing, Mike Kusters, Investigation, Writing – Review & Editing, Kim van der Braak, Investigation, Writing – Review & Editing, Lotty Hooft, conceptualization, methodology, Investigation, Writing – Review & Editing, supervision, Johanna Damen: conceptualization, methodology, writing – Original Draft, supervision.

Conflicts of Interest:
The authors disclose the following: All reported conflicts of interest reflect payments to the employing institutions not to individuals. Matthijs Versteegh and Simone Huygens are part time employees of the National Health Care Institute and report no conflicts of interest. Demy Idema, Kevin Jenniskens, Linde Huis in 't Veld, Mike Kusters, Kim van der Braak, Lotty Hooft, and Johanna Damen are employed at the Dutch Cochrane Center of the Julius Center for Health Sciences and primary care which received funding from the Dutch Health Care Institute for the execution of the systematic literature review. Tessa Römkens reports speaker fees from Janssen and advisory board fees from BMS and AbbVie. Fiona van Schaik reports an unrestricted research grant from Takeda, consulting fees from Galapagos, and honoraria for lectures, presentations or education events from Galapagos, Janssen-Cilag and Eli-Lily. Marieke Pierik reports unrestricted research grants from TKI FRESH, Maag Darm Leverstichting, and Takeda, and consulting fees from Takeda, Abbvie, Ferring, and MSD, and honoraria for lectures by Johnson and Johnson.

Funding:
This research was funded by the National Health Care Institute (Zorginstituut Nederland).

Ethical Statement:
The study did not require the approval of an institutional review board.

Data Transparency Statement:
All data including NMA code is available upon reasonable request from the authors for noncommercial ends.

Reporting Guidelines:
This research followed the PRISMA reporting guideline for systematic reviews and the CInEMA approach for evaluating Network Meta-analysis results.

Gastro Hep Advances, Volume 5

Supplemental information

Comparative Efficacy of all Available Pharmaceutical Therapies for Moderate to Severe Crohn's Disease: A Systematic Review and Network Meta-Analysis

Matthijs Versteegh, Demy L. Idema, Simone Huygens, Kevin Jenniskens, Marieke Pierik, Tessa Römkens, Fiona van Schaik, Peter Wahab, Linde F. Huis in't Veld, Mike Kusters, Kim van der Braak, Lotty Hooft, and Johanna A.A. Damen

Appendix

Table of Contents

1) Search strategy 2

2) PICOTS 3

3) Flow chart study selection 4

4) Overview of excluded studies 5

5) Characteristics of studies included in systematic review 26

6) Risk of bias assessments 74

7) Studies included in network meta-analyses 75

Induction remission 75
Maintenance 81

8) Network plots 85

Induction remission 85
Maintenance 87

9) League tables 88

Induction remission 88
Maintenance 92

10) SUCRA rankings 95

Induction remission 95
Maintenance 97

11) Direct vs indirect evidence 98

Induction remission 98
Maintenance 100

12) Scenario analyses 102

Results of sensitivity analysis on steroid-free outcomes 102
Results of sensitivity analysis excluding studies with a high risk of bias 104

13) AE related discontinuation 106

Biologically naïve 106
Biologically exposed 107

14) Adverse events – median rates 108

Biologically naïve 108
Biologically exposed 109

15) Adverse events – rates per study 110

Biologically naïve 110
Biologically exposed 113

16) CINeMA approach and results 115

Approach 115
Results Induction naive 116
Results Induction exposed 119
Results Maintenance naive 121
Results Maintenance exposed 123

1) Search strategy

CENTRAL
Search date: 18 October 2023

No.	Query	Results
#1	MESH DESCRIPTOR Crohn Disease EXPLODE ALL TREES	2481
#2	irritable colon:EH	1833
#3	MESH DESCRIPTOR Colitis, Ulcerative EXPLODE ALL TREES	1989
#4	MESH DESCRIPTOR Inflammatory Bowel Diseases EXPLODE ALL TREES	4764
#5	MESH DESCRIPTOR Irritable Bowel Syndrome EXPLODE ALL TREES	1621
#6	Crohn*:TI,AB,KY,EH	5594
#7	((ulcerative adj3 colitis) OR "inflammatory bowel disease" OR IBD):TI,AB,KY,EH	8372
#8	#1 OR #2 OR #3 OR #4 OR #5 OR #6 OR #7	14918
#9	MESH DESCRIPTOR Steroids EXPLODE ALL TREES	68143
#10	MESH DESCRIPTOR Adrenal Cortex Hormones EXPLODE ALL TREES	32278
#11	MESH DESCRIPTOR Immunosuppressive Agents EXPLODE ALL TREES	28931
#12	MESH DESCRIPTOR Antimetabolites EXPLODE ALL TREES	34147
#13	MESH DESCRIPTOR Antibodies, Monoclonal, Humanized EXPLODE ALL TREES	13130
#14	(corticoster* OR Entocort OR glucocorticoid* OR betamethasone OR budesonide OR deflazacort OR dexamethason* OR prednison* OR prednisolone OR methylprednisolone OR triamcinolone OR thiopurine OR azathioprine OR Imuran OR AZA OR mercaptopurine OR 6-MP OR 6MP OR methotrexate OR aminosalicyl* OR 5-ASA* OR 5ASA OR mesalazine OR asacol OR mezavant OR pentasa OR salofalk OR sulfasalazine OR sulphasalazine OR salazosulphapyr* OR salazosulfapyr* OR salazopyrin OR azulfidine OR salicylazosulfapyr* OR salicylazosulphapyr* OR thioguanine OR tioguanine OR biological* OR immunosupp* OR antibod* OR infliximab OR adalimumab OR vedolizumab OR ustekinumab OR "certolizumab pegol" OR upadacitinib OR etrolizumab OR mirikizumab OR brazikumab OR risankizumab OR guselkumab OR filgotinib OR natalizumab OR abrilumab OR secukinumab OR tofacitinib OR brodalumab OR ozanimod OR etrasimod OR abatacept OR mongersen OR humira OR exemptia OR remicade OR ADA OR Cimzia OR Tysabri OR antegren):TI,AB,KY,EH	167770
#15	(vedolizumab OR secukinumab OR mirikizumab OR guselkumab OR etrolizumab OR Mesalamine OR brodalumab OR Sulfasalazine OR Adalimumab OR Certolizumab Pegol OR Natalizumab OR Infliximab OR abrilumab OR Azathioprine OR risankizumab OR Thioguanine OR Mercaptopurine OR Ustekinumab OR Glucocorticoids OR Abatacept OR Methotrexate OR ozanimod OR tofacitinib OR GLPG0634 OR upadacitinib OR etrasimod OR GED0301 OR Budesonide OR Triamcinolone OR Methylprednisolone):mh	19273
#16	#9 OR #10 OR #11 OR #12 OR #13 OR #14 OR #15	241379
#17	#8 AND #16	6798
#18	(clinicaltrials OR WHO):SO	479293
#19	conference*:PT	219535
#20	#18 OR #19	698822
#21	#17 NOT #20	3017

2) PICOTS

Population	Adults (aged 18 or older) with Crohn’s disease with or without previous treatment with biologicals
Intervention	Budesonide, prednisone, prednisolone, methylprednisolone, azathioprine, mercaptopurine, methotrexate, mesalazine, sulphasalazine, abrilumab, adalimumab, bodalumab, brazikumab, certolizumab pegol, etrolizumab, guselkumab, infliximab, mirikizumab, natalizumab, risankizumab, secukinumab, ustekinumab, vedolizumab, abatacept, betamethasone, deflazacort, dexamethasone, etrasimod, filgotinib, mongersen, ozanimod, tioguanine, tofacitinib, triamcinolonacetonide, upadacitinib.<br>Any combination therapy
Comparator	Another pharmacological intervention (listed as intervention), placebo, another dose of the same intervention, discontinuation of intervention.
Outcomes	Induction of clinical remission (ideally defined as Crohn’s Disease Activity Index (CDAI) < 150)<br>Induction of clinical response (ideally defined as CDAI decrease > 100)<br>Maintenance of clinical remission (ideally defined as CDAI < 150)<br>Steroid-free remission<br>Steroid-free response<br>Discontinuations<br>Discontinuations due to adverse events<br>Serious infections<br>Malignancies<br>Major cardiovascular events including thrombosis
Timing	Minimum duration of treatment of 2 weeks for induction treatment and 22 weeks for maintenance treatment.
Study design	Phase 3 RCTs. Phase 2 RCTs were included in case there were no phase 3 RCTs available.

3) Flow chart study selection

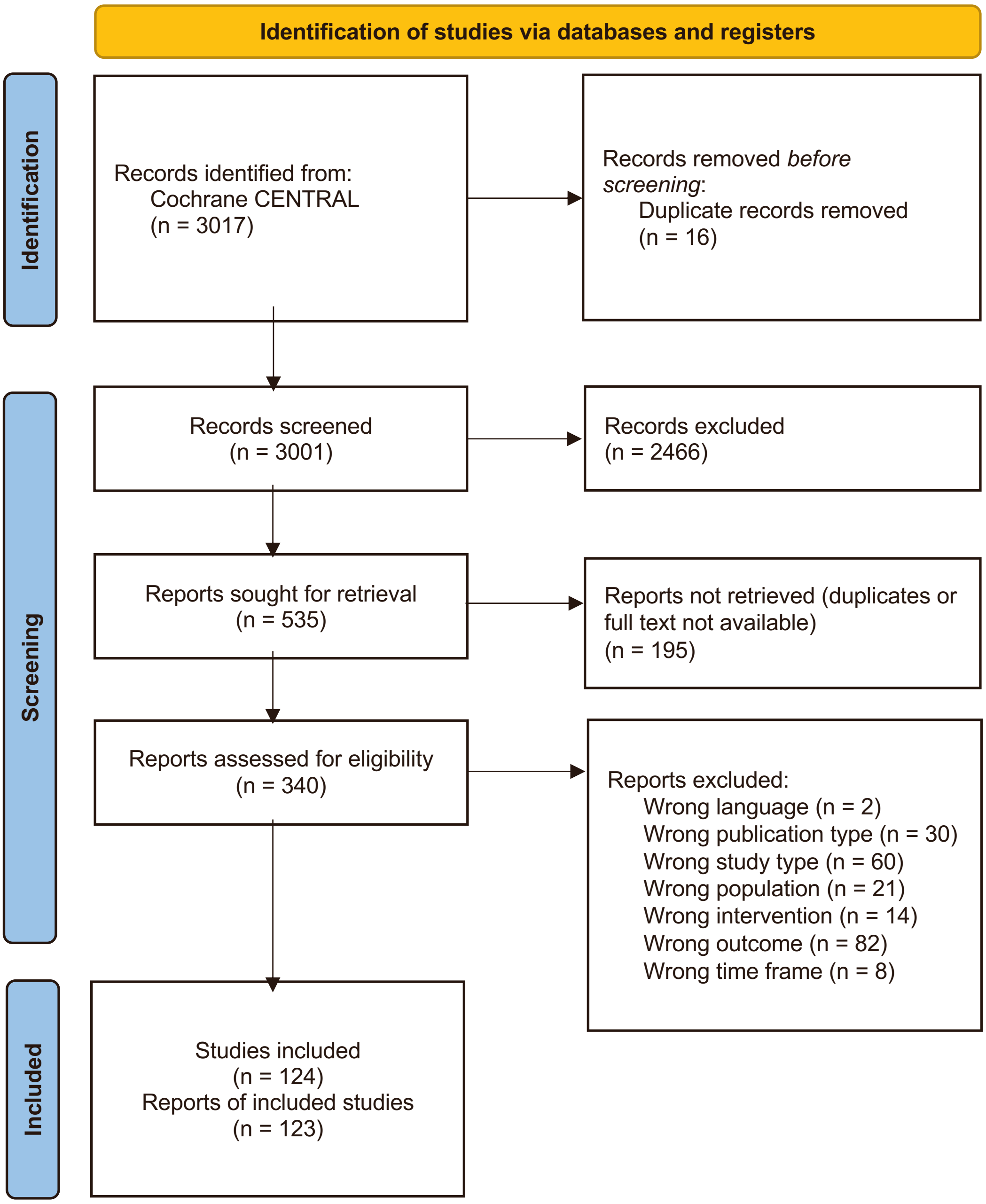

4) Overview of excluded studies

Reference	Reason for exclusion
No author (1999) – "Comparison of budesonide…"	Wrong publication type
No author (2017) – "Efficacy and safety…"	Wrong population
Abushamma (2023)	Wrong population
Adedokun (2016)	Wrong study type
Amiot (2017)	Wrong study type
Angelberger (2013)	Wrong outcome
Armuzzi (2013)	Wrong study type
Baert (1999)	Wrong outcome
Barbara (2016)	Wrong population
Bodini (2014)	Wrong study type
Bouguen (2015)	Wrong outcome
Brignola (1988)	Wrong study type
Brignola (1992)	Wrong time frame
Broekman (2017)	Wrong study type
Buisson (2021)	Wrong study type
Cada (2005)	Wrong publication type
Chao (2021)	Wrong intervention
Christian (2020)	Wrong outcome
Cohen (2002)	Wrong publication type
Colombel (2006)	Wrong publication type
Colombel (2009)	Wrong outcome
Colombel (2014)	Wrong outcome
Colombel (2015)	Wrong outcome
Colombel (2017)	Wrong outcome
Colombel (2019)	Wrong outcome
Conroy (2001)	Wrong publication type
Cornillie (2014)	Wrong outcome
Cortot (2001)	Wrong time frame
Cottone (2006)	Wrong publication type
D'Haens (1998)	Wrong outcome
D'Haens (1999)	Wrong outcome
D'Haens (2018)	Wrong study type
D'Haens (2021)	Wrong study type
D'Haens (2023)	Wrong study type
Danese (2019)	Wrong study type
Danese (2022)	Wrong intervention
Dassopoulos (2014)	Wrong population
De Cruz (2015)	Wrong study type
De Souza (2013)	Wrong population
Deibert (2003)	Wrong study type

Reference	Reason for exclusion
Dudley-Brown (2009)	Wrong outcome
Duijvestein (2020)	Wrong intervention
Dulai (2021)	Wrong population
East (2007)	Wrong study type
Edlund (2019)	Wrong study type
Egan (1999)	Wrong study type
Ewe (1989)	Wrong outcome
Farkas (2014)	Wrong population
Fasanmade (2011)	Wrong study type
Feagan (1998)	Wrong publication type
Feagan (2003)	Wrong outcome
Feagan (2005)	Wrong intervention
Feagan (2006)	Wrong intervention
Feagan (2007)	Wrong outcome
Feagan (2008) – "Effects of adalimumab…"	Wrong outcome
Feagan (2008) – "Treatment of active…"	Wrong study type
Feagan (2009)	Wrong outcome
Feagan (2010)	Wrong outcome
Feagan (2011) – "Evaluation of a…"	Wrong study type
Feagan (2011) – "Randomised clinical trial…"	Wrong outcome
Feagan (2014)	Wrong population
Feagan (2018) – "Efficacy of vedolizumab…"	Wrong outcome
Feagan (2018) – "Effects of Mongersen…"	Wrong study type
Feagan (2018) – "Performance of Crohn's…"	Wrong study type
Feagan (2018) – "Respiratory tract infections…"	Wrong outcome
Feagan (2018) – "Risankizumab in patients…"	Wrong study type
Feagan (2019)	Wrong outcome
Feagan (2022)	Wrong publication type
Feleshtynskly (2021)	Wrong intervention
Fishman (2001)	Wrong publication type
Florent (1996)	Wrong time frame
Fölsch (2010)	Wrong language
Geboes (2005)	Wrong outcome
Ghosh (2019)	Wrong outcome
Giaffer (1992)	Wrong population
Goll (2019)	Wrong study type
Greenberg (1996)	Wrong study type
Greenberg (2002)	Wrong publication type
Gu (2021)	Wrong publication type
Gupta Konijeti (2013)	Wrong publication type
Gurwara (2022)	Wrong publication type
Haifer (2021)	Wrong study type

Reference	Reason for exclusion
Hanauer (2004)	Wrong outcome
Hanauer (2010)	Wrong time frame
Hanauer (2020)	Wrong outcome
Hanzel (2022)	Wrong intervention
Herfarth (2004)	Wrong study type
Hisamatsu (2019)	Wrong outcome
Howaldt (1993)	Wrong intervention
Irvine (2000)	Wrong outcome
Irving (2007)	Wrong publication type
Isaacs (2006)	Wrong publication type
Jansen (2023)	Wrong outcome
Kiszka-Kanowitz (2016)	Wrong population
Klotz (1980)	Wrong outcome
Kotze (2015)	Wrong study type
Kucharzik (2023)	Wrong intervention
Laharie (2011)	Wrong study type
Laharie (2022)	Wrong study type
Lam (2014)	Wrong outcome
Landi (1992)	Wrong outcome
Li (2019)	Wrong outcome
Lichtenstein (2002)	Wrong outcome
Lichtenstein (2004)	Wrong outcome
Lichtenstein (2005)	Wrong outcome
Lichtenstein (2009) – "Clinical trial: benefits…"	Wrong outcome
Lichtenstein (2009) – "Oral budesonide for…"	Wrong outcome
Lichtenstein (2010)	Wrong study type
Lichtenstein (2012)	Wrong outcome
Lloyd-Still (1990)	Wrong publication type
Lofberg (1999)	Wrong publication type
Loftus (2008)	Wrong outcome
Louis (2023)	Wrong intervention
Mannon (2004)	Wrong intervention
Mascheretti (2002)	Wrong study type
Melmed (2016)	Wrong study type
Milassin (2019)	Wrong publication type
Mohamed (2019)	Wrong study type
Monteleone (2016)	Wrong study type
Narula (2021)	Wrong outcome
Narula (2022)	Wrong outcome
Ng (2018)	Wrong study type
O'Donoghue (1978)	Wrong outcome
Oren (2015)	Wrong publication type

Reference	Reason for exclusion
Panaccione (2010)	Wrong study type
Panaccione (2013)	Wrong study type
Panaccione (2018)	Wrong study type
Panaccione (2019)	Wrong outcome
Panés (2013)	Wrong population
Papamichael (2021)	Wrong outcome
Parikh (2013)	Wrong study type
Peyrin-Biroulet (2010)	Wrong publication type
Peyrin-Biroulet (2014)	Wrong outcome
Peyrin-Biroulet (2021)	Wrong outcome
Peyrin-Biroulet (2022)	Wrong outcome
Plosker (2007)	Wrong publication type
Present (1980)	Wrong study type
Present (1999)	Wrong outcome
Rasmussen (1987)	Wrong population
Regueiro (2009)	Wrong population
Regueiro (2011)	Wrong population
Regueiro (2014)	Wrong population
Reinisch (2012)	Wrong outcome
Reinisch (2017)	Wrong outcome
Reinisch (2021)	Wrong outcome
Rhodes (1970)	Wrong publication type
Rhodes (1971)	Wrong study type
Rhodes (1972)	Wrong publication type
Ricart (1999)	Wrong publication type
Rijk (1991)	Wrong outcome
Roblin (2017)	Wrong population
Roblin (2020)	Wrong population
Rosenberg (1975)	Wrong outcome
Roznowski (2001)	Wrong language
Rubin (2012)	Wrong outcome
Rutgeerts (1999)	Wrong publication type
Rutgeerts (2001)	Wrong publication type
Rutgeerts (2006) – "Onercept for moderate…"	Wrong intervention
Rutgeerts (2006) – "Scheduled maintenance treatment…"	Wrong outcome
Rutgeerts (2008)	Wrong outcome
Rutgeerts (2018)	Wrong outcome
Sandborn (1997)	Wrong publication type
Sandborn (2005)	Wrong outcome
Sandborn (2010) – "Certolizumab pegol in…"	Wrong time frame
Sandborn (2010) – "Reinduction with certolizumab…"	Wrong study type
Sandborn (2011)	Wrong study type

Reference	Reason for exclusion
Sandborn (2013)	Wrong outcome
Sandborn (2014)	Wrong study type
Sandborn (2018)	Wrong outcome
Sandborn (2021)	Wrong outcome
Sandborn (2022) – “Association between proposed…”	Wrong outcome
Sandborn (2022) – “Five-year efficacy…”	Wrong outcome
Sands (2004)	Wrong outcome
Sands (2006)	Wrong outcome
Sands (2018)	Wrong outcome
Schoon (2005)	Wrong outcome
Schreiber (1994)	Wrong population
Schreiber (2005)	Wrong study type
Schreiber (2007)	Wrong time frame
Schreiber (2010)	Wrong time frame
Schreiber (2011)	Wrong time frame
Schreiber (2013)	Wrong study type
Schulberg (2022)	Wrong population
Schwartz (2005)	Wrong publication type
Schwartz (2022)	Wrong outcome
Shao (2009)	Wrong study type
Singh (2019)	Wrong outcome
Singleton (1993)	Wrong study type
Singleton (1995)	Wrong outcome
Sluiter (2019)	Wrong population
Smedh (1995)	Wrong publication type
Smith (1978)	Wrong outcome
Steenholdt (2015)	Wrong outcome
Stricker (1998)	Wrong publication type
Strik (2018)	Wrong study type
Strik (2021)	Wrong intervention
Su (2018)	Wrong outcome
Syversen (2021)	Wrong intervention
Taxonera (2019)	Wrong outcome
Thomsen (2002)	Wrong outcome
Tillinger (1998)	Wrong outcome
Toedter (2009)	Wrong outcome
Treton (2009)	Wrong study type
Tursi (2014)	Wrong study type
Van Assche (2008)	Wrong population
Van Hees (1981)	Wrong study type
Vaysse (2016)	Wrong study type
Vermeire (2017)	Wrong study type

Reference	Reason for exclusion
Villen (2004)	Wrong study type
Watanabe (2014)	Wrong study type
Watanabe (2018)	Wrong outcome
Willoughby (1971)	Wrong study type
Winter (2004)	Wrong study type
Wong (2021)	Wrong outcome
Wong (2022)	Wrong study type
Yajnik (2017)	Wrong outcome
Yoshida (2012)	Wrong study type

References

1. Comparison of budesonide with mesalazine in Crohn's disease. Geneesmiddelenbulletin. 1999;33(2):21-2.
2. Efficacy and safety of ustekinumab in Japanese patients with moderately to severely active Crohn's disease: a subpopulation analysis of phase 3 induction and maintenance studies. Intestinal research. 2017;15(4):475-86.
3. Abushamma S, Walker T, Garza K, Chen L, Nix D, Chen CH**.** Accelerated Infliximab Infusion Safety and Tolerability Is Non-inferior to Standard Infusion Protocol in Inflammatory Bowel Disease Patients: a Randomized Controlled Study. Crohn's and colitis 360. 2023;5(3).
4. Adedokun OJ, Xu Z, Gasink C, Szapary P, Johanns J, Gao LL, et al. Pharmacokinetics and exposure-response relationships of ustekinumab during IV induction and SC maintenance treatment of patients with Crohn's disease with ustekinumab: results from the UNITI-1, UNITI-2, and IM-UNITI studies. Gastroenterology. 2016;150(4 Suppl 1):S408.
5. Amiot A, Gagniere C, Serrero M, Grimaud JC, Peyrin-Biroulet L, Zallot C, et al. One-year effectiveness and safety of vedolizumab therapy for inflammatory bowel disease: a prospective multicentre cohort study. Alimentary pharmacology & therapeutics. 2017;46(3):310-21.
6. Angelberger S, Schaeffeler E, Teml A, Petritsch W, Shonova O, Lukas M, et al. Mucosal improvement in patients with moderate to severe postoperative endoscopic recurrence of Crohn's disease and azathioprine metabolite levels. Inflammatory bowel diseases. 2013;19(3):590-8.
7. Armuzzi A, Felice C, Papa A, Marzo M, Pugliese D, Andrisani G, et al. Prevention of postoperative recurrence with azathioprine or infliximab in patients with Crohn's disease: an open-label pilot study. Journal of Crohn's & colitis. 2013;7(12):e623-9.
8. Baert FJ, D'Haens GR, Peeters M, Hiele MI, Schaible TF, Shealy D, et al. Tumor necrosis factor alpha antibody (infliximab) therapy profoundly down-regulates the inflammation in Crohn's ileocolitis. Gastroenterology. 1999;116(1):22-8.
9. Barbara G, Cremon C, Annese V, Basilisco G, Bazzoli F, Bellini M, et al. Randomised controlled trial of mesalazine in IBS. Gut. 2016;65(1):82-90.
10. Bodini G, Savarino V, Peyrin-Biroulet L, de Cassan C, Dulbecco P, Baldissarro I, et al. Low serum trough levels are associated with post-surgical recurrence in Crohn's disease patients undergoing prophylaxis with adalimumab. Digestive and liver disease. 2014;46(11):1043-6.

11. Bouguen G, Sninsky C, Tang KL, Colombel JF, D'Haens G, Kornbluth A, et al. Change in erythrocyte mean corpuscular volume during combination therapy with azathioprine and infliximab is associated with mucosal healing: a post hoc analysis from SONIC. Inflammatory bowel diseases. 2015;21(3):606-14.
12. Brignola C, Campieri M, Farruggia P, Tragnone A, Pasquali S, Iannone P, et al. The possible utility of steroids in the prevention of relapses of Crohn's disease in remission. A preliminary study. Journal of clinical gastroenterology. 1988;10(6):631-4.
13. Brignola C, Iannone P, Pasquali S, Campieri M, Gionchetti P, Belluzzi A, et al. Placebo-controlled trial of oral 5-ASA in relapse prevention of Crohn's disease. Digestive diseases and sciences. 1992;37(1):29-32.
14. Broekman M, Coenen MJH, Wanten GJ, van Marrewijk CJ, Klungel OH, Verbeek ALM, et al. Risk factors for thiopurine-induced myelosuppression and infections in inflammatory bowel disease patients with a normal TPMT genotype. Alimentary pharmacology & therapeutics. 2017;46(10):953-63.
15. Buisson A, Nancey S, Manlay L, Rubin DT, Hebuterne X, Pariente B, et al. Ustekinumab is more effective than azathioprine to prevent endoscopic postoperative recurrence in Crohn's disease. United European gastroenterology journal. 2021;9(5):552-60.
16. Cada DJ, Levien T, Baker DE. Natalizumab. Hospital pharmacy. 2005;40(4):336-46.
17. Chao K, Huang Y, Zhu X, Tang J, Wang X, Lin L, et al. Randomised clinical trial: dose optimising strategy by NUDT15 genotyping reduces leucopenia during thiopurine treatment of Crohn's disease. Alimentary pharmacology & therapeutics. 2021;54(9):1124-33.
18. Christian KE, Russman KM, Rajan DP, Barr EA, Cross RK. Gender Differences and Other Factors Associated with Weight Gain Following Initiation of Infliximab: a Post Hoc Analysis of Clinical Trials. Inflammatory bowel diseases. 2020;26(1):125-31.
19. Cohen RD. Infliximab as first-line therapy for severe Crohn's disease? Inflammatory bowel diseases. 2002;8(1):58-9; discussion 63.
20. Colombel JF. The CHARM trial of adalimumab in Crohn's disease. Gastroenterology and hepatology. 2006;2(7):486-8.
21. Colombel JF, Adedokun OJ, Gasink C, Gao LL, Cornillie FJ, D'Haens GR, et al. Combination Therapy With Infliximab and Azathioprine Improves Infliximab Pharmacokinetic Features and Efficacy: a Post Hoc Analysis. Clinical gastroenterology and hepatology. 2019;17(8):1525-32.e1.
22. Colombel JF, Jharap B, Sandborn WJ, Feagan B, Peyrin-Biroulet L, Eichner SF, et al. Effects of concomitant immunomodulators on the pharmacokinetics, efficacy and safety of adalimumab in patients with Crohn's disease or ulcerative colitis who had failed conventional therapy. Alimentary pharmacology & therapeutics. 2017;45(1):50-62.
23. Colombel JF, Reinisch W, Mantzaris GJ, Kornbluth A, Rutgeerts P, Tang KL, et al. Randomised clinical trial: deep remission in biologic and immunomodulator na□ve patients with Crohn's disease - a SONIC post hoc analysis. Alimentary pharmacology & therapeutics. 2015;41(8):734-46.
24. Colombel JF, Rutgeerts PJ, Sandborn WJ, Yang M, Camez A, Pollack PF, et al. Adalimumab induces deep remission in patients with Crohn's disease. Clinical gastroenterology and hepatology. 2014;12(3):414-22.e5.
25. Colombel JF, Schwartz DA, Sandborn WJ, Kamm MA, D'Haens G, Rutgeerts P, et al. Adalimumab for the treatment of fistulas in patients with Crohn's disease. Gut. 2009;58(7):940-8.

26. Conroy CA, Cattell R. Infliximab treatment for Crohn's disease. Postgraduate medical journal. 2001;77(909):436-40.
27. Cornillie F, Hanauer SB, Diamond RH, Wang J, Tang KL, Xu Z, et al. Postinduction serum infliximab trough level and decrease of C-reactive protein level are associated with durable sustained response to infliximab: a retrospective analysis of the ACCENT I trial. Gut. 2014;63(11):1721-7.
28. Cortot A, Colombel JF, Rutgeerts P, Lauritsen K, Malchow H, H□mling J, et al. Switch from systemic steroids to budesonide in steroid dependent patients with inactive Crohn's disease. Gut. 2001;48(2):186-90.
29. Cottone M, Mocciaro F, Scimeca D. Adalimumab induction for Crohn's disease. Gastroenterology. 2006;130(6):1929.
30. D'Haens G, Panes J, Louis E, Lacerda A, Zhou Q, Liu J, et al. Upadacitinib Was Efficacious and Well Tolerated Over 30 Months in Patients with Crohn's Disease in the CELEST Extension Study. Clinical gastroenterology and hepatology. 2021.
31. D'Haens G, Reinisch W, Schreiber S, Cummings F, Irving PM, Ye BD, et al. Subcutaneous Infliximab Monotherapy Versus Combination Therapy with Immunosuppressants in Inflammatory Bowel Disease: a Post Hoc Analysis of a Randomised Clinical Trial. Clinical drug investigation. 2023;43(4):277-88.
32. D'Haens G, Van Deventer S, Van Hogezand R, Chalmers D, Kothe C, Baert F, et al. Endoscopic and histological healing with infliximab anti-tumor necrosis factor antibodies in Crohn's disease: a European multicenter trial. Gastroenterology. 1999;116(5):1029-34.
33. D'Haens G, Vermeire S, Lambrecht G, Baert F, Bossuyt P, Pariente B, et al. Increasing Infliximab Dose Based on Symptoms, Biomarkers, and Serum Drug Concentrations Does Not Increase Clinical, Endoscopic, and Corticosteroid-Free Remission in Patients With Active Luminal Crohn's Disease. Gastroenterology. 2018;154(5):1343-51.e1.
34. D'Haens G, Verstraete A, Cheyns K, Aerden I, Bouillon R, Rutgeerts P. Bone turnover during short-term therapy with methylprednisolone or budesonide in Crohn's disease. Alimentary pharmacology & therapeutics. 1998;12(5):419-24.
35. Danese S, Vermeire S, D'Haens G, Pan□s J, Dignass A, Magro F, et al. Treat to target versus standard of care for patients with Crohn's disease treated with ustekinumab (STARDUST): an open-label, multicentre, randomised phase 3b trial. The lancet. Gastroenterology & hepatology. 2022;7(4):294-306.
36. Danese S, Vermeire S, Hellstern P, Panaccione R, Rogler G, Fraser G, et al. Randomised trial and open-label extension study of an anti-interleukin-6 antibody in Crohn's disease (ANDANTE I and II). Gut. 2019;68(1):40-8.
37. Dassopoulos T, Dubinsky MC, Bentsen JL, Martin CF, Galanko JA, Seidman EG, et al. Randomised clinical trial: individualised vs. weight-based dosing of azathioprine in Crohn's disease. Alimentary pharmacology & therapeutics. 2014;39(2):163-75.
38. De Cruz P, Kamm MA, Hamilton AL, Ritchie KJ, Krejany EO, Gorelik A, et al. Efficacy of thiopurines and adalimumab in preventing Crohn's disease recurrence in high-risk patients - a POCER study analysis. Alimentary pharmacology & therapeutics. 2015;42(7):867-79.
39. de Souza GS, Vidigal FM, Chebli LA, da Rocha Ribeiro TC, Furtado MC, de Lima Pace FH, et al. Effect of azathioprine or mesalazine therapy on incidence of re-hospitalization in sub-occlusive ileocecal Crohn's disease patients. Medical science monitor. 2013;19:716-22.
40. Deibert P, Dilger K, Fischer C, Hofmann U, Nauck S, Stoelben S, et al. High variation of tioguanine absorption in patients with chronic active Crohn's disease. Alimentary pharmacology & therapeutics. 2003;18(2):183-9.

41. Dudley-Brown S, Nag A, Cullinan C, Ayers M, Hass S, Panjabi S. Health-related quality-of-life evaluation of crohn disease patients after receiving natalizumab therapy. Gastroenterology nursing. 2009;32(5):327-39.
42. Duijvestein M, Jeyarajah J, Guizzetti L, Zou G, Parker CE, van Viegen T, et al. Response to Placebo, Measured by Endoscopic Evaluation of Crohn's Disease Activity, in a Pooled Analysis of Data from 5 Randomized Controlled Induction Trials. Clinical gastroenterology and hepatology. 2020;18(5):1121-32.e2.
43. Dulai PS, Jairath V, Zou G, Stitt LW, Khanna R, Sandborn WJ, et al. Early Combined Immunosuppression May Be More Effective for Reducing Complications in Isolated Colonic- vs Ileal-Dominant Crohn Disease. Inflammatory bowel diseases. 2021;27(5):639-46.
44. East JE, Brooker JC, Rutter MD, Saunders BP. A pilot study of intrastricture steroid versus placebo injection after balloon dilatation of Crohn's strictures. Clinical gastroenterology and hepatology. 2007;5(9):1065-9.
45. Edlund H, Grisic AM, Steenholdt C, Ainsworth MA, Brynskov J, Huisinga W, et al. Absence of Relationship Between Crohn's Disease Activity Index or C-Reactive Protein and Infliximab Exposure Calls for Objective Crohn's Disease Activity Measures for the Evaluation of Treatment Effects at Treatment Failure. Therapeutic drug monitoring. 2019;41(2):235-42.
46. Egan LJ, Sandborn WJ, Tremaine WJ, Leighton JA, Mays DC, Pike MG, et al. A randomized dose-response and pharmacokinetic study of methotrexate for refractory inflammatory Crohn's disease and ulcerative colitis. Alimentary pharmacology & therapeutics. 1999;13(12):1597-604.
47. Ewe K, Herfarth C, Malchow H, Jesdinsky HJ. Postoperative recurrence of Crohn's disease in relation to radicality of operation and sulfasalazine prophylaxis: a multicenter trial. Digestion. 1989;42(4):224-32.
48. Farkas K, B□lint A, Valkusz Z, Szepes Z, Nagy F, Sz?cs M, et al. Bolus administration of steroid therapy is more favorable than the conventional use in preventing decrease of bone density and the increase of body fat percentage in patients with inflammatory bowel disease. Journal of Crohn's & colitis. 2014;8(9):992-7.
49. Fasanmade AA, Adedokun OJ, Blank M, Zhou H, Davis HM. Pharmacokinetic Properties of Infliximab in Children and Adults with Crohn's Disease: a Retrospective Analysis of Data from 2 Phase III Clinical Trials. Clinical therapeutics. 2011;33(7):946-64.
50. Feagan B, Sandborn WJ, Rutgeerts P, Levesque BG, Khanna R, Huang B, et al. Performance of Crohn's disease Clinical Trial Endpoints based upon Different Cutoffs for Patient Reported Outcomes or Endoscopic Activity: analysis of EXTEND Data. Inflammatory bowel diseases. 2018;24(5):932-42.
51. Feagan BG. Aminosalicylates for active disease and in the maintenance of remission in Crohn's disease. European journal of surgery = Acta chirurgica. 1998;164(12):903-9.
52. Feagan BG, Bhayat F, Khalid M, Blake A, Travis SPL. Respiratory tract infections in patients with inflammatory bowel disease: safety analyses from vedolizumab clinical trials. Journal of Crohn's & colitis. 2018;12(8):905-19.
53. Feagan BG, Coteur G, Tan S, Keininger DL, Schreiber S. Clinically meaningful improvement in health-related quality of life in a randomized controlled trial of certolizumab pegol maintenance therapy for Crohn's disease. American journal of gastroenterology. 2009;104(8):1976-83.
54. Feagan BG, Greenberg GR, Wild G, Fedorak RN, Par□ P, McDonald JW, et al. Treatment of active Crohn's disease with MLN0002, a humanized antibody to the alpha4beta7 integrin. Clinical gastroenterology and hepatology. 2008;6(12):1370-7.

55. Feagan BG, Hanauer SB, Coteur G, Schreiber S. Evaluation of a daily practice composite score for the assessment of Crohn's disease: the treatment impact of certolizumab pegol. Alimentary pharmacology & therapeutics. 2011;33(10):1143-51.
56. Feagan BG, McDonald JW, Panaccione R, Enns RA, Bernstein CN, Ponich TP, et al. Methotrexate in combination with infliximab is no more effective than infliximab alone in patients with Crohn's disease. Gastroenterology. 2014;146(3):681-8.e1.
57. Feagan BG, Panaccione R, Sandborn WJ, D'Haens GR, Schreiber S, Rutgeerts PJ, et al. Effects of adalimumab therapy on incidence of hospitalization and surgery in Crohn's disease: results from the CHARM study. Gastroenterology. 2008;135(5):1493-9.
58. Feagan BG, Panes J, Ferrante M, Kaser A, D'Haens GR, Sandborn WJ, et al. Risankizumab in patients with moderate to severe Crohn's disease: an open-label extension study. The lancet gastroenterology and hepatology. 2018;3(10):671-80.
59. Feagan BG, Reilly MC, Gerlier L, Brabant Y, Brown M, Schreiber S. Clinical trial: the effects of certolizumab pegol therapy on work productivity in patients with moderate-to-severe Crohn's disease in the PRECiSE 2 study. Alimentary pharmacology & therapeutics. 2010;31(12):1276-85.
60. Feagan BG, Sandborn WJ, Baker JP, Cominelli F, Sutherland LR, Elson CO, et al. A randomized, double-blind, placebo-controlled trial of CDP571, a humanized monoclonal antibody to tumour necrosis factor-alpha, in patients with corticosteroid-dependent Crohn's disease. Alimentary pharmacology & therapeutics. 2005;21(4):373-84.
61. Feagan BG, Sandborn WJ, Colombel JF, Byrne SO, Khalid JM, Kempf C, et al. Incidence of Arthritis/Arthralgia in Inflammatory Bowel Disease with Long-term Vedolizumab Treatment: post Hoc Analyses of the GEMINI Trials. Journal of Crohn's & colitis. 2019;13(1):50-7.
62. Feagan BG, Sandborn WJ, Hass S, Niecko T, White J. Health-related quality of life during natalizumab maintenance therapy for Crohn's disease. American journal of gastroenterology. 2007;102(12):2737-46.
63. Feagan BG, Sandborn WJ, Lichtenstein G, Radford-Smith G, Patel J, Innes A. CDP571, a humanized monoclonal antibody to tumour necrosis factor-alpha, for steroid-dependent Crohn's disease: a randomized, double-blind, placebo-controlled trial. Alimentary pharmacology & therapeutics. 2006;23(5):617-28.
64. Feagan BG, Sandborn WJ, Wolf DC, Coteur G, Purcaru O, Brabant Y, et al. Randomised clinical trial: improvement in health outcomes with certolizumab pegol in patients with active Crohn's disease with prior loss of response to infliximab. Alimentary pharmacology & therapeutics. 2011;33(5):541-50.
65. Feagan BG, Sands BE, Rossiter G, Li X, Usiskin K, Zhan X, et al. Effects of Mongersen (GED-0301) on Endoscopic and Clinical Outcomes in Patients With Active Crohn's Disease. Gastroenterology. 2018;154(1):61-4.e6.
66. Feagan BG, Schreiber S, Afzali A, Rieder F, Hyams J, Kollengode K, et al. Ozanimod as a novel oral small molecule therapy for the treatment of Crohn's disease: the YELLOWSTONE clinical trial program. Contemporary clinical trials. 2022;122:106958.
67. Feagan BG, Schwartz D, Danese S, Rubin DT, Lissoos TW, Xu J, et al. Efficacy of vedolizumab in fistulising Crohn's disease: exploratory analyses of data from GEMINI 2. Journal of Crohn's & colitis. 2018;12(5):621-6.
68. Feagan BG, Yan S, Bala M, Bao W, Lichtenstein GR. The effects of infliximab maintenance therapy on health-related quality of life. American journal of gastroenterology. 2003;98(10):2232-8.

69. Feleshtynskiy Y, Mylianovska A, Pirogovsky V, Dyadyk O. Evaluation of the endoscopic treatment with topical prednisolone administration for intestinal strictures in Crohn's disease. Polski przeglad chirurgiczny. 2021;94(1):28-33.
70. Fishman M. Methotrexate and maintenance of remission in Crohn's disease. Journal canadien de gastroenterologie [Canadian journal of gastroenterology]. 2001;15(7):428.
71. Florent C, Cortot A, Quandale P, Sahmound T, Modigliani R, Sarfaty E, et al. Placebo-controlled clinical trial of mesalazine in the prevention of early endoscopic recurrences after resection for Crohn's disease. Groupe d'Etudes Th□rapeutiques des Affections Inflammatoires Digestives (GETAID). European journal of gastroenterology & hepatology. 1996;8(3):229-33.
72. F□lsch UR, Nitschmann S. New therapeutic option for M. Crohn : SONIC study (study of biologic and immunomodulator naive patients in Crohn's disease). Der Internist. 2010;51(9):1202-4.
73. Geboes K, Rutgeerts P, Opdenakker G, Olson A, Patel K, Wagner CL, et al. Endoscopic and histologic evidence of persistent mucosal healing and correlation with clinical improvement following sustained infliximab treatment for Crohn's disease. Current medical research and opinion. 2005;21(11):1741-54.
74. Ghosh S, Gensler LS, Yang Z, Gasink C, Chakravarty SD, Farahi K, et al. Ustekinumab Safety in Psoriasis, Psoriatic Arthritis, and Crohn's Disease: an Integrated Analysis of Phase II/III Clinical Development Programs. Drug safety. 2019;42(6):751-68.
75. Giaffer MH, O'Brien CJ, Holdsworth CD. Clinical tolerance to three 5-aminosalicylic acid releasing preparations in patients with inflammatory bowel disease intolerant or allergic to sulphasalazine. Alimentary pharmacology & therapeutics. 1992;6(1):51-9.
76. Goll GL, J□rgensen KK, Sexton J, Olsen IC, Bolstad N, Haavardsholm EA, et al. Long-term efficacy and safety of biosimilar infliximab (CT-P13) after switching from originator infliximab: open-label extension of the NOR-SWITCH trial. Journal of internal medicine. 2019;285(6):653-69.
77. Greenberg GR. Infliximab as first-line therapy for Crohn's disease is premature. Inflammatory bowel diseases. 2002;8(1):60-2; discussion 3.
78. Greenberg GR, Feagan BG, Martin F, Sutherland LR, Thomson AB, Williams CN, et al. Oral budesonide as maintenance treatment for Crohn's disease: a placebo-controlled, dose-ranging study. Canadian Inflammatory Bowel Disease Study Group. Gastroenterology. 1996;110(1):45-51.
79. Gu B, De Gregorio M, Pipicella JL, Vande Casteele N, Andrews JM, Begun J, et al. Prospective randomised controlled trial of adults with perianal fistulising Crohn's disease and optimised therapeutic infliximab levels: PROACTIVE trial study protocol. BMJ open. 2021;11(7):e043921.
80. Gupta Konijeti G, Chan AT. Ustekinumab for moderate-to-severe crohn's disease. Gastroenterology. 2013;144(4):846-8.
81. Gurwara S, Hou JK. Shooting for the Stars: review of the STARDUST Trial and the Treat-to-Target Approach for Crohn's Disease. Gastroenterology and hepatology. 2022;18(5):243-7.
82. Haifer C, Srinivasan A, An YK, Picardo S, van Langenberg D, Menon S, et al. Switching Australian patients with moderate to severe inflammatory bowel disease from originator to biosimilar infliximab: a multicentre, parallel cohort study. Medical journal of Australia. 2021;214(3):128-33.
83. Hanauer SB, Panes J, Colombel JF, Bloomfield R, Schreiber S, Sandborn WJ. Clinical trial: impact of prior infliximab therapy on the clinical response to certolizumab pegol maintenance therapy for Crohn's disease. Alimentary pharmacology & therapeutics. 2010;32(3):384-93.

84. Hanauer SB, Sandborn WJ, Feagan BG, Gasink C, Jacobstein D, Zou B, et al. IM-UNITI: three-year Efficacy, Safety, and Immunogenicity of Ustekinumab Treatment of Crohn's Disease. Journal of Crohn's & colitis. 2020;14(1):23-32.
85. Hanauer SB, Wagner CL, Bala M, Mayer L, Travers S, Diamond RH, et al. Incidence and importance of antibody responses to infliximab after maintenance or episodic treatment in Crohn's disease. Clinical gastroenterology and hepatology. 2004;2(7):542-53.
86. Hanzel J, Ma C, Zou G, Singh S, Dulai PS, Feagan BG, et al. Early Combined Immunosuppression Reduces Complications in Long-standing Crohn's Disease: a Post Hoc Analysis of REACT. Clinical gastroenterology and hepatology. 2022;20(1):236-8.
87. Herfarth H, Gross V, Andus T, Caesar I, Vogelsang H, Adler G, et al. Analysis of the therapeutic efficacy of different doses of budesonide in patients with active Crohn's ileocolitis depending on disease activity and localization. International journal of colorectal disease. 2004;19(2):147-52.
88. Hisamatsu T, Matsumoto T, Watanabe K, Nakase H, Motoya S, Yoshimura N, et al. Concerns and Side Effects of Azathioprine During Adalimumab Induction and Maintenance Therapy for Japanese Patients With Crohn's Disease: a Subanalysis of a Prospective Randomised Clinical Trial. Journal of Crohn's & colitis. 2019;13(9):1097-104.
89. Howaldt S, Raedler A, Reinecker HC, Berghaus D, Hoyer S, Kaiser B, et al. Comparative trial of remission prophylaxis in quiescent Crohn's disease with oral 4 aminosalicylic acid versus 5 aminosalicylic acid slow release tablets. Canadian journal of gastroenterology. 1993;7:241-4.
90. Irvine EJ, Greenberg GR, Feagan BG, Martin F, Sutherland LR, Thomson AB, et al. Quality of life rapidly improves with budesonide therapy for active Crohn's disease. Canadian Inflammatory Bowel Disease Study Group. Inflammatory bowel diseases. 2000;6(3):181-7.
91. Irving PM, Gibson PR. Infliximab: getting the most for your money. Journal of gastroenterology and hepatology. 2007;22(10):1559-61.
92. Isaacs KL. Adalimumab induction therapy in Crohn disease. Evidence-based gastroenterology. 2006;7(3):67-8.
93. Jansen FM, van Linschoten RCA, Kievit W, Smits LJT, Pauwels RWM, de Jong DJ, et al. Cost-effectiveness analysis of increased adalimumab dose intervals in Crohn's disease patients in stable remission: the randomised controlled LADI trial. Journal of Crohn's & colitis. 2023.
94. Kiszka-Kanowitz M, Theede K, Mertz-Nielsen A. Randomized clinical trial: a pilot study comparing efficacy of low-dose azathioprine and allopurinol to azathioprine on clinical outcomes in inflammatory bowel disease. Scandinavian journal of gastroenterology. (pp 1-6), 2016. Date of publication: 09 aug 2016. 2016.
95. Klotz U, Maier K, Fischer C, Heinkel K. Therapeutic efficacy of sulfasalazine and its metabolites in patients with ulcerative colitis and Crohn's disease. New England journal of medicine. 1980;303(26):1499-502.
96. Kotze PG, Yamamoto T, Danese S, Suzuki Y, Teixeira FV, De Albuquerque IC, et al. Direct retrospective comparison of adalimumab and infliximab in preventing early postoperative endoscopic recurrence after ileocaecal resection for Crohn's disease: results from the MULTIPER database. Journal of Crohn's & colitis. 2015;9(7):541-7.
97. Kucharzik T, Wilkens R, D'Agostino MA, Maconi G, Le Bars M, Lahaye M, et al. Early ultrasound response and progressive transmural remission after treatment with ustekinumab in Crohn's disease. Clinical gastroenterology and hepatology. 2023;21(1):153-63.e12.

98. Laharie D, D'Haens G, Nachury M, Lambrecht G, Bossuyt P, Bouhnik Y, et al. Steroid-Free Deep Remission at One Year Does Not Prevent Crohn's Disease Progression: long-Term Data From the TAILORIX Trial. Clinical gastroenterology and hepatology. 2022;20(9):2074-82.
99. Laharie D, Reffet A, Belleann□e G, Chabrun E, Subtil C, Razaire S, et al. Mucosal healing with methotrexate in Crohn's disease: a prospective comparative study with azathioprine and infliximab. Alimentary pharmacology & therapeutics. 2011;33(6):714-21.
100. Lam MC, Bressler B. Vedolizumab for ulcerative colitis and Crohn's disease: results and implications of GEMINI studies. Immunotherapy. 2014;6(9):963-71.
101. Landi B, Anh TN, Cortot A, Soule JC, Rene E, Gendre JP, et al. Endoscopic monitoring of Crohn's disease treatment: a prospective, randomized clinical trial. The Groupe d'Etudes Therapeutiques des Affections Inflammatoires Digestives. Gastroenterology. 1992;102(5):1647-53.
102. Li K, Friedman JR, Chan D, Pollack P, Yang F, Jacobstein D, et al. Effects of Ustekinumab on Histologic Disease Activity in Patients With Crohn's Disease. Gastroenterology. 2019;157(4):1019-31.e7.
103. Lichtenstein GR, Bala M, Han C, DeWoody K, Schaible T. Infliximab improves quality of life in patients with Crohn's disease. Inflammatory bowel diseases. 2002;8(4):237-43.
104. Lichtenstein GR, Bengtsson B, Hapten-White L, Rutgeerts P. Oral budesonide for maintenance of remission of Crohn's disease: a pooled safety analysis. Alimentary pharmacology & therapeutics. 2009;29(6):643-53.
105. Lichtenstein GR, Diamond RH, Wagner CL, Fasanmade AA, Olson AD, Marano CW, et al. Clinical trial: benefits and risks of immunomodulators and maintenance infliximab for IBD-subgroup analyses across four randomized trials. Alimentary pharmacology & therapeutics. 2009;30(3):210-26.
106. Lichtenstein GR, Rutgeerts P, Sandborn WJ, Sands BE, Diamond RH, Blank M, et al. A pooled analysis of infections, malignancy, and mortality in infliximab- and immunomodulator-treated adult patients with inflammatory bowel disease. American journal of gastroenterology. 2012;107(7):1051-63.
107. Lichtenstein GR, Thomsen O, Schreiber S, Lawrance IC, Hanauer SB, Bloomfield R, et al. Continuous therapy with certolizumab pegol maintains remission of patients with Crohn's disease for up to 18 months. Clinical gastroenterology and hepatology. 2010;8(7):600-9.
108. Lichtenstein GR, Yan S, Bala M, Blank M, Sands BE. Infliximab maintenance treatment reduces hospitalizations, surgeries, and procedures in fistulizing Crohn's disease. Gastroenterology. 2005;128(4):862-9.
109. Lichtenstein GR, Yan S, Bala M, Hanauer S. Remission in patients with Crohn's disease is associated with improvement in employment and quality of life and a decrease in hospitalizations and surgeries. American journal of gastroenterology. 2004;99(1):91-6.
110. Lloyd-Still JD. Azathioprine and the treatment of chronic inflammatory bowel disease. Journal of pediatrics. 1990;117(5):732-5.
111. Lofberg R. Treatment of fistulas in Crohn's disease with infliximab. Gut. 1999;45(5):642-3.
112. Loftus EV, Feagan BG, Colombel JF, Rubin DT, Wu EQ, Yu AP, et al. Effects of adalimumab maintenance therapy on health-related quality of life of patients with Crohn's disease: patient-reported outcomes of the CHARM trial. American journal of gastroenterology. 2008;103(12):3132-41.

113. Louis E, Resche-Rigon M, Laharie D, Satsangi J, Ding N, Siegmund B, et al. Withdrawal of infliximab or concomitant immunosuppressant therapy in patients with Crohn's disease on combination therapy (SPARE): a multicentre, open-label, randomised controlled trial. The lancet. Gastroenterology & hepatology. 2023;8(3):215-27.
114. Mannon PJ, Fuss IJ, Mayer L, Elson CO, Sandborn WJ, Present D, et al. Anti-interleukin-12 antibody for active Crohn's disease. New England journal of medicine. 2004;351(20):2069-79.
115. Mascheretti S, Hampe J, Croucher PJ, Nikolaus S, Andus T, Schubert S, et al. Response to infliximab treatment in Crohn's disease is not associated with mutations in the CARD15 (NOD2) gene: an analysis in 534 patients from two multicenter, prospective GCP-level trials. Pharmacogenetics. 2002;12(7):509-15.
116. Melmed GY, McGovern D, Schreiber S, Kosutic G, Spearman M, Coarse J, et al. Early remission status predicts long-term outcomes in patients with Crohn's disease treated with certolizumab pegol. Current medical research and opinion. 2016;32(12):1937-41.
117. Milassin A, Fabian A, Molnar T. Switching from infliximab to biosimilar in inflammatory bowel disease: overview of the literature and perspective. Therapeutic advances in gastroenterology. 2019;12.
118. Mohamed ME, Klunder B, Lacerda AP, Othman AA. Exposure□??Response Analyses for Upadacitinib Efficacy and Safety in the Crohn's Disease CELEST Study and Bridging to the Extended-Release Formulation. Clinical pharmacology and therapeutics. 2019.
119. Monteleone G, Di Sabatino A, Ardizzone S, Pallone F, Usiskin K, Zhan X, et al. Impact of patient characteristics on the clinical efficacy of mongersen (GED-0301), an oral Smad7 antisense oligonucleotide, in active Crohn's disease. Alimentary pharmacology & therapeutics. 2016;43(6):717-24.
120. Narula N, Aruljothy A, Wong ECL, Homenauth R, Alshahrani AA, Marshall JK, et al. The impact of ustekinumab on extraintestinal manifestations of Crohn's disease: a post hoc analysis of the UNITI studies. United European gastroenterology journal. 2021;9(5):581-9.
121. Narula N, Wong ECL, Dulai PS, Sengupta NK, Marshall JK, Colombel JF, et al. Comparative Efficacy and Rapidity of Action for Infliximab vs Ustekinumab in Biologic Na□ve Crohn's Disease. Clinical gastroenterology and hepatology. 2022;20(7):1579-87.e2.
122. Ng SC, Hilmi IN, Blake A, Bhayat F, Adsul S, Khan QR, et al. Low Frequency of Opportunistic Infections in Patients Receiving Vedolizumab in Clinical Trials and Post-Marketing Setting. Inflammatory bowel diseases. 2018;24(11):2431-41.
123. O'Donoghue DP, Dawson AM, Powell-Tuck J, Bown RL, Lennard-Jones JE. Double-blind withdrawal trial of azathioprine as maintenance treatment for Crohn's disease. Lancet (london, england). 1978;2(8097):955-7.
124. Oren R, Moshkowitz M, Odes S, Becker S, Keter D, Pomeranz I, et al. Erratum: methotrexate in chronic active crohn's disease: a double-blind, randomized, israeli multicenter trial (American Journal of Gastroenterology (1997) 92 (2203-2209)). American journal of gastroenterology. 2015;110(4):608.
125. Panaccione R, Colombel JF, Sandborn WJ, D'Haens G, Zhou Q, Pollack PF, et al. Adalimumab maintains remission of Crohn's disease after up to 4 years of treatment: data from CHARM and ADHERE. Alimentary pharmacology & therapeutics. 2013;38(10):1236-47.
126. Panaccione R, Colombel JF, Sandborn WJ, Rutgeerts P, D'Haens GR, Robinson AM, et al. Adalimumab sustains clinical remission and overall clinical benefit after 2 years of

therapy for Crohn's disease. Alimentary pharmacology & therapeutics. 2010;31(12):1296-309.
127. Panaccione R, L□fberg R, Rutgeerts P, Sandborn WJ, Schreiber S, Berg S, et al. Efficacy and Safety of Adalimumab by Disease Duration: analysis of Pooled Data From Crohn's Disease Studies. Journal of Crohn's & colitis. 2019;13(6):725-34.
128. Panaccione R, Sandborn WJ, D'Haens G, Wolf DC, Berg S, Maa JF, et al. Clinical benefit of long-term adalimumab treatment in patients with Crohn's disease following loss of response or intolerance to infliximab: 96-week efficacy data from gain/adhere trials. Journal of Crohn's & colitis. 2018;12(8):930-8.
129. Pan□s J, L□pez-Sanrom□n A, Bermejo F, Garc□a-S□nchez V, Esteve M, Torres Y, et al. Early azathioprine therapy is no more effective than placebo for newly diagnosed Crohn's disease. Gastroenterology. 2013;145(4):766-74.e1.
130. Papamichael K, Vande Casteele N, Jeyarajah J, Jairath V, Osterman MT, Cheifetz AS. Higher Postinduction Infliximab Concentrations Are Associated With Improved Clinical Outcomes in Fistulizing Crohn's Disease: an ACCENT-II Post Hoc Analysis. American journal of gastroenterology. 2021;116(5):1007-14.
131. Parikh A, Fox I, Leach T, Xu J, Scholz C, Patella M, et al. Long-term clinical experience with vedolizumab in patients with inflammatory bowel disease. Inflammatory bowel diseases. 2013;19(8):1691-9.
132. Peyrin-Biroulet L, Ghosh S, Lee SD, Lee WJ, Griffith J, Wallace K, et al. Effect of risankizumab on health-related quality of life in patients with Crohn's disease: results from phase 3 MOTIVATE, ADVANCE and FORTIFY clinical trials. Alimentary pharmacology & therapeutics. 2022.
133. Peyrin-Biroulet L, Louis E, Loftus EV, Lacerda A, Zhou Q, Sanchez Gonzalez Y, et al. Quality of Life and Work Productivity Improvements with Upadacitinib: phase 2b Evidence from Patients with Moderate to Severe Crohn's Disease. Advances in therapy. 2021;38(5):2339-52.
134. Peyrin-Biroulet L, Reinisch W, Colombel JF, Mantzaris GJ, Kornbluth A, Diamond R, et al. Clinical disease activity, C-reactive protein normalisation and mucosal healing in Crohn's disease in the SONIC trial. Gut. 2014;63(1):88-95.
135. Peyrinbiroulet L, Danese S. Infliximab and azathioprine for Crohn's disease: a super-sonic combination? Gastroenterology. 2010;139(3):1059-61.
136. Plosker GL, Lyseng-Williamson KA. Adalimumab: in Crohn's disease. BioDrugs. 2007;21(2):125-32; discussion 33.
137. Present DH, Korelitz BI, Wisch N, Glass JL, Sachar DB, Pasternack BS. Treatment of Crohn's disease with 6-mercaptopurine. A long-term, randomized, double-blind study. New England journal of medicine. 1980;302(18):981-7.
138. Present DH, Rutgeerts P, Targan S, Hanauer SB, Mayer L, van Hogezand RA, et al. Infliximab for the treatment of fistulas in patients with Crohn's disease. New England journal of medicine. 1999;340(18):1398-405.
139. Rasmussen SN, Lauritsen K, Tage-Jensen U, Nielsen OH, Bytzer P, Jacobsen O, et al. 5-Aminosalicylic acid in the treatment of Crohn's disease. A 16-week double-blind, placebo-controlled, multicentre study with Pentasa. Scandinavian journal of gastroenterology. 1987;22(7):877-83.
140. Regueiro M, El-Hachem S, Kip KE, Schraut W, Baidoo L, Watson A, et al. Postoperative infliximab is not associated with an increase in adverse events in Crohn's disease. Digestive diseases and sciences. 2011;56(12):3610-5.
141. Regueiro M, Kip KE, Baidoo L, Swoger JM, Schraut W. Postoperative therapy with infliximab prevents long-term Crohn's disease recurrence. Clinical gastroenterology and hepatology. 2014;12(9):1494-502.e1.

142. Regueiro M, Schraut W, Baidoo L, Kip KE, Sepulveda AR, Pesci M, et al. Infliximab prevents Crohn's disease recurrence after ileal resection. Gastroenterology. 2009;136(2):441-50.e1; quiz 716.
143. Reinisch W, Colombel JF, D'Haens G, Sandborn WJ, Rutgeerts P, Geboes K, et al. Characterisation of Mucosal Healing with Adalimumab Treatment in Patients with Moderately to Severely Active Crohn's Disease: results from the EXTEND Trial. Journal of Crohn's & colitis. 2017;11(4):425-34.
144. Reinisch W, Mishkin DS, Oh YS, Schreiber S, Hussain F, Jacob R, et al. Impact of various central endoscopy reading models on treatment outcome in Crohn's disease using data from the randomized, controlled, exploratory cohort arm of the BERGAMOT trial. Gastrointestinal endoscopy. 2021;93(1):174-82.e2.
145. Reinisch W, Wang Y, Oddens BJ, Link R. C-reactive protein, an indicator for maintained response or remission to infliximab in patients with Crohn's disease: a post-hoc analysis from ACCENT I. Alimentary pharmacology & therapeutics. 2012;35(5):568-76.
146. Rhodes J. Azathioprine in the treatment of Crohn's disease. British journal of surgery. 1972;59(10):819-21.
147. Rhodes J, Bainton D, Beck P. Azathioprine in Crohn's disease. Lancet (london, england). 1970;2(7683):1142.
148. Rhodes J, Bainton D, Beck P, Campbell H. Controlled trial of azathioprine in Crohn's disease. Lancet (london, england). 1971;2(7737):1273-6.
149. Ricart E, Sandborn WJ. Infliximab for the treatment of fistulas in patients with Crohn'S disease. Gastroenterology. 1999;117(5):1247-8.
150. Rijk MC, van Hogezand RA, van Lier HJ, van Tongeren JH. Sulphasalazine and prednisone compared with sulphasalazine for treating active Crohn disease. A double-blind, randomized, multicenter trial. Annals of internal medicine. 1991;114(6):445-50.
151. Roblin X, Boschetti G, Williet N, Nancey S, Marotte H, Berger A, et al. Azathioprine dose reduction in inflammatory bowel disease patients on combination therapy: an open-label, prospective and randomised clinical trial. Alimentary pharmacology & therapeutics. 2017;46(2):142-9.
152. Roblin X, Williet N, Boschetti G, Phelip JM, Del Tedesco E, Berger AE, et al. Addition of azathioprine to the switch of anti-TNF in patients with IBD in clinical relapse with undetectable anti-TNF trough levels and antidrug antibodies: a prospective randomised trial. Gut. 2020;69(7):1206-12.
153. Rosenberg JL, Levin B, Wall AJ, Kirsner JB. A controlled trial of azathioprine in Crohn's disease. American journal of digestive diseases. 1975;20(8):721-6.
154. Roznowski AB, Dignass A. Sustaining remission in Crohn disease with methotrexate--a placebo controlled study. Zeitschrift fur Gastroenterologie. 2001;39(3):265-7.
155. Rubin DT, Mulani P, Chao J, Pollack PF, Bensimon AG, Yu AP, et al. Effect of adalimumab on clinical laboratory parameters in patients with Crohn's disease: results from the CHARM trial. Inflammatory bowel diseases. 2012;18(5):818-25.
156. Rutgeerts P. Budesonide led to a greater remission rate and fewer severe adverse events than did mesalamine in Crohn's disease. Gut. 1999;45(1):13-4.
157. Rutgeerts P. Little benefit from mesalazine taken prophylactically after surgery for Crohn's disease. Gut. 2001;48(4):452-3.
158. Rutgeerts P, Diamond RH, Bala M, Olson A, Lichtenstein GR, Bao W, et al. Scheduled maintenance treatment with infliximab is superior to episodic treatment for the healing of mucosal ulceration associated with Crohn's disease. Gastrointestinal endoscopy. 2006;63(3):433-42; quiz 64.

159. Rutgeerts P, Gasink C, Chan D, Lang Y, Pollack P, Colombel JF, et al. Efficacy of Ustekinumab for Inducing Endoscopic Healing in Patients With Crohn's Disease. Gastroenterology. 2018;155(4):1045-58.
160. Rutgeerts P, Sandborn WJ, Fedorak RN, Rachmilewitz D, Tarabar D, Gibson P, et al. Onercept for moderate-to-severe Crohn's disease: a randomized, double-blind, placebo-controlled trial. Clinical gastroenterology and hepatology. 2006;4(7):888-93.
161. Rutgeerts P, Schreiber S, Feagan B, Keininger DL, O'Neil L, Fedorak RN, et al. Certolizumab pegol, a monthly subcutaneously administered Fc-free anti-TNFalpha, improves health-related quality of life in patients with moderate to severe Crohn's disease. International journal of colorectal disease. 2008;23(3):289-96.
162. Sandborn WJ**.** A controlled trial of anti-tumor necrosis factor alpha antibody for Crohn's disease. Gastroenterology. 1997;113(3):1042-3.
163. Sandborn WJ, Abreu MT, D'Haens G, Colombel JF, Vermeire S, Mitchev K, et al. Certolizumab pegol in patients with moderate to severe Crohn's disease and secondary failure to infliximab. Clinical gastroenterology and hepatology. 2010;8(8):688-95.e2.
164. Sandborn WJ, Colombel JF, Pan□s J, Castillo M, Robinson AM, Zhou Q, et al. Exploring the use of adalimumab for patients with moderate Crohn's disease: subanalyses from induction and maintenance trials. Journal of Crohn's & colitis. 2013;7(12):958-67.
165. Sandborn WJ, Colombel JF, Schreiber S, Plevy SE, Pollack PF, Robinson AM, et al. Dosage adjustment during long-term adalimumab treatment for Crohn's disease: clinical efficacy and pharmacoeconomics. Inflammatory bowel diseases. 2011;17(1):141-51.
166. Sandborn WJ, Feagan BG, Danese S, O'Brien CD, Ott E, Marano C, et al. Safety of Ustekinumab in Inflammatory Bowel Disease: pooled Safety Analysis of Results from Phase 2/3 Studies. Inflammatory bowel diseases. 2021;27(7):994-1007.
167. Sandborn WJ, Lee SD, Randall C, Gutierrez A, Schwartz DA, Ambarkhane S, et al. Long-term safety and efficacy of certolizumab pegol in the treatment of Crohn's disease: 7-year results from the PRECiSE 3 study. Alimentary pharmacology & therapeutics. 2014;40(8):903-16.
168. Sandborn WJ, Lewis JD, Panes J, Loftus EV, D'Haens G, Yu Z, et al. Association Between Proposed Definitions of Clinical Remission/Response and Well-Being in Patients With Crohn's Disease. Journal of Crohn's & colitis. 2022;16(3):444-51.
169. Sandborn WJ, L□fberg R, Feagan BG, Hanauer SB, Campieri M, Greenberg GR**.** Budesonide for maintenance of remission in patients with Crohn's disease in medically induced remission: a predetermined pooled analysis of four randomized, double-blind, placebo-controlled trials. American journal of gastroenterology. 2005;100(8):1780-7.
170. Sandborn WJ, Rebuck R, Wang Y, Zou B, Adedokun OJ, Gasink C, et al. Five-Year Efficacy and Safety of Ustekinumab Treatment in Crohn's Disease: the IM-UNITI Trial. Clinical gastroenterology and hepatology. 2022;20(3):578-90.e4.
171. Sandborn WJ, Rutgeerts P, Gasink C, Jacobstein D, Zou B, Johanns J, et al. Long-term efficacy and safety of ustekinumab for Crohn's disease through the second year of therapy. Alimentary pharmacology & therapeutics. 2018;48(1):65-77.
172. Sandborn WJ, Schreiber S, Hanauer SB, Colombel JF, Bloomfield R, Lichtenstein GR, et al. Reinduction with certolizumab pegol in patients with relapsed Crohn's disease: results from the PRECiSE 4 Study. Clinical gastroenterology and hepatology. 2010;8(8):696-702.e1.
173. Sands BE, Blank MA, Diamond RH, Barrett JP, Van Deventer SJ**.** Maintenance infliximab does not result in increased abscess development in fistulizing Crohn's

disease: results from the ACCENT II study. Alimentary pharmacology & therapeutics. 2006;23(8):1127-36.
174. Sands BE, Blank MA, Patel K, van Deventer SJ, Study AI. Long-term treatment of rectovaginal fistulas in Crohn's disease: response to infliximab in the ACCENT II Study. Clinical gastroenterology and hepatology. 2004;2(10):912-20.
175. Sands BE, Han C, Gasink C, Jacobstein D, Szapary P, Gao LL, et al. The Effects of Ustekinumab on Health-related Quality of Life in Patients With Moderate to Severe Crohn's Disease. Journal of Crohn's & colitis. 2018;12(8):883-95.
176. Schoon EJ, Bollani S, Mills PR, Israeli E, Felsenberg D, Ljunghall S, et al. Bone mineral density in relation to efficacy and side effects of budesonide and prednisolone in Crohn's disease. Clinical gastroenterology and hepatology. 2005;3(2):113-21.
177. Schreiber S, Colombel JF, Bloomfield R, Nikolaus S, Sch□lmerich J, Pan□s J, et al. Increased response and remission rates in short-duration Crohn's disease with subcutaneous certolizumab pegol: an analysis of PRECiSE 2 randomized maintenance trial data. American journal of gastroenterology. 2010;105(7):1574-82.
178. Schreiber S, Howaldt S, Raedler A. Oral 4-aminosalicylic acid versus 5-aminosalicylic acid slow release tablets. Double blind, controlled pilot study in the maintenance treatment of Crohn's ileocolitis. Gut. 1994;35(8):1081-5.
179. Schreiber S, Khaliq-Kareemi M, Lawrance IC, Thomsen O, Hanauer SB, McColm J, et al. Maintenance therapy with certolizumab pegol for Crohn's disease. New England journal of medicine. 2007;357(3):239-50.
180. Schreiber S, Lawrance IC, Thomsen O, Hanauer SB, Bloomfield R, Sandborn WJ. Randomised clinical trial: certolizumab pegol for fistulas in Crohn's disease - subgroup results from a placebo-controlled study. Alimentary pharmacology & therapeutics. 2011;33(2):185-93.
181. Schreiber S, Reinisch W, Colombel JF, Sandborn WJ, Hommes DW, Robinson AM, et al. Subgroup analysis of the placebo-controlled CHARM trial: increased remission rates through 3 years for adalimumab-treated patients with early Crohn's disease. Journal of Crohn's & colitis. 2013;7(3):213-21.
182. Schreiber S, Rutgeerts P, Fedorak RN, Khaliq-Kareemi M, Kamm MA, Boivin M, et al. A randomized, placebo-controlled trial of certolizumab pegol (CDP870) for treatment of Crohn's disease. Gastroenterology. 2005;129(3):807-18.
183. Schulberg JD, Wright EK, Holt BA, Hamilton AL, Sutherland TR, Ross AL, et al. Intensive drug therapy versus standard drug therapy for symptomatic intestinal Crohn's disease strictures (STRIDENT): an open-label, single-centre, randomised controlled trial. The lancet. Gastroenterology & hepatology. 2022;7(4):318-31.
184. Schwartz D. Can we reset the immunostat? A randomized, double-blind, controlled withdrawal trial in Crohn's disease patients win long-term remission taking azathioprine. Evidence-based gastroenterology. 2005;6(4):114-5.
185. Schwartz DA, Peyrin-Biroulet L, Lasch K, Adsul S, Danese S. Efficacy and Safety of 2 Vedolizumab Intravenous Regimens for Perianal Fistulizing Crohn's Disease: ENTERPRISE Study. Clinical gastroenterology and hepatology. 2022;20(5):1059-67.e9.
186. Shao LM, Chen MY, Cai JT. Meta-analysis: the efficacy and safety of certolizumab pegol in Crohn's disease. Alimentary pharmacology & therapeutics. 2009;29(6):605-14.
187. Singh S, Stitt LW, Zou G, Khanna R, Dulai PS, Sandborn WJ, et al. Early combined immunosuppression may be effective and safe in older patients with Crohn's disease: post hoc analysis of REACT. Alimentary pharmacology & therapeutics. 2019;49(9):1188-94.

188. Singleton JW, Hanauer S, Robinson M**.** Quality-of-life results of double-blind, placebo-controlled trial of mesalamine in patients with Crohn's disease. Digestive diseases and sciences. 1995;40(5):931-5.
189. Singleton JW, Hanauer SB, Gitnick GL, Peppercorn MA, Robinson MG, Wruble LD, et al. Mesalamine capsules for the treatment of active Crohn's disease: results of a 16-week trial. Pentasa Crohn's Disease Study Group. Gastroenterology. 1993;104(5):1293-301.
190. Sluiter RL, Van Marrewijk C, De Jong D, Scheffer H, Guchelaar HJ, Derijks L, et al. Genotype-Guided Thiopurine Dosing Does not Lead to Additional Costs in Patients With Inflammatory Bowel Disease. Journal of Crohn's & colitis. 2019;13(7):838-45.
191. Smedh K, Olaison G, Teder K, Sj□dahl R**.** Low-dose prednisolone as prophylaxis for endoscopic ileal recurrence after resection in patients with Crohn's disease. European journal of gastroenterology & hepatology. 1995;7(5):485-6.
192. Smith RC, Rhodes J, Heatley RV, Hughes LE, Crosby DL, Rees BI, et al. Low dose steroids and clinical relapse in Crohn's disease: a controlled trial. Gut. 1978;19(7):606-10.
193. Steenholdt C, Bendtzen K, Brynskov J, Thomsen O, Munck LK, Christensen LA, et al. Changes in serum trough levels of infliximab during treatment intensification but not in anti-infliximab antibody detection are associated with clinical outcomes after therapeutic failure in Crohn's disease. Journal of Crohn's & colitis. 2015;9(3):238-45.
194. Stricker T, Braegger CP**.** Antibody to tumor necrosis factor in the treatment of Crohn's disease. Journal of pediatric gastroenterology and nutrition. 1998;27(3):369-70.
195. Strik AS, L□wenberg M, Mould DR, Berends SE, Ponsioen CI, van den Brande JMH, et al. Efficacy of dashboard driven dosing of infliximab in inflammatory bowel disease patients; a randomized controlled trial. Scandinavian journal of gastroenterology. 2021;56(2):145-54.
196. Strik AS, van de Vrie W, Bloemsaat-Minekus JPJ, Nurmohamed M, Bossuyt PJJ, Bodelier A, et al. Serum concentrations after switching from originator infliximab to the biosimilar CT-P13 in patients with quiescent inflammatory bowel disease (SECURE): an open-label, multicentre, phase 4 non-inferiority trial. The lancet gastroenterology and hepatology. 2018;3(6):404-12.
197. Su H, Kang Q, Wang H, Yin H, Duan L, Liu Y, et al. Effects of glucocorticoids combined with probiotics in treating crohn's disease on inflammatory factors and intestinal microflora. Experimental and therapeutic medicine. 2018;16(4):2999-3003.
198. Syversen SW, Goll GL, J□rgensen KK, Sandanger, Sexton J, Olsen IC, et al. Effect of Therapeutic Drug Monitoring vs Standard Therapy During Infliximab Induction on Disease Remission in Patients With Chronic Immune-Mediated Inflammatory Diseases: a Randomized Clinical Trial. JAMA. 2021;325(17):1744-54.
199. Taxonera C, L□pez-Sanrom□n A, Vera-Mendoza I, Dom□nech E, Ruiz VV, Mar□n-Jim□nez I, et al. Quality of life during one year of postoperative prophylactic drug therapy after intestinal resection in Crohn's patients: results of the APPRECIA trial. Digestive and liver disease. 2019;51(4):529-35.
200. Thomsen OO, Cortot A, Jewell D, Wright JP, Winter T, Veloso FT, et al. Budesonide and mesalazine in active Crohn's disease: a comparison of the effects on quality of life. American journal of gastroenterology. 2002;97(3):649-53.
201. Tillinger W, Gasche C, Reinisch W, Lichtenberger C, Bakos S, Dejaco C, et al. Influence of topically and systemically active steroids on circulating leukocytes in Crohn's disease. American journal of gastroenterology. 1998;93(10):1848-53.

202. Toedter GP, Blank M, Lang Y, Chen D, Sandborn WJ, de Villiers WJ. Relationship of C-reactive protein with clinical response after therapy with ustekinumab in Crohn's disease. American journal of gastroenterology. 2009;104(11):2768-73.
203. Treton X, Bouhnik Y, Mary JY, Colombel JF, Duclos B, Soule JC, et al. Azathioprine withdrawal in patients with Crohn's disease maintained on prolonged remission: a high risk of relapse. Clinical gastroenterology and hepatology. 2009;7(1):80-5.
204. Tursi A, Elisei W, Picchio M, Zampaletta C, Pelecca G, Faggiani R, et al. Comparison of the effectiveness of infliximab and adalimumab in preventing postoperative recurrence in patients with Crohn's disease: an open-label, pilot study. Techniques in coloproctology. 2014;18(11):1041-6.
205. Van Assche G, Magdelaine-Beuzelin C, D'Haens G, Baert F, Noman M, Vermeire S, et al. Withdrawal of immunosuppression in Crohn's disease treated with scheduled infliximab maintenance: a randomized trial. Gastroenterology. 2008;134(7):1861-8.
206. Van Hees PAM, Van Lier HJJ, Van Elteren PH, Driessen WMM, Van Hogezand RA, Ten Velde GPM, et al. Effect of sulphasalazine in patients with active Crohn's disease: a controlled double-blind study. Gut. 1981;22(5):404-9.
207. Vaysse T, Bourrier A, Amiot A, Grimaud JC, Peyrin-Biroulet L, Zallot C, et al. Effectiveness and Safety of Vedolizumab Induction Therapy for Patients With Inflammatory Bowel Disease. Clinical gastroenterology and hepatology. 2016;14(11):1593-601.e2.
208. Vermeire S, Loftus EV, Colombel JF, Feagan BG, Sandborn WJ, Sands BE, et al. Long-term Efficacy of Vedolizumab for Crohn's Disease. Journal of Crohn's & colitis. 2017;11(4):412-24.
209. Vilien M, Dahlerup JF, Munck LK, N□rregaard P, Gr□nbaek K, Fallingborg J. Randomized controlled azathioprine withdrawal after more than two years treatment in Crohn's disease: increased relapse rate the following year. Alimentary pharmacology & therapeutics. 2004;19(11):1147-52.
210. Watanabe K, Matsumoto T, Hisamatsu T, Nakase H, Motoya S, Yoshimura N, et al. Clinical and Pharmacokinetic Factors Associated With Adalimumab-Induced Mucosal Healing in Patients With Crohn's Disease. Clinical gastroenterology and hepatology. 2018;16(4):542-9.e1.
211. Watanabe M, Hibi T, Mostafa NM, Chao J, Arora V, Camez A, et al. Long-term safety and efficacy of adalimumab in Japanese patients with moderate to severe Crohn's disease. Journal of Crohn's & colitis. 2014;8(11):1407-16.
212. Willoughby JM, Beckett J, Kumar PJ, Dawson AM. Controlled trial of azathioprine in Crohn's disease. Lancet (london, england). 1971;2(7731):944-7.
213. Winter TA, Wright J, Ghosh S, Jahnsen J, Innes A, Round P. Intravenous CDP870, a PEGylated Fab' fragment of a humanized antitumour necrosis factor antibody, in patients with moderate-to-severe Crohn's disease: an exploratory study. Alimentary pharmacology & therapeutics. 2004;20(11-12):1337-46.
214. Wong ECL, Dulai PS, Marshall JK, Jairath V, Reinisch W, Narula N. Predictors of Clinical Remission to Placebo in Clinical Trials of Crohn's Disease. Inflammatory bowel diseases. 2022.
215. Wong ECL, Marshall JK, Reinisch W, Narula N. Body Mass Index Does Not Impact Clinical Efficacy of Ustekinumab in Crohn's Disease: a Post Hoc Analysis of the IM-UNITI Trial. Inflammatory bowel diseases. 2021;27(6):848-54.
216. Yajnik V, Khan N, Dubinsky M, Axler J, James A, Abhyankar B, et al. Efficacy and Safety of Vedolizumab in Ulcerative Colitis and Crohn's Disease Patients Stratified by Age. Advances in therapy. 2017;34(2):542-59.

217. Yoshida K, Fukunaga K, Ikeuchi H, Kamikozuru K, Hida N, Ohda Y, et al. Scheduled infliximab monotherapy to prevent recurrence of Crohn's disease following ileocolic or ileal resection: a 3-year prospective randomized open trial. Inflammatory bowel diseases. 2012;18(9):1617-23.

5) Characteristics of studies included in systematic review

Reference<br><br>Acronym	Trial registration number<br><br>Study phase	Countries<br><br>Number of study sites<br><br>Study dates	Study type*	Population	Sample size	Intervention(s): start dosage and -frequency	Compara-tor(s)	Outcomes	Follow-up duration	Overall RoB
Arber 1995 (1)	NR<br><br>NR	Israel<br><br>9 study sites<br><br>NR	Maintenance study	Patients with Crohn's disease for at least 1 year, diagnosed by accepted clinical, radiological and endoscopic criteria<br><br>HBI < 4<br><br>Biological-naïve	59	Mesalazine 500 mg two times per day	Placebo	Maintenance of clinical remission<br>Steroid-free remission (maintenance)<br>Discontinuations<br>Discontinuations due to adverse events	52 weeks	Some concerns
Ardizzone 2003 (2)	NR<br><br>NR	Italy<br><br>1 study site<br><br>Screening: January 1997 to March 1998	Induction study	Patients (18-75 years) with chronic active Crohn's disease, confirmed by routine clinical, radiographic, endoscopic and pathologic criteria<br><br>CDAI ≥ 200<br><br>Biological-naïve	54	Methotrexate 25 mg once per week<br><br>Azathioprine 2 mg/kg once per day	-	Induction of clinical remission<br>Steroid-free remission (induction)	26 weeks	Some concerns
Ardizzone 2004 (3)	NR<br><br>NR	Italy<br><br>1 study site<br><br>March 1994 to August 2001	Maintenance study	Patients with Crohn's disease, confirmed by routine clinical, radiographic, endoscopic and pathologic criteria, who underwent surgery for symptomatic intestinal stenoses or occlusion<br><br>Previous treatment with biologicals unclear	142	Mesalamine 1000 mg three times per day<br><br>Azathioprine 2 mg/kg once per day	-	Maintenance of clinical remission<br>Steroid-free remission (maintenance)<br>Discontinuations<br>Discontinuations due to adverse events	104 weeks	High

Reference<br><br>Acronym	Trial registration number<br><br>Study phase	Countries<br><br>Number of study sites<br><br>Study dates	Study type*	Population	Sample size	Intervention(s): start dosage and -frequency	Comparator(s)	Outcomes	Follow-up duration	Overall RoB
Bar-Meir 1998 (4)	NR<br><br>NR	Israel<br><br>14 study sites<br><br>NR	Induction study	Patients (18-70 years) with a current exacerbation of previously diagnosed Crohn's disease or ^{3} 3 months of active disease in a new diagnosis<br><br>CDAI 150-350<br><br>Biological-naïve	201	Budesonide 3 mg three times per day<br><br>Prednisone 30 mg once per day	-	Induction of clinical remission and response combined<br>Steroid-free remission/response (induction)	8 weeks	Some concerns
Brignola 1995 (5)	NR<br><br>NR	Italy<br><br>8 study sites<br><br>Recruitment: June 1990 to December 1991	Maintenance study	Patients with Crohn's disease who have undergone curative resection<br><br>Biological-naïve	87	Mesalamine 1000 mg three times per day	Placebo	Maintenance of clinical remission and response combined<br>Discontinuations<br>Discontinuations due to adverse events	52 weeks	Some concerns
Campieri 1997 (6)	NR<br><br>NR	Australia, Belgium, Germany, Ireland, Italy, Netherlands, New Zealand, Sweden, UK<br><br>26 study sites<br><br>NR	Induction study	Patients (at least 18 years) with a confirmed diagnosis of active Crohn's disease<br><br>CDAI $\geq$ 200<br><br>Biological-naïve	178	Budesonide 9 mg once per day<br><br>Budesonide 4.5 mg two times per day<br><br>Prednisolone 40 mg once per day	-	Induction of clinical remission	12 weeks	Some concerns
Candy 1995 (7)	NR<br><br>NR	South Africa<br><br>1 study site<br><br>NR	Induction and maintenance study<br><br>Treat-through	Patients (15-65 years) with confirmed (by radiology or endoscopy) active Crohn's disease	63	Induction: Azathioprine 50 mg once per day + Prednisolone 1 mg/kg once per day	-	Induction of clinical remission	12 weeks	Some concerns

Reference<br><br>Acronym	Trial registration number<br><br>Study phase	Countries<br><br>Number of study sites<br><br>Study dates	Study type*	Population	Sample size	Intervention(s): start dosage and -frequency	Compara-tor(s)	Outcomes	Follow-up duration	Overall RoB
				CDAI ≥ 200<br><br>Biological-naïve		Prednisolone 1 mg/kg once per day				
						Maintenance: Azathioprine 50 mg once per day	Placebo	Maintenance of clinical remission<br>Steroid-free remission (maintenance)<br>Discontinuations<br>Discontinuations due to adverse events	53 weeks	
Caprilli 2003 (8)	NR<br><br>NR	Italy<br><br>17 study sites<br><br>January 1997 to June 2000	Maintenance study	Patients (18-65 years) operated on for Crohn's disease (first or second resection); diagnosis confirmed macroscopically or microscopically by standard criteria<br><br>Biological-naïve	206	Mesalazine 1333 mg three times per day<br><br>Mesalazine 800 mg three times per day	-	Maintenance of clinical remission and response combined<br>Steroid-free remission/response (maintenance)<br>Discontinuations<br>Discontinuations due to adverse events	52 weeks	High
Chen 2020 (9)	NCT02499783<br><br>Phase 3	China<br><br>15 study sites<br><br>August 2015 to December 2017	Induction study	Patients (18-70 years) with moderately-to-severely active Crohn's disease who did not improve with conventional therapy of oral corticosteroids and/or immunosuppressants<br><br>CDAI 220-450<br><br>Biological-naïve	205	Adalimumab 160 mg once per 2 weeks	Placebo	Induction of clinical remission<br>Induction of clinical response	4 weeks	Some concerns

Reference<br><br>Acronym	Trial registration number<br><br>Study phase	Countries<br><br>Number of study sites<br><br>Study dates	Study type*	Population	Sample size	Intervention(s): start dosage and -frequency	Comparator(s)	Outcomes	Follow-up duration	Overall RoB
Colombel 2007 (10)<br><br>CHARM<br><br>*Substudies/post-hoc analyses:*<br>*Colombel 2009 (11)*<br>*Kamm 2011 (12)*<br>*Sandborn 2013a (13)*	NCT00077779<br><br>Phase 3	Worldwide<br><br>92 study sites<br><br>July 2003 to September 2005	Maintenance study	Patients (18-75 years) with known Crohn's disease (radiologic or endoscopic confirmation) for at least 4 months that was moderately-to-severely active<br><br>CDAI 220-450<br><br>Combination of biological-naïve and biological-exposed	778	Adalimumab 40 mg once per 2 weeks<br><br>Adalimumab 40 mg once per week	Placebo	Maintenance of clinical remission<br>Steroid-free remission (maintenance)<br>Discontinuations<br>Discontinuations due to adverse events<br>Serious infections<br>Malignancies<br>Major cardiovascular events (including thrombosis)	52 weeks	High
Colombel 2010 (14)<br><br>SONIC	NCT00094458<br><br>NR	Worldwide (15 countries)<br><br>92 study sites<br><br>March 2005 to November 2008	Induction study	Patients (at least 21 years) with moderate-to-severe Crohn's disease for at least 6 weeks<br><br>CDAI 220-450<br><br>Biological-naïve	508	Infliximab 5 mg/kg once per multiple weeks<br><br>Azathioprine 2.5 mg/kg once per day<br><br>Infliximab 5 mg/kg once per multiple weeks + Azathioprine 2.5 mg/kg once per day	-	Induction of clinical remission<br>Induction of clinical response<br>Steroid-free remission (induction)	50 weeks	Some concerns
Cosnes 2013 (15)<br><br>RAPID	NCT00546546<br><br>NR	France<br><br>24 study sites<br><br>July 2005 to November 2010	Maintenance study	Patients (at least 18 years) diagnosed with Crohn's disease, according to validated criteria, within 6 months before screening<br><br>Biological-naïve	147	Azathioprine 2.5 mg/kg once per day (immediately)<br><br>Azathioprine 2.5 mg/kg once per	-	Maintenance of clinical remission<br>Steroid-free remission (maintenance)<br>Discontinuations<br>Discontinuations due to adverse events	156 weeks	Some concerns

Reference<br><br>Acronym	Trial registration number<br><br>Study phase	Countries<br><br>Number of study sites<br><br>Study dates	Study type*	Population	Sample size	Intervention(s): start dosage and -frequency	Compara-tor(s)	Outcomes	Follow-up duration	Overall RoB
						day (when symptoms start)				
D'Haens 2008 (16)	NCT00554710<br><br>NR	Belgium, Germany, Netherlands<br><br>18 study sites<br><br>May 2001 to January 2004	Induction and maintenance study<br><br>Treat-through	Patients (16-75 years) diagnosed with Crohn's disease within the past 4 years, and currently having active disease<br><br>CDAI ≥ 200<br><br>Biological-naïve	133	Infliximab 5 mg/kg at weeks 0, 2, 6 + Azathioprine 2-2.5 mg/kg once per day	Usual care (methylprednisolone or budesonide)	Induction of clinical remission<br>Maintenance of clinical remission<br>Steroid-free remission (induction)<br>Steroid-free remission (maintenance)<br>Discontinuations<br>Discontinuations due to adverse events<br>Serious infections	104 weeks	Some concerns
D'Haens 2022a (17)<br><br>ADVANCE<br>MOTIVATE	ADVANCE: NCT03105128<br><br>Phase 3	Worldwide (39 countries)<br><br>297 study sites<br><br>May 2017 to August 2020	Induction study	Patients (16-80 years) with a confirmed diagnosis of Crohn's disease for at least 3 months<br><br>CDAI 220-450<br><br>Combination of biological-naïve and biological-exposed	931	Risankizumab 600 mg once per 4 weeks<br><br>Risankizumab 1200 mg once per 4 weeks	Placebo	Induction of clinical remission<br>Induction of clinical reponse	12 weeks	Low
	MOTIVATE: NCT03104413<br><br>Phase 3	Worldwide (40 countries)<br><br>214 study sites<br><br>December 2017 to September 2020	Induction study	Patients (16-80 years) with a confirmed diagnosis of Crohn's disease for at least 3 months, in which previous therapy with biologicals has failed<br><br>CDAI 220-450	618	Risankizumab 600 mg once per 4 weeks<br><br>Risankizumab 1200 mg once per 4 weeks	Placebo	Induction of clinical remission<br>Induction of clinical reponse	12 weeks	

Reference<br><br>Acronym	Trial registration number<br><br>Study phase	Countries<br><br>Number of study sites<br><br>Study dates	Study type*	Population	Sample size	Intervention(s): start dosage and -frequency	Compara-tor(s)	Outcomes	Follow-up duration	Overall RoB
				Biological-exposed						
D'Haens 2022b (18)<br><br>SERENE CD	NCT02065570<br><br>Phase 3	Worldwide (19 countries)<br><br>93 study sites<br><br>May 2014 to January 2020	Induction and maintenance study<br><br>Responder re-randomization	Patients (18-75 years) with moderately-to-severely active Crohn's disease (confirmed by endoscopy) despite adequate treatment with standard therapies<br><br>CDAI 220-450<br><br>Combination of biological-naïve and biological-exposed	Induction: 514	Adalimumab 160 mg once per week (two induction regimens)	-	Induction of clinical remission<br>Induction of clinical response<br>Steroid-free remission (induction)	12 weeks	Low
					Maintenance: 184	Adalimumab 40 mg once per 2 weeks (two treatment strategies)	-	Maintenance of clinical remission<br>Steroid-free remission (maintenance)<br>Discontinuations<br>Discontinuations due to adverse events<br>Serious infections<br>Malignancies<br>Major cardiovascular events (including thrombosis)	44 weeks	
D'Haens 2023 (19)<br><br>DIVERGENCE 1	NCT03046056<br><br>Phase 2	Europe (10 countries), Canada, USA<br><br>39 study sites<br><br>April 2017 to July 2020	Induction and maintenance study<br><br>Treat-through	Patients (18-75 years) with a documented diagnosis of moderately-to-severely active Crohn's disease (imaging, histopathology or ileoscopy) for at least 6 months<br><br>CDAI 200-450<br><br>Combination of biological-naïve and biological-exposed	78	Filgotinib 100 mg once per day<br><br>Filgotinib 200 mg once per day	Placebo	Induction of clinical response<br>Maintenance of clinical remission<br>Discontinuations<br>Discontinuations due to adverse events	24 weeks	Some concerns

Reference<br><br>Acronym	Trial registration number<br><br>Study phase	Countries<br><br>Number of study sites<br><br>Study dates	Study type*	Population	Sample size	Intervention(s): start dosage and -frequency	Compara-tor(s)	Outcomes	Follow-up duration	Overall RoB
De Franchis 1997 (20)	NR<br><br>NR	Italy<br><br>22 study sites<br><br>Enrollment: October 1991 to July 1993	Maintenance study	Patients (18-70 years) with a firmly established diagnosis of Crohn's disease (endoscopic, radiological, surgical confirmation within 1 year prior to enrollment), currently in remission after a flare-up and a standard regimen of steroids<br><br>CDAI 150-450<br><br>Biological-naïve	129	5-aminosalicylic acid 1000 mg three times per day	Placebo	Maintenance of clinical remission<br>Steroid-free remission (maintenance)<br>Discontinuations	40 weeks	Some concerns
De Jong 2007 (21)	NR<br><br>NR	Germany, Netherlands<br><br>33 study sites<br><br>Enrollment: November 1997 to February 2001	Maintenance study	Patients (at least 18 years) with confirmed Crohn's disease, and remission between 3-18 months<br><br>CDAI < 150<br><br>Biological-naïve	160	Budesonide 5 mg once per day<br><br>Budesonide 9 mg once per day	-	Maintenance of clinical remission and response combined<br>Discontinuations<br>Discontinuations due to adverse events	52 weeks	Low
Dignass 2014 (22)	NCT01086553<br><br>Phase 3	Bulgaria, Czech Republic, Germany, Hungary, Latvia, Lithuania, Romania, Russia, Slovakia, Ukraine<br><br>50 study sites	Induction study	Patients (18-75 years) with mild-to-moderately active Corhn's disease for at least 3 months, confirmed by endoscopy and histology or endoscopy and radiology<br><br>CDAI 200-400	473	Budesonide 9 mg once per day<br><br>Budsonide 3 mg three times per day	-	Induction of clinical remission	8 weeks	High

Reference<br><br>Acronym	Trial registration number<br><br>Study phase	Countries<br><br>Number of study sites<br><br>Study dates	Study type*	Population	Sample size	Intervention(s): start dosage and -frequency	Compara-tor(s)	Outcomes	Follow-up duration	Overall RoB
		November 2009 to April 2012		Biological-naïve						
Ewe 1993 (23)	NR<br><br>NR	Germany<br><br>1 study site<br><br>July 1987 to August 1990	Induction study	Patients with active Crohn's disease, based on clinical, endoscopic, radiological, and histological criteria<br><br>CDAI > 150<br><br>Biological-naïve	42	Azathioprine 2.5 mg/kg once per day + Prednisolone 60 mg once per day<br><br>Prednisolone 60 mg once per day	-	Induction of clinical remission and response combined	16 weeks	Some concerns
Ewe 1999 (24)	NR<br><br>NR	Germany<br><br>3 study sites<br><br>July 1992 to April 1994	Maintenance study	Patients with Crohn's disease who have been curatively resected<br><br>Biological-naïve	83	Budesonide 1 mg three times per day	Placebo	Maintenance of clinical remission<br>Discontinuations<br>Discontinuations due to adverse events	52 weeks	Some concerns
Feagan 1995 (25)	NR<br><br>NR	Canada, USA<br><br>7 study sites<br><br>November 1992 to February 1994	Induction study	Patients with chronically active Crohn's disease despite ≥ 3 months Prednisone therapy<br><br>Biological-naïve	141	Methotrexate 25 mg once per week	Placebo	Induction of clinical remission<br>Steroid-free remission (induction)	16 weeks	High
Feagan 2000 (26)	NR<br><br>NR	Canada, Czech Republic, Poland, USA<br><br>7 study sites<br><br>March 1993 to September 1997	Maintenance study<br><br>Responder re-randomization	Patients with chronically active Crohn's disease who entered remission after 16-24 weeks of weekly treatment with 25 mg Methotrexate<br><br>Biological-naïve	76	Methotrexate 15 mg once per week	Placebo	Maintenance of clinical remission<br>Steroid-free remission (maintenance)<br>Discontinuations due to adverse events	40 weeks	Some concerns

Reference<br><br>Acronym	Trial registration number<br><br>Study phase	Countries<br><br>Number of study sites<br><br>Study dates	Study type*	Population	Sample size	Intervention(s): start dosage and -frequency	Compara-tor(s)	Outcomes	Follow-up duration	Overall RoB
Feagan 2016 (27)<br><br>UNITI-1<br>UNITI-2<br>IM-UNITI	UNITI-1: NCT01369329<br><br>Phase 3	Worldwide (23 countries)<br><br>178 study sites<br><br>July 2011 to June 2015	Induction study	Patients (at least 18 years) who had Crohn's disease (with objective evidence) for at least 3 months, and who were primary nonresponders to one or more TNF-antagonists<br><br>CDAI 220-450<br><br>Biological-exposed	741	Ustekinumab 130 mg once<br><br>Ustekinumab 6 mg/kg once	Placebo	Induction of clinical remission<br>Induction of clinical remission and response combined	8 weeks	Low
	UNITI-2: NCT01369342<br><br>Phase 3	Worldwide (23 countries)<br><br>175 study sites<br><br>July 2011 to June 2015	Induction study	Patients (at least 18 years) who had Crohn's disease (with objective evidence) for at least 3 months, and who have had treatment failure or unacceptable side effects when treated with immunosuppressants<br><br>CDAI 220-450<br><br>Combination of biological-naïve and biological-exposed	628	Ustekinumab 130 mg once<br><br>Ustekinumab 6 mg/kg once	Placebo	Induction of clinical remission<br>Induction of clinical remission and response combined	8 weeks	
	IM-UNITI: NCT01369355<br><br>Phase 3	Worldwide (27 countries)<br><br>260 study sites<br><br>July 2011 to June 2015	Maintenance study<br><br>Responder-rerandomization	Patients with Crohn's disease who had a clinical response to Ustekinumab in UNITI-1 or UNITI-2, and are currently in remission<br><br>CDAI < 150	397	Ustekinumab 90 mg once per 12 weeks<br><br>Ustekinumab 90 mg once per 8 weeks	Placebo	Maintenance of clinical remission<br>Steroid-free remission (maintenance)<br>Discontinuations<br>Serious infections	44 weeks	

Reference<br><br>Acronym	Trial registration number<br><br>Study phase	Countries<br><br>Number of study sites<br><br>Study dates	Study type*	Population	Sample size	Intervention(s): start dosage and -frequency	Compara-tor(s)	Outcomes	Follow-up duration	Overall RoB
				Combination of biological-naïve and biological-exposed						
Ferguson 1998 (28)	NR<br><br>NR	Europe (7 countries), Australia<br><br>20 study sites<br><br>NR	Maintenance study<br><br>Responder re-randomization	Patients (18-65 years) in clinical remission of Crohn's disease<br><br>CDAI ≤ 150<br><br>Biological-naïve	75	Budesonide 6 mg once per day<br><br>Budesonide 3 mg once per day	Placebo	Maintenance of clinical remission and response combined<br>Discontinuations<br>Discontinuations due to adverse events	52 weeks	Some concerns
Ferrante 2015 (29)	NCT02247258<br><br>NR	Belgium, Czech Republic, Greece<br><br>4 study sites<br><br>October 2005 to April 2012	Maintenance study	Patients (16-75 years) with Crohn's disease for at least 4 months (diagnosis based on radiology and/or endoscopy) undergoing curative resection and at a high risk of recurrence<br><br>Previous treatment with biologicals unclear	63	Azathioprine 2-2.5 mg/kg once per day<br>(two treatment strategies)	-	Maintenance of clinical remission<br>Steroid-free remission (maintenance)<br>Malignancies	102 weeks	High
Ferrante 2022 (30)<br><br>FORTIFY SS1	NCT03105102<br><br>Phase 3	Worldwide (44 countries)<br><br>273 study sites<br><br>April 2018 to April 2020	Maintenance study<br><br>Responder re-randomization	Patients (16-80 years) with moderately-to-severely active Crohn's disease, with a clinical response to Risankizumab<br><br>CDAI 220-450<br><br>Biological-exposed	542	Risankizumab 180 mg once per 8 weeks<br><br>Risankizumab 360 mg once per 8 weeks	Placebo	Maintenance of clinical remission<br>Discontinuations<br>Discontinuations due to adverse events<br>Serious infections<br>Malignancies<br>Major cardiovascular events (including thrombosis)	52 weeks	Some concerns
Fukushima 2018 (31)	UMIN000002 604<br><br>NR	Japan<br><br>13 study sites	Maintenance study	Patients with Crohn's disease who underwent intestinal resection	43	Infliximab 5 mg/kg once per multiple weeks	No infliximab	Maintenance of clinical remission<br>Steroid-free remission (maintenance)	104 weeks	High

Reference<br><br>Acronym	Trial registration number<br><br>Study phase	Countries<br><br>Number of study sites<br><br>Study dates	Study type*	Population	Sample size	Intervention(s): start dosage and -frequency	Compara-tor(s)	Outcomes	Follow-up duration	Overall RoB
		May 2009 to March 2016		Combination of biological-naïve and biological-exposed				Discontinuations		
Gelbmann 2001 (32)	NR<br><br>NR	Austria, Germany<br><br>NR<br><br>NR	Induction study	Patients (18-70 years) with a confirmed diagnosis (by radiology and/or endoscopy and histology) of Crohn's disease for at least 6 months<br><br>CDAI ≥ 150<br><br>Biological-naïve	93	Budesonide 2 mg three times per day<br><br>Budesonide 3 mg three times per day<br><br>Budesonide 6 mg three times per day	-	Induction of clinical remission	6 weeks	High
Gendre 1993 (33)	NR<br><br>NR	France<br><br>16 study sites<br><br>December 1985 to December 1987	Maintenance study	Patients (at least 15 years) with inactive Crohn's disease for less than 24 months<br><br>CDAI < 150<br><br>Biological-naïve	161	Mesalamine 500 mg four times per day	Placebo	Maintenance of clinical remission and response combined<br>Steroid-free remission/response (maintenance)<br>Discontinuations<br>Discontinuations due to adverse events	104 weeks	Some concerns
Ghosh 2003 (34)	NR<br><br>NR	Belgrium, Czech Republic, Denmark, Israel, Netherlands, Sweden, UK<br><br>September 1999 to August 2000	Induction study	Patients (at least 18 years) with moderate-to-severe Crohn's disease<br><br>CDAI 220-450<br><br>Biological-naïve	248	Natalizumab 3 mg/kg once per 4 weeks<br><br>Natalizumab 6 mg/kg once per 4 weeks	Placebo	Induction of clinical remission<br>Induction of clinical response<br>Steroid-free remission (induction)<br>Steroid-free response (induction)	12 weeks	Some concerns

Reference<br><br>Acronym	Trial registration number<br><br>Study phase	Countries<br><br>Number of study sites<br><br>Study dates	Study type*	Population	Sample size	Intervention(s): start dosage and -frequency	Compara-tor(s)	Outcomes	Follow-up duration	Overall RoB
						Natalizumab 12 mg/kg once per 4 weeks				
Gordon 2001 (35)	NR<br><br>NR	UK<br><br>2 study sites<br><br>NR	Induction study	Patients (at least 18 years) with mild-to-moderately active Crohn's disease, confirmed by 2 or more diagnostic criteria (history, intestinal appearance, histology, fistulae, abscess formation)<br><br>CDAI 151-450<br><br>Biological-naïve	30	Natalizumab 3 mg/kg once	Placebo	Induction of clinical remission	12 weeks	Some concerns
Green 2001 (36)	NR<br><br>NR	Ireland, UK<br><br>37 study sites<br><br>May 1996 to May 1999	Maintenance study	Patients (18-80 years) in remission from Crohn's disease for at least 1 month, and who experienced at least one episode of active disease in the past year<br><br>Biological-naïve	143	Budesonide 3-9 mg once per day<br><br>Budesonide 6 mg once per day	-	Maintenance of clinical remission<br>Discontinuations<br>Discontinuations due to adverse events	52 weeks	Some concerns
Greenberg 1994 (37)	NR<br><br>NR	Canada<br><br>27 study sites<br><br>October 1991 to December 1992	Induction study	Patients (at least 18 years) with a confirmed diagnosis of active Crohn's disease for at least 2 years<br><br>CDAI $\geq$ 200<br><br>Biological-naïve	258	Budesonide 1.5 mg two times per day<br><br>Budesonide 4.5 mg two times per day	Placebo	Induction of clinical remission<br>Steroid-free remission (induction)	8 weeks	High

Reference<br><br>Acronym	Trial registration number<br><br>Study phase	Countries<br><br>Number of study sites<br><br>Study dates	Study type*	Population	Sample size	Intervention(s): start dosage and -frequency	Compara-tor(s)	Outcomes	Follow-up duration	Overall RoB
						Budesonide 7.5 mg two times per day				
Gross 1996 (38)	NR<br><br>NR	Austria, Germany<br><br>NR<br><br>NR	Induction study	Patients (18-70 years) with active Crohn's disease, who had an established diagnosis of at least 3 months<br><br>CDAI 151-350<br><br>Biological-naïve	69	Budesonide 3 mg three times per day<br><br>6-methylprednisolone 48 mg once per day	-	Induction of clinical remission and response combined	8 weeks	Some concerns
Gross 1998 (39)	NR<br><br>NR	Germany<br><br>NR<br><br>NR	Maintenance study<br><br>Responder re-randomization	Patients (18-70 years) with Crohn's disease in steroid induced remission<br><br>Biological-naïve	192	Budesonide 1 mg three times per day	Placebo	Maintenance of clinical remission<br>Discontinuations<br>Discontinuations due to adverse events	52 weeks	Some concerns
Hanauer 2002 (40)<br><br>ACCENT I<br><br>*Substudies/post-hoc analyses: Rutgeerts 2004* (41)	NCT00207662<br><br>Phase 3	Europe, Israel, North America<br><br>55 study sites<br><br>February 1999 to August 2000	Maintenance study	Patients with Crohn's disease for at least 3 months<br><br>CDAI 220-400<br><br>Biological-naïve	335	Infliximab 5 mg/kg once per 4 weeks (two treatment strategies)	Placebo	Maintenance of clinical remission<br>Discontinuations<br>Discontinuations due to adverse events<br>Serious infections<br>Malignancies	52 weeks	Some concerns
Hanauer 2004 (42)	NR<br><br>NR	Belgium, USA<br><br>5 study sites<br><br>1992 to 1996	Maintenance study	Patients with Crohn's disease who underwent ileocolonic resection<br><br>Biological-naïve	131	6-mercaptopurine 50 mg once per day	Placebo	Discontinuations<br>Discontinuations due to adverse events	104 weeks	Some concerns

Reference<br><br>Acronym	Trial registration number<br><br>Study phase	Countries<br><br>Number of study sites<br><br>Study dates	Study type*	Population	Sample size	Intervention(s): start dosage and -frequency	Compara-tor(s)	Outcomes	Follow-up duration	Overall RoB
						Mesalamine 1000 mg three times per day				
Hanauer 2005 (43)	NR<br><br>NR	USA<br><br>22 study sites<br><br>NR	Maintenance study<br><br>Responder re-randomization	Patients (at least 18 years) with a confirmed diagnosis of Crohn's disease who had previously achieved remission<br><br>CDAI < 150<br><br>Biological-naïve	110	Budesonide 6 mg once per day	Placebo	Maintenance of clinical remission and response combined<br>Discontinuations<br>Discontinuations due to adverse events	52 weeks	Some concerns
Hanauer 2006 (44)<br><br>CLASSIC-I	NR<br><br>NR	Belgium, Canada, Czech Republic, Netherlands, Poland, USA<br><br>55 study sites<br><br>July 2002 to December 2003	Induction study	Patients (18-75 years) with moderate-to-severe Crohn's disease for at least 4 months, confirmed by radiologic or endoscopic studies<br><br>CDAI 220-450<br><br>Biological-naïve	299	Adalimumab 40 mg once per 2 weeks<br><br>Adalimumab 80 mg once per 2 weeks<br><br>Adalimumab 160 mg once per 2 weeks	Placebo	Induction of clinical remission<br>Induction of clinical response	4 weeks	Some concerns
Hanauer 2021 (45)<br><br>VOLTAIRE-CD	NCT02871635<br><br>Phase 3	Europe (12 countries), USA<br><br>92 study sites<br><br>January 2017 to May 2019	Induction study	Patients (18-80 years) with moderately-to-severely active Crohn's disease for at least 4 months, confirmed with endoscopic or radiologic evidence<br><br>CDAI 220-450	147	BI 695501 160 mg once per 2 weeks<br><br>Adalimumab 160 mg once per 2 weeks	-	Induction of clinical remission<br>Induction of clinical response	48 weeks	Low

Reference<br><br>Acronym	Trial registration number<br><br>Study phase	Countries<br><br>Number of study sites<br><br>Study dates	Study type*	Population	Sample size	Intervention(s): start dosage and -frequency	Compara-tor(s)	Outcomes	Follow-up duration	Overall RoB
				Combination of biological-naïve and biological-exposed						
Hellers 1999 (46)	NR<br><br>NR	Belgium, Denmark, France, Germany, Italy, Netherlands, Sweden, UK<br><br>13 study sites<br><br>February 1992 to August 1993	Maintenance study	Patients with Crohn's disease who had resectional surgery; diagnosis was verified at surgery by inspection and biopsy<br><br>Biological-naïve	130	Budesonide 6 mg once per day	Placebo	Maintenance of clinical remission<br>Steroid-free remission (maintenance)<br>Discontinuations<br>Discontinuations due to adverse events	52 weeks	Some concerns
Hisamatsu 2019 (47)<br><br>DIAMOND2	UMIN000009 596<br><br>NR	Japan<br><br>48 study sites<br><br>Recruitment: January 2013 to December 2016	Maintenance study<br><br>Responder re-randomization	Patients (15-65 years) with Crohn's disease who were in corticosteroid-free remission with use of thiopurines and Adalimumab<br><br>CDAI < 150<br><br>Biological-exposed	52	Adalimumab 40 mg once per 2 weeks + Azathioprine 25-50 mg once per day or 6-mercaptopurine 30 mg once per day<br><br>Adalimumab 40 mg once per 2 weeks	-	Maintenance of clinical remission and response combined<br>Steroid-free remission/response (maintenance)<br>Discontinuations<br>Discontinuations due to adverse events	52 weeks	Some concerns
Hueber 2012 (48)	NCT01009281<br><br>Phase 2	Austria, Canada, Germany, Poland, USA<br><br>25 study sites	Induction study	Patients (18-75 years) with moderate-to-severe Crohn's disease for at least 3 months, with objective evidence of inflammation at baseline	59	Secukinumab 10 mg/kg once per 3 weeks	Placebo	Induction of clinical remission<br>Induction of clinical response	18 weeks	Low

Reference<br><br>Acronym	Trial registration number<br><br>Study phase	Countries<br><br>Number of study sites<br><br>Study dates	Study type*	Population	Sample size	Intervention(s): start dosage and -frequency	Compara-tor(s)	Outcomes	Follow-up duration	Overall RoB
		August 2008 to May 2010		CDAI 220-450<br><br>Combination of biological-naïve and biological-exposed						
International Mesalazine Study Group 1990 (49)	NR<br><br>NR	Belgium, Canada, France, Italy, South Africa, Spain, Sweden, UK<br><br>NR<br><br>NR	Maintenance study	Patients with inactive Crohn's disease, with a diagnosis confirmed by X-ray, biopsy and endoscopy<br><br>CDAI < 150<br><br>Biological-naïve	248	5-aminosalicylic acid 500 mg three times per day	Placebo	Maintenance of clinical remission and response combined<br>Steroid-free remission/response (maintenance)<br>Discontinuations<br>Discontinuations due to adverse events	52 weeks	High
Jørgensen 2017 (50)<br><br>NOR-SWITCH<br><br>*Substudies/post-hoc analyses: Jørgensen 2020* (51)	NCT02148640<br><br>Phase 4	Norway<br><br>25 study sites<br><br>October 2014 to July 2015	Maintenance study	Patients (at least 18 years) with a clinical diagnosis of Crohn's disease on stable treatment with infliximab originator for at least 6 months<br><br>Biological-exposed	155	Infliximab<br><br>CT-P13	-	Maintenance of clinical remission<br>Discontinuations<br>Discontinuations due to adverse events	52 weeks	Low
Lémann 2005 (52)	NR<br><br>NR	Belgium, France<br><br>12 study sites<br><br>October 1995 to November 1999	Maintenance study	Patients (at least 18 years) with Crohn's disease (according to established clinical, endoscopic, radiological, and histological criteria) in remission after at least 42 months of treatment with Azathioprine	83	Azathioprine 1.7 mg/kg once per day	Placebo	Maintenance of clinical remission<br>Discontinuations<br>Discontinuations due to adverse events	78 weeks	Some concerns

Reference<br><br>Acronym	Trial registration number<br><br>Study phase	Countries<br><br>Number of study sites<br><br>Study dates	Study type*	Population	Sample size	Intervention(s): start dosage and -frequency	Compara-tor(s)	Outcomes	Follow-up duration	Overall RoB
				CDAI ≤ 150<br><br>Biological-naïve						
Lémann 2006 (53)	NR<br><br>NR	France<br><br>22 study sites<br>June 2000 to May 2002	Induction study	Patients (at least 18 years) with active steroid-dependent Crohn's disease (according to established clinical, endoscopic, radiologic, and histologic criteria)<br><br>Biological-naïve	56	Infliximab 5 mg/kg at weeks 0, 2, 6 + Azathioprine 2-3 mg/kg once per day or 6-mercaptopurine 1-1.5 mg/kg once per day<br><br>Azathioprine 2-3 mg/kg once per day or 6-mercaptopurine 1-1.5 mg/kg once per day	-	Induction of clinical remission<br>Steroid-free remission (induction)	52 weeks	Some concerns
Lennard-Jones 1977 (54)	NR<br><br>NR	UK<br><br>9 study sites<br><br>1970 to 1975	Maintenance study	Patients with Crohn's disease treated with resection or who were asymptomatic; diagnosis confirmed by examination of the operation specimen, with radiology or at laparotomy<br><br>Biological-naïve	43	Sulphasalazine 1000 mg three times per day	Placebo	Discontinuations due to adverse events	52 weeks	Some concerns
Lochs 2000 (55)	NR<br><br>NR	Austria, Denmark, Germany, Norway,	Maintenance study	Patients (18-70 years) who underwent surgical resection for Crohn's disease (confirmed by	324	Mesalamine 1333 mg three times per day	Placebo	Maintenance of clinical remission<br>Steroid-free remission (maintenance)	78 weeks	Some concerns

Reference<br><br>Acronym	Trial registration number<br><br>Study phase	Countries<br><br>Number of study sites<br><br>Study dates	Study type*	Population	Sample size	Intervention(s): start dosage and -frequency	Compara-tor(s)	Outcomes	Follow-up duration	Overall RoB
		Sweden, Switzerland<br><br>29 study sites<br><br>July 1992 to June 1996		endoscopic, histological, and/or radiological criteria)<br><br>Biological-naïve				Discontinuations		
Löfberg 1996 (56)	NR<br><br>NR	Belgium, Denmark, Germany, Netherlands, Sweden, UK<br><br>11 study sites<br><br>NR	Maintenance study	Patients (at least 18 years) with an established diagnosis of Crohn's disease who achieved remission<br><br>CDAI ≤ 150<br><br>Biological-naïve	90	Budesonide 6 mg once per day<br><br>Budesonide 3 mg once per day	Placebo	Maintenance of clinical remission and response combined<br>Discontinuations<br>Discontinuations due to adverse events	52 weeks	Some concerns
Loftus 2023 (57)<br><br>U-EXCEED<br>U-EXCEL<br>U-ENDURE	U-EXCEED: NCT03345836<br><br>Phase 3	Worldwide (43 countries)<br><br>277 study sites<br><br>November 2017 to August 2021	Induction study	Patients (18-75 years) with a confirmed diagnosis of moderate-to-severe Crohn's disease (confirmed by biopsy results) for at least 3 months, who previously failed one or more biologic therapies<br><br>Biological-exposed	495	Upadacitinib 45 mg once per day	Placebo	Induction of clinical remission<br>Induction of clinical response<br>Steroid-free remission (induction)	12 weeks	Low
	U-EXCEL: NCT03345849<br><br>Phase 3	Worldwide (43 countries)<br><br>277 study sites<br><br>December 2017 to January 2022	Induction study	Patients (18-75 years) with a confirmed diagnosis of moderate-to-severe Crohn's disease (confirmed by biopsy results) for at least 3 months, who previously	526	Upadacitinib 45 mg once per day	Placebo	Induction of clinical remission<br>Induction of clinical response<br>Steroid-free remission (induction)	12 weeks	

Reference<br><br>Acronym	Trial registration number<br><br>Study phase	Countries<br><br>Number of study sites<br><br>Study dates	Study type*	Population	Sample size	Intervention(s): start dosage and -frequency	Compara-tor(s)	Outcomes	Follow-up duration	Overall RoB
				failed one or more conventional or biologic therapies<br><br>Combination biological-exposed and -naïve						
	U-ENDURE: NCT03345823<br><br>Phase 3	Worldwide (43 countries)<br><br>277 study sites<br><br>Started March 2018	Maintenance study<br><br>Responder re-randomization	Patients (18-75 years) with a confirmed diagnosis of moderate-to-severe Crohn's disease (confirmed by biopsy results) for at least 3 months and who participated in U-EXCEED or U-EXCEL<br><br>Combination biological-exposed and -naïve	502	Upadacitinib 15 mg once per day<br><br>Upadacitinib 30 mg once per day	Placebo	Maintenance of clinical remission<br>Steroid-free remission (maintenance)<br>Discontinuations<br>Discontinuations due to adverse events<br>Serious infections<br>Malignancies<br>Major cardiovascular events (including thrombosis)	52 weeks	
López-Sanromán 2017 (58)<br><br>APPRECIA	NCT01564823<br><br>Phase 3	Spain<br><br>22 study sites<br><br>January 2012 to January 2015	Maintenance study	Patients (18-70 years) with a confirmed diagnosis of Crohn's disease who were a candidate for resection<br><br>Combination of biological-naïve and biological-exposed	85	Azathioprine 2.5 mg/kg once per day<br><br>Adalimumab 160 mg once per 2 weeks	-	Maintenance of clinical remission<br>Discontinuations<br>Discontinuations due to adverse events	52 weeks	Some concerns
Mahida 1990 (59)	NR<br><br>NR	NR<br><br>NR<br><br>NR	Induction study	Patients (at least 18 years) with active Crohn's disease, as indicated by symptoms, physical examination and inflammatory indicators<br><br>Biological-naïve	40	5-aminosalicylic acid 1000 mg three times per day	Placebo	Induction of clinical response<br>Steroid-free response (induction)	6 weeks	Some concerns

Reference<br><br>Acronym	Trial registration number<br><br>Study phase	Countries<br><br>Number of study sites<br><br>Study dates	Study type*	Population	Sample size	Intervention(s): start dosage and -frequency	Compara-tor(s)	Outcomes	Follow-up duration	Overall RoB
Mantzaris 2003 (60)	NR<br><br>NR	Greece<br><br>1 study site<br><br>January 1994 to December 1998	Maintenance study	Patients (18-65 years) with inactive, inflammatory, steroid-dependent Crohn's disease, confirmed by gastroscopy, ileocolonoscopy, biopsy, small bowel enema and/or CT-scan<br><br>Biological-naïve	57	Budesonide 6 mg once per day<br><br>Mesalamine 1000 mg three times per day	-	Maintenance of clinical remission and response combined<br>Discontinuations due to adverse events	52 weeks	Some concerns
Mantzaris 2009 (61)	NR<br><br>NR	Greece<br><br>NR<br><br>January 1998 to November 2001	Maintenance study	Patients (18-67 years) with inflammatory steroid-dependent Crohn's disease in remission, confirmed by ileocolonoscopy, gastroscopy, and small bowel enema<br><br>CDAI < 150<br><br>Biological-naïve	77	Azathioprine 2-2.5 mg/kg once per day<br><br>Budesonide 6-9 mg once per day	-	Maintenance of clinical remission and response combined<br>Discontinuations<br>Discontinuations due to adverse events	52 weeks	Some concerns
Martin 1990 (62)	NR<br><br>NR	Canada<br><br>8 study sites<br><br>NR	Induction study	Patients (at least 18 years) with active, uncomplicated Crohn's disease, confirmed by barium enema and/or colonoscopy and biopsy<br><br>CDAI 200-450<br><br>Biological-naïve	50	5-aminosalicylic acid 1000 mg three times per day<br><br>Prednisone 40 mg once per day	-	Induction of clinical remission	12 weeks	Some concerns

Reference<br><br>Acronym	Trial registration number<br><br>Study phase	Countries<br><br>Number of study sites<br><br>Study dates	Study type*	Population	Sample size	Intervention(s): start dosage and -frequency	Compara-tor(s)	Outcomes	Follow-up duration	Overall RoB
Maté-Jiménez 2000 (63)	NR<br><br>NR	Spain<br><br>1 study site<br><br>November 1994 to May 1997	Induction and maintenance study<br><br>Treat-through	Patients (15-70 years) with Crohn's disease (radiological or endoscopic diagnosis) who were steroid-dependent<br><br>Biological-naïve	38	6-mercaptopurine 1.5 mg/kg once per day<br><br>Methotrexate 15 mg once per week<br><br>5-aminosalicylic acid 3000 mg once per day	-	Induction of clinical remission<br>Maintenance of clinical remission<br>Steroid-free remission (induction)<br>Steorid-free remission (maintenance)	106 weeks	High
Matsumoto 2016 (64)<br><br>DIAMOND	UMIN000005146<br><br>NR	Japan<br><br>73 study sites<br><br>Recruitment: June 2011 to June 2014	Induction study	Patients (15-65 years) with moderate-to-severe Crohn's disease<br><br>CDAI ≥ 220<br><br>Biological-naïve	177	Adalimumab 160 mg once per 2 weeks<br><br>Adalimumab 160 mg once per 2 weeks + Azathioprine 25-50 mg once per day	-	Induction of clinical remission	52 weeks	Some concerns
McLeod 1995 (65)	NR<br><br>NR	Canada, USA<br><br>7 study sites<br><br>November 1986 to May 1993	Maintenance study	Patients who had undergone a surgical resection for Crohn's disease, confirmed by pathology<br><br>Biological-naïve	177	Mesalamine 1500 mg two times per day	Placebo	Discontinuations	312 weeks	Some concerns
Modigliani 1996 (66)	NR<br><br>NR	Belgium, France<br><br>20 study sites	Maintenance study	Patients (at least 15 years) with active Crohn's disease<br><br>CDAI > 200	134	Mesalamine 2000 mg two times per day	Placebo	Maintenance of clinical remission and response combined	52 weeks	Some concerns

Reference<br><br>Acronym	Trial registration number<br><br>Study phase	Countries<br><br>Number of study sites<br><br>Study dates	Study type*	Population	Sample size	Intervention(s): start dosage and -frequency	Compara-tor(s)	Outcomes	Follow-up duration	Overall RoB
		February 1991 to December 1993		Biological-naïve				Steroid-free remission/response (maintenance)<br>Discontinuations		
Mowat 2016 (67)<br><br>TOPPIC	ISRCTN89489788<br><br>NR	UK<br><br>29 study sites<br><br>Screening: June 2008 to April 2012	Maintenance study	Patients (at least 16 or 18 years, depending on the country they were from) with a diagnosis of Crohn's disease and resection within the preceding 3 months<br><br>Combination of biological-naïve and biological-exposed	240	Mercaptopurine 1 mg/kg once per day	Placebo	Maintenance of clinical remission and response combined<br>Steroid-free remission/response (maintenance)<br>Discontinuations<br>Discontinuations due to adverse events<br>Malignancies	157 weeks	Some concerns
Oren 1997 (68)	NR<br><br>NR	Isreal<br><br>12 study sites<br><br>January 1992 to April 1995	Induction and maintenance study<br><br>Treat-through	Patients (17-75 years) with definite, chronic active Crohn's disease (diagnosed by the usual clinical, radiographic, endoscopic and pathologic criteria) for at least 1 year<br><br>HBI ≥ 7<br><br>Biological-naïve	84	Methotrexate 12.5 mg once per week<br><br>6-mercaptopurine 50 mg once per day	Placebo	Induction of clinical remission<br>Steroid-free remission (induction)<br>Discontinuations<br>Discontinuations due to adverse events	39 weeks	Some concerns
Orlando 2020 (69)	NR<br><br>NR	Italy<br><br>11 study sites<br><br>April 2005 to June 2010	Maintenance study	Patients (18-75 years) with Crohn's disease who had been treated with a curative resection and had severe endoscopic recurrence	46	5-aminosalicylic acid 4000 mg once per day<br><br>Azathioprine 2-2.5 mg/kg once per day	-	Maintenance of clinical remission<br>Discontinuations<br>Discontinuations due to adverse events	52 weeks	Some concerns

Reference<br><br>Acronym	Trial registration number<br><br>Study phase	Countries<br><br>Number of study sites<br><br>Study dates	Study type*	Population	Sample size	Intervention(s): start dosage and -frequency	Compara-tor(s)	Outcomes	Follow-up duration	Overall RoB
				Previous treatment with biologicals unclear						
Panés 2017 (70)	Induction: NCT01393626<br><br>Phase 2	Worldwide (18 countries)<br><br>80 study sites<br><br>October 2011 to March 2015	Induction study	Patients (18-75 years) with moderate-to-severe Crohn's disease for at least 6 months<br><br>CDAI 220-450<br><br>Combination of biological-naïve and biological-exposed	280	Tofacitinib 5 mg two times per day<br><br>Tofacitinib 10 mg two times per day<br><br>Tofacitinib 15 mg two times per day	Placebo	Induction of clinical remission<br>Induction of clinical response	8 weeks	High
	Maintenance: NCT01393899<br><br>Phase 2	Worldwide (18 countries)<br><br>80 study sites<br><br>March 2012 to July 2015	Maintenance study<br><br>Responder re-randomization		180	Tofacitinib 5 mg two times per day<br><br>Tofacitinib 10 mg two times per day	Placebo	Maintenance of clinical remission<br>Discontinuations<br>Discontinuations due to adverse events<br>Serious infections<br>Malignancies	26 weeks	
Prantera 1992 (71)	NR<br><br>NR	Italy<br><br>8 study sites<br><br>April 1988 to October 1989	Maintenance study	Patients (18-65 years) with Crohn's disease who were asymptomatic and in remission for 3 months to 2 years<br><br>CDAI < 150<br><br>Biological-naïve	125	5-aminosalicylic acid 800 mg three times per day	Placebo	Maintenance of clinical remission and response combined<br>Steroid-free remission/response (maintenance)<br>Discontinuations<br>Discontinuations due to adverse events	52 weeks	Some concerns
Prantera 1999 (72)	NR<br><br>NR	Italy<br><br>14 study sites	Induction study	Patients (at least 18 years) with Crohn's disease (diagnosed by endoscopy	94	Mesalamine 1333 mg three times per day	-	Induction of clinical remission<br>Steroid-free remission (induction)	12 weeks	Some concerns

Reference<br><br>Acronym	Trial registration number<br><br>Study phase	Countries<br><br>Number of study sites<br><br>Study dates	Study type*	Population	Sample size	Intervention(s): start dosage and -frequency	Compara-tor(s)	Outcomes	Follow-up duration	Overall RoB
		June 1994 to September 1996		or radiography) with a flare-up<br><br>CDAI 180-350<br><br>Biological-naïve		(two dosage forms)<br><br>6-methylprednisolone 13 mg three times per day				
Regueiro 2016 (73)<br><br>PREVENT	NCT01190839<br><br>Phase 3	Worldwide (14 countries)<br><br>104 study sites<br><br>November 2010 to May 2012	Maintenance study	Patients (at least 18 years) with a confirmed diagnosis of Crohn's disease who had undergone resection in the previous 45 days<br><br>CDAI < 200<br><br>Combination of biological-naïve and biological-exposed	297	Infliximab 5 mg/kg once per 8 weeks	Placebo	Maintenance of clinical remission and response combined<br>Steroid-free remission/response (maintenance)<br>Discontinuations<br>Discontinuations due to adverse events<br>Serious infections<br>Malignancies	104 weeks	Some concerns
Reinisch 2008 (74)	NR<br><br>NR	Europe, Canada, Russia, South Africa<br><br>38 study sites<br><br>April 2004 to February 2006	Maintenance study	Patients with a diagnosis of Crohn's disease (confirmed by radiological, endoscopic, and/or histological examination) within 5 years prior to study entry and currently had active disease<br><br>CDAI 220-450<br><br>Biological-naïve	81	Azathioprine 2.5 mg/kg once per day	Placebo	Maintenance of clinical remission<br>Steroid-free remission (maintenance)<br>Discontinuations<br>Discontinuations due to adverse events	52 weeks	Some concerns
Reinisch 2010 (75)<br><br>AZT-2	NCT00946946<br><br>NR	Austria, Czech Republic, Germany, Israel	Maintenance study	Patients (18-70 years) with a diagnosis of Crohn's disease (confirmed by endoscopy and histology)	78	Azathioprine 2-2.5 mg/kg once per day	-	Maintenance of clinical remission and response combined	52 weeks	Some concerns

Reference<br><br>Acronym	Trial registration number<br><br>Study phase	Countries<br><br>Number of study sites<br><br>Study dates	Study type*	Population	Sample size	Intervention(s): start dosage and -frequency	Compara-tor(s)	Outcomes	Follow-up duration	Overall RoB
		21 study sites<br><br>February 2002 to May 2007		who had undergone resection in the previous 6-24 months and have not had clinical recurrence<br><br>CDAI < 200<br><br>Combination of biological-naïve and biological-exposed		Mesalazine 4000 mg once per day		Steroid-free remission/response (maintenance)<br>Discontinuations<br>Discontinuations due to adverse events		
Reinshagen 2007 (76)	NR<br><br>NR	Germany<br><br>11 study sites<br><br>January 2001 to December 2002	Induction study	Patients (18-75 years) with active Crohn's disease (confirmed by clinical, radiological and histological criteria)<br><br>CDAI 150-450<br><br>Biological-naïve	58	Azathioprine 2.5 mg/kg once per day (two treatment strategies)	-	Induction of clinical remission<br>Steroid-free remission (induction)	22 weeks	Some concerns
Rutgeerts 1994 (77)	NR<br><br>NR	Europe<br><br>11 study sites<br><br>NR	Induction study	Patients (at least 18 years) with active Crohn's disease<br><br>CDAI ≥ 200<br><br>Biological-naïve	176	Budesonide 9 mg once per day<br><br>Prednisolone 40 mg once per day	-	Induction of clinical remission<br>Induction of clinical remission and response combined	10 weeks	High
Rutgeerts 1999 (78)	NR<br><br>NR	Europe, North America<br><br>17 study sites<br><br>NR	Maintenance study<br><br>Responder re-randomization	Patients (18-65 years) with moderate-to-severe treatment-resistant Crohn's disease who had a clinical response to infliximab	73	Infliximab 10 mg/kg once per 8 weeks	Placebo	Maintenance of clinical remission<br>Discontinuations<br>Discontinuations due to adverse events	36 weeks	Some concerns

Reference<br><br>Acronym	Trial registration number<br><br>Study phase	Countries<br><br>Number of study sites<br><br>Study dates	Study type*	Population	Sample size	Intervention(s): start dosage and -frequency	Compara-tor(s)	Outcomes	Follow-up duration	Overall RoB
				Combination of biological-naïve and biological-exposed						
Rutgeerts 2012 (79)<br><br>EXTEND	NCT00348283<br><br>NR	Austria, Belgium, Canada, France, Germany, Italy, Netherlands, USA<br><br>19 study sites<br><br>August 2006 to September 2008	Maintenance study	Patients (18-75 years) with a diagnosis of Crohn's disease for at least 4 months<br><br>CDAI 220-450<br><br>Combination of biological-naïve and biological-exposed	129	Adalimumab 40 mg once per 2 weeks	Placebo	Maintenance of clinical remission<br>Discontinuations due to adverse events<br>Serious infections<br>Malignancies<br>Major cardiovascular events (including thrombosis)	52 weeks	Some concerns
Sandborn 1999 (80)	NR<br><br>NR	Canada, USA<br><br>16 study sites<br><br>September 1996 to November 1997	Induction study	Patients (at least 18 years) with active steroid-treated Crohn's disease<br><br>CDAI 150-450<br><br>Biological-naïve	96	Azathioprine 40 mg/kg once<br><br>Azathioprine 2 mg/kg once per day	-	Induction of clinical remission<br>Induction of clinical response<br>Steroid-free remission (induction)	16 weeks	Some concerns
Sandborn 2001 (81)	NR<br><br>NR	Canada, UK, USA<br><br>21 study sites<br><br>June 1998 to June 1999	Induction study	Patients (at least 18 years) with moderate-to-severely active Crohn's disease, confirmed by radiologic, endoscopic or histologic criteria<br><br>CDAI 220-450<br><br>Biological-naïve	193	CDP571 10 mg/kg once<br><br>CDP571 20 mg/kg once	Placebo	Induction of clinical response	2 weeks	High

Reference<br><br>Acronym	Trial registration number<br><br>Study phase	Countries<br><br>Number of study sites<br><br>Study dates	Study type*	Population	Sample size	Intervention(s): start dosage and -frequency	Compara-tor(s)	Outcomes	Follow-up duration	Overall RoB
Sandborn 2004 (82)	NR<br><br>Phase 3	Australia, Bulgaria, Canada, Czech Republic, Hungary, Poland, USA<br><br>68 study sites<br><br>January 2001 to May 2002	Induction study	Patients (at least 18 years) with moderate-to-severe Crohn's disease, confirmed by radiological, endoscopic or histological evidence<br><br>CDAI 220-450<br><br>Combination of biological-naïve and biological-exposed	396	CDP571 10 mg/kg once per 8 weeks	Placebo	Induction of clinical remission<br>Induction of clinical response	28 weeks	Some concerns
Sandborn 2005 (83)<br><br>ENACT-1<br>ENACT-2	ENACT-1: NCT00032786<br><br>Phase 2	Worldwide (16 countries)<br><br>142 study sites<br><br>December 2001 to March 2004	Induction study	Patients (at least 18 years) with moderately-to-severely active Crohn's disease (with radiologic or endoscopic evidence) for at least 6 months<br><br>CDAI 220-450<br><br>Combination of biological-naïve and biological-exposed	905	Natalizumab 300 mg once per 4 weeks	Placebo	Induction of clinical remission<br>Induction of clinical response	12 weeks	Some concerns
	ENACT-2: NCT00032799<br><br>Phase 2	Worldwide (16 countries)<br><br>142 study sites<br><br>December 2001 to March 2004	Maintenance study<br><br>Responder re-randomization		339	Natalizumab 300 mg once per 4 weeks	Placebo	Maintenance of clinical remission<br>Steroid-free remission (maintenance)<br>Discontinuations due to adverse events<br>Serious infections	48 weeks	
Sandborn 2007a (84)<br><br>CLASSIC-II	NR<br><br>NR	Belgium, Canada, Czech Republic, Netherlands, Poland, USA<br><br>53 study sites	Maintenance study<br><br>Responder re-randomization	All patients who met study entry criteria and successfully completed CLASSIC-I<br><br>Biological-naïve	55	Adaliumab 40 mg once per 2 weeks<br><br>Adalimumab 40 mg once per week	Placebo	Maintenance of clinical remission<br>Discontinuations<br>Discontinuations due to adverse events<br>Serious infections<br>Malignancies	52 weeks	Some concerns

Reference<br><br>Acronym	Trial registration number<br><br>Study phase	Countries<br><br>Number of study sites<br><br>Study dates	Study type*	Population	Sample size	Intervention(s): start dosage and -frequency	Compara-tor(s)	Outcomes	Follow-up duration	Overall RoB
		August 2002 to January 2005								
Sandborn 2007b (85)<br><br>PRECiSE 1	NCT00152490<br><br>NR	Worldwide (20 countries)<br><br>171 study sites<br><br>December 2003 to May 2005	Induction and maintenance study<br><br>Treat-through	Patients (at least 18 years) who had active Crohn's disease for at least 3 months<br><br>CDAI 220-450<br><br>Combination of biological-naïve and biological-exposed	662	Certolizumab pegol 400 mg once per 2 weeks	Placebo	Induction of clinical remission<br>Induction of clinical response<br>Maintenance of clinical remission<br>Steroid-free response (induction)<br>Discontinuations<br>Discontinuations due to adverse events<br>Serious infections<br>Malignancies	26 weeks	Low
Sandborn 2007c (86)	NCT00105300<br><br>NR	Belgium, Canada, France, USA<br><br>52 study sites<br><br>November 2004 to June 2006	Induction study	Patients (18-75 years) with Crohn's disease (with radiologic or endoscopic evidence) for at least 4 months that was moderately-to-severely active, who were intolerant to or lost response to Infliximab<br><br>CDAI 220-450<br><br>Biological-exposed	325	Adalimumab 160 mg once per 2 weeks	Placebo	Induction of clinical remission<br>Induction of clinical response<br>Steroid-free remission (induction)	4 weeks	Low
Sandborn 2008 (87)	NCT00265122<br><br>NR	Belgium, Canada, USA<br><br>49 study sites	Induction study	Patients (at least 18 years) with moderate-to-severe Crohn's disease (confirmed by radiography	27	Ustekinumab 90 mg once per week	-	Induction of clinical remission<br>Induction of clinical response	28 weeks	High

Reference<br><br>Acronym	Trial registration number<br><br>Study phase	Countries<br><br>Number of study sites<br><br>Study dates	Study type*	Population	Sample size	Intervention(s): start dosage and -frequency	Compara-tor(s)	Outcomes	Follow-up duration	Overall RoB
		May 2004 to October 2006		or endoscopy) of at least 6 weeks' duration<br><br>CDAI 220-450<br><br>Biological-exposed		Ustekinumab 4.5 mg/kg once				
Sandborn 2011 (88)	NR<br><br>NR	Worldwide (20 countries)<br><br>120 study sites<br><br>March 2008 to June 2009	Induction study	Patients (18-75 years) with active moderate-to-severe Crohn's disease<br><br>CDAI 220-450<br><br>Biological-naïve	439	Certolizumab pegol 400 mg once per 2 weeks	Placebo	Induction of clinical remission<br>Induction of clinical response	6 weeks	Some concerns
Sandborn 2012a (89)<br><br>CERTIFI	NCT00771667<br><br>Phase 3	Worldwide (12 countries)<br><br>153 study sites<br><br>October 2008 to December 2010	Induction study	Patients (at least 18 years) with Crohn's disease for at least 3 months, who did not respond to who had unacceptable side effects after receiving a TNF-antagonist<br><br>CDAI 220-450<br><br>Biological-exposed	526	Ustekinumab 1 mg/kg once<br><br>Ustekinumab 3 mg/kg once<br><br>Ustekinumab 6 mg/kg once	Placebo	Induction of clinical remission	8 weeks	Some concerns
Sandborn 2012b (90)	NCT00406653<br><br>NR	Worldwide (17 countries)<br><br>142 study sites<br><br>December 2006 to November 2009	Induction and maintenance study<br><br>Responder re-randomization	Patients (at least 18 years) with moderate-to-severe Crohn's disease for at least 3 months, who did not respond to or did not tolerate at least one Crohn's disease therapy	Induction: 451	Abatacept 30 mg/kg once per 2 weeks<br><br>Abatacept 10 mg/kg once per 2 weeks	Placebo	Induction of clinical remission<br>Induction of clinical response	12 weeks	High

Reference<br><br>Acronym	Trial registration number<br><br>Study phase	Countries<br><br>Number of study sites<br><br>Study dates	Study type*	Population	Sample size	Intervention(s): start dosage and -frequency	Compara-tor(s)	Outcomes	Follow-up duration	Overall RoB
				CDAI 220-450<br><br>Combination biological-exposed and -naïve		Abatacept 3 mg/kg once per 2 weeks				
					Maintenance: 90	Abatacept 10 mg/kg once per 4 weeks	Placebo	Maintenance of clinical remission<br>Discontinuations<br>Discontinuations due to adverse events<br>Serious infections<br>Malignancies	40 weeks	
Sandborn 2013b (91)<br><br>GEMINI 2<br><br>***Substudies/post-hoc analyses:***<br>*Sands 2017 (92)*<br>*Sands 2019 (93)*	NCT00783692<br><br>Phase 3	Worldwide (39 countries)<br><br>285 study sites<br><br>December 2008 to May 2012	Induction and maintenance study<br><br>Responder re-randomization	Patients (18-80 years old) with Crohn's disease for at least 3 months<br><br>CDAI 220-450<br><br>Combination biological-exposed and -naïve	Induction: 1115	Vedolizumab 300 mg once per 2 weeks	Placebo	Induction of clinical remission<br>Induction of clinical response<br>Steroid-free remission (induction)<br>Steroid-free response (induction)	6 weeks	Some concerns
					Maintenance: 461	Vedolizumab 300 mg once per 8 weeks<br><br>Vedolizumab 300 mg once per 4 weeks	Placebo	Maintenance of clinical remission<br>Steroid-free remission (maintenance)<br>Discontinuations<br>Discontinuations due to adverse events<br>Serious infections<br>Malignancies	46 weeks	
Sandborn 2020 (94)<br><br>CELEST	NCT02365649<br><br>Phase 2	Worldwide (19 countries)<br><br>93 study sites<br><br>March 2015 to August 2017	Induction and maintenance study<br><br>Responder re-randomization	Patients (18-75 years) with Crohn's disease (confirmed by endoscopy) for at least 3 months, who had inadequate response or intolerance to at least one TNF-antagonist or	Induction: 220	Upadacitinib 3 mg two times per day<br><br>Upadacitinib 6 mg two times per day	Placebo	Induction of clinical remission<br>Induction of clinical response<br>Steroid-free remission (induction)	16 weeks	Low

Reference<br><br>Acronym	Trial registration number<br><br>Study phase	Countries<br><br>Number of study sites<br><br>Study dates	Study type*	Population	Sample size	Intervention(s): start dosage and -frequency	Compara-tor(s)	Outcomes	Follow-up duration	Overall RoB
				other Crohn's disease therapy<br><br>CDAI 220-450<br><br>Combination biological-exposed and -naïve		Upadacitinib 12 mg two times per day<br><br>Upadacitinib 24 mg two times per day<br><br>Upadacitinib 24 mg once per day				
					Maintenance: 180	Upadacitinib 3 mg two times per day<br><br>Upadacitinib 6 mg two times per day<br><br>Upadacitinib 12 mg two times per day<br><br>Upadacitinib 24 mg once per day	-	Maintenance of clinical remission<br>Steroid-free remission (maintenance)<br>Discontinuations<br>Discontinuations due to adverse events<br>Serious infections<br>Malignancies<br>Major cardiovascular events (including thrombosis)	36 weeks	
Sandborn 2022 (95)<br><br>GALAXI-1	NCT03466411<br><br>Phase 2	Worldwide (32 countries)<br><br>128 study sites<br><br>Started April 2018	Induction study	Patients (at least 18 years) with moderately-to-severely active Crohn's disease (with endoscopic evidence) for at least 3 months, and who had inadequate response or intolerance to prior conventional treatment	309	Guselkumab 200 mg once per 4 weeks<br><br>Guselkumab 600 mg once per 4 weeks	Placebo	Induction of clinical remission<br>Induction of clinical remission and response combined	12 weeks	Some concerns

Reference<br><br>Acronym	Trial registration number<br><br>Study phase	Countries<br><br>Number of study sites<br><br>Study dates	Study type*	Population	Sample size	Intervention(s): start dosage and -frequency	Compara-tor(s)	Outcomes	Follow-up duration	Overall RoB
				CDAI 220-450<br><br>Combination biological-exposed and -naïve		Guselkumab 1200 mg once per 4 weeks<br><br>Ustekinumab 6 mg/kg once per 8 weeks				
Sandborn 2023 (96)<br><br>BERGAMOT	NCT02394028<br><br>Phase 3	Worldwide (33 countries)<br><br>326 study sites<br><br>March 2015 to September 2021	Induction and maintenance study<br><br>Responder re-randomization	Patients (18-80 years) with moderately-to-severely active Crohn's disease (confirmed by clinial, endoscopic and histopathological evidence) for at least 3 months with intolerance, inadequate response or no response to one or more treatments<br><br>CDAI 220-480<br><br>Combination biological-exposed and -naïve	Induction: 385	Etrolizumab 105 mg once per 4 weeks<br><br>Etrolizumab 210 mg once per multiple weeks	Placebo	Induction of clinical remission	14 weeks	Low
					Maintenance: 487	Etrolizumab 105 mg once per 4 weeks	Placebo	Maintenance of clinical remission<br>Discontinuations<br>Discontinuations due to adverse events<br>Serious infections	52 weeks	
Sands 2004 (97)<br><br>ACCENT II	NCT00207766<br><br>NR	Europe, Israel, North America<br><br>45 study sites<br><br>January 2000 to October 2001	Maintenance study	Patients (at least 18 years) with Crohn's disease for at least 3 months and one or more draining abdominal or perianal fistulas<br><br>Biological-naïve	195	Infliximab 5 mg/kg once per 8 weeks	Placebo	Discontinuations due to adverse events<br>Serious infections	40 weeks	Some concerns
Sands 2007 (98)	NR<br><br>NR	USA<br><br>17 study sites	Induction study	Patients (at least 18 years) with active Crohn's disease for at least 6 months who were	79	Natalizumab 300 mg once per 4 weeks +	-	Induction of clinical remission	10 weeks	Some concerns

Reference<br><br>Acronym	Trial registration number<br><br>Study phase	Countries<br><br>Number of study sites<br><br>Study dates	Study type*	Population	Sample size	Intervention(s): start dosage and -frequency	Compara-tor(s)	Outcomes	Follow-up duration	Overall RoB
		November 2002 to December 2003		receiving Infliximab but who were not in remission<br><br>CDAI ≥ 150<br><br>Biological-exposed		Infliximab 5 mg/kg once<br><br>Infliximab 5 mg/kg once				
Sands 2010 (99)	NR<br><br>Phase 2	Europe, North America<br><br>60 study sites<br><br>September 2005 to June 2006	Induction and maintenance study<br><br>Treat-through	Patients (18-75 years) with Crohn's disease<br><br>CDAI 220-450<br><br>Combination biological-exposed and -naïve	220	Apilimod 50 mg once per day<br><br>Apilimod 100 mg once per day	Placebo	Induction of clinical remission<br>Induction of clinical response<br>Maintenance of clinical remission<br>Discontinuations<br>Discontinuations due to adverse events	28 weeks	Some concerns
Sands 2014 (100)<br><br>GEMINI 3<br><br>*Substudies/post-hoc analyses: Sands 2019* (93)	NCT01224171<br><br>Phase 3	Worldwide<br><br>107 study sites<br><br>November 2010 to April 2012	Induction study	Patients (18-80 years) with a diagnosis of Crohn's disease based on clinical and endoscopic evidence, who had experienced inadequate response, loss of response or intolerance to a Crohn's disease therapy<br><br>CDAI 220-400<br><br>Combination biological-exposed and -naïve	416	Vedolizumab 300 mg at week 0, 2, 6	Placebo	Induction of clinical remission<br>Induction of clinical response<br>Steroid-free remission (induction)<br>Steroid-free response (induction)	10 weeks	Some concerns
Sands 2020 (101)	NCT02596893<br><br>Phase 3	Worldwide (34 countries)<br><br>538 study sites	Induction study	Patients (at least 18 years) with active Crohn's disease for at least 3 months, who had therapeutic failure or	701	Mongersen 160 mg once per day	Placebo	Induction of clinical remission<br>Induction of clinical response	12 weeks	High

Reference<br><br>Acronym	Trial registration number<br><br>Study phase	Countries<br><br>Number of study sites<br><br>Study dates	Study type*	Population	Sample size	Intervention(s): start dosage and -frequency	Comparator(s)	Outcomes	Follow-up duration	Overall RoB
		December 2015 to January 2018		intolerance to one or more therapies<br><br>CDAI 220-450<br><br>Combination biological-exposed and -naïve						
Sands 2022 (102)<br><br>SEAVUE	NCT03464136<br><br>Phase 3	Worldwide (18 countries)<br><br>121 study sites<br><br>June 2018 to December 2019	Induction and maintenance study<br><br>Treat-through	Patients (at least 18 years) with moderately-to-severely active Crohn's disease for at least 3 months, who had not responded to or were intolerant to conventional therapy<br><br>CDAI 220-450<br><br>Biological-naïve	386	Ustekinumab 6 mg/kg once per day<br><br>Adalimumab 160 mg once per day	-	Induction of clinical remission<br>Induction of clinical response<br>Maintenance of clinical remission<br>Discontinuations<br>Discontinuations due to adverse events<br>Serious infections<br>Malignancies	52 weeks	Some concerns
Stack 1997 (103)	NR<br><br>NR	NR<br><br>NR<br><br>NR	Induction study	Patients (18-80 years) with mild-to-moderately active Crohn's disease, confirmed by radiological and histological criteria<br><br>CDAI 150-400<br><br>Biological-naïve	31	CDP571 5 mg/kg once	Placebo	Induction of clinical remission	8 weeks	Some concerns
Steenholdt 2014 (104)	NCT00851565<br><br>NR	Denmark<br><br>6 study sites<br><br>June 2009 to August 2011	Induction study	Patients (at least 18 years) with active Crohn's disease and previously beneficial clinical response to standard Infliximab maintenance therapy and	69	Infliximab 5 mg/kg once per 4 weeks or Adalimumab 80 mg once per 2 weeks	-	Induction of clinical remission<br>Induction of clinical response	12 weeks	High

Reference<br><br>Acronym	Trial registration number<br><br>Study phase	Countries<br><br>Number of study sites<br><br>Study dates	Study type*	Population	Sample size	Intervention(s): start dosage and -frequency	Compara-tor(s)	Outcomes	Follow-up duration	Overall RoB
				now had secondary treatment failure<br><br>CDAI ≥ 220<br><br>Biological-exposed		Infliximab 5 mg/kg once per 4 weeks				
Sutherland 1997 (105)	NR<br><br>NR	Canada<br><br>31 study sites<br><br>January 1990 to January 1993	Maintenance study	Patients (at least 18 years) with Crohn's disease (confirmed by X-ray, surgery, endoscopy or pathology) and at least 2 flare-ups in the past 4 years, currently in remission<br><br>CDAI < 150<br><br>Biological-naïve	293	Mesalamine 750 mg four times per day	Placebo	Maintenance of clinical remission and response combined<br>Steroid-free remission/response (maintenance)<br>Discontinuations<br>Discontinuations due to adverse events	48 weeks	Some concerns
Targan 1997 (106)	NR<br><br>NR	Europe, North America<br><br>18 study sites<br><br>June 1995 to March 1996	Induction study	Patients with Crohn's disease for at least 6 months<br><br>CDAI 220-400<br><br>Biological-naïve	108	Infliximab 5 mg/kg once<br><br>Infliximab 10 mg/kg once<br><br>Infliximab 20 mg/kg once	Placebo	Induction of clinical remission<br>Induction of clinical response	4 weeks	Some concerns
Targan 2007 (107)<br><br>ENCORE	NCT00078611<br><br>Phase 3	Worldwide (11 countries)<br><br>114 study sites<br><br>March 2004 to March 2005	Induction study	Patients (at least 18 years) with moderately-to-severely active Crohn's disease (confirmed by radiology, endoscopy or surgery) for at least 6 months	509	Natalizumab 300 mg once per 4 weeks	Placebo	Induction of clinical remission<br>Induction of clinical response	12 weeks	Some concerns

Reference<br><br>Acronym	Trial registration number<br><br>Study phase	Countries<br><br>Number of study sites<br><br>Study dates	Study type*	Population	Sample size	Intervention(s): start dosage and -frequency	Compara-tor(s)	Outcomes	Follow-up duration	Overall RoB
				CDAI 220-450<br><br>Combination biological-exposed and -naïve						
Targan 2016 (108)	NR<br><br>Phase 2	Australia, Belgium, Canada, France, Netherlands, Poland, Spain, USA<br><br>39 study sites<br><br>November 2010 to October 2011	Induction study	Patients (18-65 years) with moderate-to-severely active Crohn's disease for at least 6 months<br><br>CDAI 250-450<br><br>Combination biological-exposed and -naïve	130	Brodalumab 210 mg once per 4 weeks<br><br>Brodalumab 350 mg once per 4 weeks<br><br>Brodalumab 700 mg once per 4 weeks	Placebo	Induction of clinical remission<br>Induction of clinical response	6 weeks	High
Thomsen 1998 (109)	NR<br><br>NR	Worldwide (12 countries)<br><br>25 study sites<br><br>November 1994 to August 1996	Induction study	Patients (at least 18 years) with a confirmed diagnosis of Crohn's disease<br><br>CDAI 200-400<br><br>Biological-naïve	182	Budesonide 9 mg once per day<br><br>Mesalamine 2000 mg two times per day	-	Induction of clinical remission<br>Induction of clinical remission and response combined	16 weeks	Some concerns
Thomson 1995 (110)	NR<br><br>NR	Europe (10 countries), Canada<br><br>29 study sites<br><br>December 1988 to January 1992	Maintenance study	Patients (18-70 years) with Crohn's disease, confirmed by radiology, endoscopy or surgery, and at least one period of clinical activity within 18 months prior<br><br>CDAI < 150<br><br>Biological-naïve	286	Mesalazine 1500 mg ocne per day	Placebo	Maintenance of clinical remission and response combined<br>Discontinuations<br>Discontinuations due to adverse events	52 weeks	Some concerns

Reference<br><br>Acronym	Trial registration number<br><br>Study phase	Countries<br><br>Number of study sites<br><br>Study dates	Study type*	Population	Sample size	Intervention(s): start dosage and -frequency	Compara-tor(s)	Outcomes	Follow-up duration	Overall RoB
Tremaine 1994 (111)	NR<br><br>NR	USA<br><br>1 study site<br><br>NR	Induction study	Patients (at least 18 years) with mild-to-moderately active Crohn's disease<br><br>CDAI 150-450<br><br>Biological-naïve	38	5-aminosalicylic acid 800 mg four times per day	Placebo	Induction of clinical remission and response combined	17 weeks	Some concerns
Tremaine 2002 (112)	NR<br><br>NR	USA<br><br>24 study sites<br><br>October 1995 to August 1997	Induction study	Patients (at least 18 years) with mild-to-moderate Crohn's disease, confirmed by colonoscopy and X-ray<br><br>CDAI 200-450<br><br>Biological-naïve	200	Budesonide 9 mg once per day<br><br>Budesonide 4.5 mg two times per day	Placebo	Induction of clinical remission	8 weeks	Some concerns
Tromm 2011 (113)	NCT00300118<br><br>Phase 3	Croatia, Czech Republic, Germany, Greece, Hungary, Israel, Slovak Republic<br><br>46 study sites<br><br>November 2004 to May 2008	Induction study	Patients (18-70 years) with Crohn's disease and symptoms for at least 3 months, confirmed by endoscopy with histology or radiology<br><br>CDAI 201-399<br><br>Biological-naïve	311	Budesonide 3 mg three times per day<br><br>Budesonide 9 mg once per day<br><br>Mesalamine 1500 mg three times per day	-	Induction of clinical remission<br>Induction of clinical remission and response combined<br>Steroid-free remission (induction)	8 weeks	High
Van Assche 2012 (114)<br><br>SWITCH	NR<br><br>NR	Belgium<br><br>1 study site<br><br>NR	Maintenance study	Patients (at least 18 yearS0 with Crohn's disease on scheduled Infliximab maintenance therapy<br><br>CDAI < 200	73	Adalimumab 80 mg once per 2 weeks<br><br>Infliximab 5 mg/kg once per 6-8 weeks	-	Discontinuations<br>Discontinuations due to adverse events	56 weeks	Some concerns

Reference<br><br>Acronym	Trial registration number<br><br>Study phase	Countries<br><br>Number of study sites<br><br>Study dates	Study type*	Population	Sample size	Intervention(s): start dosage and -frequency	Comparator(s)	Outcomes	Follow-up duration	Overall RoB
				Biological-exposed						
Van Linschoten 2023 (115)<br><br>LADI	NCT03172377<br><br>NR	Netherlands<br><br>20 study sites<br><br>Enrollment: May 2017 to July 2020	Maintenance study	Patients (at least 18 years) with Crohn's disease in remission for at least 9 months on a stable dose of Adalimumab<br><br>HBI < 5<br><br>Biological-exposed	174	Adalimumab 40 mg once per 3 weeks<br><br>Adalimumab 40 mg once per 2 weeks	-	Maintenance of clinical remission<br>Steroid-free remission (maintenance)<br>Discontinuations	48 weeks	Some concerns
Vermeire 2022 (116)<br><br>VISIBLE 2	NCT02611817<br><br>Phase 3	Worldwide (30 countries)<br><br>169 study sites<br><br>December 2015 to May 2019	Maintenance study	Patients (18-80 years) with moderately-to-severely active Crohn's disease for at least 3 months, confirmed by clinical, endoscopic and histopathological evidence, and inadequate response to, loss of response to or intolerance to one or more therapies<br><br>CDAI 220-450<br><br>Combination biological-exposed and -naïve	410	Vedolizumab 108 mg once per 2 weeks	Placebo	Maintenance of clinical remission<br>Discontinuations<br>Discontinuations due to adverse events<br>Serious infections	46 weeks	Low
Watanabe 2012 (117)	Induction: NCT00445939<br><br>NR	Japan<br><br>29 study sites	Induction study	Patients (15-75 years) with moderate-to-severely active Crohn's disease for at least 4 months,	90	Adalimumab 160 mg once per 2 weeks	Placebo	Induction of clinical remission<br>Induction of clinical response	4 weeks	Some concerns

Reference<br><br>Acronym	Trial registration number<br><br>Study phase	Countries<br><br>Number of study sites<br><br>Study dates	Study type*	Population	Sample size	Intervention(s): start dosage and -frequency	Compara-tor(s)	Outcomes	Follow-up duration	Overall RoB
		January 2007 to December 2007		confirmed by endoscopy or radiology		Adalimumab 80 mg once per 2 weeks				
	Maintenance: NCT00445432<br><br>NR	Japan<br><br>29 study sites<br><br>March 2007 to December 2008	Maintenance study<br><br>Responder re-randomization	CDAI 220-450<br><br>Combination biological-exposed and -naïve	50	Adalimumab 40 mg once per 2 weeks	Placebo	Maintenance of clinical remission<br>Steroid-free remission (maintenance)<br>Discontinuations<br>Discontinuations due to adverse events<br>Serious infections<br>Malignancies<br>Major cardiovascular events (including thrombosis)	52 weeks	
Watanabe 2020 (118)	NCT02038920<br><br>Phase 3	Japan<br><br>77 study sites<br><br>January 2014 to November 2017	Induction and maintenance study<br><br>Responder re-randomization	Patients (15-80 years) with Crohn's disease for at least 3 months, who had treatment failure or intolerance to one or more therapies in the previous 5 years<br><br>CDAI 220-450<br><br>Combination biological-exposed and -naïve	Induction: 157	Vedolizumab 300 mg once per 2 weeks	Placebo	Induction of clinical remission<br>Induction of clinical response	10 weeks	Some concerns
					Maintenance: 42	Vedolizumab 300 mg once per 8 weeks	Placebo	Maintenance of clinical remission<br>Steroid-free remission (maintenance)<br>Discontinuations<br>Discontinuations due to adverse events	50 weeks	
Wenckert 1978 (119)	NR<br><br>NR	Denmark, Sweden<br><br>7 study sites<br><br>Started 1969	Maintenance study	Patients with Crohn's disease who had undergone a first resection<br><br>Biological-naïve	66	Salazosulphapyridine 3000 mg once per day	Placebo	Discontinuations<br>Discontinuations due to adverse events	104 weeks	Some concerns

Reference<br><br>Acronym	Trial registration number<br><br>Study phase	Countries<br><br>Number of study sites<br><br>Study dates	Study type*	Population	Sample size	Intervention(s): start dosage and -frequency	Compara-tor(s)	Outcomes	Follow-up duration	Overall RoB
Wenzl 2015 (120)	NR<br><br>NR	Austria<br><br>2 study sites<br><br>NR	Maintenance study	Patients (19-70 years) with Crohn's disease and in remission on continuous Azathioprine therapy for at least 4 years<br><br>CDAI < 150<br><br>Biological-naïve	52	Azathioprine 1.7 mg/kg once per day	Placebo	Maintenance of clinical remission and response combined<br>Steroid-free remission/response (maintenance)<br>Discontinuations<br>Discontinuations due to adverse events<br>Malignancies	104 weeks	Some concerns
Ye 2019 (121)	NCT02096861<br><br>Phase 3	Worldwide (16 countries)<br><br>58 study sites<br><br>August 2014 to February 2017	Induction study	Patients (18-75 years) with active Crohn's disease for at least 12 weeks who had not previously received any biological treatment<br><br>CDAI 220-450<br><br>Biological-naïve	220	CT-P13 5 mg/kg once per 2 weeks<br><br>Infliximab 5 mg/kg once per 2 weeks	-	Induction of clinical remission<br>Induction of clinical response	14 weeks	Low
Yokoyama 2018 (122)	NCT01514240<br><br>Phase 3	Japan<br><br>31 study sites<br><br>February 2012 to September 2014	Induction study	Patients (at least 15 years) with a diagnosis of active Crohn's disease, confirmed by X-ray, endoscopy or histology<br><br>CDAI 180-400<br><br>Combination biological-exposed and -naïve	112	Budesonide 9 mg once per day<br><br>Mesalazine 1000 mg three times per day	-	Induction of clinical remission and response combined<br>Steroid-free remission/response (induction)	8 weeks	Some concerns
Zhang 2016 (123)	NR<br><br>NR	China<br><br>1 study site	Induction study	Patients (18-65 years) with active Crohn's disease (clinically and pathologically diagnosed)	50	Corticosteroids 0.75-1 mg/kg once per day + Azathioprine 1	-	Induction of clinical remission<br>Steroid-free remission (induction)	48 weeks	Some concerns

Reference<br><br>Acronym	Trial registration number<br><br>Study phase	Countries<br><br>Number of study sites<br><br>Study dates	Study type*	Population	Sample size	Intervention(s): start dosage and -frequency	Compara-tor(s)	Outcomes	Follow-up duration	Overall RoB
		January 2012 to December 2013		in need of systemic steroid therapy<br><br>Biological-naïve		mg/kg once per day<br><br>Corticosteroids 0.75-1 mg/kg once per day + Azathioprine 2 mg/kg once per day				

*Reported study type is dependent on the outcomes that were eligible for inclusion in the systematic literature review, e.g. if a study conducted both an induction and a maintenance phase but the maintenance phase was too short to be included, the study is reported as an induction study.
Abbreviations: CDAI: Crohn's disease activity index; CT: computed tomography; HBI: Harvey Bradshaw index; NR: not reported; RoB: risk of bias; UK: United Kingdom; USA: United States of America.

References

1. Arber N, Odes HS, Fireman Z, Lavie A, Broide E, Bujanover Y, et al. A controlled double blind multicenter study of the effectiveness of 5-aminosalicylic acid in patients with Crohn's disease in remission. Journal of clinical gastroenterology. 1995;20(3):203-6.
2. Ardizzone S, Bollani S, Manzionna G, Imbesi V, Colombo E, Bianchi Porro G. Comparison between methotrexate and azathioprine in the treatment of chronic active Crohn's disease: a randomised, investigator-blind study. Digestive and liver disease. 2003;35(9):619-27.
3. Ardizzone S, Maconi G, Sampietro GM, Russo A, Radice E, Colombo E, et al. Azathioprine and mesalamine for prevention of relapse after conservative surgery for Crohn's disease. Gastroenterology. 2004;127(3):730-40.
4. Bar-Meir S, Chowers Y, Lavy A, Abramovitch D, Sternberg A, Leichtmann G, et al. Budesonide versus prednisone in the treatment of active Crohn's disease. The Israeli Budesonide Study Group. Gastroenterology. 1998;115(4):835-40.
5. Brignola C, Cottone M, Pera A, Ardizzone S, Scribano ML, De Franchis R, et al. Mesalamine in the prevention of endoscopic recurrence after intestinal resection for Crohn's disease. Italian Cooperative Study Group. Gastroenterology. 1995;108(2):345-9.
6. Campieri M, Ferguson A, Doe W, Persson T, Nilsson LG. Oral budesonide is as effective as oral prednisolone in active Crohn's disease. The Global Budesonide Study Group. Gut. 1997;41(2):209-14.
7. Candy S, Wright J, Gerber M, Adams G, Gerig M, Goodman R. A controlled double blind study of azathioprine in the management of Crohn's disease. Gut. 1995;37(5):674-8.

8. Caprilli R, Cottone M, Tonelli F, Sturniolo G, Castiglione F, Annese V, et al. Two mesalazine regimens in the prevention of the post-operative recurrence of Crohn's disease: a pragmatic, double-blind, randomized controlled trial. Alimentary pharmacology & therapeutics. 2003;17(4):517-23.
9. Chen B, Gao X, Zhong J, Ren J, Zhu X, Liu Z, et al. Efficacy and safety of adalimumab in Chinese patients with moderately to severely active Crohn?s disease: results from a randomized trial. Therapeutic advances in gastroenterology. 2020;13.
10. Colombel JF, Sandborn WJ, Rutgeerts P, Enns R, Hanauer SB, Panaccione R, et al. Adalimumab for maintenance of clinical response and remission in patients with Crohn's disease: the CHARM trial. Gastroenterology. 2007;132(1):52-65.
11. Colombel JF, Sandborn WJ, Rutgeerts P, Kamm MA, Yu AP, Wu EQ, et al. Comparison of two adalimumab treatment schedule strategies for moderate-to-severe Crohn's disease: results from the CHARM trial. American journal of gastroenterology. 2009;104(5):1170-9.
12. Kamm MA, Hanauer SB, Panaccione R, Colombel JF, Sandborn WJ, Pollack PF, et al. Adalimumab sustains steroid-free remission after 3 years of therapy for Crohn's disease. Alimentary pharmacology & therapeutics. 2011;34(3):306-17.
13. Sandborn WJ, Colombel JF, D'Haens G, Plevy SE, Pan�s J, Robinson AM, et al. Association of baseline C-reactive protein and prior anti-tumor necrosis factor therapy with need for weekly dosing during maintenance therapy with adalimumab in patients with moderate to severe Crohn's disease. Current medical research and opinion. 2013;29(5):483-93.
14. Colombel JF, Sandborn WJ, Reinisch W, Mantzaris GJ, Kornbluth A, Rachmilewitz D, et al. Infliximab, azathioprine, or combination therapy for Crohn's disease. New England journal of medicine. 2010;362(15):1383-95.
15. Cosnes J, Bourrier A, Laharie D, Nahon S, Bouhnik Y, Carbonnel F, et al. Early administration of azathioprine vs conventional management of Crohn's Disease: a randomized controlled trial. Gastroenterology. 2013;145(4):758-65.e2; quiz e14.
16. D'Haens G, Baert F, van Assche G, Caenepeel P, Vergauwe P, Tuynman H, et al. Early combined immunosuppression or conventional management in patients with newly diagnosed Crohn's disease: an open randomised trial. Lancet (london, england). 2008;371(9613):660-7.
17. D'Haens G, Panaccione R, Baert F, Bossuyt P, Colombel JF, Danese S, et al. Risankizumab as induction therapy for Crohn's disease: results from the phase 3 ADVANCE and MOTIVATE induction trials. Lancet. 2022;399(10340):2015-30.
18. D'Haens GR, Sandborn WJ, Loftus EV, Hanauer SB, Schreiber S, Peyrin-Biroulet L, et al. Higher vs Standard Adalimumab Induction Dosing Regimens and�Two Maintenance Strategies: randomized SERENE CD Trial�Results. Gastroenterology. 2022;162(7):1876-90.
19. D'Haens GR, Lee S, Taylor SA, Serone A, Rimola J, Colombel JF, et al. Filgotinib for the Treatment of Small Bowel Crohn's Disease: The DIVERGENCE 1 Trial. Gastroenterology. 2023;165(1):289-92 e3.
20. de Franchis R, Omodei P, Ranzi T, Brignola C, Rocca R, Prada A, et al. Controlled trial of oral 5-aminosalicylic acid for the prevention of early relapse in Crohn's disease. Alimentary pharmacology & therapeutics. 1997;11(5):845-52.
21. de Jong DJ, Bac DJ, Tan G, de Boer SY, Grabowsky IL, Jansen JB, et al. Maintenance treatment with budesonide 6 mg versus 9 mg once daily in patients with Crohn's disease in remission. Netherlands journal of medicine. 2007;65(9):339-45.
22. Dignass A, Stoynov S, Dorofeyev AE, Grigorieva GA, Tomsov� E, Altorjay I, et al. Once versus three times daily dosing of oral budesonide for active Crohn's disease: a double-blind, double-dummy, randomised trial. Journal of Crohn's & colitis. 2014;8(9):970-80.
23. Ewe K, Press AG, Singe CC, Stufler M, Ueberschaer B, Hommel G, et al. Azathioprine combined with prednisolone or monotherapy with prednisolone in active Crohn's disease. Gastroenterology. 1993;105(2):367-72.
24. Ewe K, B�ttger T, Buhr HJ, Ecker KW, Otto HF. Low-dose budesonide treatment for prevention of postoperative recurrence of Crohn's disease: a multicentre randomized placebo-controlled trial. German Budesonide Study Group. European journal of gastroenterology & hepatology. 1999;11(3):277-82.

25. Feagan BG, Rochon J, Fedorak RN, Irvine EJ, Wild G, Sutherland L, et al. Methotrexate for the treatment of Crohn's disease. The North American Crohn's Study Group Investigators. New England journal of medicine. 1995;332(5):292-7.
26. Feagan BG, Fedorak RN, Irvine EJ, Wild G, Sutherland L, Steinhart AH, et al. A comparison of methotrexate with placebo for the maintenance of remission in Crohn's disease. North American Crohn's Study Group Investigators. New England journal of medicine. 2000;342(22):1627-32.
27. Feagan BG, Sandborn WJ, Gasink C, Jacobstein D, Lang Y, Friedman JR, et al. Ustekinumab as Induction and Maintenance Therapy for Crohn's Disease. New England journal of medicine. 2016;375(20):1946-60.
28. Ferguson A, Campieri M, Doe W, Persson T, Nygards G. Oral budesonide as maintenance therapy in Crohn's disease - results of a 12-month study. Alimentary pharmacology & therapeutics. 1998;12(2):175-83.
29. Ferrante M, Papamichael K, Duricova D, D'Haens G, Vermeire S, Archavlis E, et al. Systematic versus Endoscopy-driven Treatment with Azathioprine to Prevent Postoperative Ileal Crohn's Disease Recurrence. Journal of Crohn's & colitis. 2015;9(8):617-24.
30. Ferrante M, Panaccione R, Baert F, Bossuyt P, Colombel JF, Danese S, et al. Risankizumab as maintenance therapy for moderately to severely active Crohn's disease: results from the multicentre, randomised, double-blind, placebo-controlled, withdrawal phase 3 FORTIFY maintenance trial. Lancet (london, england). 2022;399(10340):2031-46.
31. Fukushima K, Sugita A, Futami K, Takahashi KI, Motoya S, Kimura H, et al. Postoperative therapy with infliximab for Crohn's disease: a 2-year prospective randomized multicenter study in Japan. Surgery today. 2018;48(6):584-90.
32. Gelbmann CM, Rogler G, Gierend M, Gross V, Sch�lmerich J, Andus T. Association of HLA-DR genotypes and IL-1ra gene polymorphism with treatment failure of budesonide and disease patterns in Crohn's disease. European journal of gastroenterology & hepatology. 2001;13(12):1431-7.
33. Gendre JP, Mary JY, Florent C, Modigliani R, Colombel JF, Soul� JC, et al. Oral mesalamine (Pentasa) as maintenance treatment in Crohn's disease: a multicenter placebo-controlled study. The Groupe d'Etudes Th�rapeutiques des Affections Inflammatoires Digestives (GETAID). Gastroenterology. 1993;104(2):435-9.
34. Ghosh S, Goldin E, Gordon FH, Malchow HA, Rask-Madsen J, Rutgeerts P, et al. Natalizumab for active Crohn's disease. New England journal of medicine. 2003;348(1):24-32.
35. Gordon FH, Lai CW, Hamilton MI, Allison MC, Srivastava ED, Fouweather MG, et al. A randomized placebo-controlled trial of a humanized monoclonal antibody to alpha4 integrin in active Crohn's disease. Gastroenterology. 2001;121(2):268-74.
36. Green JR, Lobo AJ, Giaffer M, Travis S, Watkins HC, Freedom Investigator G. Maintenance of Crohn's disease over 12 months: fixed versus flexible dosing regimen using budesonide controlled ileal release capsules. Alimentary pharmacology & therapeutics. 2001;15(9):1331-41.
37. Greenberg GR, Feagan BG, Martin F, Sutherland LR, Thomson AB, Williams CN, et al. Oral budesonide for active Crohn's disease. Canadian Inflammatory Bowel Disease Study Group. New England journal of medicine. 1994;331(13):836-41.
38. Gross V, Andus T, Caesar I, Bischoff SC, Lochs H, Tromm A, et al. Oral pH-modified release budesonide versus 6-methylprednisolone in active Crohn's disease. German/Austrian Budesonide Study Group. European journal of gastroenterology & hepatology. 1996;8(9):905-9.
39. Gross V, Andus T, Ecker KW, Raedler A, Loeschke K, Plauth M, et al. Low dose oral pH modified release budesonide for maintenance of steroid induced remission in Crohn's disease. Gut. 1998;42(4):493-6.
40. Hanauer SB, Feagan BG, Lichtenstein GR, Mayer LF, Schreiber S, Colombel JF, et al. Maintenance infliximab for Crohn's disease: the ACCENT I randomised trial. Lancet (london, england). 2002;359(9317):1541-9.
41. Rutgeerts P, Feagan BG, Lichtenstein GR, Mayer LF, Schreiber S, Colombel JF, et al. Comparison of scheduled and episodic treatment strategies of infliximab in Crohn's disease. Gastroenterology. 2004;126(2):402-13.

42. Hanauer SB, Korelitz BI, Rutgeerts P, Peppercorn MA, Thisted RA, Cohen RD, et al. Postoperative maintenance of Crohn's disease remission with 6-mercaptopurine, mesalamine, or placebo: a 2-year trial. Gastroenterology. 2004;127(3):723-9.
43. Hanauer S, Sandborn WJ, Persson A, Persson T. Budesonide as maintenance treatment in Crohn's disease: a placebo-controlled trial. Alimentary pharmacology & therapeutics. 2005;21(4):363-71.
44. Hanauer SB, Sandborn WJ, Rutgeerts P, Fedorak RN, Lukas M, MacIntosh D, et al. Human anti-tumor necrosis factor monoclonal antibody (adalimumab) in Crohn's disease: the CLASSIC-I trial. Gastroenterology. 2006;130(2):323-33; quiz 591.
45. Hanauer S, Liedert B, Balser S, Brockstedt E, Moschetti V, Schreiber S. Safety and efficacy of BI 695501 versus adalimumab reference product in patients with advanced Crohn's disease (VOLTAIRE-CD): a multicentre, randomised, double-blind, phase 3 trial. The lancet. Gastroenterology & hepatology. 2021;6(10):816-25.
46. Hellers G, Cortot A, Jewell D, Leijonmarck CE, L�fberg R, Malchow H, et al. Oral budesonide for prevention of postsurgical recurrence in Crohn's disease. The IOIBD Budesonide Study Group. Gastroenterology. 1999;116(2):294-300.
47. Hisamatsu T, Kato S, Kunisaki R, Matsuura M, Nagahori M, Motoya S, et al. Withdrawal of thiopurines in Crohn's disease treated with scheduled adalimumab maintenance: a prospective randomised clinical trial (DIAMOND2). Journal of gastroenterology. 2019;54(10):860-70.
48. Hueber W, Sands BE, Lewitzky S, Vandemeulebroecke M, Reinisch W, Higgins PD, et al. Secukinumab, a human anti-IL-17A monoclonal antibody, for moderate to severe Crohn's disease: unexpected results of a randomised, double-blind placebo-controlled trial. Gut. 2012;61(12):1693-700.
49. Anonymous. Coated oral 5-aminosalicylic acid versus placebo in maintaining remission of inactive Crohn's disease. International Mesalazine Study Group. Alimentary pharmacology & therapeutics. 1990;4(1):55-64.
50. J�rgensen KK, Olsen IC, Goll GL, Lorentzen M, Bolstad N, Haavardsholm EA, et al. Switching from originator infliximab to biosimilar CT-P13 compared with maintained treatment with originator infliximab (NOR-SWITCH): a 52-week, randomised, double-blind, non-inferiority trial. Lancet (london, england). 2017;389(10086):2304-16.
51. J�rgensen KK, Goll GL, Sexton J, Bolstad N, Olsen IC, Asak, et al. Efficacy and Safety of CT-P13 in Inflammatory Bowel Disease after Switching from Originator Infliximab: exploratory Analyses from the NOR-SWITCH Main and Extension Trials. BioDrugs. 2020;34(5):681-94.
52. L�mann M, Mary JY, Colombel JF, Duclos B, Soule JC, Lerebours E, et al. A randomized, double-blind, controlled withdrawal trial in Crohn's disease patients in long-term remission on azathioprine. Gastroenterology. 2005;128(7):1812-8.
53. L�mann M, Mary JY, Duclos B, Veyrac M, Dupas JL, Delchier JC, et al. Infliximab plus azathioprine for steroid-dependent Crohn's disease patients: a randomized placebo-controlled trial. Gastroenterology. 2006;130(4):1054-61.
54. Lennard-Jones JE. Sulphasalazine in asymptomatic Crohn's disease. A multicentre trial. Gut. 1977;18(1):69-72.
55. Lochs H, Mayer M, Fleig WE, Mortensen PB, Bauer P, Genser D, et al. Prophylaxis of postoperative relapse in Crohn's disease with mesalamine: european Cooperative Crohn's Disease Study VI. Gastroenterology. 2000;118(2):264-73.
56. L�fberg R, Rutgeerts P, Malchow H, Lamers C, Danielsson A, Olaison G, et al. Budesonide prolongs time to relapse in ileal and ileocaecal Crohn's disease. A placebo controlled one year study. Gut. 1996;39(1):82-6.
57. Loftus EV, Jr., Panes J, Lacerda AP, Peyrin-Biroulet L, D'Haens G, Panaccione R, et al. Upadacitinib Induction and Maintenance Therapy for Crohn's Disease. N Engl J Med. 2023;388(21):1966-80.
58. L�pez-Sanrom�n A, Vera-Mendoza I, Dom�nech E, Taxonera C, Vega Ruiz V, Mar�n-Jim�nez I, et al. Adalimumab vs Azathioprine in the Prevention of Postoperative Crohn's Disease Recurrence. A GETECCU Randomised Trial. Journal of Crohn's & colitis. 2017;11(11):1293-301.
59. Mahida YR, Jewell DP. Slow-release 5-amino-salicylic acid (Pentasa) for the treatment of active Crohn's disease. Digestion. 1990;45(2):88-92.

60. Mantzaris GJ, Petraki K, Sfakianakis M, Archavlis E, Christidou A, Chadio-Iordanides H, et al. Budesonide versus mesalamine for maintaining remission in patients refusing other immunomodulators for steroid-dependent Crohn's disease. Clinical gastroenterology and hepatology. 2003;1(2):122-8.
61. Mantzaris GJ, Christidou A, Sfakianakis M, Roussos A, Koilakou S, Petraki K, et al. Azathioprine is superior to budesonide in achieving and maintaining mucosal healing and histologic remission in steroid-dependent Crohn's disease. Inflammatory bowel diseases. 2009;15(3):375-82.
62. Martin F, Sutherland L, Beck IT, Anderson AH, Williams CN, Saibil F, et al. Oral 5-ASA versus prednisolone in short term treatment of Crohn's disease: a multicentre controlled trial. Can j gastroenterol [conference paper]. 1990;4(7):452-7.
63. Mat�-Jim�nez J, Hermida C, Cantero-Perona J, Moreno-Otero R**.** 6-mercaptopurine or methotrexate added to prednisone induces and maintains remission in steroid-dependent inflammatory bowel disease. European journal of gastroenterology & hepatology. 2000;12(11):1227-33.
64. Matsumoto T, Motoya S, Watanabe K, Hisamatsu T, Nakase H, Yoshimura N, et al. Adalimumab Monotherapy and a Combination with Azathioprine for Crohn's Disease: a Prospective, Randomized Trial. Journal of Crohn's & colitis. 2016;10(11):1259-66.
65. McLeod RS, Wolff BG, Steinhart AH, Carryer PW, O'Rourke K, Andrews DF, et al. Prophylactic mesalamine treatment decreases postoperative recurrence of Crohn's disease. Gastroenterology. 1995;109(2):404-13.
66. Modigliani R, Colombel JF, Dupas JL, Dapoigny M, Costil V, Veyrac M, et al. Mesalamine in Crohn's disease with steroid-induced remission: effect on steroid withdrawal and remission maintenance, Groupe d'Etudes Th�rapeutiques des Affections Inflammatoires Digestives. Gastroenterology. 1996;110(3):688-93.
67. Mowat C, Arnott I, Cahill A, Smith M, Ahmad T, Subramanian S, et al. Mercaptopurine versus placebo to prevent recurrence of Crohn's disease after surgical resection (TOPPIC): a multicentre, double-blind, randomised controlled trial. The lancet gastroenterology and hepatology. 2016;1(4):273-82.
68. Oren R, Moshkowitz M, Odes S, Becker S, Keter D, Pomeranz I, et al. Methotrexate in chronic active Crohn's disease: a double-blind, randomized, Israeli multicenter trial. Am J Gastroenterol. 1997;92(12):2203-9.
69. Orlando A, Mocciaro F, Ventimiglia M, Renna S, Rispo A, Scribano ML, et al. Azathioprine for prevention of clinical recurrence in Crohn's disease patients with severe endoscopic recurrence: an IG-IBD randomized double-blind trial. European review for medical and pharmacological sciences. 2020;24(21):11356-64.
70. Pan�s J, L�pez-Sanrom�n A, Bermejo F, Garc�a-S�nchez V, Esteve M, Torres Y, et al. Early azathioprine therapy is no more effective than placebo for newly diagnosed Crohn's disease. Gastroenterology. 2013;145(4):766-74.e1.
71. Prantera C, Pallone F, Brunetti G, Cottone M, Miglioli M, The Italian IBDSG**.** Oral 5-aminosalicylic acid (Asacol) in the maintenance treatment of Crohn's disease. Gastroenterology. 1992;103(2):363-8.
72. Prantera C, Cottone M, Pallone F, Annese V, Franz� A, Cerutti R, et al. Mesalamine in the treatment of mild to moderate active Crohn's ileitis: results of a randomized, multicenter trial. Gastroenterology. 1999;116(3):521-6.
73. Regueiro M, Feagan BG, Zou B, Johanns J, Blank MA, Chevrier M, et al. Infliximab Reduces Endoscopic, but Not Clinical, Recurrence of Crohn's Disease After Ileocolonic Resection. Gastroenterology. 2016;150(7):1568-78.
74. Reinisch W, Pan�s J, L�mann M, Schreiber S, Feagan B, Schmidt S, et al. A multicenter, randomized, double-blind trial of everolimus versus azathioprine and placebo to maintain steroid-induced remission in patients with moderate-to-severe active Crohn's disease. American journal of gastroenterology. 2008;103(9):2284-92.
75. Reinisch W, Angelberger S, Petritsch W, Shonova O, Lukas M, Bar-Meir S, et al. Azathioprine versus mesalazine for prevention of postoperative clinical recurrence in patients with Crohn's disease with endoscopic recurrence: efficacy and safety results of a randomised, double-blind, double-dummy, multicentre trial. Gut. 2010;59(6):752-9.
76. Reinshagen M, Sch�tz E, Armstrong VW, Behrens C, von Tirpitz C, Stallmach A, et al. 6-thioguanine nucleotide-adapted azathioprine therapy does not lead to higher remission rates than standard therapy in chronic active crohn disease: results from a randomized, controlled, open trial. Clinical chemistry. 2007;53(7):1306-14.

77. Rutgeerts P, L�fberg R, Malchow H, Lamers C, Olaison G, Jewell D, et al. A comparison of budesonide with prednisolone for active Crohn's disease. New England journal of medicine. 1994;331(13):842-5.
78. Rutgeerts P, D'Haens G, Targan S, Vasiliauskas E, Hanauer SB, Present DH, et al. Efficacy and safety of retreatment with anti-tumor necrosis factor antibody (infliximab) to maintain remission in Crohn's disease. Gastroenterology. 1999;117(4):761-9.
79. Rutgeerts P, Van Assche G, Sandborn WJ, Wolf DC, Geboes K, Colombel JF, et al. Adalimumab induces and maintains mucosal healing in patients with Crohn's disease: data from the EXTEND trial. Gastroenterology. 2012;142(5):1102-11.e2.
80. Sandborn WJ, Tremaine WJ, Wolf DC, Targan SR, Sninsky CA, Sutherland LR, et al. Lack of effect of intravenous administration on time to respond to azathioprine for steroid-treated Crohn's disease. North American Azathioprine Study Group. Gastroenterology. 1999;117(3):527-35.
81. Sandborn WJ, Feagan BG, Hanauer SB, Present DH, Sutherland LR, Kamm MA, et al. An engineered human antibody to TNF (CDP571) for active Crohn's disease: a randomized double-blind placebo-controlled trial. Gastroenterology. 2001;120(6):1330-8.
82. Sandborn WJ, Feagan BG, Radford-Smith G, Kovacs A, Enns R, Innes A, et al. CDP571, a humanised monoclonal antibody to tumour necrosis factor alpha, for moderate to severe Crohn's disease: a randomised, double blind, placebo controlled trial. Gut. 2004;53(10):1485-93.
83. Sandborn WJ, Colombel JF, Enns R, Feagan BG, Hanauer SB, Lawrance IC, et al. Natalizumab induction and maintenance therapy for Crohn's disease. New England journal of medicine. 2005;353(18):1912-25.
84. Sandborn WJ, Hanauer SB, Rutgeerts P, Fedorak RN, Lukas M, MacIntosh DG, et al. Adalimumab for maintenance treatment of Crohn's disease: results of the CLASSIC II trial. Gut. 2007;56(9):1232-9.
85. Sandborn WJ, Feagan BG, Stoinov S, Honiball PJ, Rutgeerts P, Mason D, et al. Certolizumab pegol for the treatment of Crohn's disease. New England journal of medicine. 2007;357(3):228-38.
86. Sandborn WJ, Rutgeerts P, Enns R, Hanauer SB, Colombel JF, Panaccione R, et al. Adalimumab induction therapy for Crohn disease previously treated with infliximab: a randomized trial. Annals of internal medicine. 2007;146(12):829-38.
87. Sandborn WJ, Feagan BG, Fedorak RN, Scherl E, Fleisher MR, Katz S, et al. A randomized trial of Ustekinumab, a human interleukin-12/23 monoclonal antibody, in patients with moderate-to-severe Crohn's disease. Gastroenterology. 2008;135(4):1130-41.
88. Sandborn WJ, Schreiber S, Feagan BG, Rutgeerts P, Younes ZH, Bloomfield R, et al. Certolizumab pegol for active Crohn's disease: a placebo-controlled, randomized trial. Clinical gastroenterology and hepatology. 2011;9(8):670-8.e3.
89. Sandborn WJ, Gasink C, Gao LL, Blank MA, Johanns J, Guzzo C, et al. Ustekinumab induction and maintenance therapy in refractory Crohn's disease. New England journal of medicine. 2012;367(16):1519-28.
90. Sandborn WJ, Colombel JF, Sands BE, Rutgeerts P, Targan SR, Panaccione R, et al. Abatacept for Crohn's disease and ulcerative colitis. Gastroenterology. 2012;143(1):62-9.e4.
91. Sandborn WJ, Feagan BG, Rutgeerts P, Hanauer S, Colombel JF, Sands BE, et al. Vedolizumab as induction and maintenance therapy for Crohn's disease. New England journal of medicine. 2013;369(8):711-21.
92. Sands BE, Sandborn WJ, Van Assche G, Lukas M, Xu J, James A, et al. Vedolizumab as Induction and Maintenance Therapy for Crohn's Disease in Patients Na�ve to or Who Have Failed Tumor Necrosis Factor Antagonist Therapy. Inflammatory bowel diseases. 2017;23(1):97-106.
93. Sands BE, Van Assche G, Tudor D, Akhundova-Unadkat G, Curtis RI, Tan T. Vedolizumab in Combination With Corticosteroids for Induction Therapy in Crohn's Disease: a Post Hoc Analysis of GEMINI 2 and 3. Inflammatory bowel diseases. 2019;25(8):1375-82.
94. Sandborn WJ, Feagan BG, Loftus EV, Peyrin-Biroulet L, Van Assche G, D'Haens G, et al. Efficacy and Safety of Upadacitinib in a Randomized Trial of Patients With Crohn's Disease. Gastroenterology. 2020;158(8):2123-38.e8.

95. Sandborn WJ, D'Haens GR, Reinisch W, Panes J, Chan D, Gonzalez S, et al. Guselkumab for the Treatment of Crohn's Disease: Induction Results From the Phase 2 GALAXI-1 Study. Gastroenterology. 2022;162(6):1650-64 e8.
96. Sandborn WJ, Pan�s J, Danese S, Sharafali Z, Hassanali A, Jacob-Moffatt R, et al. Etrolizumab as induction and maintenance therapy in patients with moderately to severely active Crohn's disease (BERGAMOT): a randomised, placebo-controlled, double-blind, phase 3 trial. The lancet. Gastroenterology & hepatology. 2023;8(1):43-55.
97. Sands BE, Anderson FH, Bernstein CN, Chey WY, Feagan BG, Fedorak RN, et al. Infliximab maintenance therapy for fistulizing Crohn's disease. New England journal of medicine. 2004;350(9):876-85.
98. Sands BE, Kozarek R, Spainhour J, Barish CF, Becker S, Goldberg L, et al. Safety and tolerability of concurrent natalizumab treatment for patients with Crohn's disease not in remission while receiving infliximab. Inflammatory bowel diseases. 2007;13(1):2-11.
99. Sands BE, Jacobson EW, Sylwestrowicz T, Younes Z, Dryden G, Fedorak R, et al. Randomized, double-blind, placebo-controlled trial of the oral interleukin-12/23 inhibitor apilimod mesylate for treatment of active Crohn's disease. Inflammatory bowel diseases. 2010;16(7):1209-18.
100. Sands BE, Feagan BG, Rutgeerts P, Colombel JF, Sandborn WJ, Sy R, et al. Effects of vedolizumab induction therapy for patients with Crohn's disease in whom tumor necrosis factor antagonist treatment failed. Gastroenterology. 2014;147(3):618-27.e3.
101. Sands BE, Feagan BG, Sandborn WJ, Schreiber S, Peyrin-Biroulet L, Fr�d�ric Colombel J, et al. Mongersen (GED-0301) for Active Crohn's Disease: results of a Phase 3 Study. American journal of gastroenterology. 2020;115(5):738-45.
102. Sands BE, Irving PM, Hoops T, Izanec JL, Gao LL, Gasink C, et al. Ustekinumab versus adalimumab for induction and maintenance therapy in biologic-naive patients with moderately to severely active Crohn's disease: a multicentre, randomised, double-blind, parallel-group, phase 3b trial. Lancet (london, england). 2022;399(10342):2200-11.
103. Stack WA, Mann SD, Roy AJ, Heath P, Sopwith M, Freeman J, et al. Randomised controlled trial of CDP571 antibody to tumour necrosis factor-alpha in Crohn's disease. Lancet (london, england). 1997;349(9051):521-4.
104. Steenholdt C, Brynskov J, Thomsen O, Munck LK, Fallingborg J, Christensen LA, et al. Individualised therapy is more cost-effective than dose intensification in patients with Crohn's disease who lose response to anti-TNF treatment: a randomised, controlled trial. Gut. 2014;63(6):919-27.
105. Sutherland LR, Martin F, Bailey RJ, Fedorak RN, Poleski M, Dallaire C, et al. A randomized, placebo-controlled, double-blind trial of mesalamine in the maintenance of remission of Crohn's disease. The Canadian Mesalamine for Remission of Crohn's Disease Study Group. Gastroenterology. 1997;112(4):1069-77.
106. Targan SR, Hanauer SB, van Deventer SJ, Mayer L, Present DH, Braakman T, et al. A short-term study of chimeric monoclonal antibody cA2 to tumor necrosis factor alpha for Crohn's disease. Crohn's Disease cA2 Study Group. N Engl J Med. 1997;337(15):1029-35.
107. Targan SR, Feagan BG, Fedorak RN, Lashner BA, Panaccione R, Present DH, et al. Natalizumab for the treatment of active Crohn's disease: results of the ENCORE Trial. Gastroenterology. 2007;132(5):1672-83.
108. Targan SR, Feagan B, Vermeire S, Panaccione R, Melmed GY, Landers C, et al. A Randomized, Double-Blind, Placebo-Controlled Phase 2 Study of Brodalumab in Patients With Moderate-to-Severe Crohn's Disease. American journal of gastroenterology. 2016;111(11):1599-607.
109. Thomsen OO, Cortot A, Jewell D, Wright JP, Winter T, Veloso FT, et al. A comparison of budesonide and mesalamine for active Crohn's disease. New England journal of medicine. 1998;339(6):370-4.
110. Thomson AB, Wright JP, Vatn M, Bailey RJ, Rachmilewitz D, Adler M, et al. Mesalazine (Mesasal/Claversal) 1.5 g b.d. vs. placebo in the maintenance of remission of patients with Crohn's disease. Alimentary pharmacology & therapeutics. 1995;9(6):673-83.
111. Tremaine WJ, Schroeder KW, Harrison JM, Zinsmeister AR. A randomized, double-blind, placebo-controlled trial of the oral mesalamine (5-ASA) preparation, Asacol, in the treatment of symptomatic Crohn's colitis and ileocolitis. Journal of clinical gastroenterology. 1994;19(4):278-82.

112. Tremaine WJ, Hanauer SB, Katz S, Winston BD, Levine JG, Persson T, et al. Budesonide CIR capsules (once or twice daily divided-dose) in active Crohn's disease: a randomized placebo-controlled study in the United States. American journal of gastroenterology. 2002;97(7):1748-54.
113. Tromm A, Bungani I, Tomsov� E, Tulassay Z, Luk� M, Kykal J, et al. Budesonide 9 mg is at least as effective as mesalamine 4.5 g in patients with mildly to moderately active Crohn's disease. Gastroenterology. 2011;140(2):425-34.e1; quiz e13.
114. Van Assche G, Vermeire S, Ballet V, Gabriels F, Noman M, D'Haens G, et al. Switch to adalimumab in patients with Crohn's disease controlled by maintenance infliximab: prospective randomised SWITCH trial. Gut. 2012;61(2):229-34.
115. van Linschoten RCA, Jansen FM, Pauwels RWM, Smits LJT, Atsma F, Kievit W, et al. Increased versus conventional adalimumab dose interval for patients with Crohn's disease in stable remission (LADI): a pragmatic, open-label, non-inferiority, randomised controlled trial. The lancet. Gastroenterology & hepatology. 2023;8(4):343-55.
116. Vermeire S, D'Haens G, Baert F, Danese S, Kobayashi T, Loftus EV, et al. Efficacy and Safety of Subcutaneous Vedolizumab in Patients With Moderately to Severely Active Crohn's Disease: results From the VISIBLE 2 Randomised Trial. Journal of Crohn's & colitis. 2022;16(1):27-38.
117. Watanabe M, Hibi T, Lomax KG, Paulson SK, Chao J, Alam MS, et al. Adalimumab for the induction and maintenance of clinical remission in Japanese patients with Crohn's disease. Journal of Crohn's & colitis. 2012;6(2):160-73.
118. Watanabe K, Motoya S, Ogata H, Kanai T, Matsui T, Suzuki Y, et al. Effects of vedolizumab in Japanese patients with Crohn's disease: a prospective, multicenter, randomized, placebo-controlled Phase 3 trial with exploratory analyses. Journal of gastroenterology. 2020;55(3):291-306.
119. Wenckert A, Kristensen M, Eklund AE, Barany F, Jarnum S, Worning H, et al. The long-term prophylactic effect of salazosulphapyridine (Salazopyrin) in primarily resected patients with Crohn's disease. A controlled double-blind trial. Scandinavian journal of gastroenterology. 1978;13(2):161-7.
120. Wenzl HH, Primas C, Novacek G, Teml A, �fferlbauer-Ernst A, H�genauer C, et al. Withdrawal of long-term maintenance treatment with azathioprine tends to increase relapse risk in patients with Crohn's disease. Digestive diseases and sciences. 2015;60(5):1414-23.
121. Ye BD, Pesegova M, Alexeeva O, Osipenko M, Lahat A, Dorofeyev A, et al. Efficacy and safety of biosimilar CT-P13 compared with originator infliximab in patients with active Crohn's disease: an international, randomised, double-blind, phase 3 non-inferiority study. Lancet (london, england). 2019;393(10182):1699-707.
122. Yokoyama T, Ohta A, Motoya S, Takazoe M, Yajima T, Date M, et al. Efficacy and Safety of Oral Budesonide in Patients with Active Crohn's Disease in Japan: a Multicenter, Double-Blind, Randomized, Parallel-Group Phase 3 Study. Inflammatory intestinal diseases. 2018;2(3):154-62.
123. Zhang Y, Xia JJ, Xiao P, Zhao Y, Ye LN, Li XL, et al. Standard-dose versus low-dose azathioprine in the treatment of Crohn's disease: a prospective randomized study. Journal of digestive diseases. 2016;17(11):747-55.

6) Risk of bias assessments

Study	D1	D2	D3	D4	D5	Overall
Arber 1995	-	+	-	+	-	-
Ardizzone 2003	-	+	+	+	-	-
Ardizzone 2004	-	-	X	-	-	X
Bar-Meir 1998	-	+	+	+	-	-
Brignola 1995	+	+	+	+	-	-
Campieri 1997	-	+	-	+	-	-
Candy 1995	-	+	+	+	-	-
Caprilli 2003	+	X	X	+	-	X
Chen 2020	+	+	+	-	+	-
Colombel 2007	+	+	X	+	-	X
Colombel 2010	+	+	-	+	+	-
Cosnes 2013	+	-	-	+	+	-
de Franchis 1997	+	-	-	+	-	-
de Jong 2007	-	+	+	+	+	+
D'Haens 2008	-	-	-	-	+	-
D'Haens 2022a	+	+	+	+	+	+
D'Haens 2022b	+	+	+	+	+	+
D'Haens 2023	+	+	-	+	+	-
Dignass 2014	+	+	+	+	X	X
Ewe 1993	-	+	+	+	-	-
Ewe 1999	-	+	-	+	-	-
Feagan 1995	-	+	X	+	-	X
Feagan 2000	-	+	+	+	-	-
Feagan 2016 (IM-UNITI)	+	+	+	+	+	+
Feagan 2016 (UNITI-1)	+	+	+	+	+	+
Feagan 2016 (UNITI-2)	+	+	+	+	+	+
Ferguson 1998	+	+	+	+	-	-
Ferrante 2015	+	X	X	-	+	X
Ferrante 2022	+	-	-	+	+	-
Fukushima 2018	-	-	-	-	-	X
Gelbmann 2001	-	-	-	+	-	X
Gendre 1993	-	+	-	+	-	-
Ghosh 2003	+	+	-	+	-	-
Gordon 2001	+	+	+	+	-	-
Green 2001	-	+	-	+	-	-
Greenberg 1994	-	+	X	+	-	X
Gross 1996	-	+	+	+	-	-
Gross 1998	-	-	-	+	-	-
Hanauer 2002	+	+	-	+	+	-
Hanauer 2004	+	+	?	?	-	-
Hanauer 2005	-	+	-	+	-	-
Hanauer 2006	+	+	+	+	-	-
Hanauer 2021	+	+	+	+	+	+
Hellers 1999	+	+	-	+	-	-
Hisamatsu 2019	+	+	+	-	+	-
Hueber 2012	+	+	+	+	+	+
International Mesalazine Study Group 1990	+	X	+	+	-	X
Jørgensen 2017	+	+	+	+	+	+
Lémann 2005	+	+	+	+	-	-
Lémann 2006	+	+	+	+	-	-
Lennard-Jones 1977	-	+	?	?	-	-
Lochs 2000	+	+	+	+	-	-
Löfberg 1996	-	+	+	+	-	-
Loftus 2023	+	+	+	+	+	+
López-Sanromán 2017	+	+	-	-	+	-
Mahida 1990	+	+	-	+	-	-
Mantzaris 2003	-	+	+	+	-	-
Mantzaris 2009	-	+	+	+	-	-
Martin 1990	-	-	-	+	-	-

Study	D1	D2	D3	D4	D5	Overall
Maté-Jiménez 2000	-	+	-	-	-	X
Matsumoto 2016	+	+	-	-	-	-
McLeod 1995	+	+	?	?	-	-
Modigliani 1996	-	+	+	+	-	-
Mowat 2016	+	+	-	+	+	-
Oren 1997	-	+	+	+	-	-
Orlando 2020	+	+	-	+	-	-
Panés 2017	+	+	+	+	X	X
Prantera 1992	+	+	-	+	-	-
Prantera 1999	+	+	-	+	-	-
Regueiro 2016	-	+	+	+	+	-
Reinisch 2008	+	+	+	+	-	-
Reinisch 2010	+	+	-	+	+	-
Reinshagen 2007	+	+	+	-	-	-
Rutgeerts 1994	+	+	X	+	-	X
Rutgeerts 1999	-	+	-	+	-	-
Rutgeerts 2012	-	+	-	+	+	-
Sandborn 1999	+	+	-	+	-	-
Sandborn 2001	+	-	X	+	-	X
Sandborn 2004	-	+	-	+	-	-
Sandborn 2005 (ENACT-1)	+	+	+	+	-	-
Sandborn 2005 (ENACT-2)	+	-	+	+	-	-
Sandborn 2007a	+	+	+	+	-	-
Sandborn 2007b	+	+	+	+	+	+
Sandborn 2007c	+	+	+	+	+	+
Sandborn 2008	+	+	-	+	X	X
Sandborn 2011	+	+	+	+	-	-
Sandborn 2012a	+	+	-	+	+	-
Sandborn 2012b	+	+	X	+	+	X
Sandborn 2013b	+	+	-	+	+	-
Sandborn 2020	+	+	+	+	+	+
Sandborn 2022	-	-	+	+	+	-
Sandborn 2023	+	+	+	+	+	+
Sands 2004	-	+	?	?	+	-
Sands 2007	-	+	+	+	-	-
Sands 2010	+	+	+	+	-	-
Sands 2014	+	+	-	+	+	-
Sands 2020	+	+	+	+	X	X
Sands 2022	+	+	-	+	+	-
Stack 1997	+	+	+	+	-	-
Steenholdt 2014	+	X	-	+	+	X
Sutherland 1997	+	-	+	+	-	-
Targan 1997	+	+	+	+	-	-
Targan 2007	+	+	+	+	-	-
Targan 2016	-	+	X	+	-	X
Thomsen 1998	+	+	+	+	-	-
Thomson 1995	-	+	-	+	-	-
Tremaine 1994	-	+	+	+	-	-
Tremaine 2002	-	+	+	+	-	-
Tromm 2011	-	+	X	+	-	X
Van Assche 2012	+	-	?	?	-	-
van Linschoten 2023	+	-	+	-	+	-
Vermeire 2022	+	+	+	+	+	+
Watanabe 2012	-	+	+	+	+	-
Watanabe 2020	+	+	-	+	+	-
Wenckert 1978	-	+	?	?	-	-
Wenzl 2015	+	+	+	+	-	-
Ye 2019	+	+	+	+	+	+
Yokoyama 2018	+	+	-	+	+	-
Zhang 2016	-	-	+	+	-	-

Domains:
D1: Bias arising from the randomization process.
D2: Bias due to deviations from intended intervention.
D3: Bias due to missing outcome data.
D4: Bias in measurement of the outcome.
D5: Bias in selection of the reported result.

Judgement: X High; - Some concerns; + Low; ? No information

No information means not applicable because risk of bias (domains 3 and 4: missing outcome data and measurement of the outcome) was only scored for efficacy outcomes, and no efficacy outcomes were extracted for 6 studies.
Some articles reported on multiple studies for which the risk of bias was the same: D'Haens 2022a (2 studies), Loftus 2023 (3 studies), Panés (2 studies), Watanabe 2012 (2 studies).

7) Studies included in network meta-analyses

Induction remission

Immunomodulator naive

	Author	Year	Study name	Intervention	Steroid free	Percentage Biological treated	Follow-up (weeks)	N	N event
1	Colombel	2010	SONIC 2010	Infliximab 5 mg/kg	Yes	0	10	169	63
2	Colombel	2010	SONIC 2010	Azathioprine 2-2.5 mg/kg	Yes	0	10	170	41
3	Colombel	2010	SONIC 2010	Infliximab + Azathioprine 5 mg/kg + 2-2.5 mg/kg	Yes	0	10	169	79
4	D'Haens	2008	D'Haens 2008	Infliximab + Azathioprine 5 mg/kg + 2-2.5 mg/kg	Yes	0	14	65	42
5	D'Haens	2008	D'Haens 2008	Usual care (budesonide or methylprednisolone)	Yes	0	14	64	20
6	Ardizzone	2003	Ardizzone 2003	Methotrexate 25 mg	Yes	14.8	13	27	12
7	Ardizzone	2003	Ardizzone 2003	Azathioprine 2-2.5 mg/kg	Yes	7.4	13	27	9
8	Feagan	1995	Feagan 1995	Methotrexate 25 mg	Yes	0	16	94	37
9	Feagan	1995	Feagan 1995	Placebo	Yes	0	16	47	9

Biologically naive

	Author	Year	Study name	Intervention	Steroid free	Percentage Biological treated	Follow-up (weeks)	N	N event
1	Hanauer	2006	CLASSIC-I 2006	Adalimumab 40 mg	No	0	4	74	13
2	Hanauer	2006	CLASSIC-I 2006	Adalimumab 80 mg	No	0	4	75	18
3	Hanauer	2006	CLASSIC-I 2006	Adalimumab 160 mg	No	0	4	76	27
4	Hanauer	2006	CLASSIC-I 2006	Placebo	No	0	4	74	9
5	Matsumoto	2016	DIAMOND 2016	Adalimumab 160 mg	No	0	26	85	61
6	Matsumoto	2016	DIAMOND 2016	Adalimumab + Azathioprine 160 mg + 25-50 mg	No	0	26	91	62
7	Targan	2007	ENCORE 2007	Natalizumab 300 mg	No	48	12	259	98
8	Targan	2007	ENCORE 2007	Placebo	No	48	12	250	63
9	Sandborn	2007	PRECiSE 1 2007	Certolizumab pegol 400 mg	No	28	4	329	63

**10**	Sandborn	2007	PRECiSE 1 2007	Placebo	No	28	4	326	36
**11**	Sands	2022	SEAVUE 2022	Ustekinumab 6 mg/kg	No	0	8	191	96
**12**	Sands	2022	SEAVUE 2022	Adalimumab 160 mg	No	0	8	195	94
**13**	D'Haens	2022	SERENE CD 2022	Adalimumab (high induction regimen)	Yes	17	12	155	82
**14**	D'Haens	2022	SERENE CD 2022	Adalimumab 160 mg	Yes	17	12	100	48
**15**	Colombel	2010	SONIC 2010	Infliximab 5 mg/kg	Yes	0	10	169	63
**16**	Colombel	2010	SONIC 2010	Azathioprine 2-2.5 mg/kg	Yes	0	10	170	41
**17**	Colombel	2010	SONIC 2010	Infliximab + Azathioprine 5 mg/kg + 2-2.5 mg/kg	Yes	0	10	169	79
**18**	Hanauer	2021	VOLTAIRE-CD 2021	BI 695501 160 mg	Unclear	9	12	68	50
**19**	Hanauer	2021	VOLTAIRE-CD 2021	Adalimumab 160 mg	Unclear	9	12	72	49
**20**	Tromm	2011	Tromm 2011	Budesonide 3 mg	Yes	NA	8	78	56
**21**	Tromm	2011	Tromm 2011	Budesonide 9 mg	Yes	NA	8	76	51
**22**	Tromm	2011	Tromm 2011	5-aminosalicylic acid 1500 mg	Yes	NA	8	153	95
**23**	Watanabe	2012	Watanabe 2012	Adalimumab 160 mg	No	0	4	14	6
**24**	Watanabe	2012	Watanabe 2012	Adalimumab 80 mg	No	0	4	14	4
**25**	Watanabe	2012	Watanabe 2012	Placebo	No	0	4	10	2
**26**	D'Haens	2008	D'Haens 2008	Infliximab + Azathioprine 5 mg/kg + 2-2.5 mg/kg	Yes	0	14	65	42
**27**	D'Haens	2008	D'Haens 2008	Budesonide 9 mg	Yes	0	14	64	20
**28**	Hueber	2012	Hueber 2012	Secukinumab 10 mg/kg	No	12	10	39	5
**29**	Hueber	2012	Hueber 2012	Placebo	No	12	10	20	4
**30**	Dignass	2014	Dignass 2014	Budesonide 3 mg	No	NA	8	233	169
**31**	Dignass	2014	Dignass 2014	Budesonide 9 mg	No	NA	8	238	159
**32**	Yokoyama	2018	Yokoyama 2018	Budesonide 9 mg	No	13	8	56	17
**33**	Yokoyama	2018	Yokoyama 2018	5-aminosalicylic acid 500-1000 mg	Yes	13	8	56	14
**34**	Ye	2019	Ye 2019	CT-P13 5 mg/kg	No	0	14	111	59

35	Ye	2019	Ye 2019	Infliximab 5 mg/kg	No	0	14	109	60
36	Sands	2020	Sands 2020	Mongersen 160 mg	No	0	12	210	73
37	Sands	2020	Sands 2020	Placebo	No	0	12	70	24
38	Ardizzone	2003	Ardizzone 2003	Methotrexate 25 mg	Yes	NA	13	27	12
39	Ardizzone	2003	Ardizzone 2003	Azathioprine 2-2.5 mg/kg	Yes	NA	13	27	9
40	Feagan	1995	Feagan 1995	Methotrexate 25 mg	Yes	0	16	94	37
41	Feagan	1995	Feagan 1995	Placebo	Yes	0	16	47	9
42	Gelbmann	2001	Gelbmann 2001	Budesonide 2 mg	No	0	6	34	13
43	Gelbmann	2001	Gelbmann 2001	Budesonide 3 mg	No	0	6	27	18
44	Gelbmann	2001	Gelbmann 2001	Budesonide 6 mg	No	0	6	32	23
45	Ghosh	2003	Ghosh 2003	Natalizumab 3 mg/kg	Yes	0	12	68	19
46	Ghosh	2003	Ghosh 2003	Natalizumab 6 mg/kg	Yes	0	12	66	28
47	Ghosh	2003	Ghosh 2003	Natalizumab 12 mg/kg	Yes	0	12	51	20
48	Ghosh	2003	Ghosh 2003	Placebo	Yes	0	12	63	17
49	Gordon	2001	Gordon 2001	Natalizumab 3 mg/kg	No	0	2	18	7
50	Gordon	2001	Gordon 2001	Placebo	No	0	2	12	1
51	Greenberg	1994	Greenberg 1994	Budesonide 1.5 mg	Yes	0	8	67	22
52	Greenberg	1994	Greenberg 1994	Budesonide 4.5 mg	Yes	0	8	61	31
53	Greenberg	1994	Greenberg 1994	Budesonide 7.5 mg	Yes	0	8	64	28
54	Greenberg	1994	Greenberg 1994	Placebo	Yes	0	8	66	13
55	Martin	1990	Martin 1990	5-aminosalicylic acid 1000 mg	No	0	12	19	9
56	Martin	1990	Martin 1990	Prednisolone 40 mg	No	0	12	26	12
57	Reinshagen	2007	Reinshagen 2007	Azathioprine 2.5 mg/kg (dose adapted)	Yes	NA	16	25	11
58	Reinshagen	2007	Reinshagen 2007	Azathioprine 2-2.5 mg/kg	Yes	NA	16	32	14
59	Rutgeerts	1994	Rutgeerts 1994	Budesonide 9 mg	No	0	10	86	46
60	Rutgeerts	1994	Rutgeerts 1994	Prednisolone 40 mg	No	0	10	86	57
61	Sandborn	1999	Sandborn 1999	Azathioprine 40 mg/kg	Yes	0	12	51	11
62	Sandborn	1999	Sandborn 1999	Azathioprine 2-2.5 mg/kg	Yes	0	12	45	10

63	Sandborn	2004	Sandborn 2004	CDP571 10 mg/kg	No	23	12	263	66
64	Sandborn	2004	Sandborn 2004	Placebo	No	23	12	132	33
65	Sandborn	2011	Sandborn 2011	Certolizumab pegol 400 mg	No	0	6	215	68
66	Sandborn	2011	Sandborn 2011	Placebo	No	0	6	209	53
67	Thomsen	1998	Thomsen 1998	Budesonide 9 mg	No	0	12	91	58
68	Thomsen	1998	Thomsen 1998	5-aminosalicylic acid 2000 mg	Yes	0	12	83	35
69	Tremaine	2002	Tremaine 2002	Budesonide 9 mg	No	0	8	79	37
70	Tremaine	2002	Tremaine 2002	Budesonide 4.5 mg	No	0	8	78	41
71	Tremaine	2002	Tremaine 2002	Placebo	Yes	0	8	40	13
72	Chen	2020	Chen 2020	Adalimumab 160 mg	No	0	4	102	38
73	Chen	2020	Chen 2020	Placebo	No	0	4	103	7
74	Loftus	2023	Loftus 2023	Upadacitinib 45 mg	No	0	12	189	103
75	Loftus	2023	Loftus 2023	Placebo	No	0	12	98	39
76	Oren	1997	Oren 1997	Methotrexate 12.5 mg	Yes	0	12	26	5
77	Oren	1997	Oren 1997	Azathioprine 2-2.5 mg/kg	Yes	0	12	32	5
78	Oren	1997	Oren 1997	Placebo	Yes	0	12	26	6

Biologically exposed

	Author	Year	Study name	Intervention	Steroid free	Percentage Biological treated	Follow Up (weeks)	N	N event
1	Sandborn	2023	BERGAMOT 2023	Etrolizumab 105 mg	No	NA	14	143	49
2	Sandborn	2023	BERGAMOT 2023	Etrolizumab 210 mg	No	NA	14	145	56
3	Sandborn	2023	BERGAMOT 2023	Placebo	No	NA	14	96	35
4	Sandborn	2012	CERTIFI 2012	Ustekinumab 1 mg/kg	No	100	8	131	23
5	Sandborn	2012	CERTIFI 2012	Ustekinumab 3 mg/kg	No	100	8	132	24
6	Sandborn	2012	CERTIFI 2012	Ustekinumab 6 mg/kg	No	100	8	131	24
7	Sandborn	2012	CERTIFI 2012	Placebo	No	100	8	132	14
8	Sands	2019	GEMINI 3 2014	Vedolizumab 300 mg	Yes	76	10	97	22
9	Sands	2019	GEMINI 3 2014	Placebo	Yes	76	10	97	14

**10**	Feagan	2016	UNITI-1 2016	Ustekinumab 130 mg	No	100	8	245	39
**11**	Feagan	2016	UNITI-1 2016	Ustekinumab 6 mg/kg	No	100	8	249	52
**12**	Feagan	2016	UNITI-1 2016	Placebo	No	100	8	247	18
**13**	Sandborn	2007	Sandborn 2007	Adalimumab 160 mg	Yes	100	4	104	16
**14**	Sandborn	2007	Sandborn 2007	Placebo	Yes	100	4	93	9
**15**	Sandborn	2012	Sandborn 2012	Abatacept 30 mg/kg	No	NA	12	65	5
**16**	Sandborn	2012	Sandborn 2012	Abatacept 10 mg/kg	No	NA	12	128	5
**17**	Sandborn	2012	Sandborn 2012	Abatacept 3 mg/kg	No	NA	12	130	6
**18**	Sandborn	2012	Sandborn 2012	Placebo	No	NA	12	127	8
**19**	Watanabe	2012	Watanabe 2012	Adalimumab 160 mg	No	100	4	19	5
**20**	Watanabe	2012	Watanabe 2012	Adalimumab 80 mg	No	100	4	20	2
**21**	Watanabe	2012	Watanabe 2012	Placebo	No	100	4	13	1
**22**	Watanabe	2020	Watanabe 2020	Vedolizumab 300 mg	No	100	10	61	5
**23**	Watanabe	2020	Watanabe 2020	Placebo	No	100	10	62	6
**24**	Sands	2020	Sands 2020	Mongersen 160 mg	No	100	12	273	37
**25**	Sands	2020	Sands 2020	Placebo	No	100	12	90	16
**26**	D'Haens	2022	D'Haens 2022	Risankizumab 600 mg	No	63	12	336	152
**27**	D'Haens	2022	D'Haens 2022	Risankizumab 1200 mg	No	63	12	339	141
**28**	D'Haens	2022	D'Haens 2022	Placebo	No	63	12	175	43
**29**	D'Haens	2022	D'Haens 2022	Risankizumab 600 mg	No	100	12	191	80
**30**	D'Haens	2022	D'Haens 2022	Risankizumab 1200 mg	No	100	12	191	77
**31**	D'Haens	2022	D'Haens 2022	Placebo	No	100	12	187	37
**32**	PanŽs	2017	PanŽs 2017	Tofacitinib 5 mg	No	100	8	68	26
**33**	PanŽs	2017	PanŽs 2017	Tofacitinib 10 mg	No	100	8	66	28
**34**	PanŽs	2017	PanŽs 2017	Tofacitinib 15 mg	No	100	8	11	4
**35**	PanŽs	2017	PanŽs 2017	Placebo	No	100	8	69	25
**36**	Loftus	2023	Loftus 2023	Upadacitinib 45 mg	No	100	12	161	71
**37**	Loftus	2023	Loftus 2023	Placebo	No	100	12	78	12

**38**	Loftus	2023	Loftus 2023	Upadacitinib 45 mg	Yes	100	12	108	37
**39**	Loftus	2023	Loftus 2023	Placebo	Yes	100	12	60	7

Maintenance

Biologically naive

	Author	Year	Study name	Intervention	Steroid free	Percentage biological treated	Follow up (weeks)	N	N.event
1	Hanauer	2002	ACCENT I 2002	Infliximab 5 mg/kg	No	NA	28	113	44
2	Hanauer	2002	ACCENT I 2002	Infliximab 10 mg/kg	No	NA	28	112	50
3	Hanauer	2002	ACCENT I 2002	Placebo	No	NA	28	110	23
4	Kamm	2011	CHARM 2007	Adalimumab 40 mg	Yes	49	52	206	54
5	Kamm	2011	CHARM 2007	Placebo	Yes	49	52	107	6
6	Sandborn	2007	CLASSIC-II 2007	Adalimumab 40 mg once per 2 weeks	No	0	52	19	15
7	Sandborn	2007	CLASSIC-II 2007	Adalimumab 40 mg	No	0	52	18	15
8	Sandborn	2007	CLASSIC-II 2007	Placebo	No	0	52	18	8
9	Rutgeerts	2012	EXTEND 2012	Adalimumab 40 mg once per 2 weeks	No	52	52	64	21
10	Rutgeerts	2012	EXTEND 2012	Placebo	No	52	52	65	6
11	Sandborn	2007	PRECiSE 1 2007	Certolizumab pegol 400 mg	No	28	26	327	47
12	Sandborn	2007	PRECiSE 1 2007	Placebo	No	28	26	326	32
13	Cosnes	2013	RAPID 2013	Azathioprine 2-2.5 mg/kg	Yes	NA	156	65	28
14	Cosnes	2013	RAPID 2013	Step-up to azathioprine 2-2.5 mg/kg	Yes	NA	156	67	16
15	Sands	2022	SEAVUE 2022	Ustekinumab 6 mg/kg	No	0	52	109	94
16	Sands	2022	SEAVUE 2022	Adalimumab 40 mg once per 2 weeks	No	0	52	117	94
17	Vermeire	2022	VISIBLE 2 2022	Vedolizumab 108 mg	No	0	46	107	52
18	Vermeire	2022	VISIBLE 2 2022	Placebo	No	0	46	63	27
19	D'Haens	2008	D'Haens 2008	Infliximab + Azathioprine 5 mg/kg + 2-2.5 mg/kg	Yes	0	52	65	40
20	D'Haens	2008	D'Haens 2008	Azathioprine 2-2.5 mg/kg	Yes	0	52	64	27

**21**	International Mesalazine Study Group	1990	International Mesalazine Study Group 1990	5-aminosalicylic acid 500-1000 mg	Yes	0	52	101	84
**22**	International Mesalazine Study Group	1990	International Mesalazine Study Group 1990	Placebo	Yes	0	52	105	76
**23**	Arber	1995	Arber 1995	5-aminosalicylic acid 500-1000 mg	Yes	0	52	28	16
**24**	Arber	1995	Arber 1995	Placebo	Yes	0	52	31	12
**25**	Candy	1995	Candy 1995	Azathioprine 2-2.5 mg/kg	Yes	0	53	33	14
**26**	Candy	1995	Candy 1995	Placebo	Yes	0	53	30	2
**27**	de Franchis	1997	de Franchis 1997	5-aminosalicylic acid 1000 mg	Yes	0	40	58	24
**28**	de Franchis	1997	de Franchis 1997	Placebo	Yes	0	40	59	30
**29**	de Jong	2007	de Jong 2007	Budesonide 6 mg	No	NA	52	76	18
**30**	de Jong	2007	de Jong 2007	Budesonide 9 mg	No	NA	52	81	15
**31**	Feagan	2000	Feagan 2000	Methotrexate 15 mg	Yes	0	40	40	26
**32**	Feagan	2000	Feagan 2000	Placebo	Yes	0	40	36	14
**33**	Ferguson	1998	Ferguson 1998	Budesonide 6 mg	No	0	52	22	11
**34**	Ferguson	1998	Ferguson 1998	Budesonide 3 mg	No	0	52	26	14
**35**	Ferguson	1998	Ferguson 1998	Placebo	Yes	0	52	27	11
**36**	Gendre	1993	Gendre 1993	5-aminosalicylic acid 500-1000 mg	Yes	0	104	80	50
**37**	Gendre	1993	Gendre 1993	Placebo	Yes	0	104	81	45
**38**	Green	2001	Green 2001	Budesonide 3-9 mg	No	0	52	66	58
**39**	Green	2001	Green 2001	Budesonide 6 mg	No	0	52	75	64
**40**	Gross	1998	Gross 1998	Budesonide 1 mg	No	0	52	84	28
**41**	Gross	1998	Gross 1998	Placebo	No	0	52	95	33
**42**	Hanauer	2005	Hanauer 2005	Budesonide 6 mg	No	NA	52	55	16
**43**	Hanauer	2005	Hanauer 2005	Placebo	No	NA	52	55	13
**44**	Lšfberg	1996	Lšfberg 1996	Budesonide 6 mg	No	0	52	32	13
**45**	Lšfberg	1996	Lšfberg 1996	Budesonide 3 mg	No	0	52	31	8

**46**	Lšfberg	1996	Lšfberg 1996	Placebo	Yes	0	52	27	10
**47**	Mantzaris	2003	Mantzaris 2003	Budesonide 6 mg	No	0	52	29	13
**48**	Mantzaris	2003	Mantzaris 2003	5-aminosalicylic acid 500-1000 mg	Yes	0	52	28	5
**49**	Mantzaris	2009	Mantzaris 2009	Azathioprine 2-2.5 mg/kg	Yes	0	52	38	32
**50**	Mantzaris	2009	Mantzaris 2009	Budesonide 6 mg	No	0	52	39	25
**51**	Modigliani	1996	Modigliani 1996	5-aminosalicylic acid 2000 mg	Yes	0	52	65	19
**52**	Modigliani	1996	Modigliani 1996	Placebo	Yes	0	52	64	14
**53**	Reinisch	2008	Reinisch 2008	Azathioprine 2-2.5 mg/kg	Yes	NA	30	36	22
**54**	Reinisch	2008	Reinisch 2008	Placebo	Yes	NA	30	22	8
**55**	Sutherland	1997	Sutherland 1997	5-aminosalicylic acid 500-1000 mg	Yes	0	48	118	88
**56**	Sutherland	1997	Sutherland 1997	Placebo	Yes	0	48	128	81
**57**	Thomson	1995	Thomson 1995	5-aminosalicylic acid 1500 mg	Yes	0	52	136	103
**58**	Thomson	1995	Thomson 1995	Placebo	Yes	0	52	148	110
**59**	Loftus	2023	Loftus 2023	Upadacitinib 15 mg	No	0	52	45	21
**60**	Loftus	2023	Loftus 2023	Upadacitinib 30 mg	No	0	52	41	23
**61**	Loftus	2023	Loftus 2023	Placebo	No	0	52	39	10
**62**	D'Haens	2023	D'Haens 2023	Filgotinib 100 mg	No	58	24	32	8
**63**	D'Haens	2023	D'Haens 2023	Filgotinib 200 mg	No	58	24	28	7
**64**	D'Haens	2023	D'Haens 2023	Placebo	No	58	24	18	3

Biologically exposed

	**Author**	**Year**	**Study.name**	**Intervention**	**Steroid.free**	**Percentage biological treated**	**Follow up (weeks)**	**N**	**N event**
**1**	Sandborn	2023	BERGAMOT 2023	Etrolizumab 105 mg	No	NA	52	217	83
**2**	Sandborn	2023	BERGAMOT 2023	Placebo	No	NA	52	217	66
**3**	Ferrante	2022	FORTIFY SS1 2022	Risankizumab 180 mg	No	100	52	157	87

**4**	Ferrante	2022	FORTIFY SS1 2022	Risankizumab 360 mg	No	100	52	141	74
**5**	Ferrante	2022	FORTIFY SS1 2022	Placebo	No	100	52	164	67
**6**	Feagan	2016	IM-UNITI 2016	Ustekinumab (90 mg/12 wk)	Yes	60	44	72	38
**7**	Feagan	2016	IM-UNITI 2016	Ustekinumab 90 mg	Yes	60	44	68	41
**8**	Feagan	2016	IM-UNITI 2016	Placebo	Yes	60	44	73	30
**9**	Vermeire	2022	VISIBLE 2 2022	Vedolizumab 108 mg	No	100	46	168	80
**10**	Vermeire	2022	VISIBLE 2 2022	Placebo	No	100	46	71	19
**11**	Sandborn	2012	Sandborn 2012	Abatacept 10 mg/kg	No	NA	40	42	10
**12**	Sandborn	2012	Sandborn 2012	Placebo	No	NA	40	45	5
**13**	PanŽs	2017	PanŽs 2017	Tofacitinib 5 mg	No	100	26	35	12
**14**	PanŽs	2017	PanŽs 2017	Tofacitinib 10 mg	No	100	26	35	12
**15**	PanŽs	2017	PanŽs 2017	Placebo	No	100	26	27	8
**16**	Loftus	2023	Loftus 2023	Upadacitinib 15 mg	No	100	52	124	42
**17**	Loftus	2023	Loftus 2023	Upadacitinib 30 mg	No	100	52	127	57
**18**	Loftus	2023	Loftus 2023	Placebo	No	100	52	126	15

8) Network plots

Induction remission

Immunomodulator naive

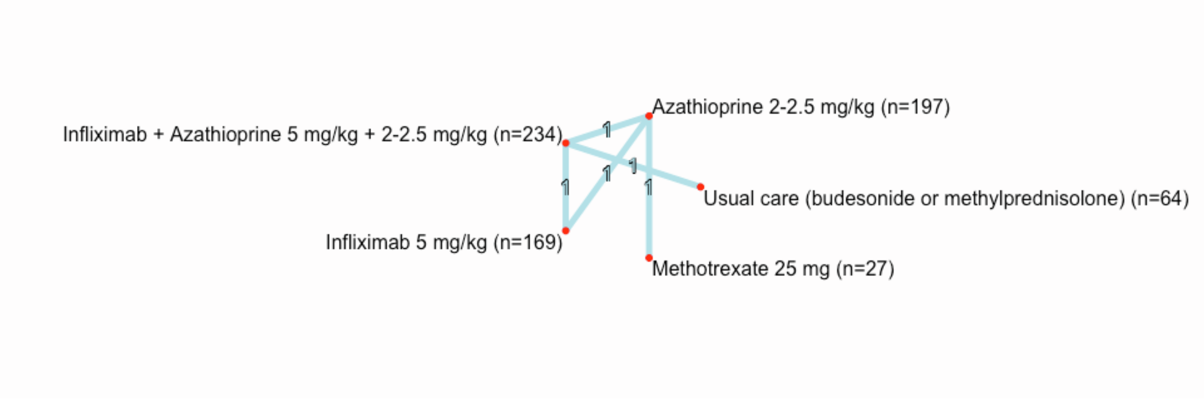

Biologically naive

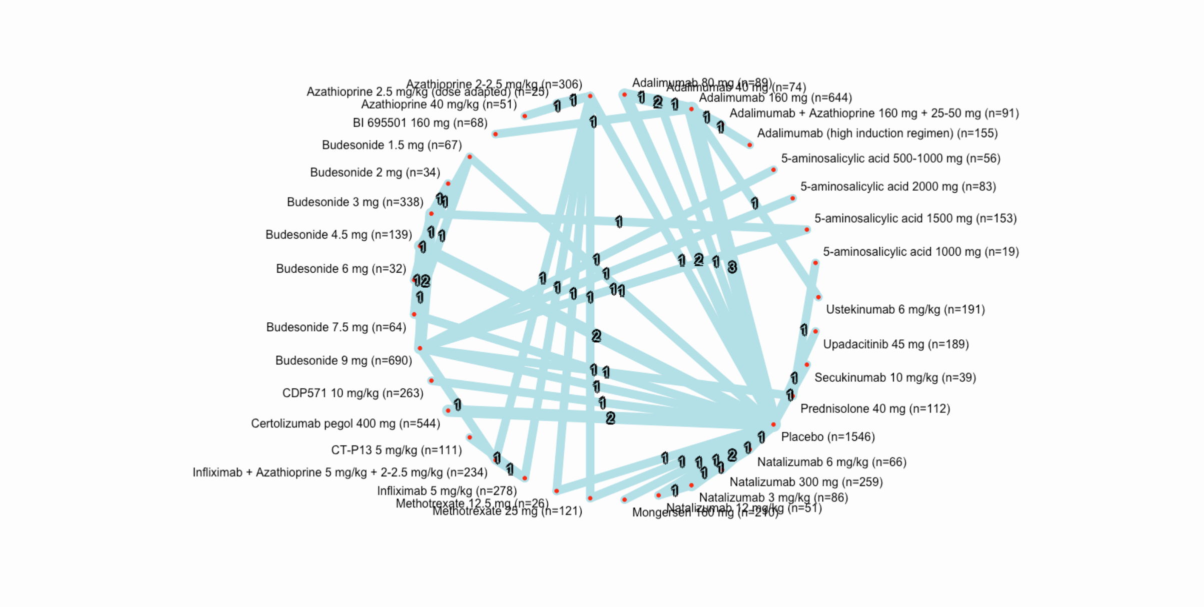

Biologically exposed

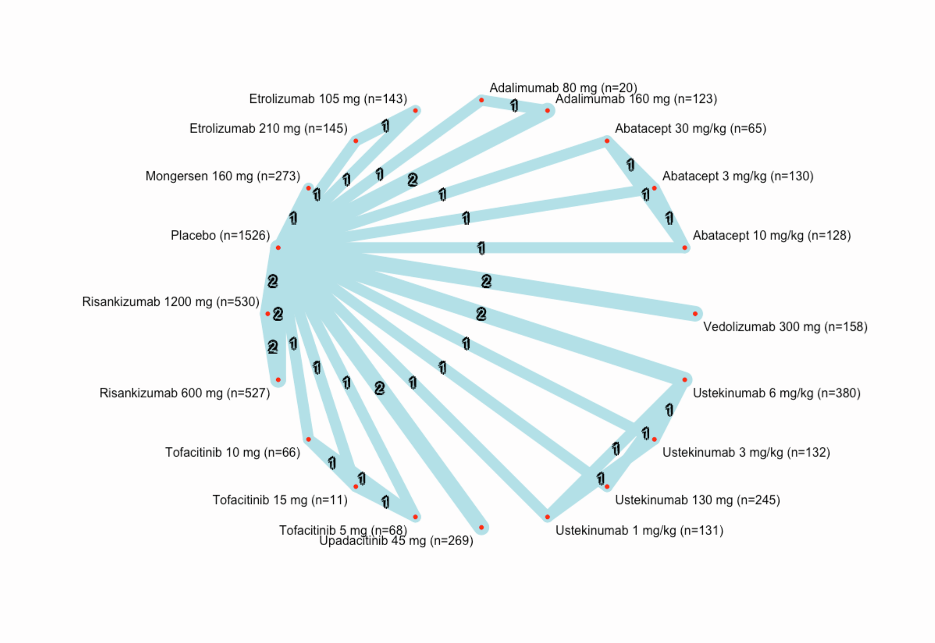

Maintenance

Biologically naive

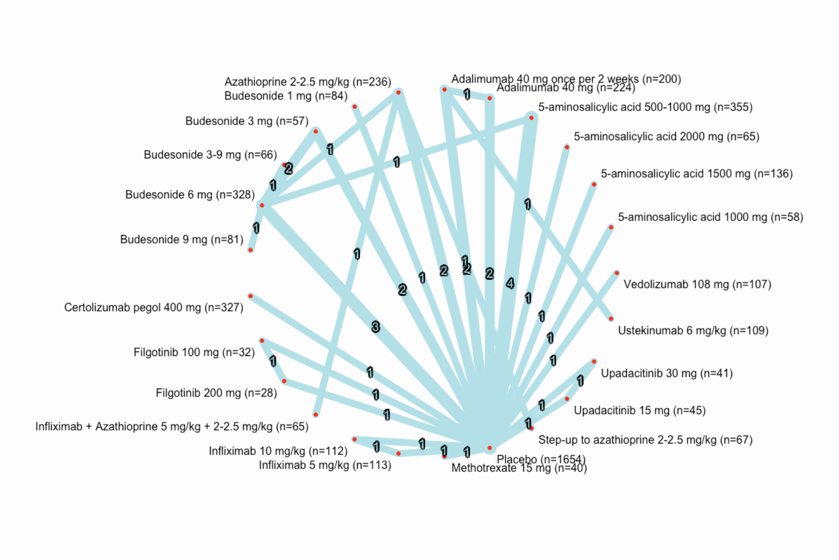

Biologically exposed

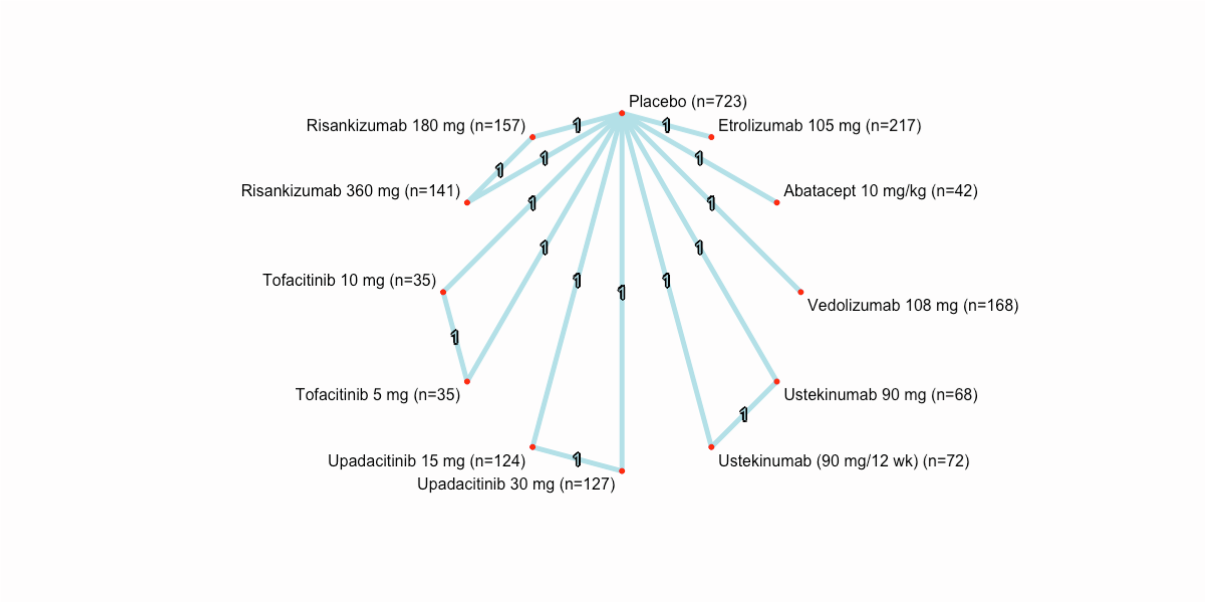

9) League tables

Induction remission

Immunomodulator naive

Placebo					
0.69 [0.24; 2.01]	Usual care (budesonide or methylprednisolone)				
0.65 [0.26; 1.65]	0.94 [0.56; 1.56]	Azathioprine 2-2.5 mg/kg			
0.49 [0.26; 0.92]	0.70 [0.30; 1.65]	0.75 [0.38; 1.48]	Methotrexate 25 mg		
0.42 [0.16; 1.13]	0.61 [0.38; 0.98]	0.65 [0.46; 0.90]	0.86 [0.40; 1.84]	Infliximab 5 mg/kg	
0.33 [0.13; 0.89]	0.48 [0.32; 0.73]	0.52 [0.38; 0.70]	0.69 [0.33; 1.45]	0.80 [0.62; 1.03]	Infliximab + Azathioprine 5 mg/kg + 2-2.5 mg/kg

Biologically naive

Secukinumab 10 mg/kg				.	.	.	.	.	.	.	.	.	.	.	.																	.				
0.64 [0.19; 2.14]	Placebo	.		.					.					.	.																	.				
0.67 [0.17; 2.67]	1.05 [0.54; 2.05]	Budesonide 2 mg	.	.	.	.	.	.	.	.	.	.	.	.	.																	.				
0.64 [0.18; 2.26]	1.00 [0.68; 1.46]	0.95 [0.44; 2.05]	CDP571 10 mg/kg	.	.	.	.	.	.	.	.	.	.	.	.																	.				
0.63 [0.17; 2.33]	0.98 [0.59; 1.64]	0.94 [0.50; 1.76]	0.99 [0.52; 1.87]	5-aminosalicylic acid 2000 mg	.	.	.	.	.	.	.	.	.	.	.																	.				
0.63 [0.18; 2.25]	0.99 [0.66; 1.46]	0.94 [0.43; 2.05]	0.99 [0.57; 1.72]	1.00 [0.53; 1.92]	Mongersen 160 mg	.	.	.	.	.	.	.	.	.	.																	.				
0.55 [0.15; 2.07]	0.86 [0.50; 1.49]	0.82 [0.34; 1.95]	0.86 [0.44; 1.69]	0.87 [0.41; 1.85]	0.87 [0.44; 1.71]	Natalizumab 3 mg/kg	.	.	.	.	.	.	.	.	.																	.				
0.56 [0.12; 2.65]	0.88 [0.33; 2.33]	0.83 [0.26; 2.63]	0.88 [0.31; 2.52]	0.89 [0.31; 2.58]	0.89 [0.31; 2.55]	1.02 [0.33; 3.13]	Methotrexate 12.5 mg	.	.	.	.		.	.	.																	.				
0.49 [0.13; 1.82]	0.77 [0.46; 1.28]	0.73 [0.34; 1.55]	0.77 [0.41; 1.46]	0.78 [0.42; 1.44]	0.78 [0.41; 1.48]	0.89 [0.42; 1.89]	0.88 [0.30; 2.60]	Budesonide 1.5 mg	.	.	.	.	.	.	.																	.				
0.51 [0.12; 2.08]	0.79 [0.38; 1.64]	0.75 [0.33; 1.71]	0.79 [0.35; 1.81]	0.80 [0.40; 1.61]	0.80 [0.35; 1.84]	0.92 [0.37; 2.30]	0.90 [0.28; 2.96]	1.03 [0.46; 2.31]	5-aminosalicylic acid 500-1000 mg	.	.	.	.	.	.																	.				
0.47 [0.13; 1.62]	0.73 [0.54; 0.99]	0.70 [0.33; 1.45]	0.73 [0.45; 1.20]	0.74 [0.41; 1.35]	0.74 [0.45; 1.22]	0.85 [0.45; 1.59]	0.83 [0.30; 2.33]	0.95 [0.53; 1.72]	0.92 [0.42; 2.05]	Upadacitinib 45 mg	.	.	.	.	.																	.				
0.47 [0.10; 2.14]	0.73 [0.29; 1.83]	0.70 [0.25; 1.98]	0.74 [0.27; 1.99]	0.75 [0.29; 1.93]	0.74 [0.27; 2.02]	0.85 [0.29; 2.48]	0.84 [0.24; 2.95]	0.96 [0.35; 2.61]	0.93 [0.31; 2.75]	1.00 [0.38; 2.64]	Azathioprine 40 mg/kg		.	.	.																	.				
0.46 [0.12; 1.68]	0.71 [0.43; 1.17]	0.68 [0.33; 1.37]	0.71 [0.38; 1.34]	0.72 [0.41; 1.27]	0.72 [0.38; 1.36]	0.83 [0.39; 1.74]	0.81 [0.30; 2.20]	0.93 [0.49; 1.77]	0.90 [0.42; 1.94]	0.97 [0.54; 1.75]	0.97 [0.45; 2.09]	Azathioprine 2-2.5 mg/kg	.		.																	.				
0.45 [0.13; 1.54]	0.70 [0.54; 0.91]	0.67 [0.33; 1.37]	0.70 [0.44; 1.12]	0.71 [0.40; 1.26]	0.71 [0.44; 1.14]	0.82 [0.45; 1.50]	0.80 [0.29; 2.21]	0.91 [0.52; 1.62]	0.89 [0.41; 1.93]	0.96 [0.65; 1.43]	0.96 [0.37; 2.48]	0.99 [0.56; 1.73]	Certolizumab pegol 400 mg	.	.																	.				
0.45 [0.11; 1.91]	0.71 [0.32; 1.55]	0.67 [0.27; 1.71]	0.71 [0.30; 1.70]	0.72 [0.32; 1.64]	0.72 [0.30; 1.73]	0.82 [0.32; 2.15]	0.81 [0.25; 2.60]	0.92 [0.38; 2.24]	0.90 [0.34; 2.38]	0.97 [0.42; 2.25]	0.97 [0.36; 2.57]	0.99 [0.54; 1.82]	1.01 [0.44; 2.30]	Azathioprine 2.5 mg/kg (dose adapted)	.																	.				
0.45 [0.12; 1.62]	0.70 [0.45; 1.10]	0.67 [0.38; 1.17]	0.70 [0.39; 1.27]	0.71 [0.48; 1.05]	0.71 [0.39; 1.29]	0.81 [0.40; 1.66]	0.80 [0.28; 2.25]	0.91 [0.52; 1.60]	0.89 [0.46; 1.70]	0.96 [0.56; 1.65]	0.95 [0.38; 2.38]	0.98 [0.59; 1.62]	1.00 [0.59; 1.67]	0.99 [0.45; 2.17]	5-aminosalicylic																	.				

															acid 1500 mg																					
0.43 [0.12; 1.48]	0.67 [0.50; 0.89]	0.63 [0.31; 1.32]	0.67 [0.41; 1.08]	0.68 [0.38; 1.22]	0.68 [0.41; 1.11]	0.78 [0.42; 1.45]	0.76 [0.27; 2.12]	0.87 [0.48; 1.56]	0.84 [0.38; 1.86]	0.91 [0.60; 1.39]	0.91 [0.35; 2.38]	0.94 [0.52; 1.67]	0.95 [0.64; 1.40]	0.94 [0.41; 2.18]	0.95 [0.56; 1.63]	Natalizumab 300 mg	.	.	.													.				
0.41 [0.11; 1.55]	0.65 [0.38; 1.11]	0.62 [0.26; 1.46]	0.65 [0.33; 1.26]	0.66 [0.31; 1.38]	0.66 [0.34; 1.28]	0.75 [0.45; 1.27]	0.74 [0.24; 2.26]	0.84 [0.40; 1.77]	0.82 [0.33; 2.04]	0.89 [0.48; 1.65]	0.88 [0.30; 2.55]	0.91 [0.44; 1.89]	0.92 [0.51; 1.67]	0.91 [0.35; 2.37]	0.93 [0.46; 1.87]	0.97 [0.53; 1.80]	Natalizumab 12 mg/kg	.	.													.				
0.42 [0.12; 1.48]	0.65 [0.44; 0.97]	0.62 [0.36; 1.06]	0.65 [0.38; 1.13]	0.66 [0.48; 0.91]	0.66 [0.38; 1.15]	0.76 [0.38; 1.49]	0.74 [0.27; 2.05]	0.85 [0.50; 1.43]	0.82 [0.44; 1.53]	0.89 [0.54; 1.47]	0.89 [0.36; 2.16]	0.91 [0.58; 1.44]	0.93 [0.58; 1.49]	0.92 [0.43; 1.96]	0.93 [0.75; 1.15]	0.98 [0.60; 1.60]	1.01 [0.51; 1.96]	Budesonide 9 mg	.													.				
0.38 [0.09; 1.51]	0.59 [0.30; 1.17]	0.56 [0.21; 1.46]	0.59 [0.27; 1.30]	0.60 [0.25; 1.41]	0.60 [0.27; 1.32]	0.68 [0.28; 1.65]	0.67 [0.20; 2.23]	0.77 [0.33; 1.80]	0.75 [0.27; 2.04]	0.81 [0.38; 1.71]	0.80 [0.26; 2.52]	0.83 [0.35; 1.93]	0.84 [0.40; 1.75]	0.83 [0.29; 2.36]	0.84 [0.37; 1.91]	0.88 [0.42; 1.87]	0.91 [0.38; 2.18]	0.90 [0.41; 2.00]	Adalimumab 40 mg													.				
0.38 [0.10; 1.42]	0.60 [0.36; 0.99]	0.57 [0.25; 1.32]	0.60 [0.32; 1.13]	0.61 [0.30; 1.25]	0.61 [0.32; 1.15]	0.70 [0.43; 1.13]	0.68 [0.23; 2.05]	0.78 [0.38; 1.59]	0.76 [0.31; 1.84]	0.82 [0.45; 1.47]	0.81 [0.29; 2.32]	0.84 [0.41; 1.71]	0.85 [0.48; 1.50]	0.84 [0.33; 2.15]	0.86 [0.44; 1.68]	0.90 [0.50; 1.61]	0.92 [0.58; 1.47]	0.92 [0.48; 1.75]	1.02 [0.43; 2.38]	Natalizumab 6 mg/kg	.	.	.	.								.				
0.39 [0.11; 1.38]	0.60 [0.39; 0.92]	0.57 [0.34; 0.96]	0.60 [0.34; 1.07]	0.61 [0.43; 0.87]	0.61 [0.34; 1.09]	0.70 [0.35; 1.40]	0.69 [0.25; 1.92]	0.78 [0.46; 1.35]	0.76 [0.40; 1.44]	0.82 [0.49; 1.39]	0.82 [0.33; 2.03]	0.85 [0.52; 1.36]	0.86 [0.52; 1.40]	0.85 [0.39; 1.84]	0.86 [0.70; 1.06]	0.90 [0.54; 1.51]	0.93 [0.47; 1.85]	0.93 [0.80; 1.07]	1.02 [0.46; 2.29]	1.01 [0.52; 1.94]	Budesonide 3 mg	.		.								.				
0.37 [0.10; 1.35]	0.58 [0.36; 0.92]	0.55 [0.27; 1.13]	0.58 [0.32; 1.06]	0.59 [0.33; 1.05]	0.58 [0.32; 1.08]	0.67 [0.33; 1.38]	0.66 [0.23; 1.91]	0.75 [0.47; 1.19]	0.73 [0.33; 1.60]	0.79 [0.45; 1.38]	0.79 [0.29; 2.10]	0.81 [0.44; 1.50]	0.82 [0.48; 1.40]	0.81 [0.34; 1.93]	0.82 [0.49; 1.40]	0.86 [0.50; 1.50]	0.89 [0.44; 1.82]	0.89 [0.55; 1.44]	0.98 [0.43; 2.25]	0.96 [0.48; 1.91]	0.96 [0.58; 1.58]	Budesonide 7.5 mg	.	.								.				
0.36 [0.09; 1.35]	0.56 [0.32; 0.98]	0.53 [0.32; 0.87]	0.56 [0.28; 1.10]	0.57 [0.34; 0.95]	0.57 [0.29; 1.12]	0.65 [0.30; 1.42]	0.64 [0.21; 1.90]	0.73 [0.38; 1.40]	0.71 [0.34; 1.47]	0.76 [0.40; 1.45]	0.76 [0.29; 2.02]	0.78 [0.43; 1.43]	0.80 [0.43; 1.47]	0.79 [0.34; 1.85]	0.80 [0.52; 1.22]	0.84 [0.45; 1.58]	0.86 [0.40; 1.88]	0.86 [0.58; 1.27]	0.95 [0.39; 2.30]	0.93 [0.44; 1.98]	0.93 [0.64; 1.34]	0.97 [0.52; 1.81]	Budesonide 6 mg	.								.				
0.33 [0.08; 1.40]	0.51 [0.23; 1.14]	0.49 [0.20; 1.18]	0.51 [0.21; 1.25]	0.52 [0.24; 1.13]	0.52 [0.21; 1.27]	0.60 [0.22; 1.58]	0.58 [0.17; 2.01]	0.67 [0.28; 1.60]	0.65 [0.25; 1.65]	0.70 [0.30; 1.66]	0.70 [0.22; 2.17]	0.72 [0.31; 1.66]	0.73 [0.31; 1.70]	0.72 [0.26; 2.03]	0.73 [0.35; 1.52]	0.77 [0.33; 1.81]	0.79 [0.30; 2.09]	0.79 [0.39; 1.59]	0.87 [0.30; 2.51]	0.86 [0.33; 2.21]	0.85 [0.42; 1.74]	0.89 [0.38; 2.08]	0.92 [0.41; 2.05]	5-aminosalicylic acid 1000 mg								.				
0.34 [0.09; 1.23]	0.52 [0.32; 0.85]	0.50 [0.27; 0.92]	0.53 [0.28; 0.98]	0.53 [0.35; 0.82]	0.53 [0.28; 0.99]	0.61 [0.29; 1.27]	0.60 [0.21; 1.72]	0.68 [0.38; 1.24]	0.66 [0.34; 1.31]	0.72 [0.41; 1.27]	0.72 [0.28; 1.82]	0.74 [0.43; 1.26]	0.75 [0.43; 1.29]	0.74 [0.33; 1.66]	0.75 [0.53; 1.07]	0.79 [0.45; 1.39]	0.81 [0.39; 1.68]	0.81 [0.61; 1.07]	0.89 [0.38; 2.07]	0.88 [0.44; 1.77]	0.87 [0.64; 1.19]	0.91 [0.52; 1.59]	0.94 [0.58; 1.52]	1.03 [0.54; 1.95]	Prednisolone 40 mg	.	.	.	.	.	.	.				
0.34 [0.10; 1.20]	0.53 [0.37; 0.76]	0.51 [0.27; 0.95]	0.53 [0.32; 0.90]	0.54 [0.34; 0.85]	0.54 [0.32; 0.92]	0.62 [0.32; 1.19]	0.61 [0.22; 1.68]	0.69 [0.45; 1.07]	0.67 [0.34; 1.35]	0.73 [0.45; 1.17]	0.72 [0.29; 1.82]	0.75 [0.45; 1.25]	0.76 [0.49; 1.18]	0.75 [0.34; 1.66]	0.76 [0.52; 1.12]	0.80 [0.50; 1.27]	0.82 [0.43; 1.57]	0.82 [0.59; 1.13]	0.90 [0.42; 1.96]	0.89 [0.48; 1.65]	0.88 [0.62; 1.26]	0.92 [0.63; 1.35]	0.95 [0.57; 1.58]	1.04 [0.48; 2.25]	1.01 [0.66; 1.55]	Budesonide 4.5 mg	.	.	.	.	.	.				
0.33 [0.09; 1.22]	0.51 [0.30; 0.86]	0.48 [0.22; 1.07]	0.51 [0.27; 0.98]	0.52 [0.26; 1.01]	0.52 [0.27; 1.00]	0.59 [0.28; 1.27]	0.58 [0.20; 1.69]	0.66 [0.33; 1.34]	0.64 [0.27; 1.51]	0.70 [0.38; 1.28]	0.69 [0.27; 1.77]	0.71 [0.41; 1.23]	0.72 [0.40; 1.30]	0.72 [0.32; 1.62]	0.73 [0.39; 1.36]	0.76 [0.42; 1.40]	0.79 [0.37; 1.67]	0.78 [0.43; 1.41]	0.86 [0.36; 2.06]	0.85 [0.41; 1.77]	0.84 [0.46; 1.54]	0.88 [0.45; 1.73]	0.91 [0.45; 1.85]	0.99 [0.40; 2.48]	0.97 [0.51; 1.86]	0.96 [0.53; 1.74]	Methotrexate 25 mg	.	.	.	.	.				
0.29 [0.08; 1.13]	0.46 [0.25; 0.84]	0.44 [0.20; 0.93]	0.46 [0.22; 0.94]	0.47 [0.25; 0.87]	0.46 [0.23; 0.95]	0.53 [0.24; 1.20]	0.52 [0.18; 1.52]	0.60 [0.29; 1.22]	0.58 [0.26; 1.31]	0.63 [0.32; 1.23]	0.62 [0.26; 1.51]	0.64 [0.41; 1.00]	0.65 [0.34; 1.26]	0.65 [0.31; 1.37]	0.65 [0.37; 1.17]	0.69 [0.35; 1.34]	0.71 [0.31; 1.59]	0.70 [0.41; 1.21]	0.78 [0.31; 1.94]	0.77 [0.35; 1.68]	0.76 [0.43; 1.33]	0.79 [0.40; 1.59]	0.82 [0.42; 1.60]	0.89 [0.37; 2.17]	0.87 [0.47; 1.60]	0.86 [0.47; 1.57]	0.90 [0.46; 1.76]	CT-P13 5 mg/kg	.		.	.				
0.28 [0.07; 1.07]	0.43 [0.24; 0.78]	0.41 [0.17; 1.01]	0.44 [0.22; 0.88]	0.44 [0.20; 0.97]	0.44 [0.22; 0.90]	0.51 [0.23; 1.13]	0.50 [0.16; 1.56]	0.57 [0.26; 1.23]	0.55 [0.21; 1.41]	0.60 [0.31; 1.16]	0.59 [0.20; 1.76]	0.61 [0.28; 1.32]	0.62 [0.33; 1.18]	0.61 [0.23; 1.64]	0.62 [0.30; 1.31]	0.65 [0.34; 1.26]	0.67 [0.30; 1.50]	0.67 [0.33; 1.36]	0.74 [0.39; 1.39]	0.73 [0.34; 1.58]	0.72 [0.35; 1.49]	0.76 [0.36; 1.60]	0.78 [0.35; 1.76]	0.85 [0.31; 2.31]	0.83 [0.39; 1.78]	0.82 [0.41; 1.63]	0.86 [0.39; 1.89]	0.95 [0.41; 2.21]	Adalimumab 80 mg	.	.	.				
0.28 [0.08; 1.06]	0.44 [0.26; 0.76]	0.42 [0.21; 0.86]	0.44 [0.23; 0.86]	0.45 [0.26; 0.79]	0.45 [0.23; 0.87]	0.51 [0.24; 1.11]	0.50 [0.18; 1.42]	0.58 [0.30; 1.12]	0.56 [0.26; 1.21]	0.60 [0.33; 1.12]	0.60 [0.26; 1.40]	0.62 [0.44; 0.88]	0.63 [0.35; 1.14]	0.62 [0.31; 1.25]	0.63 [0.38; 1.05]	0.66 [0.36; 1.22]	0.68 [0.32; 1.46]	0.68 [0.43; 1.08]	0.75 [0.31; 1.80]	0.74 [0.35; 1.54]	0.73 [0.45; 1.19]	0.77 [0.41; 1.45]	0.79 [0.43; 1.45]	0.86 [0.37; 2.00]	0.84 [0.49; 1.45]	0.83 [0.49; 1.42]	0.87 [0.47; 1.60]	0.97 [0.73; 1.27]	1.02 [0.46; 2.25]	Infliximab 5 mg/kg		.				
0.22 [0.06; 0.81]	0.35 [0.21; 0.56]	0.33 [0.17; 0.64]	0.35 [0.19; 0.64]	0.35 [0.21; 0.58]	0.35 [0.19; 0.66]	0.40 [0.19; 0.84]	0.40 [0.14; 1.09]	0.45 [0.24; 0.84]	0.44 [0.21; 0.91]	0.47 [0.27; 0.84]	0.47 [0.21; 1.08]	0.49 [0.35; 0.67]	0.49 [0.29; 0.85]	0.49 [0.25; 0.97]	0.49 [0.32; 0.77]	0.52 [0.29; 0.92]	0.53 [0.26; 1.10]	0.53 [0.36; 0.78]	0.59 [0.25; 1.36]	0.58 [0.29; 1.16]	0.57 [0.38; 0.87]	0.60 [0.33; 1.08]	0.62 [0.36; 1.08]	0.68 [0.30; 1.51]	0.66 [0.41; 1.06]	0.65 [0.41; 1.04]	0.68 [0.38; 1.21]	0.76 [0.51; 1.12]	0.80 [0.37; 1.71]	0.78 [0.59; 1.04]	Infliximab + Azathioprine 5 mg/kg + 2-2.5 mg/kg	.				
0.19 [0.05; 0.70]	0.29 [0.17; 0.50]	0.28 [0.12; 0.66]	0.29 [0.15; 0.57]	0.30 [0.14; 0.62]	0.30 [0.15; 0.58]	0.34 [0.16; 0.73]	0.33 [0.11; 1.02]	0.38 [0.18; 0.80]	0.37 [0.15; 0.92]	0.40 [0.22; 0.74]	0.40 [0.14; 1.15]	0.41 [0.20; 0.85]	0.42 [0.23; 0.75]	0.41 [0.16; 1.07]	0.42 [0.21; 0.84]	0.44 [0.24; 0.81]	0.45 [0.21; 0.97]	0.45 [0.23; 0.88]	0.50 [0.27; 0.93]	0.49 [0.23; 1.02]	0.49 [0.25; 0.96]	0.51 [0.25; 1.03]	0.52 [0.24; 1.14]	0.57 [0.22; 1.50]	0.56 [0.27; 1.15]	0.55 [0.29; 1.05]	0.57 [0.27; 1.22]	0.64 [0.28; 1.43]	0.67 [0.40; 1.12]	0.66 [0.31; 1.41]	0.84 [0.41; 1.74]	Adalimumab + Azathioprine 160 mg + 25-50 mg				
0.18 [0.05; 0.65]	0.28 [0.17; 0.45]	0.26 [0.12; 0.60]	0.28 [0.15; 0.52]	0.28 [0.14; 0.57]	0.28 [0.15; 0.52]	0.32 [0.16; 0.67]	0.32 [0.11; 0.94]	0.36 [0.18; 0.73]	0.35 [0.15; 0.84]	0.38 [0.21; 0.67]	0.38 [0.13; 1.06]	0.39 [0.19; 0.78]	0.40 [0.23; 0.68]	0.39 [0.16; 0.98]	0.40 [0.20; 0.77]	0.42 [0.24; 0.73]	0.43 [0.21; 0.88]	0.43 [0.23; 0.80]	0.47 [0.26; 0.84]	0.46 [0.23; 0.93]	0.46 [0.24; 0.88]	0.48 [0.25; 0.94]	0.50 [0.24; 1.04]	0.54 [0.21; 1.39]	0.53 [0.27; 1.05]	0.52 [0.29; 0.95]	0.55 [0.27; 1.12]	0.61 [0.28; 1.31]	0.64 [0.40; 1.01]	0.63 [0.30; 1.29]	0.80 [0.40; 1.59]	0.95 [0.75; 1.20]	Adalimumab 160 mg	0.96 [0.5; 1.22]		
0.17 [0.05; 0.64]	0.27 [0.15; 0.46]	0.25 [0.11; 0.60]	0.27 [0.14; 0.52]	0.27 [0.13; 0.57]	0.27 [0.14; 0.53]	0.31 [0.14; 0.67]	0.30 [0.10; 0.93]	0.35 [0.17; 0.73]	0.34 [0.14; 0.84]	0.36 [0.20; 0.68]	0.36 [0.13; 1.05]	0.37 [0.18; 0.78]	0.38 [0.21; 0.69]	0.38 [0.14; 0.97]	0.38 [0.19; 0.77]	0.40 [0.22; 0.74]	0.41 [0.19; 0.88]	0.41 [0.21; 0.80]	0.45 [0.24; 0.85]	0.44 [0.21; 0.93]	0.44 [0.22; 0.88]	0.46 [0.23; 0.94]	0.48 [0.22; 1.04]	0.52 [0.20; 1.37]	0.51 [0.25; 1.05]	0.50 [0.26; 0.96]	0.52 [0.25; 1.11]	0.58 [0.26; 1.31]	0.61 [0.36; 1.03]	0.60 [0.28; 1.29]	0.77 [0.37; 1.59]	0.91 [0.65; 1.27]	0.96 [0.75; 1.22]	Ustekinumab 6 mg/kg	.	.
0.16 [0.04; 0.62]	0.26 [0.15; 0.44]	0.24 [0.10; 0.58]	0.26 [0.13; 0.50]	0.26 [0.12; 0.55]	0.26 [0.13; 0.51]	0.30 [0.14; 0.65]	0.29 [0.10; 0.90]	0.33 [0.16; 0.70]	0.32 [0.13; 0.81]	0.35 [0.19; 0.66]	0.35 [0.12; 1.01]	0.36 [0.17; 0.75]	0.37 [0.20; 0.67]	0.36 [0.14; 0.94]	0.37 [0.18; 0.74]	0.39 [0.21; 0.71]	0.40 [0.18; 0.85]	0.39 [0.20; 0.77]	0.44 [0.23; 0.82]	0.43 [0.20; 0.90]	0.43 [0.21; 0.85]	0.45 [0.22; 0.91]	0.46 [0.21; 1.00]	0.50 [0.19; 1.33]	0.49 [0.24; 1.01]	0.48 [0.25; 0.93]	0.50 [0.24; 1.08]	0.56 [0.25; 1.26]	0.59 [0.35; 0.99]	0.58 [0.27; 1.25]	0.74 [0.36; 1.54]	0.88 [0.62; 1.24]	0.93 [0.72; 1.19]	0.97 [0.68; 1.36]	BI 695501 160 mg	.
0.16 [0.04; 0.61]	0.25 [0.14; 0.44]	0.24 [0.10; 0.57]	0.25 [0.13; 0.50]	0.26 [0.12; 0.55]	0.26 [0.13; 0.51]	0.29 [0.13; 0.64]	0.29 [0.09; 0.89]	0.33 [0.15; 0.70]	0.32 [0.13; 0.80]	0.34 [0.18; 0.65]	0.34 [0.12; 1.00]	0.35 [0.17; 0.75]	0.36 [0.19; 0.66]	0.36 [0.14; 0.93]	0.36 [0.18; 0.74]	0.38 [0.20; 0.71]	0.39 [0.18; 0.85]	0.39 [0.19; 0.77]	0.43 [0.22; 0.82]	0.42 [0.20; 0.89]	0.42 [0.21; 0.84]	0.44 [0.21; 0.91]	0.45 [0.20; 0.99]	0.49 [0.18; 1.31]	0.48 [0.23; 1.01]	0.47 [0.24; 0.92]	0.49 [0.23; 1.07]	0.55 [0.24; 1.25]	0.58 [0.34; 0.99]	0.57 [0.26; 1.24]	0.73 [0.35; 1.52]	0.86 [0.60; 1.24]	0.91 [0.68; 1.20]	0.95 [0.65; 1.37]	0.98 [0.67; 1.43]	Adalimumab (high induction

																																					regimen<br>)

Biologically exposed

Abatacept 10 mg/kg	.		.	.		.	.	.	.		.	.							
0.81 [0.24; 2.74]	Mongersen 160 mg	.	.	.		.	.												
0.85 [0.26; 2.70]	1.04 [0.33; 3.32]	Abatacept 3 mg/kg	.	.		.	.												
0.85 [0.12; 5.95]	1.05 [0.19; 5.72]	1.00 [0.15; 6.81]	Adalimumab 80 mg	.		.	.												
0.66 [0.21; 2.07]	0.81 [0.43; 1.54]	0.78 [0.26; 2.31]	0.78 [0.15; 4.04]	Etrolizumab 105 mg		.													
0.62 [0.21; 1.84]	0.76 [0.45; 1.30]	0.73 [0.26; 2.05]	0.73 [0.15; 3.66]	0.94 [0.66; 1.33]	Placebo														
0.62 [0.16; 2.45]	0.76 [0.28; 2.06]	0.73 [0.19; 2.76]	0.73 [0.12; 4.48]	0.94 [0.38; 2.33]	1.00 [0.43; 2.31]	Tofacitinib 15 mg													
0.59 [0.19; 1.83]	0.72 [0.38; 1.35]	0.69 [0.23; 2.04]	0.69 [0.13; 3.57]	0.89 [0.65; 1.20]	0.94 [0.68; 1.32]	0.95 [0.38; 2.34]	Etrolizumab 210 mg												
0.59 [0.18; 1.90]	0.72 [0.36; 1.44]	0.69 [0.23; 2.12]	0.69 [0.13; 3.67]	0.89 [0.51; 1.55]	0.95 [0.61; 1.46]	0.95 [0.41; 2.20]	1.00 [0.58; 1.74]	Tofacitinib 5 mg		.	.	.							
0.53 [0.16; 1.70]	0.65 [0.33; 1.29]	0.63 [0.21; 1.90]	0.62 [0.12; 3.30]	0.80 [0.46; 1.39]	0.85 [0.56; 1.30]	0.86 [0.37; 1.97]	0.90 [0.53; 1.55]	0.90 [0.60; 1.36]	Tofacitinib 10 mg	.	.	.							
0.51 [0.15; 1.69]	0.62 [0.19; 2.08]	0.60 [0.19; 1.89]	0.60 [0.09; 4.15]	0.77 [0.25; 2.39]	0.82 [0.28; 2.40]	0.82 [0.21; 3.22]	0.87 [0.28; 2.68]	0.86 [0.27; 2.76]	0.96 [0.30; 3.05]	Abatacept 30 mg/kg	.	.							
0.45 [0.13; 1.53]	0.56 [0.26; 1.19]	0.54 [0.17; 1.71]	0.53 [0.10; 2.91]	0.69 [0.36; 1.30]	0.73 [0.43; 1.25]	0.73 [0.27; 1.99]	0.77 [0.41; 1.46]	0.77 [0.39; 1.54]	0.86 [0.43; 1.69]	0.89 [0.27; 2.97]	Vedolizumab 300 mg	.							
0.35 [0.10; 1.31]	0.44 [0.18; 1.07]	0.42 [0.12; 1.47]	0.42 [0.09; 1.85]	0.54 [0.24; 1.19]	0.57 [0.28; 1.17]	0.57 [0.19; 1.73]	0.61 [0.27; 1.34]	0.60 [0.26; 1.40]	0.67 [0.29; 1.54]	0.70 [0.19; 2.54]	0.78 [0.32; 1.92]	Adalimumab 160 mg							
0.34 [0.11; 1.03]	0.42 [0.23; 0.74]	0.40 [0.14; 1.15]	0.40 [0.08; 2.03]	0.51 [0.34; 0.77]	0.55 [0.44; 0.68]	0.55 [0.23; 1.31]	0.58 [0.39; 0.86]	0.58 [0.35; 0.94]	0.64 [0.40; 1.03]	0.67 [0.22; 2.00]	0.75 [0.42; 1.33]	0.96 [0.45; 2.02]	Risankizumab 1200 mg	.		.	.	.	.
0.33 [0.10; 1.09]	0.41 [0.20; 0.83]	0.39 [0.13; 1.22]	0.39 [0.07; 2.10]	0.50 [0.28; 0.90]	0.54 [0.34; 0.86]	0.54 [0.21; 1.41]	0.57 [0.32; 1.01]	0.57 [0.30; 1.07]	0.63 [0.33; 1.18]	0.65 [0.20; 2.12]	0.73 [0.36; 1.49]	0.94 [0.40; 2.21]	0.98 [0.59; 1.65]	Ustekinumab 130 mg	.	.	.		.
0.32 [0.10; 0.96]	0.39 [0.22; 0.69]	0.37 [0.13; 1.07]	0.37 [0.07; 1.89]	0.48 [0.32; 0.72]	0.51 [0.41; 0.63]	0.51 [0.21; 1.22]	0.54 [0.36; 0.80]	0.54 [0.33; 0.88]	0.60 [0.37; 0.96]	0.62 [0.21; 1.87]	0.70 [0.39; 1.24]	0.89 [0.42; 1.89]	0.93 [0.81; 1.07]	0.95 [0.57; 1.59]	Risankizumab 600 mg	.	.	.	.
0.31 [0.09; 1.04]	0.38 [0.18; 0.81]	0.36 [0.11; 1.16]	0.36 [0.07; 1.98]	0.47 [0.25; 0.88]	0.50 [0.29; 0.85]	0.50 [0.18; 1.35]	0.53 [0.28; 0.99]	0.52 [0.26; 1.05]	0.58 [0.29; 1.15]	0.61 [0.18; 2.02]	0.68 [0.32; 1.45]	0.87 [0.35; 2.13]	0.91 [0.51; 1.62]	0.93 [0.51; 1.68]	0.97 [0.55; 1.73]	Ustekinumab 1 mg/kg			.
0.30 [0.09; 1.00]	0.37 [0.17; 0.78]	0.35 [0.11; 1.12]	0.35 [0.06; 1.91]	0.45 [0.24; 0.85]	0.48 [0.28; 0.81]	0.48 [0.18; 1.30]	0.51 [0.27; 0.95]	0.51 [0.25; 1.00]	0.56 [0.29; 1.10]	0.59 [0.18; 1.94]	0.66 [0.31; 1.39]	0.84 [0.34; 2.05]	0.88 [0.49; 1.56]	0.89 [0.50; 1.61]	0.94 [0.53; 1.67]	0.97 [0.58; 1.62]	Ustekinumab 3 mg/kg		.
0.27 [0.08; 0.85]	0.33 [0.17; 0.63]	0.31 [0.10; 0.94]	0.31 [0.06; 1.64]	0.40 [0.24; 0.68]	0.43 [0.29; 0.63]	0.43 [0.17; 1.09]	0.45 [0.27; 0.76]	0.45 [0.25; 0.81]	0.50 [0.28; 0.89]	0.52 [0.17; 1.64]	0.59 [0.30; 1.14]	0.75 [0.33; 1.70]	0.78 [0.50; 1.23]	0.80 [0.55; 1.15]	0.84 [0.54; 1.31]	0.86 [0.53; 1.41]	0.89 [0.55; 1.45]	Ustekinumab 6 mg/kg	.
0.21 [0.07; 0.70]	0.26 [0.13; 0.53]	0.25 [0.08; 0.78]	0.25 [0.05; 1.34]	0.33 [0.19; 0.57]	0.35 [0.22; 0.54]	0.35 [0.13; 0.90]	0.37 [0.21; 0.64]	0.37 [0.20; 0.68]	0.41 [0.22; 0.75]	0.42 [0.13; 1.35]	0.47 [0.24; 0.95]	0.61 [0.26; 1.41]	0.63 [0.39; 1.04]	0.64 [0.34; 1.23]	0.68 [0.41; 1.11]	0.70 [0.35; 1.40]	0.72 [0.36; 1.44]	0.81 [0.45; 1.46]	Upadacitinib 45 mg

Maintenance

Biologically naive

Infliximab + Azathioprine 5 mg/kg + 2-2.5 mg/kg	.		.	.	.	.	.	.	.	.	.	.	.	.	.	.	.	.	.	.	.	.	.	.
0.78 [0.31; 1.96]	Ustekinumab 6 mg/kg	.	.	.		.	.	.							.	.	.	.	.					
0.67 [0.42; 1.06]	0.85 [0.38; 1.89]	Azathioprine 2-2.5 mg/kg	.	.	.	.	.	.							.	.	.	.	.					
0.67 [0.30; 1.49]	0.86 [0.34; 2.16]	1.01 [0.52; 1.93]	Methotrexate 15 mg	.	.	.	.	.							.	.	.	.	.					
0.65 [0.31; 1.36]	0.83 [0.35; 1.99]	0.98 [0.55; 1.73]	0.97 [0.46; 2.04]	Upadacitinib 30 mg	.	.	.								.	.	.	.	.					
0.55 [0.29; 1.04]	0.70 [0.36; 1.35]	0.82 [0.52; 1.29]	0.82 [0.43; 1.57]	0.84 [0.47; 1.50]	Adalimumab 40 mg once per 2 weeks	.	.	.							.	.	.	.	.					
0.55 [0.28; 1.05]	0.70 [0.31; 1.57]	0.82 [0.52; 1.31]	0.82 [0.42; 1.58]	0.84 [0.47; 1.52]	1.00 [0.63; 1.59]	Infliximab 10 mg/kg	.	.							.	.	.	.	.					
0.56 [0.19; 1.63]	0.71 [0.22; 2.31]	0.84 [0.32; 2.21]	0.83 [0.28; 2.46]	0.86 [0.30; 2.43]	1.02 [0.39; 2.70]	1.02 [0.38; 2.72]	Budesonide 3-9 mg	.							.	.	.	.	.					
0.53 [0.26; 1.08]	0.68 [0.29; 1.60]	0.80 [0.47; 1.37]	0.80 [0.39; 1.63]	0.82 [0.49; 1.39]	0.98 [0.57; 1.67]	0.98 [0.57; 1.69]	0.96 [0.35; 2.65]	Upadacitinib 15 mg	.	.	.	.	.	.	.	.	.	.	.					
0.52 [0.28; 0.97]	0.66 [0.30; 1.44]	0.78 [0.51; 1.19]	0.77 [0.41; 1.46]	0.80 [0.46; 1.39]	0.94 [0.62; 1.44]	0.94 [0.61; 1.46]	0.93 [0.35; 2.43]	0.97 [0.58; 1.62]	Adalimumab 40 mg	.	.	.	.	.	.	.	.	.	.					
0.50 [0.26; 0.95]	0.64 [0.29; 1.41]	0.75 [0.47; 1.18]	0.74 [0.38; 1.43]	0.76 [0.43; 1.37]	0.91 [0.58; 1.43]	0.91 [0.64; 1.29]	0.89 [0.33; 2.36]	0.93 [0.54; 1.60]	0.96 [0.62; 1.48]	Infliximab 5 mg/kg	.	.	.	.	.	.	.	.	.					
0.50 [0.27; 0.90]	0.64 [0.26; 1.53]	0.75 [0.51; 1.09]	0.74 [0.35; 1.57]	0.77 [0.39; 1.52]	0.91 [0.51; 1.63]	0.91 [0.50; 1.65]	0.89 [0.32; 2.52]	0.93 [0.49; 1.79]	0.96 [0.55; 1.70]	1.00 [0.56; 1.81]	Step-up to azathioprine 2-2.5 mg/kg	.	.	.	.	.	.	.	.					
0.47 [0.25; 0.86]	0.60 [0.28; 1.29]	0.70 [0.48; 1.04]	0.70 [0.38; 1.29]	0.72 [0.42; 1.23]	0.85 [0.58; 1.27]	0.85 [0.57; 1.29]	0.84 [0.33; 2.14]	0.87 [0.54; 1.43]	0.90 [0.63; 1.30]	0.94 [0.63; 1.41]	0.94 [0.55; 1.61]	5-aminosalicylic acid		.	.	.	.	.	.					

												500-1000 mg												
0.46 [0.25; 0.84]	0.59 [0.28; 1.27]	0.69 [0.48; 1.01]	0.69 [0.37; 1.27]	0.71 [0.42; 1.21]	0.84 [0.57; 1.25]	0.84 [0.56; 1.26]	0.83 [0.34; 2.02]	0.86 [0.53; 1.40]	0.89 [0.62; 1.28]	0.93 [0.62; 1.38]	0.93 [0.55; 1.57]	0.99 [0.74; 1.31]	Budesonide 6 mg		.	.	.	.	.					
0.43 [0.22; 0.85]	0.55 [0.24; 1.27]	0.65 [0.40; 1.06]	0.65 [0.32; 1.29]	0.67 [0.36; 1.24]	0.79 [0.48; 1.31]	0.79 [0.47; 1.32]	0.77 [0.30; 2.00]	0.81 [0.45; 1.45]	0.84 [0.52; 1.36]	0.87 [0.52; 1.45]	0.87 [0.47; 1.61]	0.92 [0.60; 1.42]	0.94 [0.68; 1.29]	Budesonide 9 mg	.	.	.	.	.	.	.	.	.	.
0.43 [0.21; 0.85]	0.55 [0.24; 1.25]	0.64 [0.38; 1.07]	0.64 [0.32; 1.27]	0.66 [0.35; 1.22]	0.78 [0.47; 1.30]	0.78 [0.46; 1.31]	0.76 [0.28; 2.08]	0.80 [0.44; 1.43]	0.82 [0.51; 1.34]	0.86 [0.51; 1.44]	0.86 [0.45; 1.61]	0.91 [0.57; 1.45]	0.92 [0.58; 1.46]	0.99 [0.56; 1.73]	Filgotinib 100 mg		.	.	.	.	.	.		.
0.43 [0.21; 0.85]	0.55 [0.24; 1.26]	0.64 [0.38; 1.07]	0.64 [0.32; 1.28]	0.66 [0.35; 1.23]	0.78 [0.47; 1.30]	0.78 [0.46; 1.32]	0.76 [0.28; 2.09]	0.80 [0.44; 1.44]	0.82 [0.50; 1.35]	0.86 [0.51; 1.45]	0.86 [0.45; 1.62]	0.91 [0.57; 1.46]	0.92 [0.58; 1.47]	0.99 [0.56; 1.73]	1.00 [0.67; 1.50]	Filgotinib 200 mg	.	.	.	.	.	.		.
0.43 [0.21; 0.85]	0.55 [0.24; 1.25]	0.64 [0.39; 1.06]	0.64 [0.32; 1.27]	0.66 [0.35; 1.22]	0.78 [0.47; 1.29]	0.78 [0.46; 1.31]	0.76 [0.28; 2.08]	0.80 [0.44; 1.43]	0.82 [0.51; 1.34]	0.86 [0.51; 1.43]	0.86 [0.46; 1.61]	0.91 [0.58; 1.44]	0.92 [0.59; 1.46]	0.99 [0.57; 1.72]	1.00 [0.57; 1.75]	1.00 [0.57; 1.76]	Vedolizumab 108 mg	.	.	.	.	.		.
0.42 [0.22; 0.82]	0.54 [0.24; 1.21]	0.64 [0.40; 1.01]	0.63 [0.33; 1.23]	0.65 [0.36; 1.18]	0.77 [0.49; 1.23]	0.77 [0.48; 1.25]	0.76 [0.28; 2.03]	0.79 [0.46; 1.37]	0.82 [0.53; 1.28]	0.85 [0.53; 1.37]	0.85 [0.47; 1.55]	0.91 [0.60; 1.37]	0.92 [0.61; 1.38]	0.98 [0.58; 1.65]	0.99 [0.59; 1.68]	0.99 [0.58; 1.69]	0.99 [0.59; 1.68]	5-aminosalicylic acid 2000 mg	.	.	.	.		.
0.41 [0.19; 0.85]	0.52 [0.22; 1.25]	0.61 [0.34; 1.09]	0.61 [0.29; 1.28]	0.62 [0.31; 1.24]	0.74 [0.41; 1.33]	0.74 [0.41; 1.34]	0.73 [0.26; 2.06]	0.76 [0.40; 1.46]	0.79 [0.45; 1.38]	0.82 [0.45; 1.47]	0.81 [0.41; 1.63]	0.87 [0.50; 1.49]	0.88 [0.51; 1.51]	0.94 [0.50; 1.76]	0.95 [0.51; 1.79]	0.95 [0.50; 1.80]	0.95 [0.51; 1.79]	0.96 [0.53; 1.74]	5-aminosalicylic acid 1500 mg	.	.	.		.
0.40 [0.22; 0.76]	0.52 [0.24; 1.13]	0.61 [0.40; 0.93]	0.60 [0.32; 1.14]	0.62 [0.36; 1.09]	0.74 [0.48; 1.13]	0.74 [0.47; 1.14]	0.72 [0.28; 1.89]	0.76 [0.45; 1.26]	0.78 [0.52; 1.17]	0.81 [0.53; 1.25]	0.81 [0.46; 1.43]	0.86 [0.60; 1.25]	0.88 [0.61; 1.26]	0.93 [0.58; 1.52]	0.95 [0.58; 1.55]	0.95 [0.58; 1.55]	0.95 [0.58; 1.54]	0.95 [0.61; 1.49]	1.00 [0.57; 1.75]	Certolizumab pegol 400 mg	.	.		.
0.39 [0.20; 0.74]	0.50 [0.22; 1.11]	0.58 [0.37; 0.92]	0.58 [0.30; 1.12]	0.60 [0.34; 1.07]	0.71 [0.45; 1.12]	0.71 [0.44; 1.14]	0.70 [0.27; 1.81]	0.73 [0.42; 1.25]	0.75 [0.49; 1.16]	0.78 [0.49; 1.25]	0.78 [0.44; 1.40]	0.83 [0.57; 1.23]	0.84 [0.61; 1.18]	0.90 [0.57; 1.43]	0.91 [0.55; 1.53]	0.91 [0.54; 1.54]	0.91 [0.55; 1.53]	0.92 [0.57; 1.48]	0.96 [0.53; 1.73]	0.96 [0.63; 1.49]	Budesonide 3 mg	.		.
0.38 [0.19; 0.73]	0.48 [0.21; 1.08]	0.56 [0.35; 0.90]	0.56 [0.29; 1.09]	0.58 [0.32; 1.05]	0.69 [0.43; 1.10]	0.69 [0.42; 1.11]	0.67 [0.25; 1.80]	0.70 [0.40; 1.22]	0.73 [0.46; 1.14]	0.76 [0.47; 1.22]	0.75 [0.41; 1.37]	0.80 [0.53; 1.22]	0.81 [0.54; 1.23]	0.87 [0.51; 1.47]	0.88 [0.52; 1.50]	0.88 [0.52; 1.50]	0.88 [0.52; 1.49]	0.89 [0.54; 1.45]	0.93 [0.51; 1.69]	0.93 [0.59; 1.45]	0.96 [0.60; 1.55]	Budesonide 1 mg		.
0.38 [0.22; 0.67]	0.49 [0.24; 1.02]	0.58 [0.42; 0.79]	0.57 [0.32; 1.01]	0.59 [0.37; 0.95]	0.70 [0.51; 0.96]	0.70 [0.50; 0.98]	0.69 [0.27; 1.72]	0.72 [0.47; 1.10]	0.74 [0.56; 0.98]	0.77 [0.56; 1.07]	0.77 [0.47; 1.26]	0.82 [0.65; 1.04]	0.83 [0.66; 1.04]	0.89 [0.60; 1.31]	0.90 [0.60; 1.34]	0.90 [0.60; 1.35]	0.90 [0.61; 1.34]	0.91 [0.64; 1.28]	0.95 [0.58; 1.54]	0.95 [0.72; 1.26]	0.98 [0.71; 1.37]	1.02 [0.72; 1.45]	Placebo	
0.32 [0.16; 0.65]	0.41 [0.18; 0.96]	0.48 [0.28; 0.83]	0.48 [0.23; 0.98]	0.50 [0.26; 0.95]	0.59 [0.34; 1.01]	0.59 [0.34; 1.02]	0.58 [0.21; 1.60]	0.60 [0.33; 1.11]	0.62 [0.37; 1.05]	0.65 [0.38; 1.12]	0.65 [0.33; 1.24]	0.69 [0.42; 1.13]	0.70 [0.43; 1.14]	0.74 [0.41; 1.34]	0.75 [0.42; 1.36]	0.75 [0.42; 1.37]	0.75 [0.42; 1.36]	0.76 [0.44; 1.32]	0.79 [0.41; 1.53]	0.80 [0.47; 1.34]	0.83 [0.48; 1.42]	0.86 [0.49; 1.50]	0.84 [0.54; 1.30]	5-aminosalicylic acid 1000 mg

Biologically exposed

Upadacitinib 30 mg	.	.		.	.	.	.	.	.	.	
0.93 [0.63; 1.37]	Ustekinumab 90 mg	.	.	.	.		.	.	.	.	
0.87 [0.67; 1.14]	0.94 [0.63; 1.41]	Vedolizumab 108 mg	.	.	.	.	.	.	.	.	
0.83 [0.68; 1.02]	0.90 [0.62; 1.31]	0.95 [0.74; 1.22]	Upadacitinib 15 mg	.	.	.	.	.	.	.	
0.83 [0.63; 1.09]	0.89 [0.59; 1.35]	0.95 [0.71; 1.27]	1.00 [0.77; 1.29]	Risankizumab 180 mg		.	.	.	.	.	
0.78 [0.59; 1.02]	0.84 [0.56; 1.27]	0.89 [0.66; 1.20]	0.93 [0.72; 1.21]	0.94 [0.73; 1.20]	Risankizumab 360 mg	.	.	.	.	.	
0.78 [0.55; 1.11]	0.84 [0.57; 1.23]	0.89 [0.62; 1.29]	0.94 [0.67; 1.32]	0.94 [0.64; 1.37]	1.00 [0.69; 1.46]	Ustekinumab (90 mg/12 wk)	.	.	.	.	
0.73 [0.56; 0.95]	0.79 [0.53; 1.18]	0.83 [0.63; 1.11]	0.88 [0.69; 1.12]	0.88 [0.66; 1.18]	0.94 [0.70; 1.26]	0.94 [0.65; 1.35]	Abatacept 10 mg/kg	.	.	.	
0.71 [0.57; 0.88]	0.76 [0.52; 1.11]	0.81 [0.63; 1.03]	0.85 [0.69; 1.03]	0.85 [0.66; 1.10]	0.91 [0.70; 1.17]	0.90 [0.64; 1.27]	0.97 [0.76; 1.23]	Etrolizumab 105 mg	.	.	
0.67 [0.46; 0.98]	0.72 [0.44; 1.18]	0.77 [0.51; 1.14]	0.80 [0.56; 1.16]	0.81 [0.54; 1.21]	0.86 [0.57; 1.29]	0.86 [0.54; 1.36]	0.92 [0.62; 1.36]	0.95 [0.66; 1.37]	Tofacitinib 10 mg		
0.67 [0.46; 0.98]	0.72 [0.44; 1.18]	0.77 [0.51; 1.14]	0.80 [0.56; 1.16]	0.81 [0.54; 1.21]	0.86 [0.57; 1.29]	0.86 [0.54; 1.36]	0.92 [0.62; 1.36]	0.95 [0.66; 1.37]	1.00 [0.71; 1.40]	Tofacitinib 5 mg	
0.63 [0.53; 0.74]	0.67 [0.48; 0.96]	0.72 [0.58; 0.87]	0.75 [0.65; 0.86]	0.75 [0.61; 0.94]	0.80 [0.65; 1.00]	0.80 [0.59; 1.09]	0.86 [0.70; 1.04]	0.89 [0.77; 1.02]	0.93 [0.66; 1.32]	0.93 [0.66; 1.32]	Placebo

10) SUCRA rankings

Induction remission

Immunomodulator naive

Treatment	SUCRA rank
Placebo	0.1
Usual care (budesonide or methylprednisolone)	0.2874
Azathioprine 2-2.5 mg/kg	0.3262
Methotrexate 25 mg	0.599
Infliximab 5 mg/kg	0.7394
Infliximab + Azathioprine 5 mg/kg + 2-2.5 mg/kg	0.948

Biologic naive

Treatment	SUCRA rank
Adalimumab (high induction regimen)	0.9372
BI 695501 160 mg	0.9366
Ustekinumab 6 mg/kg	0.9319
Adalimumab 160 mg	0.9164
Adalimumab + Azathioprine 160 mg + 25-50 mg	0.9039
Infliximab + Azathioprine 5 mg/kg + 2-2.5 mg/kg	0.8505
Infliximab 5 mg/kg	0.7393
Adalimumab 80 mg	0.7341
CT-P13 5 mg/kg	0.7184
Methotrexate 25 mg	0.6377
Prednisolone 40 mg	0.6279
Budesonide 4.5 mg	0.6193
5-aminosalicylic acid 1000 mg	0.6023
Budesonide 6 mg	0.5807
Budesonide 7.5 mg	0.5362
Budesonide 3 mg	0.5248
Adalimumab 40 mg	0.5198
Natalizumab 6 mg/kg	0.5109
Natalizumab 12 mg/kg	0.46
Budesonide 9 mg	0.4594
Natalizumab 300 mg	0.4344
5-aminosalicylic acid 1500 mg	0.4024
Azathioprine 2.5 mg/kg (dose adapted)	0.3977
Azathioprine 2-2.5 mg/kg	0.3876
Certolizumab pegol 400 mg	0.3777
Azathioprine 40 mg/kg	0.3698
Upadacitinib 45 mg	0.3547
5-aminosalicylic acid 500-1000 mg	0.3196

Budesonide 1.5 mg	0.3123
Methotrexate 12.5 mg	0.2886
Natalizumab 3 mg/kg	0.2571
5-aminosalicylic acid 2000 mg	0.1614
Mongersen 160 mg	0.1605
Budesonide 2 mg	0.1538
CDP571 10 mg/kg	0.1476
Placebo	0.1296
Secukinumab 10 mg/kg	0.0979

Biologic exposed

Treatment	SUCRA rank
Upadacitinib 45 mg	0.9479
Ustekinumab 6 mg/kg	0.8622
Ustekinumab 3 mg/kg	0.7935
Ustekinumab 1 mg/kg	0.7634
Risankizumab 600 mg	0.7616
Ustekinumab 130 mg	0.7175
Risankizumab 1200 mg	0.7082
Adalimumab 160 mg	0.6618
Vedolizumab 300 mg	0.5139
Abatacept 30 mg/kg	0.4668
Tofacitinib 10 mg	0.4119
Tofacitinib 5 mg	0.3505
Tofacitinib 15 mg	0.3494
Etrolizumab 210 mg	0.341
Placebo	0.3041
Adalimumab 80 mg	0.2699
Etrolizumab 105 mg	0.2629
Abatacept 3 mg/kg	0.2055
Mongersen 160 mg	0.1705
Abatacept 10 mg/kg	0.1374

Maintenance

Biologic naive

Treatment	SUCRA rank
Infliximab + Azathioprine 5 mg/kg + 2-2.5 mg/kg	0.9568
Ustekinumab 6 mg/kg	0.841
Azathioprine 2-2.5 mg/kg	0.8295
Upadacitinib 30 mg	0.7781
Methotrexate 15 mg	0.7776
Infliximab 10 mg/kg	0.6597
Adalimumab 40 mg once per 2 weeks	0.652
Upadacitinib 15 mg	0.63
Adalimumab 40 mg	0.5969
Budesonide 3-9 mg	0.5881
Infliximab 5 mg/kg	0.5547
Step-up to azathioprine 2-2.5 mg/kg	0.5289
5-aminosalicylic acid 500-1000 mg	0.4703
Budesonide 6 mg	0.4598
Budesonide 9 mg	0.3682
Filgotinib 200 mg	0.3558
Vedolizumab 108 mg	0.3529
Filgotinib 100 mg	0.3452
5-aminosalicylic acid 2000 mg	0.3445
5-aminosalicylic acid 1500 mg	0.3212
Certolizumab pegol 400 mg	0.2881
Budesonide 3 mg	0.2606
Budesonide 1 mg	0.2265
Placebo	0.2013
5-aminosalicylic acid 1000 mg	0.1121

Biologic exposed

Treatment	SUCRA rank
Upadacitinib 30 mg	0.9237
Ustekinumab 90 mg	0.7619
Vedolizumab 108 mg	0.7259
Upadacitinib 15 mg	0.6471
Risankizumab 180 mg	0.6384
Ustekinumab (90 mg/12 wk)	0.5022
Risankizumab 360 mg	0.5026
Abatacept 10 mg/kg	0.3788
Etrolizumab 105 mg	0.3085
Tofacitinib 5 mg	0.2615
Tofacitinib 10 mg	0.263
Placebo	0.0865

11) Direct vs indirect evidence

Induction remission

Immunomodulator naive

In this network meta-analysis no indirect evidence was included and there are no closed loops within the network, therefore we do not provide the figure with comparisons of direct and indirect evidence for this network.

Biological naive

Comparison	Number of Studies	Direct Evidence	I2	RR	95%-CI
**5-aminosalicylic acid 1500 mg:Budesonide 3 mg**					
Direct estimate	1	0.84		0.86	[0.69; 1.08]
Indirect estimate				0.84	[0.50; 1.41]
Network estimate				0.86	[0.70; 1.06]
**5-aminosalicylic acid 1500 mg:Budesonide 9 mg**					
Direct estimate	1	0.79		0.93	[0.73; 1.17]
Indirect estimate				0.95	[0.60; 1.50]
Network estimate				0.93	[0.75; 1.15]
**Adalimumab 160 mg:Adalimumab 40 mg**					
Direct estimate	1	0.96		2.02	[1.12; 3.66]
Indirect estimate				5.91	[0.38; 90.96]
Network estimate				2.12	[1.19; 3.79]
**Adalimumab 160 mg:Adalimumab 80 mg**					
Direct estimate	2	0.97	0%	1.48	[0.93; 2.36]
Indirect estimate				9.61	[0.67; 137.45]
Network estimate				1.57	[0.99; 2.48]
**Adalimumab 40 mg:Adalimumab 80 mg**					
Direct estimate	1	0.94		0.73	[0.38; 1.40]
Indirect estimate				0.87	[0.06; 12.82]
Network estimate				0.74	[0.39; 1.39]
**Adalimumab 40 mg:Placebo**					
Direct estimate	1	0.75		1.44	[0.65; 3.20]
Indirect estimate				2.74	[0.70; 10.70]
Network estimate				1.70	[0.85; 3.38]
**Adalimumab 80 mg:Placebo**					
Direct estimate	2	0.78	0%	1.85	[0.95; 3.60]
Indirect estimate				5.03	[1.42; 17.81]
Network estimate				2.30	[1.28; 4.15]
**Azathioprine 2-2.5 mg/kg:Infliximab + Azathioprine 5 mg/kg + 2-2.5 mg/kg**					
Direct estimate	1	0.89		0.52	[0.37; 0.72]
Indirect estimate				0.30	[0.12; 0.78]
Network estimate				0.49	[0.35; 0.67]
**Azathioprine 2-2.5 mg/kg:Infliximab 5 mg/kg**					
Direct estimate	1	0.95		0.65	[0.45; 0.92]
Indirect estimate				0.28	[0.06; 1.31]
Network estimate				0.62	[0.44; 0.88]
**Azathioprine 2-2.5 mg/kg:Methotrexate 12.5 mg**					
Direct estimate	1	0.77		0.81	[0.26; 2.52]
Indirect estimate				5.02	[0.62; 40.61]
Network estimate				1.23	[0.45; 3.33]
**Azathioprine 2-2.5 mg/kg:Methotrexate 25 mg**					
Direct estimate	1	0.62		0.75	[0.38; 1.50]
Indirect estimate				0.66	[0.27; 1.59]
Network estimate				0.71	[0.41; 1.23]
**Azathioprine 2-2.5 mg/kg:Placebo**					
Direct estimate	1	0.22		0.68	[0.23; 1.99]
Indirect estimate				1.72	[0.98; 3.02]
Network estimate				1.40	[0.85; 2.31]
**Budesonide 1.5 mg:Budesonide 4.5 mg**					
Direct estimate	1	0.95		0.65	[0.42; 1.00]
Indirect estimate				2.92	[0.39; 21.81]
Network estimate				0.69	[0.45; 1.07]
**Budesonide 1.5 mg:Placebo**					
Direct estimate	1	0.70		1.67	[0.91; 3.07]
Indirect estimate				0.74	[0.29; 1.86]
Network estimate				1.30	[0.78; 2.17]
**Budesonide 4.5 mg:Budesonide 7.5 mg**					
Direct estimate	1	0.94		1.16	[0.78; 1.72]
Indirect estimate				0.35	[0.07; 1.71]
Network estimate				1.08	[0.74; 1.59]
**Budesonide 4.5 mg:Budesonide 9 mg**					
Direct estimate	1	0.90		1.12	[0.80; 1.58]
Indirect estimate				2.65	[0.96; 7.37]
Network estimate				1.22	[0.89; 1.69]
**Budesonide 4.5 mg:Placebo**					
Direct estimate	2	0.91	35%	2.00	[1.37; 2.91]
Indirect estimate				1.03	[0.31; 3.41]
Network estimate				1.88	[1.31; 2.70]
**Budesonide 7.5 mg:Placebo**					
Direct estimate	1	0.66		2.22	[1.25; 3.95]
Indirect estimate				1.08	[0.48; 2.40]
Network estimate				1.74	[1.09; 2.77]
**Budesonide 9 mg:Infliximab + Azathioprine 5 mg/kg + 2-2.5 mg/kg**					
Direct estimate	1	0.82		0.48	[0.32; 0.74]
Indirect estimate				0.82	[0.33; 2.02]
Network estimate				0.53	[0.36; 0.78]
**Budesonide 9 mg:Placebo**					
Direct estimate	1	0.58		1.44	[0.86; 2.43]
Indirect estimate				1.68	[0.91; 3.11]
Network estimate				1.54	[1.03; 2.29]
**Infliximab + Azathioprine 5 mg/kg + 2-2.5 mg/kg:Infliximab 5 mg/kg**					
Direct estimate	1	0.99		1.25	[0.94; 1.67]
Indirect estimate				4.35	[0.42; 45.23]
Network estimate				1.28	[0.96; 1.69]
**Methotrexate 12.5 mg:Placebo**					
Direct estimate	1	0.85		0.83	[0.29; 2.41]
Indirect estimate				6.79	[0.54; 84.90]
Network estimate				1.14	[0.43; 3.04]
**Methotrexate 25 mg:Placebo**					
Direct estimate	1	0.66		2.06	[1.07; 3.94]
Indirect estimate				1.81	[0.73; 4.48]
Network estimate				1.97	[1.16; 3.34]
**Natalizumab 12 mg/kg:Natalizumab 3 mg/kg**					
Direct estimate	1	0.98		1.40	[0.83; 2.38]
Indirect estimate				0.09	[0.00; 3.62]
Network estimate				1.33	[0.79; 2.24]
**Natalizumab 12 mg/kg:Placebo**					
Direct estimate	1	0.98		1.45	[0.84; 2.51]
Indirect estimate				19.41	[0.59; 633.86]
Network estimate				1.55	[0.90; 2.65]
**Natalizumab 3 mg/kg:Natalizumab 6 mg/kg**					
Direct estimate	1	0.98		0.66	[0.40; 1.08]
Indirect estimate				6.97	[0.29; 166.49]
Network estimate				0.70	[0.43; 1.13]
**Natalizumab 6 mg/kg:Placebo**					
Direct estimate	1	0.97		1.57	[0.94; 2.62]
Indirect estimate				15.13	[0.72; 316.01]
Network estimate				1.67	[1.01; 2.77]

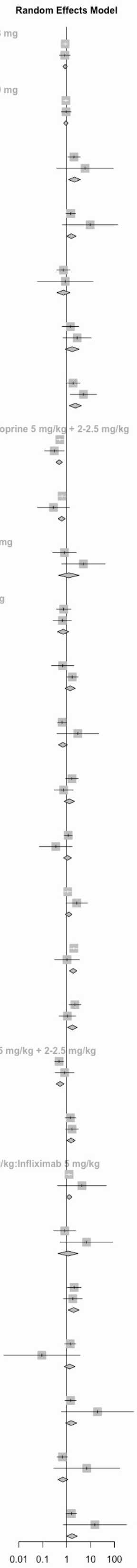

Biological exposed

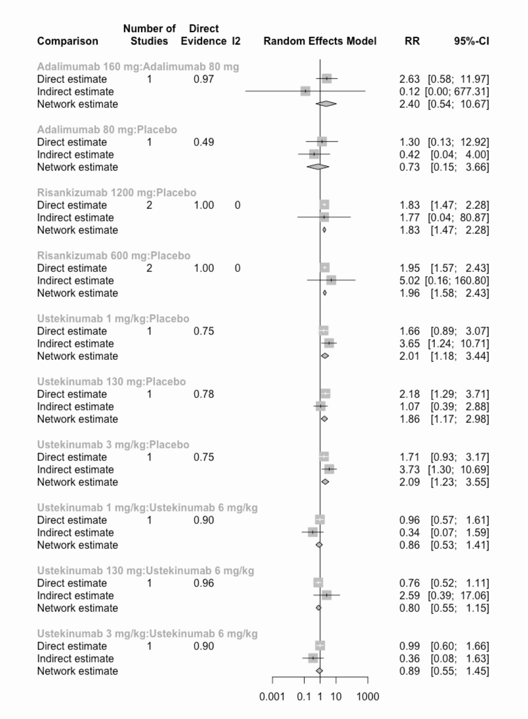

Maintenance

Immunomodulator naive

Only direct evidence included

Biological naive

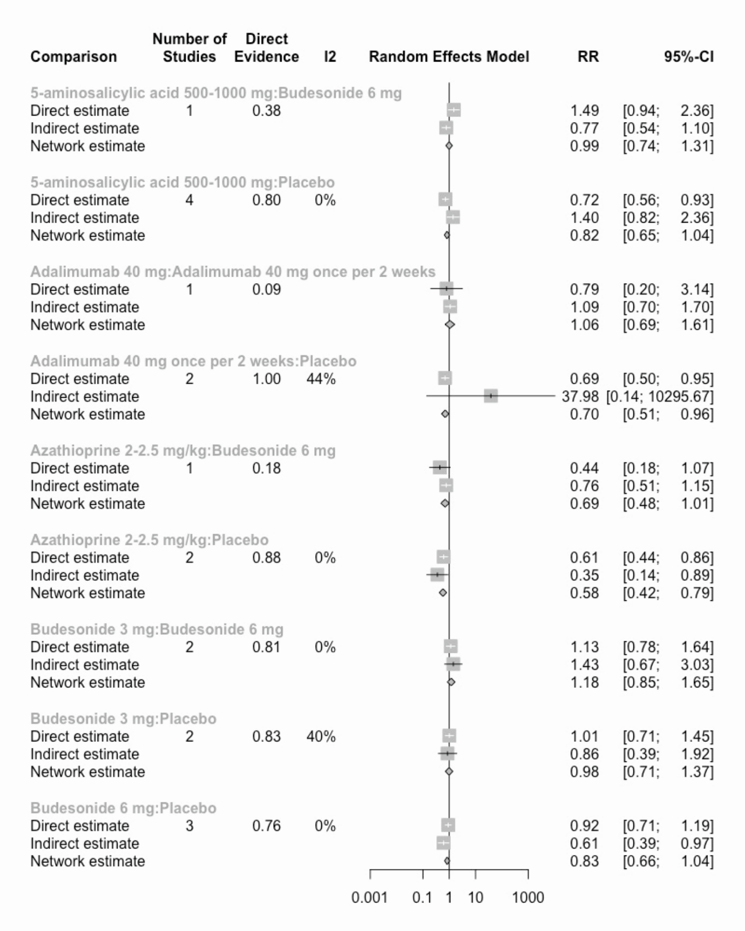

Biological exposed

Only direct evidence included

12) Scenario analyses

Results of sensitivity analysis on steroid-free outcomes

Induction of remission in biologically naive patients, only 'steroid-free' outcome studies

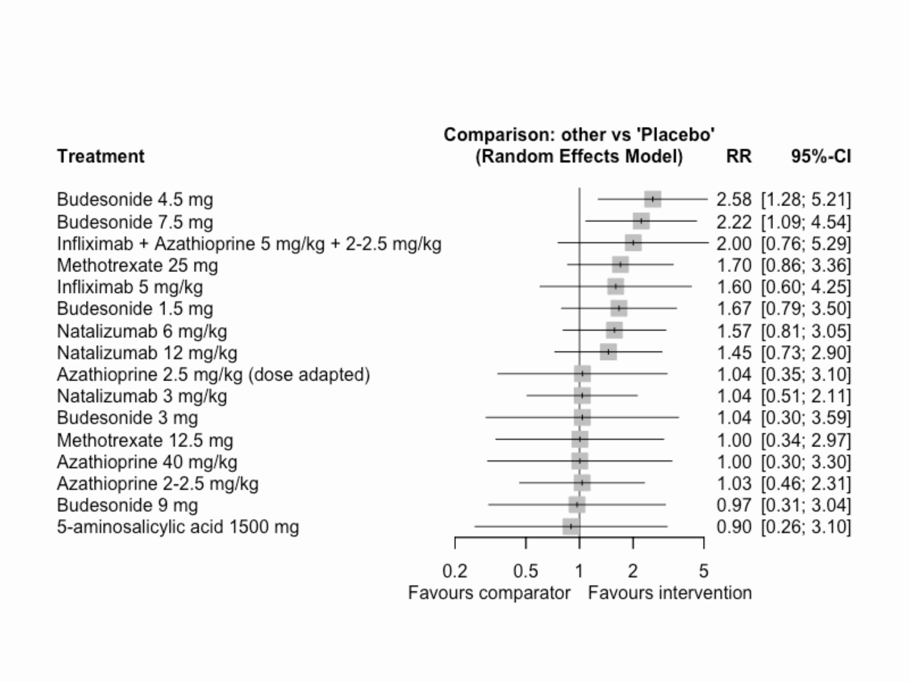

Induction of remission in biologically naive patients, only 'Not steroid-free' outcome studies

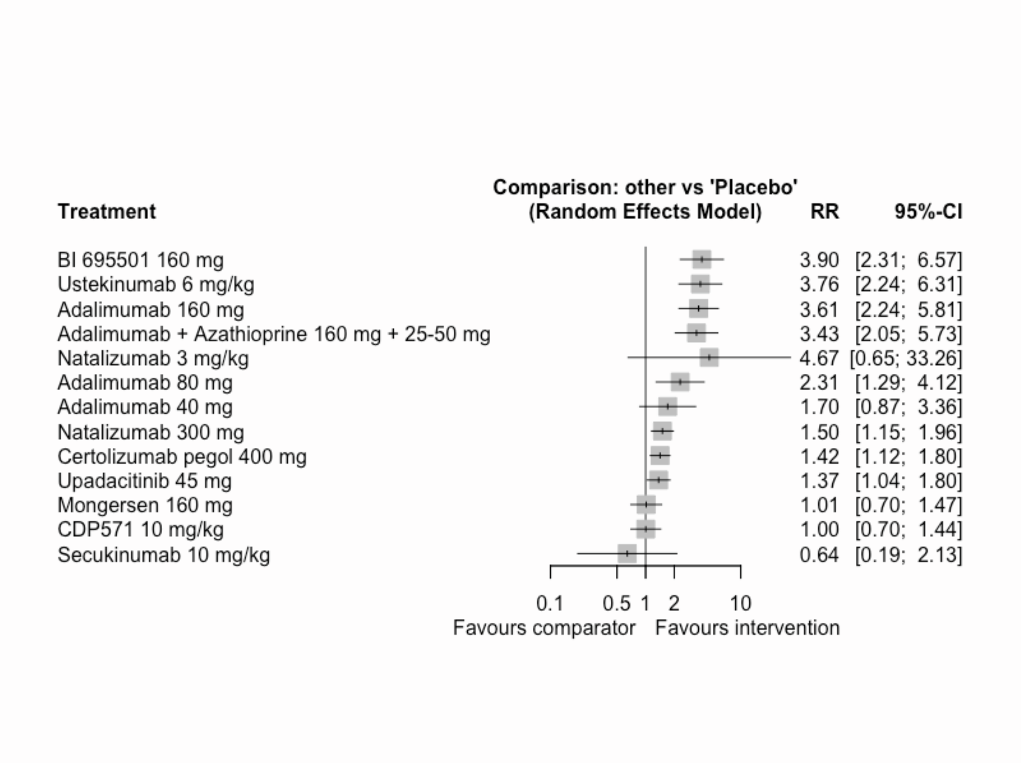

Induction of remission in biologically exposed patients, only 'steroid-free' outcome studies

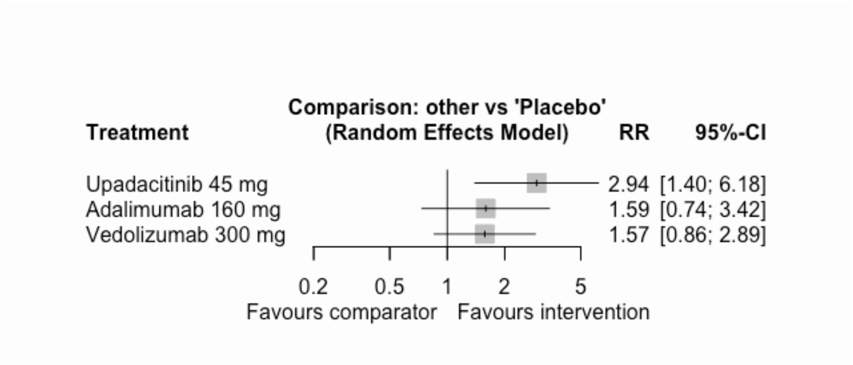

Induction of remission in biologically exposed patients, only 'Non-steroid-free' outcome studies

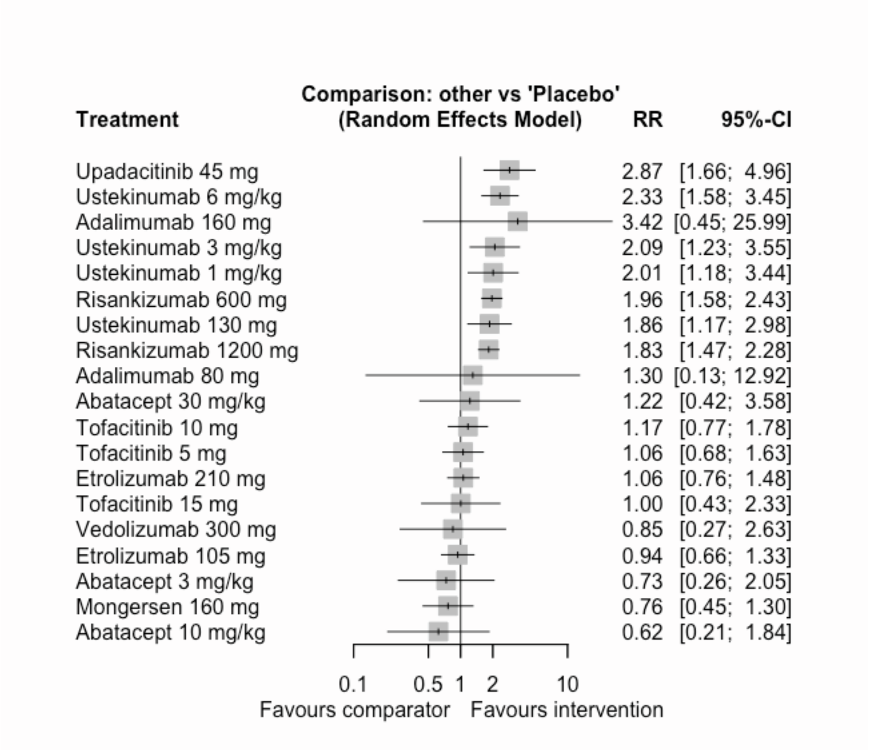

Results of sensitivity analysis excluding studies with a high risk of bias

Induction of remission in biologically naive patients

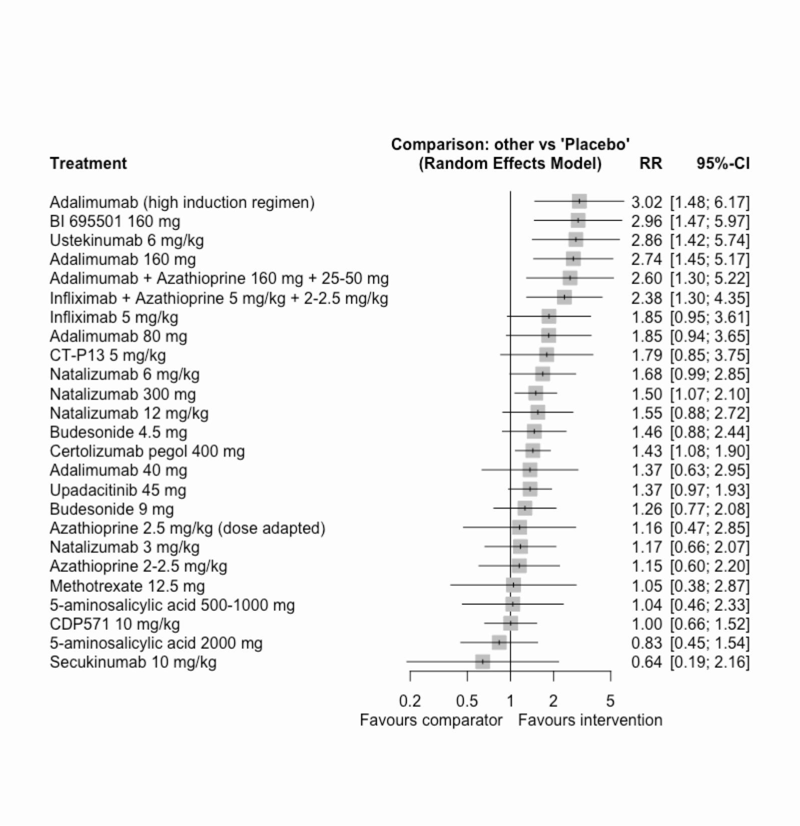

Induction of remission in biologically exposed patients

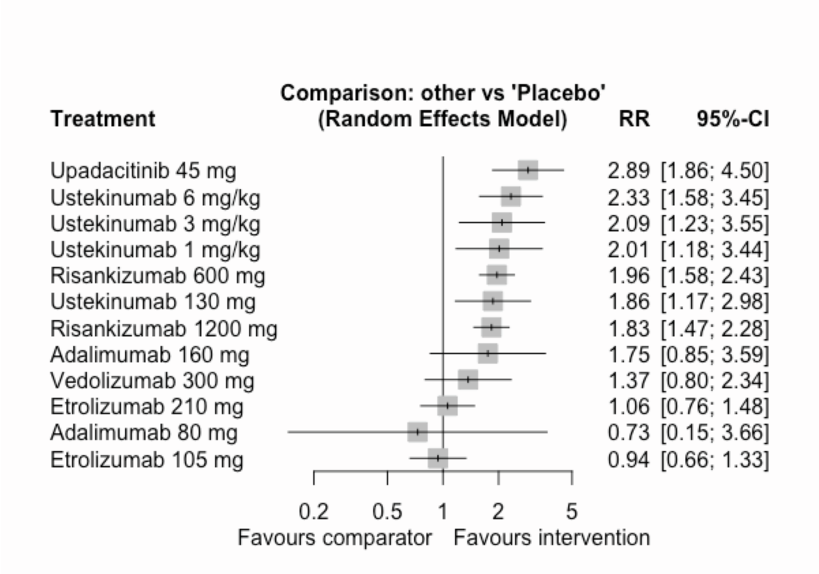

Maintenance of remission in biologically naive patients

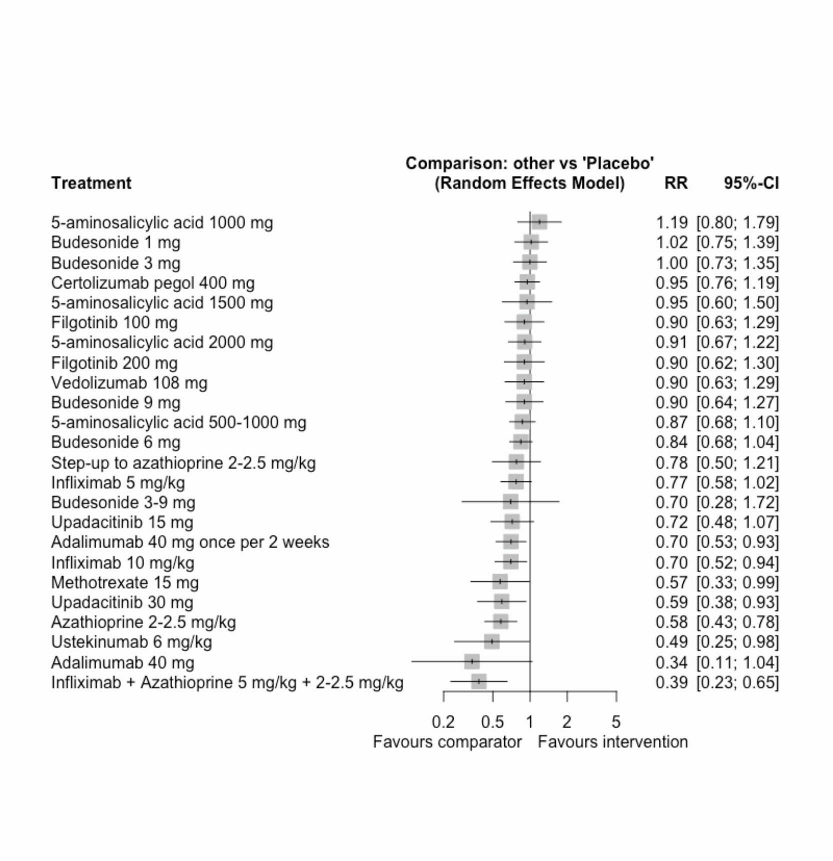

Maintenance of remission in biologically exposed patients

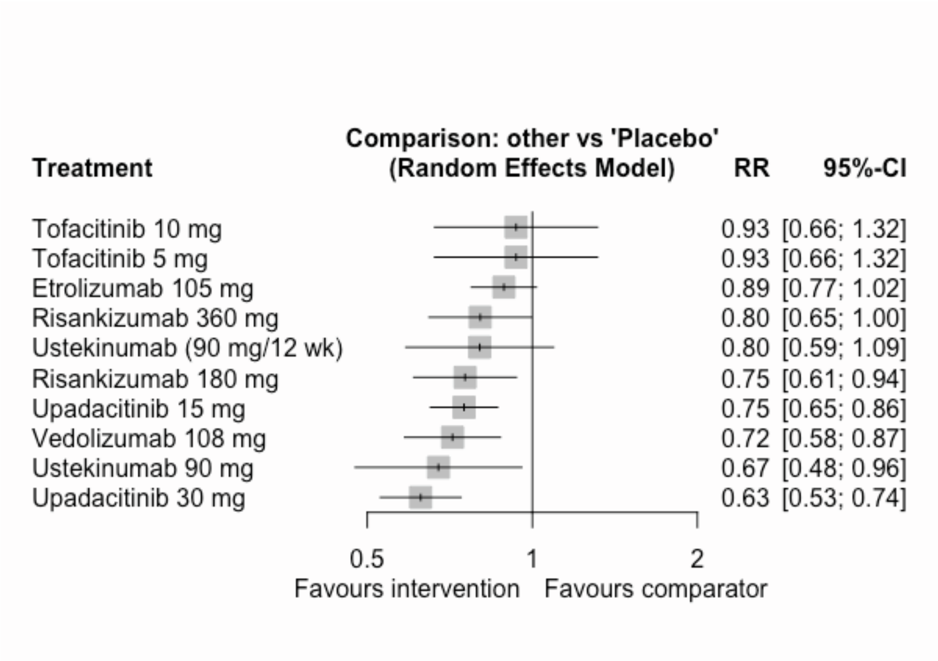

13) AE related discontinuation

Discontinuations due to adverse events

There was substantial (biological-naive) and moderate (biological-exposed) inconsistency between direct and indirect evidence regarding adverse event-related discontinuation of treatments in maintenance trials (I^2 66.3% and 36.8%, respectively). Wide confidence intervals were observed and none of the included interventions was statistically different from placebo. In the biological-naive subgroup, the numerically highest relative risks for discontinuation due to adverse events were observed for the (unregistered) humanized (rather than chimeric) anti-TNF CDP571 (RR = 3.67 [95%CI 0.08-161.07]), azathioprine (RR = 2.65 [95%CI 0.2-35.94]) and methotrexate (RR = 2.7 [95%CI 0.05 – 141.42]). In the biological-exposed subgroup the numerically highest relative risks for discontinuation due to adverse events were for (unregistered) tofacitinib (RR = 4.92 [95%CI 0.49-49.05]) and upadacitinib (RR = 1.64 [95%CI 0.43-6.23]) (Appendix 13).

Other adverse events

Information on adverse events other than adverse event related discontinuations was limited (Appendix 14 and 15). In both populations (biological-naive and -exposed) the absolute numbers of participants with major cardiovascular events and malignancies were very low. In biological-naive participants, malignancies occurred most often with infliximab (1.0% [0.8-1.3%]) and serious infections with vedolizumab (3.9% [2.7-4.9%]). In biological-exposed participants, this was risankizumab (0.3% [0.1-0.4%]) and etrolizumab (5.5% [5.5-5.5%]), respectively.

Biologically naïve

Two studies were not connected to the network, Sandborn (2020) and D'Haens (2022). Candy (1997) was excluded due to zero events. 25 studies were included in the network, with 24 treatments and 4965 patients. Inconsistency was high (I-square = 66.3%) mainly due to

Sands 2004, in which infliximab 5mg/kg had a lower event rate than placebo, which was contrary to the other studies.

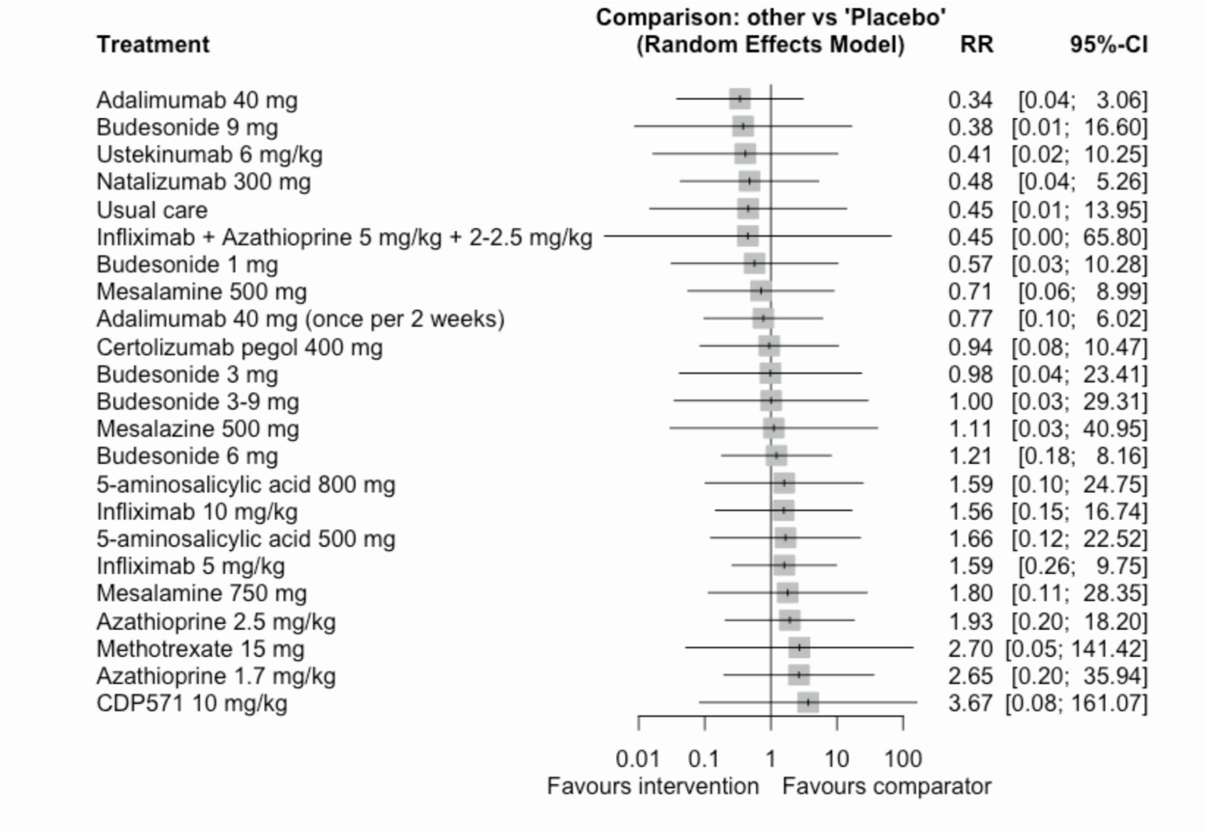

Biologically exposed

Three studies were not connected to the network, Sandborn (2020), van Assche (2012) and Jorgensson (2017). 13 studies were included in the network, with 171 treatments and 3869 patients. Inconsistency was low to moderate (I-square = 36.8)

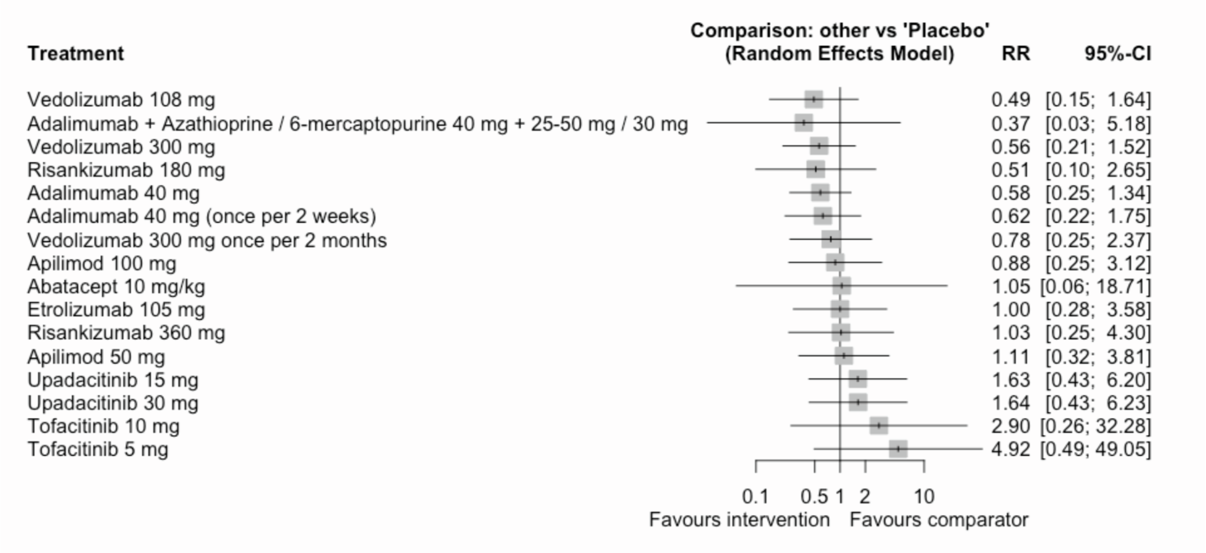

14) Adverse events – median rates

Biologically naïve

Type of adverse event	Intervention	Number of participants with adverse event	Total number of participants analyzed	Median percentage (IQR) of participants with adverse event
Major cardiovascular events	Adalimumab	0	218	0.0 (0.0 to 0.0)
Malignancies	Adalimumab	1	1227	0.0 (0.0 to 0.0)
Malignancies	Certolizumab pegol	2	331	0.6 (0.6 to 0.6)
Malignancies	Infliximab	4	385	1.0 (0.8 to 1.3)
Malignancies	Ustekinumab	0	191	0.0 (0.0 to 0.0)
Malignancies	Vedolizumab	1	308	0.3 (0.2 to 0.5)
Serious infection	Adalimumab	33	1227	2.4 (0.0 to 3.3)
Serious infection	Certolizumab pegol	7	331	2.1 (2.1 to 2.1)
Serious infection	Infliximab	18	523	3.1 (3.0 to 3.6)
Serious infection	Natalizumab	6	214	2.8 (2.8 to 2.8)
Serious infection	Ustekinumab	4	191	2.1 (2.1 to 2.1)
Serious infection	Vedolizumab	19	583	3.9 (2.7 to 4.9)

Biologically exposed

Type of adverse event	Intervention	Number of participants with adverse event	Total number of participants analyzed	Median percentage (IQR) of participants with adverse event
Major cardiovascular events	Risankizumab	2	716	0.3 (0.0 to 0.6)
Major cardiovascular events	Upadacitinib	1	628	0.0 (0.0 to 0.0)
Malignancies	Abatacept	0	44	0.0 (0.0 to 0.0)
Malignancies	Adalimumab	0	157	0.0 (0.0 to 0.0)
Malignancies	Risankizumab	1	358	0.3 (0.1 to 0.4)
Malignancies	Tofacitinib	0	121	0.0 (0.0 to 0.0)
Malignancies	Upadacitinib	5	628	0.2 (0.0 to 0.8)
Serious infection	Abatacept	1	44	2.3 (2.3 to 2.3)
Serious infection	Adalimumab	5	157	3.0 (2.2 to 3.8)
Serious infection	Etrolizumab	12	217	5.5 (5.5 to 5.5)
Serious infection	Risankizumab	13	358	3.6 (3.2 to 4.1)
Serious infection	Tofacitinib	5	121	4.1 (3.7 to 4.6)
Serious infection	Upadacitinib	28	628	2.9 (0.4 to 5.3)
Serious infection	Ustekinumab	10	263	3.8 (3.0 to 4.5)

15) Adverse events – rates per study

Biologically naïve

Study reference	Intervention	Type of adverse event	Follow-up time	Number of participants with adverse event	Total number of participants analyzed	Percentage of participants with adverse event
D'Haens 2022	Adalimumab 40 mg (once per 2 weeks)	Major cardiovascular events	44 weeks	0	109	0.00%
D'Haens 2022	Adalimumab 40 mg (once per 2 weeks)	Major cardiovascular events	44 weeks	0	109	0.00%
Colombel 2009	Adalimumab 40 mg	Malignancies	52 weeks	0	257	0.00%
Colombel 2009	Adalimumab 40 mg (once per 2 weeks)	Malignancies	52 weeks	0	260	0.00%
D'Haens 2022	Adalimumab 40 mg (once per 2 weeks)	Malignancies	44 weeks	0	109	0.00%
D'Haens 2022	Adalimumab 40 mg (once per 2 weeks)	Malignancies	44 weeks	0	109	0.00%
Rutgeerts 2004	Infliximab 10 mg/kg	Malignancies	52 weeks	1	192	0.52%
Rutgeerts 2004	Infliximab 5 mg/kg	Malignancies	52 weeks	3	193	1.55%
Rutgeerts 2012	Adalimumab 40 mg (once per 2 weeks)	Malignancies	52 weeks	0	64	0.00%
Sandborn 2007	Adalimumab 40 mg	Malignancies	52 weeks	0	18	0.00%
Sandborn 2007	Adalimumab 40 mg (once per 2 weeks)	Malignancies	52 weeks	0	19	0.00%
Sandborn 2007	Certolizumab pegol 400 mg	Malignancies	26 weeks	2	331	0.60%
Sandborn 2013	Adalimumab 40 mg	Malignancies	52 weeks	0	86	0.00%
Sandborn 2013	Adalimumab 40 mg (once per 2 weeks)	Malignancies	52 weeks	0	85	0.00%
Sandborn 2013	Vedolizumab 300 mg	Malignancies	46 weeks	0	154	0.00%

Sandborn 2013	Vedolizumab 300 mg (once per 2 months)	Malignancies	46 weeks	1	154	0.65%
Sands 2022	Adalimumab 40 mg (once per 2 weeks)	Malignancies	52 weeks	1	195	0.51%
Sands 2022	Ustekinumab 6 mg/kg	Malignancies	52 weeks	0	191	0.00%
Watanabe 2012	Adalimumab 40 mg (once per 2 weeks)	Malignancies	52 weeks	0	25	0.00%
Colombel 2009	Adalimumab 40 mg	Serious infection	52 weeks	12	257	4.67%
Colombel 2009	Adalimumab 40 mg (once per 2 weeks)	Serious infection	52 weeks	10	260	3.85%
D'Haens 2022	Adalimumab 40 mg (once per 2 weeks)	Serious infection	44 weeks	0	109	0.00%
D'Haens 2022	Adalimumab 40 mg (once per 2 weeks)	Serious infection	44 weeks	3	109	2.75%
Hanauer 2002	Infliximab 10 mg/kg	Serious infection	52 weeks	6	192	3.12%
Hanauer 2002	Infliximab 5 mg/kg	Serious infection	52 weeks	8	193	4.15%
Rutgeerts 2012	Adalimumab 40 mg (once per 2 weeks)	Serious infection	52 weeks	0	64	0.00%
Sandborn 2005	Natalizumab 300 mg	Serious infection	48 weeks	6	214	2.80%
Sandborn 2007	Adalimumab 40 mg	Serious infection	52 weeks	0	18	0.00%
Sandborn 2007	Adalimumab 40 mg (once per 2 weeks)	Serious infection	52 weeks	0	19	0.00%
Sandborn 2007	Certolizumab pegol 400 mg	Serious infection	26 weeks	7	331	2.11%
Sandborn 2013	Adalimumab 40 mg	Serious infection	52 weeks	0	86	0.00%
Sandborn 2013	Adalimumab 40 mg (once per 2 weeks)	Serious infection	52 weeks	2	85	2.35%
Sandborn 2013	Vedolizumab 300 mg	Serious infection	46 weeks	9	154	5.84%
Sandborn 2013	Vedolizumab 300 mg (once per 2 months)	Serious infection	46 weeks	6	154	3.90%
Sands 2004	Infliximab 5 mg/kg	Serious infection	40 weeks	4	138	2.90%

Sands 2022	Adalimumab 40 mg (once per 2 weeks)	Serious infection	52 weeks	5	195	2.56%
Sands 2022	Ustekinumab 6 mg/kg	Serious infection	52 weeks	4	191	2.09%
Vermeire 2022	Vedolizumab 108 mg	Serious infection	46 weeks	4	275	1.45%
Watanabe 2012	Adalimumab 40 mg (once per 2 weeks)	Serious infection	52 weeks	1	25	4.00%

Biologically exposed

Study reference	Intervention	Type of adverse event	Follow-up time	Number of participants with adverse event	Total number of participants analyzed	Percentage of participants with adverse event
Ferrante 2022	Risankizumab 180 mg	Major cardiovascular events	52 weeks	0	179	0.00%
Ferrante 2022	Risankizumab 180 mg	Major cardiovascular events	52 weeks	0	179	0.00%
Ferrante 2022	Risankizumab 360 mg	Major cardiovascular events	52 weeks	1	179	0.56%
Ferrante 2022	Risankizumab 360 mg	Major cardiovascular events	52 weeks	1	179	0.56%
Loftus 2023	Upadacitinib 15 mg	Major cardiovascular events	52 weeks	0	221	0.00%
Loftus 2023	Upadacitinib 30 mg	Major cardiovascular events	52 weeks	0	229	0.00%
Sandborn 2020	Upadacitinib 24 mg (once per day)	Major cardiovascular events	36 weeks	0	36	0.00%
Sandborn 2020	Upadacitinib 3 mg (two times per day)	Major cardiovascular events	36 weeks	1	60	1.67%
Sandborn 2020	Upadacitinib 6 mg (two times per day)	Major cardiovascular events	36 weeks	0	23	0.00%
Sandborn 2020	Upadaticinib 12 mg (two times per day)	Major cardiovascular events	36 weeks	0	59	0.00%
Ferrante 2022	Risankizumab 180 mg	Malignancies	52 weeks	0	179	0.00%
Ferrante 2022	Risankizumab 360 mg	Malignancies	52 weeks	1	179	0.56%
Loftus 2023	Upadacitinib 15 mg	Malignancies	52 weeks	1	221	0.45%
Loftus 2023	Upadacitinib 30 mg	Malignancies	52 weeks	2	229	0.87%
Panés 2017	Tofacitinib 10 mg	Malignancies	26 weeks	0	61	0.00%
Panés 2017	Tofacitinib 5 mg	Malignancies	26 weeks	0	60	0.00%
Sandborn 2012	Abatacept 10 mg/kg	Malignancies	40 weeks	0	44	0.00%
Sandborn 2013	Adalimumab 40 mg	Malignancies	52 weeks	0	71	0.00%
Sandborn 2013	Adalimumab 40 mg (once per 2 weeks)	Malignancies	52 weeks	0	86	0.00%

Sandborn 2020	Upadacitinib 24 mg (once per day)	Malignancies	36 weeks	0	36	0.00%
Sandborn 2020	Upadacitinib 3 mg (two times per day)	Malignancies	36 weeks	0	60	0.00%
Sandborn 2020	Upadacitinib 6 mg (two times per day)	Malignancies	36 weeks	0	23	0.00%
Sandborn 2020	Upadaticinib 12 mg (two times per day)	Malignancies	36 weeks	2	59	3.39%
Feagan 2016	Ustekinumab (90 mg/12 wk)	Serious infection	44 weeks	7	132	5.30%
Feagan 2016	Ustekinumab 90 mg	Serious infection	44 weeks	3	131	2.29%
Ferrante 2022	Risankizumab 180 mg	Serious infection	52 weeks	5	179	2.79%
Ferrante 2022	Risankizumab 360 mg	Serious infection	52 weeks	8	179	4.47%
Loftus 2023	Upadacitinib 15 mg	Serious infection	52 weeks	9	221	4.07%
Loftus 2023	Upadacitinib 30 mg	Serious infection	52 weeks	13	229	5.68%
Panés 2017	Tofacitinib 10 mg	Serious infection	26 weeks	2	61	3.28%
Panés 2017	Tofacitinib 5 mg	Serious infection	26 weeks	3	60	5.00%
Sandborn 2012	Abatacept 10 mg/kg	Serious infection	40 weeks	1	44	2.27%
Sandborn 2013	Adalimumab 40 mg	Serious infection	52 weeks	1	71	1.41%
Sandborn 2013	Adalimumab 40 mg (once per 2 weeks)	Serious infection	52 weeks	4	86	4.65%
Sandborn 2020	Upadacitinib 24 mg (once per day)	Serious infection	36 weeks	0	36	0.00%
Sandborn 2020	Upadacitinib 3 mg (two times per day)	Serious infection	36 weeks	5	60	8.33%
Sandborn 2020	Upadacitinib 6 mg (two times per day)	Serious infection	36 weeks	0	23	0.00%
Sandborn 2020	Upadaticinib 12 mg (two times per day)	Serious infection	36 weeks	1	59	1.69%
Sandborn 2023	Etrolizumab 105 mg	Serious infection	52 weeks	12	217	5.53%

16) CINeMA approach and results

Approach

Indirectness Scoring: We assessed indirectness based on three factors with predefined thresholds:

- Biological exposure: Studies were downgraded if naive groups had >20% prior biological exposure or exposed groups had <80% exposure
- Follow-up duration: Downgraded if induction follow-up was <4 weeks (none found) or maintenance follow-up was <30 or >100 weeks
- Composite outcomes: Downgraded if CDAI was part of a composite endpoint requiring additional clinical/laboratory criteria
- Maximum downgrade: Limited to 1 level (score 1=no concerns, 2=downgraded once)

CINEMA Configuration:

- Model: Random effects with risk ratios (fixed effects for maintenance exposed)
- Reference treatments: IFX+AZA (naive induction/maintenance), ustekinumab 6mg/kg (exposed induction), ustekinumab 90mg (exposed maintenance)
- Within-study bias: Average risk-of-bias rule (1=low, 2=some concerns, 3=high risk)
- Reporting bias: Not suspected for all comparisons
- Indirectness: Average indirectness rule
- Clinical significance threshold: RR 1.25 for imprecision, heterogeneity, and incoherence assessments

Confidence Rating: Applied systematic downgrading without subjective judgment—some concerns = 1 downgrade, major concerns = 2 downgrades. Incoherence concerns were ignored for comparisons with only indirect evidence.

Induction biologically naive

Comparison	Number of studies	Within-study bias	Reporting bias	Indirectness	Imprecision	Heterogeneity	Incoherence	Confidence rating
**Azathioprine 2-2.5 mg/kg:Infliximab + Azathioprine 5 mg/kg + 2-2.5 mg/kg**	1	Some concerns	Low risk	No concerns	No concerns	No concerns	No concerns	Moderate
**Budesonide 9 mg:Infliximab + Azathioprine 5 mg/kg + 2-2.5 mg/kg**	1	Some concerns	Low risk	Some concerns	No concerns	No concerns	No concerns	Low
**Infliximab 5 mg/kg:Infliximab + Azathioprine 5 mg/kg + 2-2.5 mg/kg**	1	Some concerns	Low risk	No concerns	Some concerns	No concerns	No concerns	Low
**5-aminosalicylic acid 1000 mg:Infliximab + Azathioprine 5 mg/kg + 2-2.5 mg/kg**	0	Some concerns	Low risk	No concerns	Major concerns	No concerns	No concerns	Very low
**5-aminosalicylic acid 1500 mg:Infliximab + Azathioprine 5 mg/kg + 2-2.5 mg/kg**	0	Major concerns	Low risk	No concerns	No concerns	No concerns	No concerns	Low
**5-aminosalicylic acid 2000 mg:Infliximab + Azathioprine 5 mg/kg + 2-2.5 mg/kg**	0	Some concerns	Low risk	No concerns	No concerns	No concerns	No concerns	Moderate
**5-aminosalicylic acid 500-1000 mg:Infliximab + Azathioprine 5 mg/kg + 2-2.5 mg/kg**	0	Some concerns	Low risk	No concerns	No concerns	Some concerns	No concerns	Low
**Adalimumab (high induction regimen):Infliximab + Azathioprine 5 mg/kg + 2-2.5 mg/kg**	0	Some concerns	Low risk	No concerns	Major concerns	No concerns	No concerns	Very low
**Adalimumab + Azathioprine 160 mg + 25-50 mg:Infliximab + Azathioprine 5 mg/kg + 2-2.5 mg/kg**	0	Some concerns	Low risk	No concerns	Major concerns	No concerns	No concerns	Very low
**Adalimumab 160 mg:Infliximab + Azathioprine 5 mg/kg + 2-2.5 mg/kg**	0	Some concerns	Low risk	No concerns	Major concerns	No concerns	No concerns	Very low
**Adalimumab 40 mg:Infliximab + Azathioprine 5 mg/kg + 2-2.5 mg/kg**	0	Some concerns	Low risk	No concerns	Major concerns	No concerns	No concerns	Very low
**Adalimumab 80 mg:Infliximab + Azathioprine 5 mg/kg + 2-2.5 mg/kg**	0	Some concerns	Low risk	No concerns	Major concerns	No concerns	No concerns	Very low
**Azathioprine 2.5 mg/kg (dose adapted):Infliximab + Azathioprine 5 mg/kg + 2-2.5 mg/kg**	0	Some concerns	Low risk	No concerns	No concerns	Some concerns	No concerns	Low
**Azathioprine 40 mg/kg:Infliximab + Azathioprine 5 mg/kg + 2-2.5 mg/kg**	0	Some concerns	Low risk	No concerns	Some concerns	Some concerns	No concerns	Very low
**BI 695501 160 mg:Infliximab + Azathioprine 5 mg/kg + 2-2.5 mg/kg**	0	Some concerns	Low risk	No concerns	Major concerns	No concerns	No concerns	Very low
**Budesonide 1.5 mg:Infliximab + Azathioprine 5 mg/kg + 2-2.5 mg/kg**	0	Some concerns	Low risk	No concerns	No concerns	No concerns	No concerns	Moderate

**Budesonide 2 mg:Infliximab + Azathioprine 5 mg/kg + 2-2.5 mg/kg**	0	Major concerns	Low risk	No concerns	No concerns	No concerns	No concerns	Low
**Budesonide 3 mg:Infliximab + Azathioprine 5 mg/kg + 2-2.5 mg/kg**	0	Some concerns	Low risk	No concerns	No concerns	No concerns	No concerns	Moderate
**Budesonide 4.5 mg:Infliximab + Azathioprine 5 mg/kg + 2-2.5 mg/kg**	0	Some concerns	Low risk	No concerns	Some concerns	No concerns	No concerns	Low
**Budesonide 6 mg:Infliximab + Azathioprine 5 mg/kg + 2-2.5 mg/kg**	0	Major concerns	Low risk	No concerns	Some concerns	No concerns	No concerns	Very low
**Budesonide 7.5 mg:Infliximab + Azathioprine 5 mg/kg + 2-2.5 mg/kg**	0	Some concerns	Low risk	No concerns	Some concerns	No concerns	No concerns	Low
**CDP571 10 mg/kg:Infliximab + Azathioprine 5 mg/kg + 2-2.5 mg/kg**	0	Some concerns	Low risk	No concerns	No concerns	No concerns	No concerns	Moderate
**CT-P13 5 mg/kg:Infliximab + Azathioprine 5 mg/kg + 2-2.5 mg/kg**	0	Some concerns	Low risk	No concerns	Some concerns	No concerns	No concerns	Low
**Certolizumab pegol 400 mg:Infliximab + Azathioprine 5 mg/kg + 2-2.5 mg/kg**	0	Some concerns	Low risk	No concerns	No concerns	No concerns	No concerns	Moderate
**Infliximab + Azathioprine 5 mg/kg + 2-2.5 mg/kg:Methotrexate 12.5 mg**	0	Some concerns	Low risk	No concerns	Some concerns	Some concerns	No concerns	Very low
**Infliximab + Azathioprine 5 mg/kg + 2-2.5 mg/kg:Methotrexate 25 mg**	0	Some concerns	Low risk	No concerns	Some concerns	Some concerns	No concerns	Very low
**Infliximab + Azathioprine 5 mg/kg + 2-2.5 mg/kg:Mongersen 160 mg**	0	Some concerns	Low risk	No concerns	No concerns	No concerns	No concerns	Moderate
**Infliximab + Azathioprine 5 mg/kg + 2-2.5 mg/kg:Natalizumab 12 mg/kg**	0	Some concerns	Low risk	No concerns	Some concerns	Some concerns	No concerns	Very low
**Infliximab + Azathioprine 5 mg/kg + 2-2.5 mg/kg:Natalizumab 3 mg/kg**	0	Some concerns	Low risk	No concerns	No concerns	No concerns	No concerns	Moderate
**Infliximab + Azathioprine 5 mg/kg + 2-2.5 mg/kg:Natalizumab 300 mg**	0	Some concerns	Low risk	No concerns	No concerns	Some concerns	No concerns	Low
**Infliximab + Azathioprine 5 mg/kg + 2-2.5 mg/kg:Natalizumab 6 mg/kg**	0	Some concerns	Low risk	No concerns	Some concerns	Some concerns	No concerns	Very low
**Infliximab + Azathioprine 5 mg/kg + 2-2.5 mg/kg:Placebo**	0	Some concerns	Low risk	No concerns	No concerns	No concerns	No concerns	Moderate
**Infliximab + Azathioprine 5 mg/kg + 2-2.5 mg/kg:Prednisolone 40 mg**	0	Some concerns	Low risk	No concerns	Some concerns	No concerns	No concerns	Low
**Infliximab + Azathioprine 5 mg/kg + 2-2.5 mg/kg:Secukinumab 10 mg/kg**	0	Some concerns	Low risk	No concerns	No concerns	Some concerns	No concerns	Low

**Infliximab + Azathioprine 5 mg/kg + 2-2.5 mg/kg:Upadacitinib 45 mg**	0	Some concerns	Low risk	No concerns	No concerns	No concerns	No concerns	Moderate
**Infliximab + Azathioprine 5 mg/kg + 2-2.5 mg/kg:Ustekinumab 6 mg/kg**	0	Some concerns	Low risk	No concerns	Major concerns	No concerns	No concerns	Very low

Induction biologically exposed

Comparison	Number of studies	Within-study bias	Reporting bias	Indirectness	Imprecision	Heterogeneity	Incoherence	Confidence rating
**Placebo:Ustekinumab 6 mg/kg**	2	No concerns	Low risk	No concerns	No concerns	No concerns	No concerns	High
**Ustekinumab 1 mg/kg:Ustekinumab 6 mg/kg**	1	Some concerns	Low risk	No concerns	Major concerns	No concerns	No concerns	Very low
**Ustekinumab 130 mg:Ustekinumab 6 mg/kg**	1	No concerns	Low risk	No concerns	Some concerns	Some concerns	No concerns	Low
**Ustekinumab 3 mg/kg:Ustekinumab 6 mg/kg**	1	Some concerns	Low risk	No concerns	Major concerns	No concerns	No concerns	Very low
**Abatacept 10 mg/kg:Ustekinumab 6 mg/kg**	0	Some concerns	Low risk	Some concerns	No concerns	Some concerns	No concerns	Very low
**Abatacept 3 mg/kg:Ustekinumab 6 mg/kg**	0	Some concerns	Low risk	Some concerns	No concerns	Major concerns	No concerns	Very low
**Abatacept 30 mg/kg:Ustekinumab 6 mg/kg**	0	Some concerns	Low risk	Some concerns	Major concerns	No concerns	No concerns	Very low
**Adalimumab 160 mg:Ustekinumab 6 mg/kg**	0	No concerns	Low risk	No concerns	Major concerns	No concerns	No concerns	Low
**Adalimumab 80 mg:Ustekinumab 6 mg/kg**	0	Some concerns	Low risk	No concerns	Major concerns	No concerns	No concerns	Very low
**Etrolizumab 105 mg:Ustekinumab 6 mg/kg**	0	No concerns	Low risk	No concerns	No concerns	No concerns	No concerns	High
**Etrolizumab 210 mg:Ustekinumab 6 mg/kg**	0	No concerns	Low risk	No concerns	No concerns	No concerns	No concerns	High
**Mongersen 160 mg:Ustekinumab 6 mg/kg**	0	Some concerns	Low risk	No concerns	No concerns	No concerns	No concerns	Moderate
**Risankizumab 1200 mg:Ustekinumab 6 mg/kg**	0	No concerns	Low risk	No concerns	Some concerns	Some concerns	No concerns	Low
**Risankizumab 600 mg:Ustekinumab 6 mg/kg**	0	No concerns	Low risk	No concerns	Major concerns	No concerns	No concerns	Low
**Tofacitinib 10 mg:Ustekinumab 6 mg/kg**	0	Some concerns	Low risk	No concerns	No concerns	Some concerns	No concerns	Low
**Tofacitinib 15 mg:Ustekinumab 6 mg/kg**	0	Some concerns	Low risk	No concerns	Some concerns	Some concerns	No concerns	Very low
**Tofacitinib 5 mg:Ustekinumab 6 mg/kg**	0	Some concerns	Low risk	No concerns	No concerns	No concerns	No concerns	Moderate
**Upadacitinib 45 mg:Ustekinumab 6 mg/kg**	0	No concerns	Low risk	No concerns	Major concerns	No concerns	No concerns	Low

**Ustekinumab 6 mg/kg:Vedolizumab 300 mg**	0	Some concerns	Low risk	No concerns	Some concerns	Some concerns	No concerns	Very low

Maintenance biologically naive

Comparison	Number of studies	Within-study bias	Reporting bias	Indirectness	Imprecision	Heterogeneity	Incoherence	Confidence rating
**Azathioprine 2-2.5 mg/kg:Infliximab + Azathioprine 5 mg/kg + 2-2.5 mg/kg**	1	Some concerns	Low risk	Some concerns	Some concerns	No concerns	Major concerns	Very low
**5-aminosalicylic acid 1000 mg:Infliximab + Azathioprine 5 mg/kg + 2-2.5 mg/kg**	0	Some concerns	Low risk	Some concerns	No concerns	No concerns	Major concerns	Low
**5-aminosalicylic acid 1500 mg:Infliximab + Azathioprine 5 mg/kg + 2-2.5 mg/kg**	0	Some concerns	Low risk	No concerns	No concerns	Some concerns	Major concerns	Low
**5-aminosalicylic acid 2000 mg:Infliximab + Azathioprine 5 mg/kg + 2-2.5 mg/kg**	0	Some concerns	Low risk	Some concerns	No concerns	No concerns	Major concerns	Low
**5-aminosalicylic acid 500-1000 mg:Infliximab + Azathioprine 5 mg/kg + 2-2.5 mg/kg**	0	Some concerns	Low risk	Some concerns	No concerns	No concerns	Major concerns	Low
**Adalimumab 40 mg:Infliximab + Azathioprine 5 mg/kg + 2-2.5 mg/kg**	0	Some concerns	Low risk	Some concerns	No concerns	Some concerns	Major concerns	Very low
**Adalimumab 40 mg once per 2 weeks:Infliximab + Azathioprine 5 mg/kg + 2-2.5 mg/kg**	0	Some concerns	Low risk	Some concerns	Some concerns	No concerns	Major concerns	Very low
**Budesonide 1 mg:Infliximab + Azathioprine 5 mg/kg + 2-2.5 mg/kg**	0	Some concerns	Low risk	No concerns	No concerns	No concerns	Major concerns	Moderate
**Budesonide 3 mg:Infliximab + Azathioprine 5 mg/kg + 2-2.5 mg/kg**	0	Some concerns	Low risk	Some concerns	No concerns	No concerns	Major concerns	Low
**Budesonide 3-9 mg:Infliximab + Azathioprine 5 mg/kg + 2-2.5 mg/kg**	0	Some concerns	Low risk	Some concerns	Major concerns	No concerns	Major concerns	Very low
**Budesonide 6 mg:Infliximab + Azathioprine 5 mg/kg + 2-2.5 mg/kg**	0	Some concerns	Low risk	Some concerns	No concerns	No concerns	Major concerns	Low
**Budesonide 9 mg:Infliximab + Azathioprine 5 mg/kg + 2-2.5 mg/kg**	0	Some concerns	Low risk	No concerns	No concerns	No concerns	Major concerns	Moderate
**Certolizumab pegol 400 mg:Infliximab + Azathioprine 5 mg/kg + 2-2.5 mg/kg**	0	Some concerns	Low risk	Some concerns	No concerns	No concerns	Major concerns	Low
**Filgotinib 100 mg:Infliximab + Azathioprine 5 mg/kg + 2-2.5 mg/kg**	0	Some concerns	Low risk	Some concerns	No concerns	No concerns	Major concerns	Low
**Filgotinib 200 mg:Infliximab + Azathioprine 5 mg/kg + 2-2.5 mg/kg**	0	Some concerns	Low risk	Some concerns	No concerns	No concerns	Major concerns	Low
**Infliximab 10 mg/kg:Infliximab + Azathioprine 5 mg/kg + 2-2.5 mg/kg**	0	Some concerns	Low risk	Some concerns	Some concerns	No concerns	Major concerns	Very low

**Infliximab 5 mg/kg:Infliximab + Azathioprine 5 mg/kg + 2-2.5 mg/kg**	0	Some concerns	Low risk	Some concerns	No concerns	Some concerns	Major concerns	Very low
**Infliximab + Azathioprine 5 mg/kg + 2-2.5 mg/kg:Methotrexate 15 mg**	0	Some concerns	Low risk	No concerns	Major concerns	No concerns	Major concerns	Very low
**Infliximab + Azathioprine 5 mg/kg + 2-2.5 mg/kg:Placebo**	0	Some concerns	Low risk	Some concerns	No concerns	No concerns	Major concerns	Low
**Infliximab + Azathioprine 5 mg/kg + 2-2.5 mg/kg:Step-up to azathioprine 2-2.5 mg/kg**	0	Some concerns	Low risk	Some concerns	No concerns	Some concerns	Major concerns	Very low
**Infliximab + Azathioprine 5 mg/kg + 2-2.5 mg/kg:Upadacitinib 15 mg**	0	Some concerns	Low risk	No concerns	Some concerns	Some concerns	Major concerns	Very low
**Infliximab + Azathioprine 5 mg/kg + 2-2.5 mg/kg:Upadacitinib 30 mg**	0	Some concerns	Low risk	No concerns	Major concerns	No concerns	Major concerns	Very low
**Infliximab + Azathioprine 5 mg/kg + 2-2.5 mg/kg:Ustekinumab 90 mg**	0	Some concerns	Low risk	Some concerns	Major concerns	No concerns	Major concerns	Very low
**Infliximab + Azathioprine 5 mg/kg + 2-2.5 mg/kg:Vedolizumab 108 mg**	0	Some concerns	Low risk	No concerns	No concerns	No concerns	Major concerns	Moderate

Maintenance biologically exposed

Comparison	Number of studies	Within-study bias	Reporting bias	Indirectness	Imprecision	Heterogeneity	Incoherence	Confidence rating
**Placebo:Ustekinumab 90 mg**	1	No concerns	Low risk	Some concerns	No concerns	Major concerns	Major concerns	Very low
**Ustekinumab 90 mg:Ustekinumab (90 mg/12 wk)**	1	No concerns	Low risk	Some concerns	Some concerns	Some concerns	Major concerns	Very low
**Abatacept 10 mg/kg:Ustekinumab 90 mg**	0	Some concerns	Low risk	Some concerns	Some concerns	Some concerns	Major concerns	Very low
**Etrolizumab 105 mg:Ustekinumab 90 mg**	0	No concerns	Low risk	Some concerns	Some concerns	Some concerns	Major concerns	Very low
**Risankizumab 180 mg:Ustekinumab 90 mg**	0	No concerns	Low risk	Some concerns	Major concerns	No concerns	Major concerns	Very low
**Risankizumab 360 mg:Ustekinumab 90 mg**	0	No concerns	Low risk	Some concerns	Major concerns	No concerns	Major concerns	Very low
**Tofacitinib 10 mg:Ustekinumab 90 mg**	0	No concerns	Low risk	Some concerns	Some concerns	Some concerns	Major concerns	Very low
**Tofacitinib 5 mg:Ustekinumab 90 mg**	0	No concerns	Low risk	Some concerns	Some concerns	Some concerns	Major concerns	Very low
**Upadacitinib 15 mg:Ustekinumab 90 mg**	0	No concerns	Low risk	Some concerns	Major concerns	No concerns	Major concerns	Very low
**Upadacitinib 30 mg:Ustekinumab 90 mg**	0	No concerns	Low risk	Some concerns	Major concerns	No concerns	Major concerns	Very low
**Ustekinumab 90 mg:Vedolizumab 108 mg**	0	No concerns	Low risk	Some concerns	Major concerns	No concerns	Major concerns	Very low
